# Afrotropical Cynipoidea (Hymenoptera)

**DOI:** 10.3897/zookeys.493.6353

**Published:** 2015-04-01

**Authors:** Simon van Noort, Matthew L. Buffington, Mattias Forshage

**Affiliations:** 1 Natural History Department, Iziko South African Museum, PO Box 61, Cape Town, 8000, South Africa; 2 Department of Biological Sciences, University of Cape Town, Private Bag, Rondebosch, 7701, South Africa; 3 Systematic Entomology Lab, USDA, c/o Smithsonian NMNH, 10th & Constitution Ave NW, Washington DC 20013; 4 Swedish Museum of Natural History, Department of Entomology, Box 50007, SE-104 05 Stockholm, Sweden

**Keywords:** Africa, Afrotropical, Cynipidae, Cynipoidea, Figitidae, Ibaliidae, identification key, Liopteridae, Madagascar

## Abstract

The Afrotropical Cynipoidea are represented by 306 described species and 54 genera in four families: Cynipidae, Figitidae, Liopteridae and Ibaliidae, the latter represented by a single introduced species. Seven of these genera are only represented by undescribed species in the region. Seven new genus-level synonymies, one genus resurrected from synonymy, 54 new combinations, one combination reinstated, and one new replacement name are presented. We provide identification keys to the families, subfamilies and genera of cynipoid wasps occurring in the Afrotropical region (Africa south of the Sahara, including Madagascar and southern Arabian Peninsula). Online interactive Lucid Phoenix and Lucid matrix keys are available at: http://www.waspweb.org/Cynipoidea/Keys/index.htm. An overview of the biology and checklists of species for each genus are provided. This paper constitutes the first contributory chapter to the book on Afrotropical Hymenoptera.

## Introduction

The Afrotropical Cynipoidea are taxonomically and biologically poorly known, a situation typical for wasp taxa from this region. The lack of knowledge in cynipoid systematics is exemplified by the recent revisions that have increased the number of described species (Pycnostigminae: Buffington and van Noort 2007; *Anacharoides*: Buffington and van Noort 2009; *Stentorceps*: Nielsen and Buffington 2011) as well as the description of a new genus (Buffington 2012). As a consequence of the under-documentation of the region’s diversity, the process of unraveling the biology of the Afrotropical cynipoid wasps is also in its infancy. Some headway has been made with a recent biological study of the cynipid *Rhoophilus loewi* Mayr, 1881 (van Noort et al. 2006) and the discovery of two true indigenous gall formers, *Phanacis neserorum* Melika and Prinsloo (2007), and *Qwaqwaia scolopiae* Liljeblad, Nieves-Aldrey & Melika (2011), the latter meriting the description of a new genus and establishment of a new tribe (Liljeblad et al. 2011). However, the majority of the species and generic level diversity within the Afrotropical cynipoids can be found within the eucoiline Figitidae, a monophyletic group that are primary koinobiont endoparasitoids of muscomorphan Diptera (Ronquist 1999, Fontal-Cazalla et al. 2002, Buffington et al. 2007, 2012).

Cynipoid knowledge was first systematised in a scientific form in Europe. To the systematic entomologists in Linnaeus's time, most cynipoids were too small to warrant recognition, and it was mostly the large-sized ibaliids and the cynipids (accessible via their obvious galls) that were studied in the early days. The first more systematical treatment of figitids was that of Westwood in the 1830s, followed by Hartig, Dahlbom, Giraud, Thomson and Förster, and eventually the three massively productive authors of the previous turn of century: Ashmead, Cameron and Kieffer. Of these, Peter Cameron and Jean-Jacques Kieffer were given single cynipoid specimens from European expeditions to different parts of Africa, and thereby became the first to treat Afrotropical taxa of Cynipoidea (Cameron 1904, 1905, Kieffer 1904, 1910a, b, c, d, 1911a, b, 1912, 1913). Single Afrotropical taxa were added by Hedicke (1912), Bridwell (1919) and Kinsey (1919). Later, Louis Weld in the United States made a major effort to summarise and recheck all cynipoid knowledge and revise all classifications based on type studies. His thoroughness made his compendium of cynipoidology (Weld 1952, with a major appendix: Weld 1962) an important groundwork, which was also applicable for the Afrotropical fauna. Further, single taxa were added by Weld (1944), Risbec (1956), Masner (1960), Belizin (1973) and Dessart (1976).

Two systematists authored major faunistic efforts, describing a large number of Afrotropical Cynipoidea in a comprehensive format. The first was carried out by P L G Benoit (1955, 1956a, b, c, d, e), investigating the fauna of Congo and Rwanda. The second was John Quinlan, whose work attempted to document the entire Afrotropical region (Quinlan 1979, 1984, 1986, 1988). Quinlan’s papers followed Nordlander’s (1982 and papers therein), providing the first treatment of Afrotropical cynipoids that employed phylogenetic considerations (Quinlan 1986, 1988). More recently there have been only smaller taxonomic group revisions: Ronquist (1995) on Liopteridae (worldwide); Allemand et al. (2002) on Afrotropical *Leptopilina*; Ros-Farré and Pujade-Villar (2006) on world *Prosaspicera*; Liu et al. (2007) on *Paramblynotus*; Buffington and van Noort (2007) on Pycnostigminae; Buffington and van Noort (2009) on *Anacharoides*; Nielsen and Buffington (2011) on *Stentorceps*; Paretas-Martinez et al. (2009) and Pujade-Villar and Ferrer-Suay (2012) on Afrotropical *Dilyta*; Ferrer-Suay et al. (2012, 2013) on Afrotropical *Alloxysta*; or single taxa added Buffington (2010, 2012), Jimenez and Pujade-Villar (2008); Pujade-Villar (2012); and Mata-Casanova et al. (2014). Buffington and van Noort (2012) and van Noort and Buffington (2013) treated the Afrotropical Liopteridae to the species level. The Afrotropical Figitinae were reviewed by van Noort et al. (2014).

Higher-level cynipoid phylogenetics traditionally follows Ronquist (1999), whose data suggested the rare, Australian-endemic Austrocynipidae was sister-group to Ibaliidae + Liopteridae + (Figitidae + Cynipidae), or (Ibaliidae + Liopteridae) + (Figitidae + Cynipidae). Liopterid phylogenetics were investigated by Ronquist (1995) based solely on morphology; the rarity of many groups of Liopteridae precludes the use of molecular data, so this study remains the most thorough and up to date treatment. Cynipidae phylogenetics has been intensely studied by Liljeblad et al. (2008), Rokas et al. (2002), Ronquist and Liljeblad (2001), as well as the doctoral dissertation of Nylander (2004); Nieves-Aldrey et al. (2009) recently described a new tribe of cynipids, while Liljeblad et al. (2011) provided an updated key to cynipid tribes. A preliminary analysis of the phylogeny of Figitidae demonstrated that the family was monophyletic (Ronquist 1999). Subsequent analyses, employing more thorough taxon and character sampling, found the family monophyletic (Buffington et al. 2007) and paraphyletic (Buffington et al. 2012), the latter study having recovered Cynipidae as sister-group to the figitid subfamilies Thrasorinae + (Pycnostigminae + Mikeiinae). Further phylogenetic interpretation of cynipoids is beyond the scope of this study, however, it should be pointed out that cynipids are very rare in the Afrotropical region, and the gall-associated figitid subfamilies Thrasorinae, Parnipinae, Mikeiinae and Plectocynipinae have not been recorded to date.

A few species of Cynipoidea are of agricultural importance. The siricid wood-wasp, *Sirex noctilio* Fabricius is detrimental to commercial pine tree plantations in South Africa (Tribe and Cillie 2004, van Noort and Picker 2011), and the ibaliid *Ibalia leucospoides* (Hochenwarth) has been introduced to both Australia and South Africa for biological control (Hurley et al. 2007, van Noort and Picker 2011). The gall wasp *Dryocosmus kuriphilus* (Yasumatsu) negatively impacts commercial chestnut (*Castanea*) production, destroying the chestnut itself through galling, and is spreading rapidly throughout Europe (Brussino et al. 2003), Asia (Yasumatsu 1951, reviewed by Melika 2006) and North America (Payne and Anagnostakis 1993). A few eucoiline Figitidae have been evaluated for biological control of pestiferous Diptera, but only *Banacuniculus utilis* (Beardsley) (formerly *Ganaspidium*) and *Aganaspis daci* (Weld) have been actually utilized commercially (Petcherat and Johnson 1988, Wharton et al. 1998).

The world cynipoid fauna has not been catalogued since Dalla Torre and Kieffer (1910), but in recent years efforts have been made to provide overviews of regional faunas, starting with the European cynipoid catalogue within the Fauna Europaea database project (Ronquist and Forshage 2004). A North American catalog of Eucoilinae (Forshage et al. 2013) and an Australian catalog of the entire superfamily have been recently published (Paretas-Martínez et al. 2013). The Australian region comprises 37 recorded genera: one each for Austrocynipidae, Ibaliidae and Liopteridae; two for Cynipidae; and 32 for Figitidae (Paretas-Martínez et al. 2013). While ibaliids are species-poor (with 20 species) and largely confined to the Holarctic, the Liopteridae are relatively rare in all biogeographic regions with the exception of the Oriental region, which supports an unusually high diversity of *Paramblynotus* species (Ronquist 1995a, Liu et al. 2007). The Holarctic region supports a rich gall-wasp fauna (Askew et al. 2013, Nieves-Aldrey 2001, Ronquist and Forshage 2004, Melika 2006), but Eastern (Abe et al. 2007) and Southeast Asia (Tang et al. 2009, 2011a, 2011b, Melika et al. 2011a, 2011b) are just beginning to be explored. The Neotropical cynipids are relatively understudied as well, and recent descriptions suggest our knowledge of this region is in its infancy (Medianero et al. 2011, Nieves-Aldrey et al. 2009, Medianero and Nieves-Aldrey 2010, 2011, Nieves-Aldrey and Medianero 2010, 2011, Melika et al. 2009, 2010, 2012, Melika et al. 2011). Cynipids are very rare in the Afrotropical region, with the recent descriptions of Aylacini (Melika and Prinsloo 2007), Synergini (van Noort et al. 2006) and Qwaqwaini (Liljeblad et al. 2011) providing a glimpse into a hitherto unknown fauna.

Figitidae are likely the most diverse cynipoid group, though the majority of species remain to be described (Nordlander 1984, Ronquist 1999, Buffington et al. 2007, Buffington, Forshage, and van Noort pers. obsv.). Within Figitidae, the Eucoilinae dominate at both the generic and species level of diversity; tropical areas tend to be more species rich, though Holarctic regions can be very rich, including tall-grass prairie and high-elevation deserts in North America (Buffington pers. obsv.). Eucoiline species diversity of Australia is relatively depauperate compared to the rest of the world (Paretas-Martinez et al. 2012). Afrotropical species and generic diversity of Cynipoidea is not as high as that of the Neotropics (Buffington and Forshage pers. obsv.), but there is one endemic subfamily (Oberthuerellinae, with three genera), a few endemic (*Angustacorpa*, *Pycnostigmus*, *Stentorceps*, and *Nanocthulhu*) or almost endemic (*Anacharoides*, *Tylosema*) genera, as well as species-rich genera (*Leptopilina*, *Rhoptromeris*, *Afrostilba*). Furthermore, much of this species and generic richness is widespread in the Old World Tropics, with numerous species and genera found in Southeast Asia, the Afrotropical region and the Oceanic region (Lin 1988, Buffington and Forshage pers. obsv.).

This treatment is part of the initiative to document Afrotropical hymenopteran richness published as a series in ZooKeys (a peer-reviewed, open-access, rapidly produced journal launched to support free exchange of ideas and information in systematic zoology) (http://www.waspweb.org/Afrotropical_Hymenoptera_book/index.htm). The virtual book, including well-illustrated dichotomous identification keys in each chapter will be published as a series of stand alone peer-reviewed scientific papers with all chapters linked together as a virtual book on the ZooKeys website. The publication will include links to online interactive Lucid dichotomous and matrix based keys hosted on WaspWeb. The goal of this paper is to provide a current synthesis of Afrotropical cynipoid systematics, including an overview of biological associations, and the first key to cynipoid genera of the Afrotropical region. The development of this resource is aimed to facilitate future research on this ecologically and agriculturally important superfamily of wasps. To this end, we provide keys to all of the genera of Afrotropical Cynipoidea. As part of this overview assessment we present seven new genus-level synonymies, one genus resurrected from synonymy, 54 new combinations, one combination reinstated, and one new replacement name.

## Materials and methods

Character states diagnostic of each taxon were discerned from material in extensive recent collections of African Hymenoptera housed at the Iziko South African Museum, Cape Town; California Academy of Arts and Science, San Francisco; National Museum of Natural History (Smithsonian Institution), Washington DC, and Natural History Museum, London. Historically there are important Hymenoptera collections from the region that are housed in a number of European Museums including the Natural History Museums in Paris, Tervuren, and Berlin, to name a few. We have also made use of material housed in the Museum of Zoology of Lund University, Sweden, the Swedish Museum of Natural History, Stockholm; South African National Collection, Pretoria; Biologiezentrum, Linz; and elsewhere. The wealth of recently sampled Hymenoptera residing in African, European and USA museums has been built up over the last 20 years by extensive and rigorous quantified and replicated inventory surveys using a wide diversity of collecting methods (Malaise traps, yellow pan traps, sweeping, pitfall traps, Winkler bag extraction of leaf litter, UV light trapping and tree canopy fogging) carried out across large parts of Africa and Madagascar by Simon van Noort (Iziko South Afrian Museum); Brian Fisher and colleagues (Californa Academy of Sciences); Bob Copeland (affiliated with National Museums of Kenya and the National Museum of Natural History); Michael Sharkey (University of Kentucky); and John Noyes (Natural History Museum London). The Hymenoptera from these samples provide an unparalleled resource from which the systematics and diversity of Afrotropical Hymenoptera can continue to be elucidated. All the collections where we have been studying Afrotropical Cynipoidea are listed below.

Freshly collected specimens were point-mounted on black or white, acid-free cards for examination (using a Leica MZ9.5, Z16 or M205c stereomicroscope with incandescent and fluorescent light sources), photography and long-term preservation. Representative specimens were imaged using the EntoVision multiple-focus imaging system to illustrate diagnostic characters. Methods for generating these photographs follow those in Buffington and van Noort (2009). Diffused lighting was achieved using techniques summarized in Buffington et al. (2005), Kerr et al. (2009) and Buffington and Gates (2009). Scanning electron micrographs were generated using a Hitachi TM3000 desktop scanning electron microscope; specimens were coated in 25–30 nm gold-palladium alloy, or imaged uncoated, using ‘analysis’ voltage, running in ‘compo’ mode. All new images generated for this project are deposited in Morphbank.

Morphological terminology follows that of Fontal-Cazalla et al. (2002) and Ronquist and Nordlander (1989); cuticular surface terminology follows Harris (1979). Character matrices were generated and edited using Microsoft Excel; matrices were then used as input into Lucid matrix key production (Penev et al. 2009) Online interactive keys were produced using Lucid and Lucid Phoenix meeting the requirements of publishing both static and dynamic interactive keys under an open access model (Penev et al. 2009). All keys were illustrated using high quality annotated images, highlighting diagnostic characters. The images are integrated into the key above each couplet resulting in a user-friendly output. This key format circumvents the requirement of familiarity with morphological terminology associated with a particular taxonomic group, because the characters are visually illustrated making the keys usable by a wide range of end-users including the lay person. These keys are available at: http://www.waspweb.org/Cynipoidea/Keys/index.htm. End users can choose between three different key formats depending on their personal preference. The keys are available in three formats. Although Lucid Phoenix keys are interactive keys they are still dichotomous and a choice needs to be made at each key couplet to continue. Lucid matrix keys, on the other hand, use a different approach where relevant states from multiple character features can be selected independently until identification is achieved. For more information concerning Lucid keys visit http://www.lucidcentral.org. This publication is available in 4 different formats: (1) high-resolution, full-colour print version, to satisfy the current requirements of the International Code of Zoological Nomenclature (ICZN), as well as for readers who prefer hardcopy, and for the purposes of paper archiving; (2) PDF to provide an electronic version identical to the printed one, to be archived in BHL and PubMedCentral; (3) HTML to provide links to external resources and semantic enhancements to published texts for interactive reading, and (4) XML version based on the TaxPub XML schema to provide archiving document format for PubMedCentral and a machine-readable copy of the contents to facilitate future data mining (Penev et al. 2010b).

**Figure 1.**
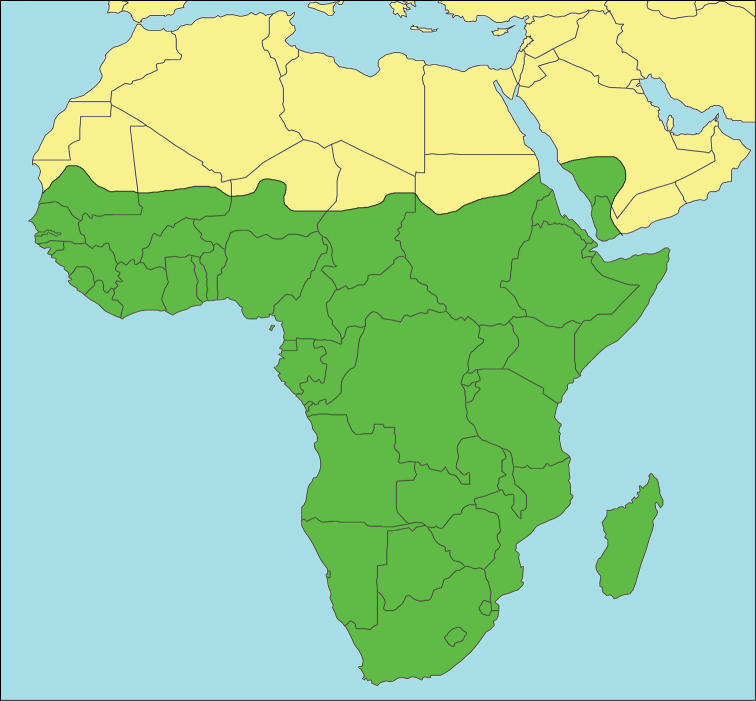
The Afrotropical region is depicted in green. The northern limits correspond to the boundary between the arid and hyper-arid climatological zones, delimited by the 150 mm precipitation isohyet.

The Afrotropical region is a relatively uncontroversial concept (Fig. 1) conforming to the old Ethiopian region of Sclater and Wallace from the earliest days of zoogeography, with the name changed as of Crosskey and White (1977). As in all the authoritative versions of delimitations, Madagascar and the islands of the western Indian Ocean are included, as is the southern part of the Arabian Peninsula, the South Atlantic islands of Ascension, St. Helena, Tristan da Cunha and Gough, as well as the Cape Verde Islands and the Gulf of Guinea islands (Darlington 1957, Crosskey 1980). Crosskey and White (1977) defined the northern limit of the region by the 10 inch (254 mm) precipitation isohyet. With climate change and aridification this boundary is constantly in a state of flux. Here we use the boundary between the arid and hyper-arid climatological zones as defined by the World Meterological Organization and United Nations Environment Program derived from mean monthly precipitation and potential evapotranspiration surfaces (Desanker and Magadza 2001). This corresponds to the northern edge of the Sahel region bordering with the southern limits of the Sahara desert, a boundary which is usually demarcated by the 150 mm isohyet (White 1983, Fensholt et al. 2013). White (1983) and Linder (2012) further subdivide the sub-Saharan region into cohesive biogeographical subentities based on plant and vertebrate data. Patterns of invertebrate distributions would be expected to correlate with the environmental partitioning of the region.

We have not included major systematic revisionary work in the preparation of this paper, but have implemented taxonomic nomenclatural acts, as required, to bring the systematic treatment of the Cynipoidea in line with a contemporary assessment of the superfamily. The main rationale behind this initiative is to produce workable, accessible keys to generic level based on current taxonomic knowledge, a contemporary state-of-the-art resource that will be available to facilitate future systematic revisions. With continued ongoing sampling new taxon discoveries are being made all the time and this treatment will be out of date as soon as it is published. However, the online taxon treatment and identification keys available on WaspWeb will be expanded and updated as taxonomic progress is made. This is one of the major advantages of electronic output.

All images presented in this paper are available through http://morphbank.net and http://www.waspweb.org using the link to individual collections.

Synonyms (for species-level taxa as well as higher taxa) are cited only to the extent they are relevant for the discussion of the knowledge of the Afrotropical fauna. For each genus, we summarise the knowledge of its biology and its worldwide distribution, as well as provide a list of Afrotropical country records with references. In most of the figitids, our knowledge is not yet at the level where the diversity has been systematically treated on the species level. As a result, only a minority of the specimens (and thus of the country records) are assignable to a species name; for these, it is the genus-level treatment which is the basic unit and which contains the most information. Since this is a checklist rather than an actual catalogue, we do not give full references to original descriptions, cite type repositories, full synonymies and nomenclatural histories (except in the cases involving nomenclatural acts made here); we provide standard authorship designation and list the genus if different to the original combination in a parenthesis. In the most extensive treatment of Afrotropical taxa before this, Quinlan (1979, 1986, 1988) was sometimes inconsistent in terms of gender agreement in the scientific names, and a certain number of gender endings are changed accordingly. Further, certain genera have been inconsistently treated in terms of gender throughout scientific history, and we strive to add consistency here.

This project is the result of more than 40 years’ worth of cumulative research by all three authors. SvN sampled extensive areas across Africa from 1992 to present for Cynipoidea, supplemented by MB and other initiatives listed above. Management of curation of the extensive recent collections of material in Iziko SAMC was conducted by SvN, of those in USNM by MB. MF spent the last 10 years reviewing generic and species concepts and generating generic keys for Eucoilinae; MB has spent the last 10 years similarly studying the entire Figitidae, but especially the genera of Diglyphosematini. MB and SvN have been publishing on endemic African groups of Cynipoidea for some time, and SvN about the region’s Cynipidae. Responsibilities for taxonomic assessment (specimen examination and identification of material in said museums as well as numerous international museums, including assessment of generic concepts and delimitation, dichotomous and matrix key formulation and catalogue production) were generally divided as follows: Liopteridae and Figitidae: Pycnostigminae (MB and SvN); Figitidae: Anacharitinae, Charipinae, Emargininae, Eucoilinae (MF and MB); Cynipidae; Figitidae: Aspicerinae, Figitinae (MB, MF and SvN). Imaging of type material and representative taxa in various international institutions was carried out by SvN and MB. New SEM images were done by MB (others downloaded from MorphBank). Lucid key production and image plate production for the keys was done by SvN. Keys to genera of figitids was originally developed by MF; keys to liopterids by SvN and MB. The format and production of this project was conceptualised by SvN in consultation with MB and MF as part of the Afrotopical Hymenoptera Initiative virtual book project (http://www.waspweb.org/Afrotropical_Hymenoptera_book/index.htm). All authors collaborated on the final writing and editing of the paper.

### List of depositories

We have considered it relevant to list in some detail the collections referred to in this paper, especially in terms of holdings of Afrotropical Cynipoidea.

BMNH Natural History Museum, London, UK. Curator David Notton. Large amounts of material, mostly identified by John Quinlan and MF, a lot from Cameroon, Democratic Republic of Congo, Kenya, Madagascar, Mauritius, Nigeria, South Africa, Uganda, Zambia but also dozens of other countries, and mostly older material. Several Cynipoidea type specimens from Quinlan, and recently described types by SvN, MB & MF from historical material.

CASC California Academy of Sciences, San Francisco, USA. Curator Bob Zuparko. Vast recently collected material from Madagascar; the mounted parts thereof currently housed in USNM.

CNCI Canadian National Collection of Insects, Ottawa, Canada. Curator Andrew Bennett. Large amount of material, mostly wet. Especially Botswana, Burkina Faso, Kenya, Madagascar, Rwanda, South Africa, Uganda, but also various other countries.

CUMZ Cambridge University Museum of Zoology, Cambridge, UK. Curator William Foster. Has Kieffer paratypes from the Seychelles.

DEI Senckenberg Deutsches Entomologisches Institut, Müncheberg, Germany. Curator Andreas Taeger. Rather large material in alcohol (coll Tschirnhaus) from Ethiopia, Ivory Coast, Madagascar and elsewhere in Africa.

HNHM Magyar Természettudományi Múzeum, Budapest, Hungary. Curator Zoltán Vas. Small amounts of recently collected Afrotropical material.

MNHN Natural History Museum, Paris, France. Curator Claire Villemant. Several Kieffer types, and recently described types by SvN and MB from historical Seyrig Madagascan collections, but no recently collected material.

MZLU Zoologiska Museet Lunds Universitet, Lund, Sweden. Curator Christer Hansson. Especially South Africa, Gambia, Senegal, Cameroon and Sierra Leone, but also other countries, especially in the important Sporrong collection, including recently collected material, identified by Michael Sporrong and MF.

NHRS Naturhistoriska Riksmuseet, Stockholm, Sweden. Curator Hege Vårdal. Recent collecting mostly from Madagascar, plus Cynipoid fractions of some other recent collecting efforts, identified by MF.

NMKE National Museums of Kenya, Nairobi. Curator Martha Gikunga. Recently collected material from East Africa, including a couple of recently described cynipoid types by SvN and MB.

OLML Biologiezentrum, Oberösterreichische Landesmuseen, Linz, Austria. Curator Fritz Gusenleitner. Some recently collected material from Kenya, Zambia and elsewhere, identified by MF.

RMCA Musée Royal de l’Afrique Centrale, Tervuren, Belgium. Curator Eliane de Coninck. Several Quinlan, Benoit types and some recently described types by SvN and MB from historical DRC material, but no recent material.

SAMC Iziko South African Museum, Cape Town, South Africa. Curator Simon van Noort. Vast recently collected materials from Central African Republic, Gabon, South Africa, Tanzania, Uganda, as well as Yemen and many other countries; identified by SvN, MB and MF.

SANC South African National Collection of Insects, Pretoria, South Africa. Curator Ros Urban. Some South African material.

SLU Sveriges Lantbruksuniversitet, Ultuna, Sweden. Houses the Göran Nordlander collection with a number of types.

TARI Taiwan Agricultural Research Institute, Taichung, Taiwan. No important Afrotropical material to our knowledge.

USNM National Museum of Natural History, Washington DC, USA. Curator Matthew Buffington. Vast recently collected materials mainly from Madagascar (from CASC) and Kenya, but also dozens of other countries, and including older material.

ZMBH Museum für Naturkunde, Humboldt-Universität, Berlin, Germany. Curator Frank Koch. Several Kieffer types, but no recent material to our knowledge.

ZMUH Zoologisches Institut und zoologisches Museum, Universität von Hamburg, Germany. Curator: Kai Schütte. Historical material destroyed, currently no important Afrotropical material to our knowledge.

## Key to families of Afrotropical Cynipoidea

**Table:** 

	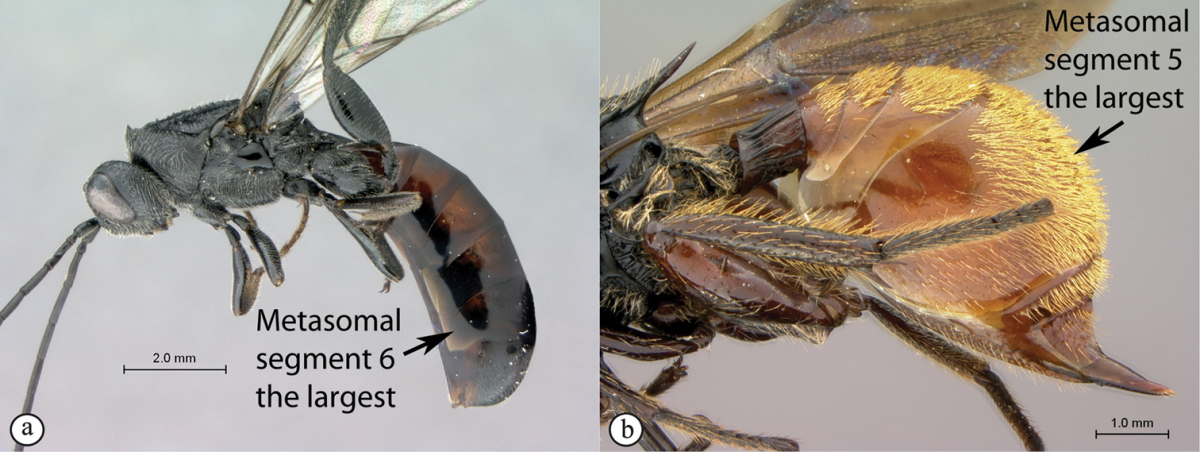	
1	Metasomal segment four, five or six the largest, when viewed laterally, with two to four small segments preceding largest segment (a, b). Rarely collected	**2**
	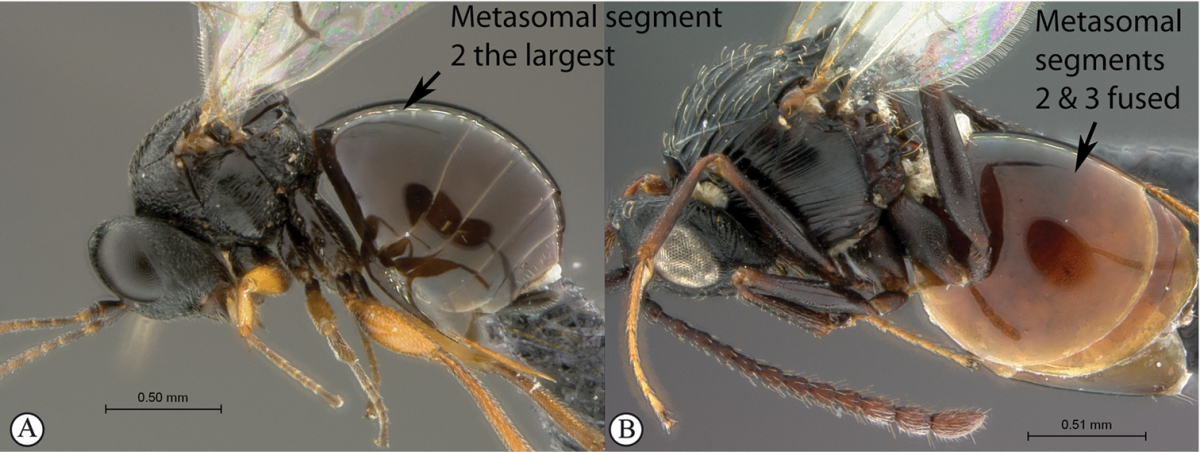	
–	Metasomal segment two or three the largest, when viewed laterally (A), or fused into a syntergum (B), with at most only one small segment preceding the largest. Commonly collected	**3**
	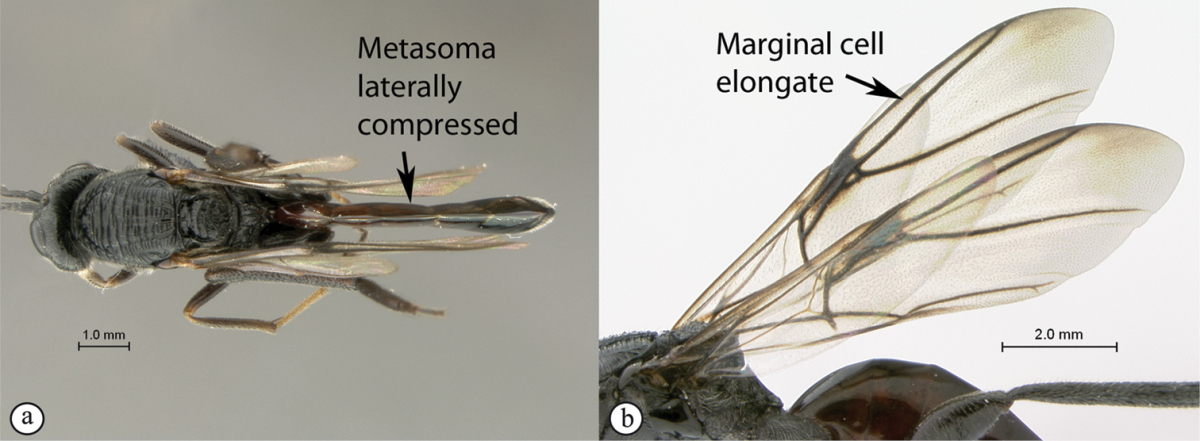	
2	Metasoma, when viewed dorsally, extremely thin, blade-like (a); marginal cell of forewing at least nine times as long as broad, closed on front margin (b). Large wasps, exceeding 10 mm in body length (extra-limital family – one introduced species, *Ibalia leucospoides* in South Africa)	**Ibaliidae**
	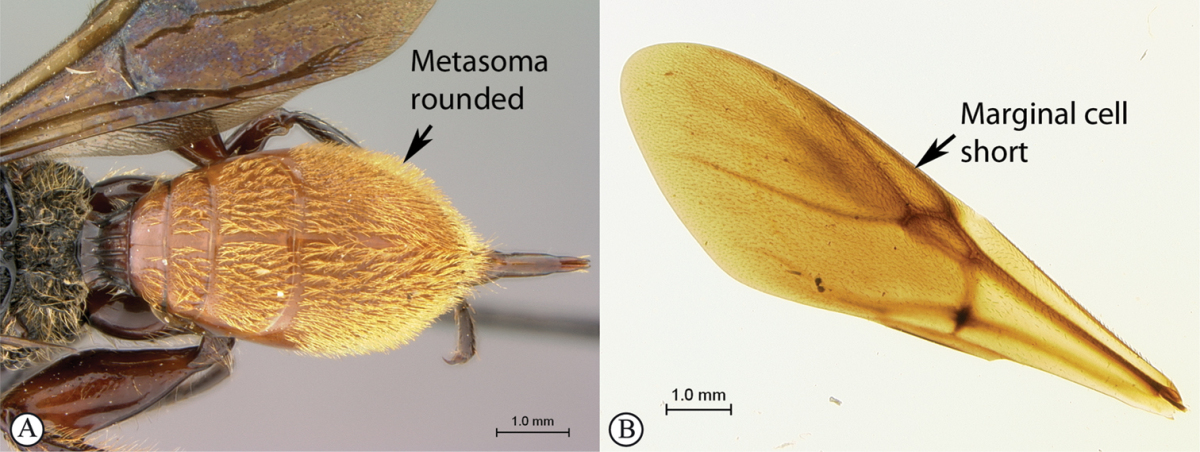	
–	Metasoma, when viewed dorsally, evenly rounded on both sides, often rather stout (A); marginal cell of forewing at most six times as long as broad (B). Small to large wasps, ranging from 2 to 10 mm in body length	**Liopteridae**
	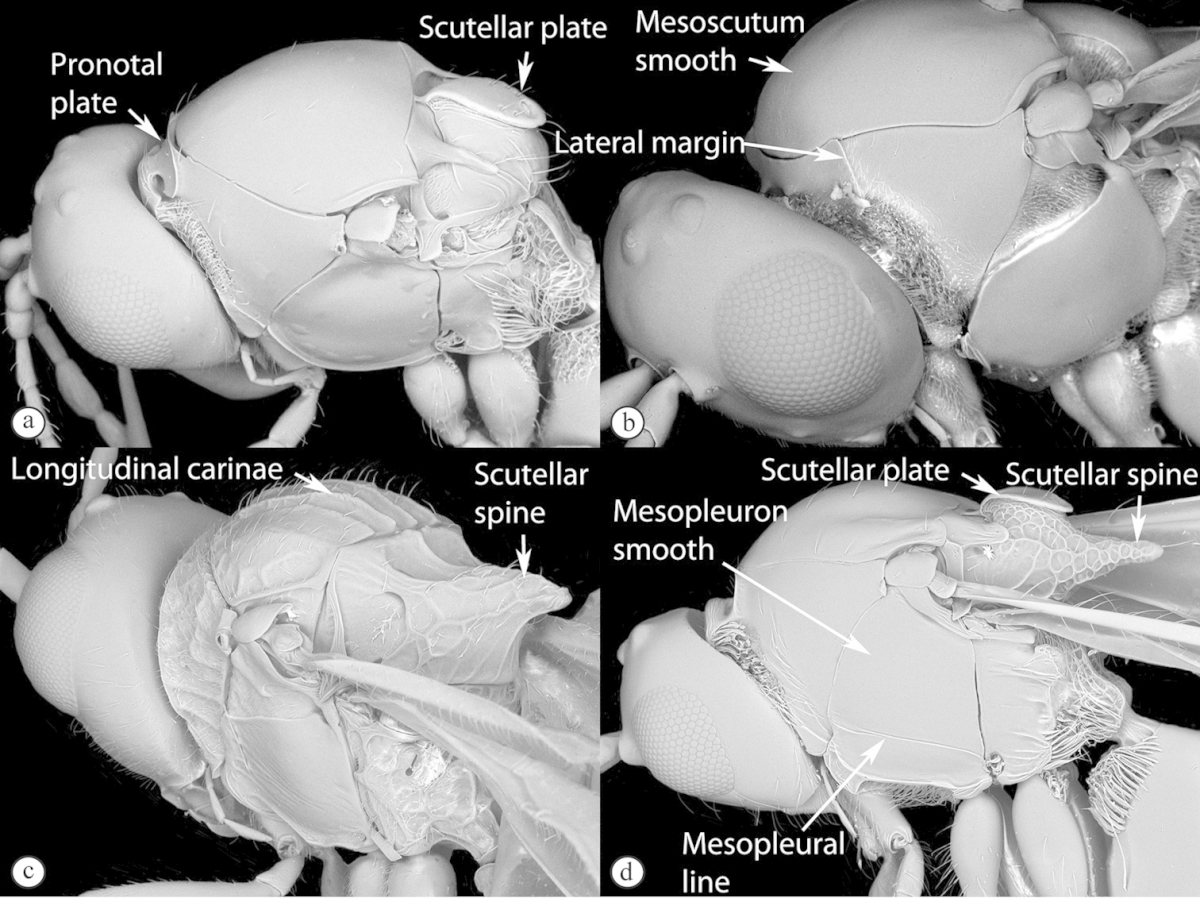	
3	Pronotal plate present, often projecting forward, with distinct ventro-lateral margins (a, b). Mesoscutum usually smooth (at least dorsally) (a, b), occasionally microcoriaceous; may be longitudinally carinate (c). Mesopleuron usually smooth (d), often with a single longitudinal line (d). Scutellum frequently with distinct central plate (a, d), and/or posteriorly directed spine (c, d)	**Figitidae**
	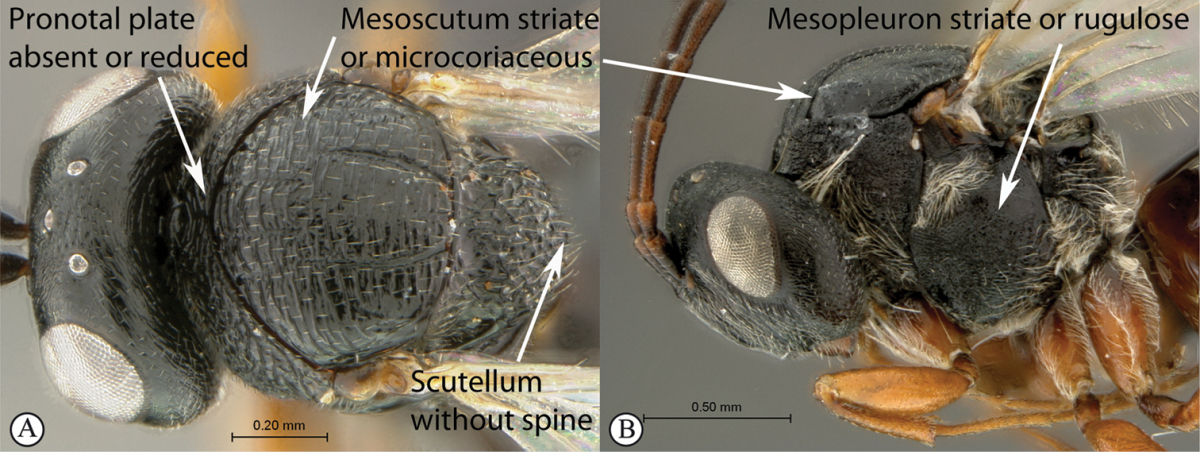	
–	Pronotal plate absent or reduced, ventro-lateral margins not visible (A). Mesoscutum horizontally striate (A) or microcoriaceous (B). Mesopleuron usually entirely striate or gently rugulose, lacking a distinct longitudinal line (B). Scutellum never with a central plate or an apical spine (A)	**Cynipidae**

## Cynipidae

The Cynipidae are represented in the Afrotropical region by three of the world’s eight tribes: Aylacini, Synergini and Qwaqwaiini, and four described species. *Phanacis* contains a number of undescribed species indicating that the genus is likely to be far richer in southern Africa than currently recorded.

**Biology.** Afrotropical cynipids are biologically better known than other African cynipoids and include an endemic, specialist lethal inquiline (van Noort et al. 2007) and gall formers of both herbs and trees (Liljeblad et al. 2011, Melika and Prinsloo 2007).

**Distribution.** The family is represented in all biogeographical regions with the majority of species occurring in the northern hemisphere (Liljeblad et al. 2008, 2011).

### Key to Afrotropical cynipid genera

**Table:** 

	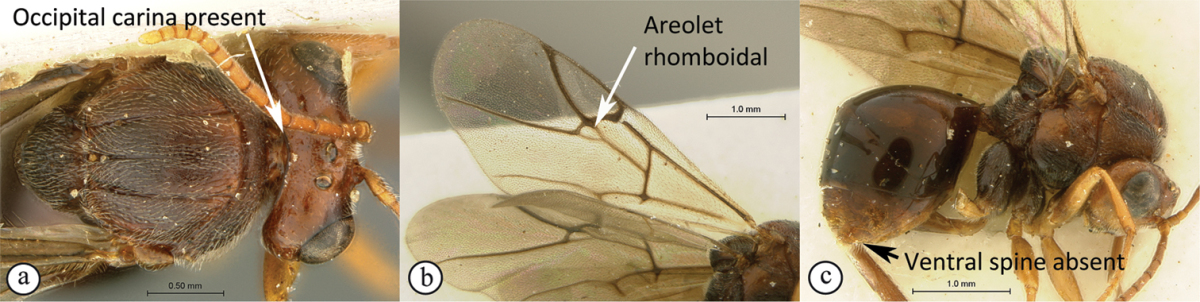	
1	Occipital carina present (a); forewing areolet rhomboidal, elongate (b); hypopygium abrupt, ventral spine absent (c), with a dense tuft of long setae; gall-inducers on *Scolopia*	***Qwaqwaia* (Qwaqwaiini)**
	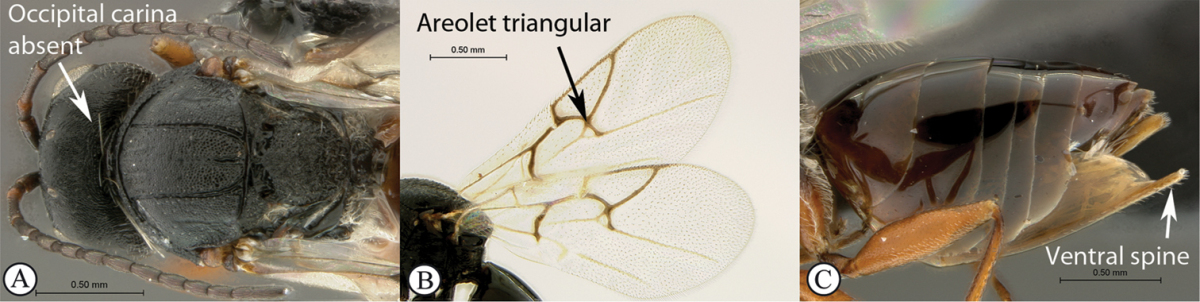	
–	Occipital carina absent (A); forewing areolet triangular (B); hypopygium with a distinct, elongate ventral spine (C), subapical setae never forming a dense tuft	**2**
	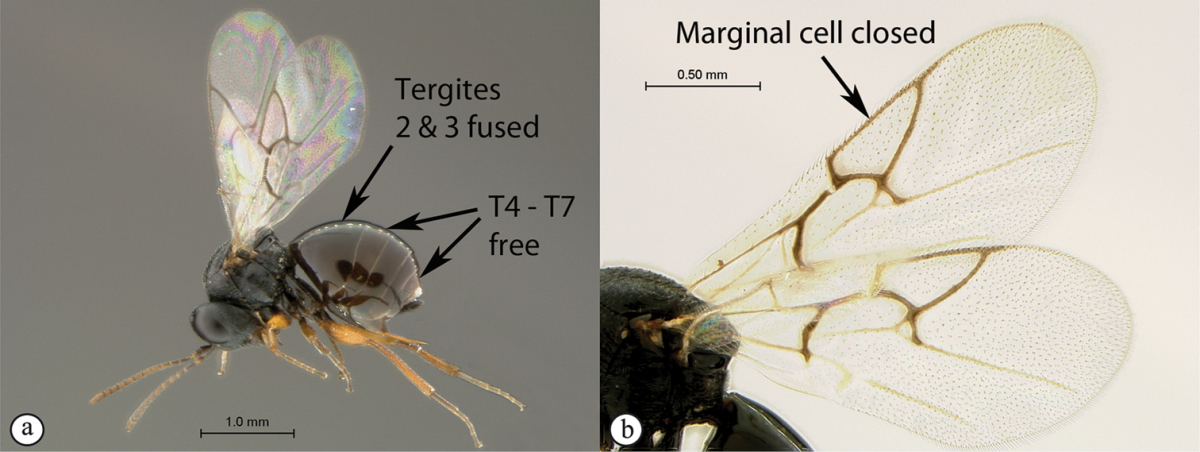	
2	Metasomal tergites 2+3 fused into a single segment (a); marginal cell closed, areolet present (b); inquiline in galls on *Searsia*	***Rhoophilus* (Synergini)**
	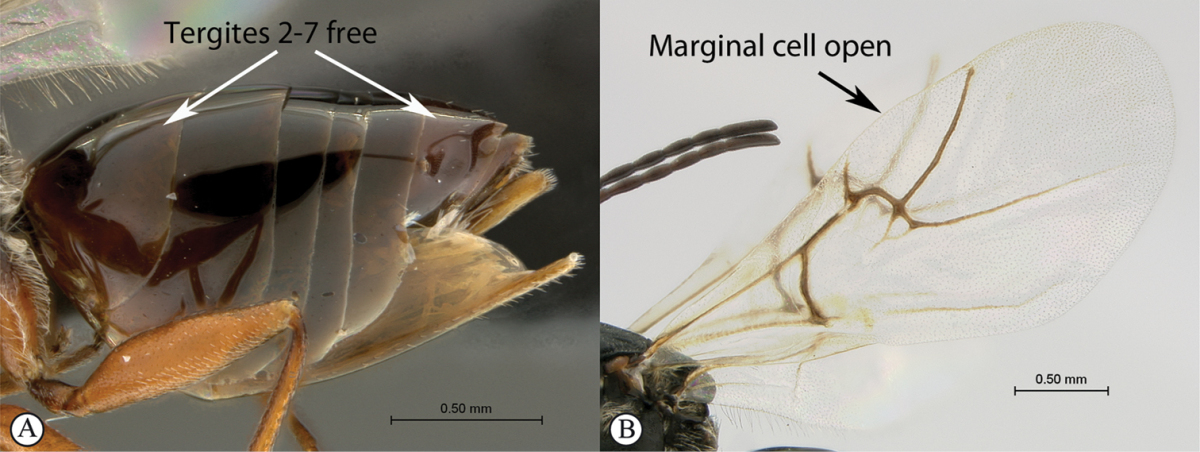	
–	Tergites 2–7 free (A); marginal cell open, may be semi-closed (B); areolet present or absent (B); gall-inducers on herbaceous plants	***Phanacis* (Aylacini)**

### Aylacini

#### Phanacis

Taxon classificationAnimaliaHymenopteraCynipidae

Förster, 1860

##### Remarks.

*Phanacis* Förster is closely related to *Timaspis* Mayr, which is considered to be a junior synonym by some authors (Eady and Quinlan 1963, Melika 2006, Melika and Prinsloo 2007), but not others (Nieves-Aldrey 1994, 2001).

##### Diagnosis.

*Phanacis* is immediately distinguishable from the other cynipid genera present in the Afrotropical region by the open marginal cell, which may be semi-closed (completely closed in both *Qwaqwaia* and *Rhoophilus*). The areolet, if defined, is triangular, but may be inconspicuous or absent in some species. It is large and distinct in both other genera, triangular in *Rhoophilus*, elongate rhomboidal in *Qwaqwaia*. *Phanacis* lacks an occipital carina as in *Rhoophilus*, but has free metasomal tergites, whereas *Rhoophilus* has tergites 2 and 3 fused.

**Figure 2.**
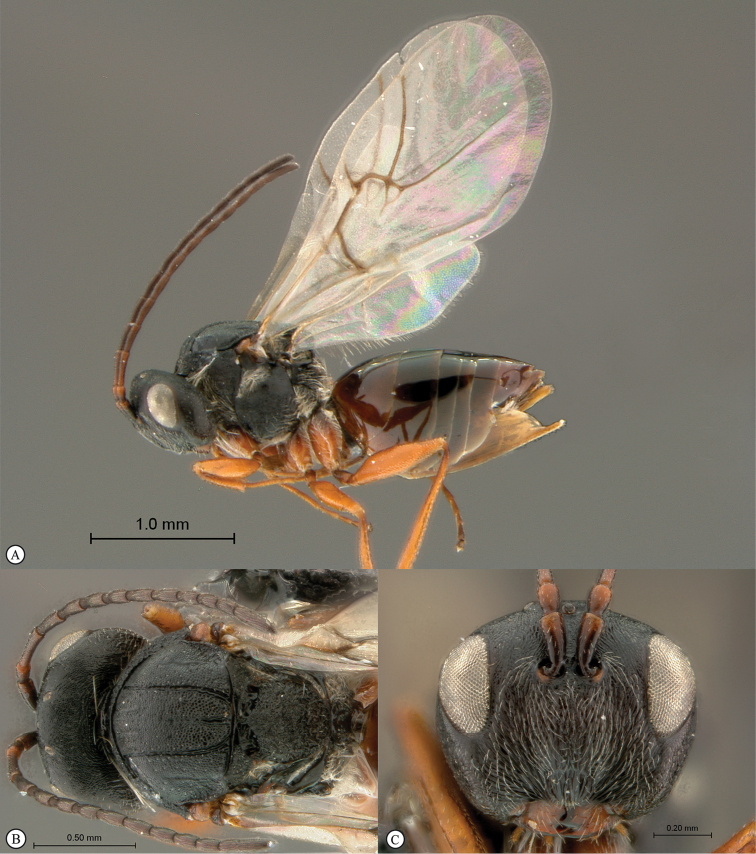
*Phanacis* species (South Africa). **A** habitus lateral view **B** head and mesosoma dorsal view **C** head anterior view.

##### Distribution.

Afrotropical: South Africa: Western Cape Province and Eastern Cape Province. Also Palearctic including North Africa; Nearctic: USA and Canada (introduced); Neotropical: Argentina (introduced); Australasia: Australia (introduced).

##### Biology.

Phytophagous: *Phanacis neserorum* oviposit in young, soft stems of *Chrysanthemoides monolifera* (L.) Norl. (Asteraceae), larvae developing in cells in the pith without any visible gall formation. Adults emerge in July and August. Gall formation shows no swelling or external deformation of the stem with surface emergence holes the only indication of infestation (Melika and Prinsloo 2007).

The introduced *Phanacis hypochoeridis* is a gall former in stalks of *Hypochaeris radicata* L. (Asteraceae) (Melika and Prinsloo 2007).

**Species richness.**

*Phanacis hypochoeridis* (Kieffer, 1887) (*Aulax*) (introduced to South Africa from Europe)

*Phanacis neserorum* Melika & Prinsloo, 2007 (South Africa)

A number of undescribed species are known from South Africa.

### Synergini

#### Rhoophilus

Taxon classificationAnimaliaHymenopteraCynipidae

Mayr, 1881

##### Remarks.

*Rhoophilus* is morphologically similar to the Holarctic inquiline genera *Synergus*, *Saphonecrus*, and *Synophrus*, all of which typically attack oak cynipid galls. The transverse ridges of the mesoscutum, and a mesopleuron sculptured with longitudinal ridges in *Rhoophilus* closely resemble characters in several species of the *Synergus*/*Saphonecrus* complex. A sister group relationship between *Rhoophilus* and these three oak inquiline genera was hypothesized by Ronquist (1994) and Liljeblad and Ronquist (1998).

##### Diagnosis.

*Rhoophilus* has a closed marginal cell, a character shared with *Qwaqwaia*. The areolet is triangular as in *Phanacis*, but larger and more distinct, whereas it is elongate rhomboidal in *Qwaqwaia*. *Rhoophilus* lacks an occipital carina as in *Phanacis* (present in *Qwaqwaia*), but has tergites 2 and 3 fused, whereas these are free in *Phanacis*.

**Figure 3.**
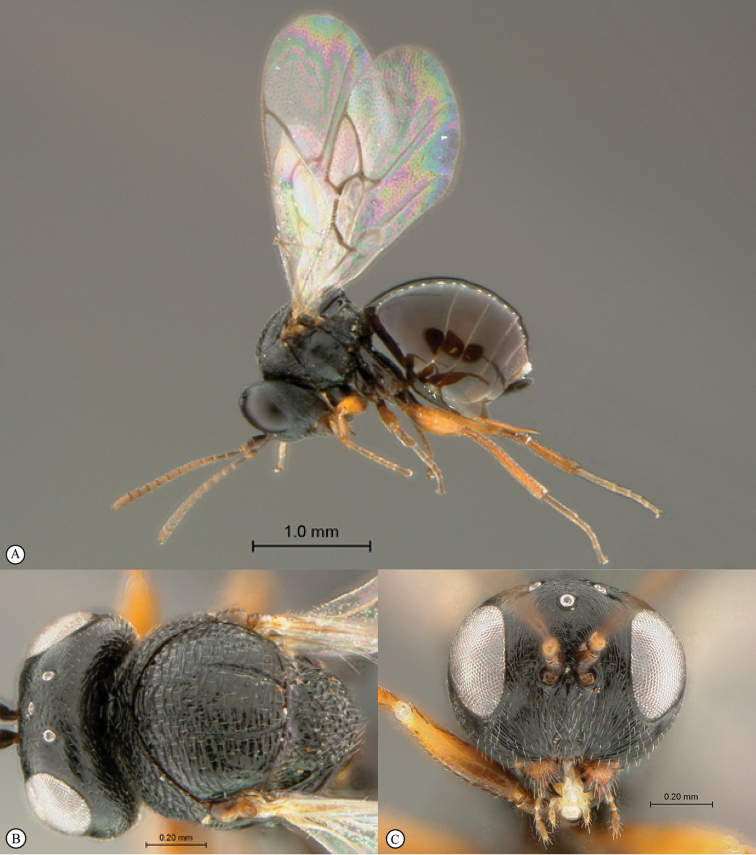
*Rhoophilus loewi* (South Africa). **A** habitus lateral view **B** head and mesosoma dorsal view **C** head, anterior view.

##### Distribution.

South Africa: Western, Eastern and Northern Cape Provinces.

##### Biology.

Inquiline forming secondary cells in galls induced by *Scyrotis* moths (Cecidosidae) on *Searsia* (formerly *Rhus*) (Anacardiaceae) shrubs and trees. The larval cells expand into the hollow interior of the host gall resulting in death of the gall inducing moth larva (van Noort et al. 2007).

##### Species richness.

*Rhoophilus loewi* Mayr, 1881 (South Africa)

### Qwaqwaiini

#### Qwaqwaia

Taxon classificationAnimaliaHymenopteraCynipidae

Liljeblad, Nieves-Aldrey & Melika, 2011

##### Remarks.

*Qwaqwaia* has a dorsally wide pronotum, a character shared with members of the Synergini and the Aylacini (especially some species of *Phanacis*). However, the presence of only two prominent teeth on the right mandible and a parascutal carina that extends anteriorly all the way to the notaulus separate *Qwaqwaia scolopiae* from species of these two tribes (Liljeblad et al. 2011).

##### Diagnosis.

*Qwaqwaia* has a strong, sharp occipital carina, which distinguishes this genus from both other Afrotropical representatives of this family where the carina is absent. Marginal cell of forewing closed, with veins heavily pigmented, a character state shared with *Rhoophilus*, however, the areolet is elongate rhomboidal whereas it is triangular in *Rhoophilus*. The hypopygium is short, abrupt, without a projecting ventral spine (present in both other genera), with dense setae forming an apical tuft.

**Figure 4.**
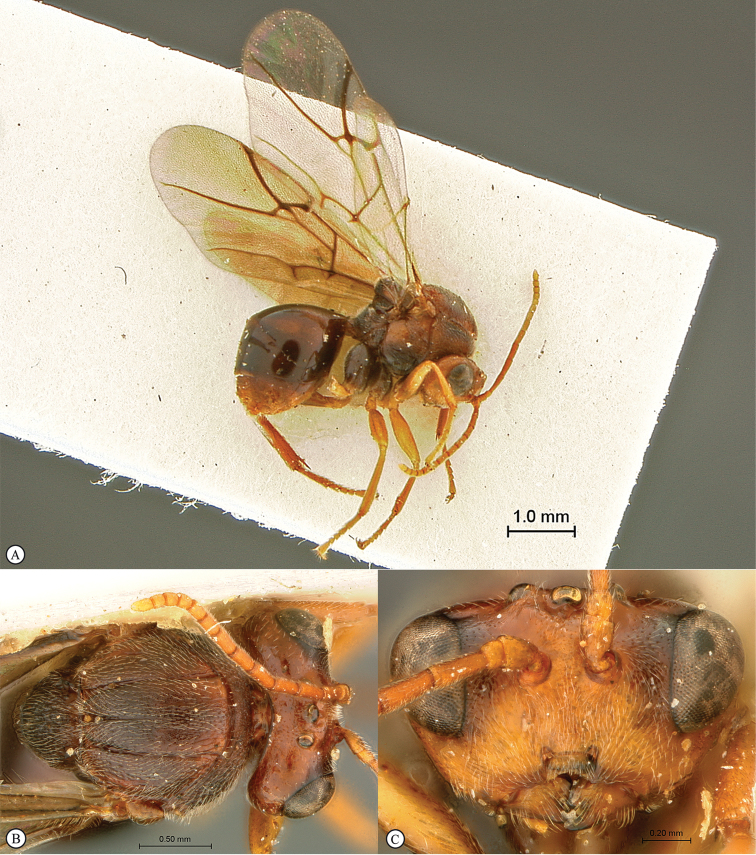
*Qwaqwaia scolopiae* female (Holotype, South Africa). **A** habitus lateral view **B** head and mesosoma dorsal view **C** head, anterior view.

##### Biology.

Gall inducer on *Scolopia mundii* (Eckl. & Zeyh.) Warb. (Salicaceae), a tree in the eudicot subclass Rosidae (Liljeblad et al. 2011).

##### Distribution.

South Africa: Kwazulu-Natal; Mpumalanga.

##### Species richness.

*Qwaqwaia scolopiae* Liljeblad, Nieves-Aldrey & Melika, 2011 (South Africa)

## Figitidae

The Figitidae are represented in the Afrotropical region by seven of the world’s 12 subfamilies and comprise the richest component of the Afrotropical cynipoid fauna with 236 described species. All subfamilies contain numerous undescribed species, dominated by the hyper-diverse Eucoilinae.

**Biology.** Afrotropical figitids are primarily endoparasitoids of Muscomorpha Diptera, attacking the early instar stages of their hosts and emerging from the host puparium (Buffington et al. 2012). The two exceptions to this pattern are the Anacharitinae and Charipinae, of which the former are Chrysopidae parasitoids (Neuroptera), and the latter are hyperparasitoids of braconids and chalcidoids in plant lice (Sternorrhyncha) (Buffington et al. 2012). Hosts are unknown for the Emargininae and Pycnostigminae, as well as numerous species of Eucoilinae. Ecologically, figitids are mainly associated with three environments: aphid communities on plants, where they attack aphid enemies (Charipinae, Anacharitinae, Aspicerinae); that of plant leafminers, attacking the mining flies (many Eucoilinae); and arguably the most common is various decomposing matter, where saprophagous flies are the hosts (many Eucoilinae, Figitinae).

**Distribution.** The family is represented in all biogeographical regions, also on subantarctic islands. The largely undescribed tropical faunas are difficult to compare, but eventually the Neotropical fauna might very well turn out to be the richest of all (Buffington and Forshage pers. obsv.).

### Key to Afrotropical figitid subfamilies

**Table:** 

	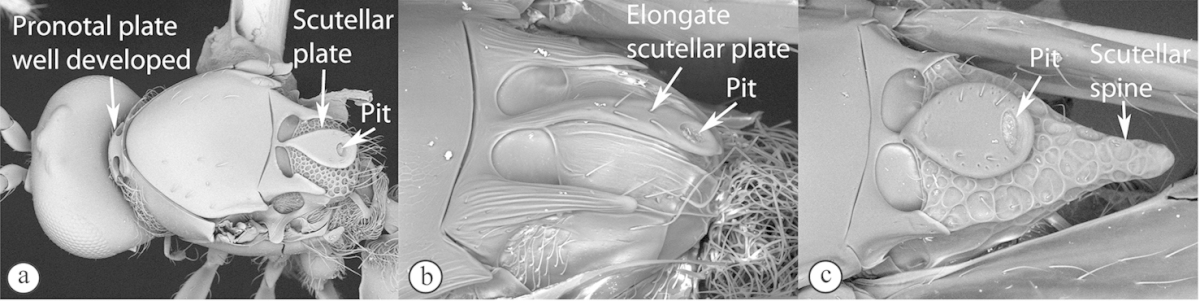	
1	Scutellum with a disc or cup on dorsal surface with a central or posterior pit (a, b, c). Pronotal plate well-developed, frequently produced anterio-dorsally into an anterior plate with a strong postero-lateral margin (a). Segments 2 and 3 of metasoma fused, without visible suture	**Eucoilinae**
	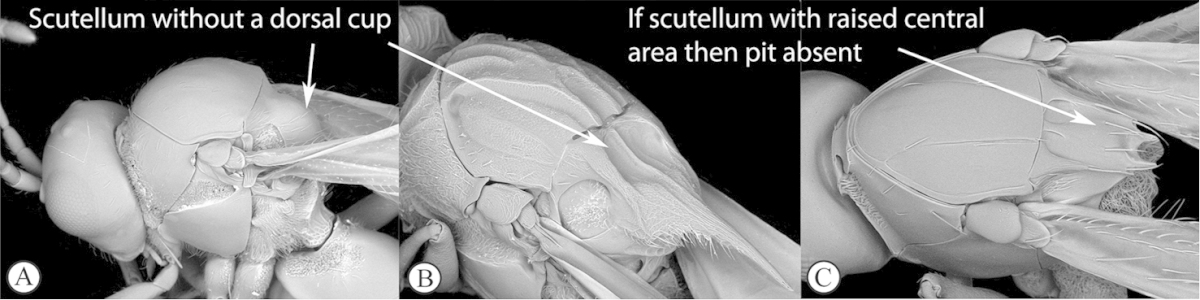	
–	Scutellum without a disc or cup dorsally (A, B); occasionally with raised central area, but lacking a central or posterior pit (C). Pronotal plate frequently lacking a strong posterior margin. Segments two and three rarely fused	**2**
	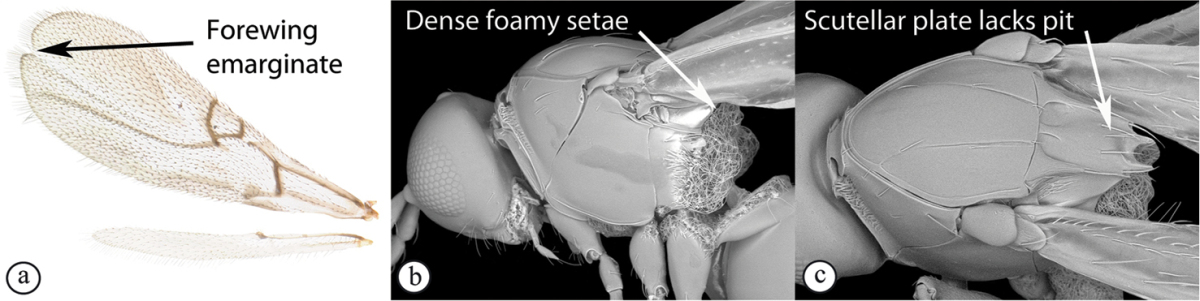	
2	Apical margin of forewing distinctly emarginate (a). Dense ‘foamy’ setae present on propodeum and anterior of metasoma (b). Scutellar plate occasionally present, but lacking central pit (c)	**Emargininae (*Thoreauella* )**
	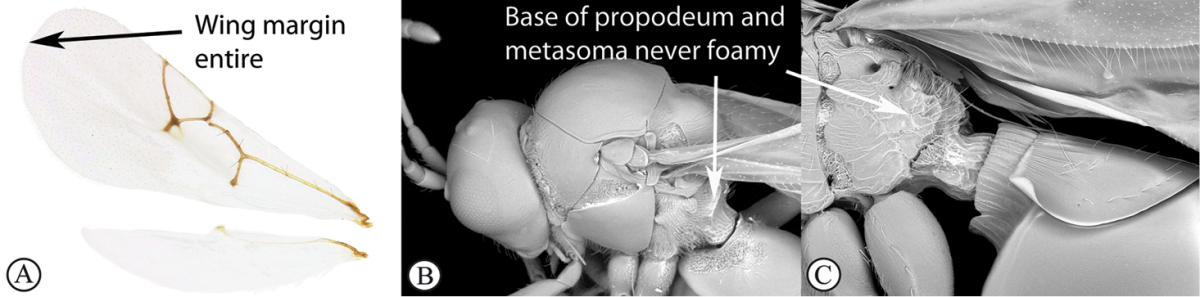	
–	Apical margin of forewing entire (A). Propodeum and base of metasoma setose or glabrous, but never with dense, ‘foamy’ setae (B, C)	**3**
	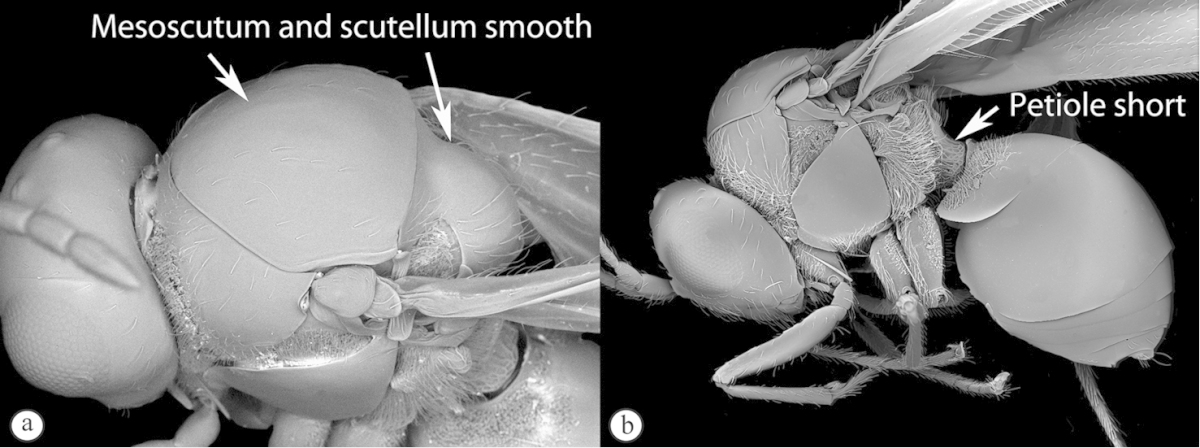	
3	Mesoscutum and scutellum completely smooth and rounded, entirely lacking sculpture (a). Petiole very short, at most as long as broad (b)	**Charipinae**
	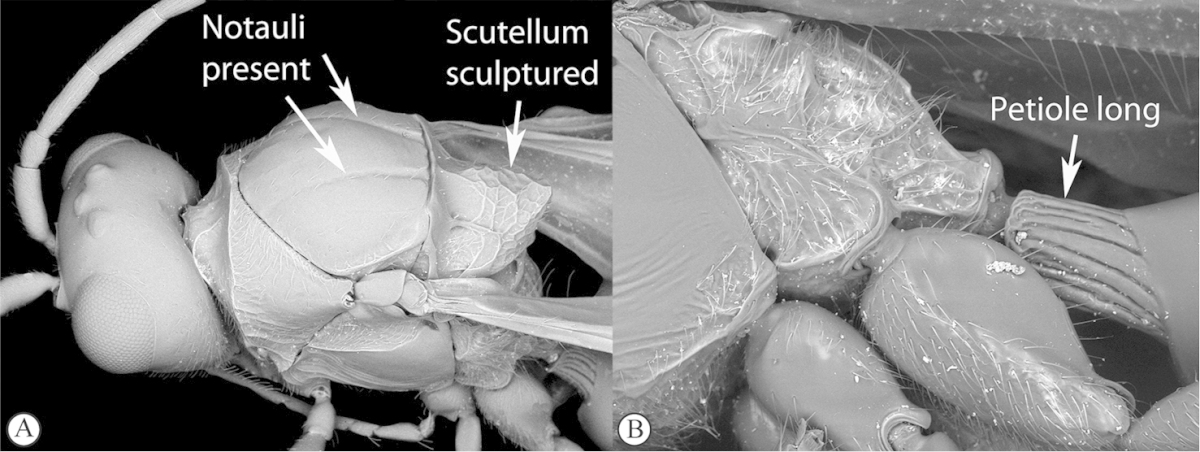	
–	Mesoscutum and scutellum frequently sculptured, most often with notauli present on mesoscutum, and the scutellum crenulate to rugose; if scutellum smooth, then with distinct posterior carina and petiole long, at least 4× longer than wide	**4**
	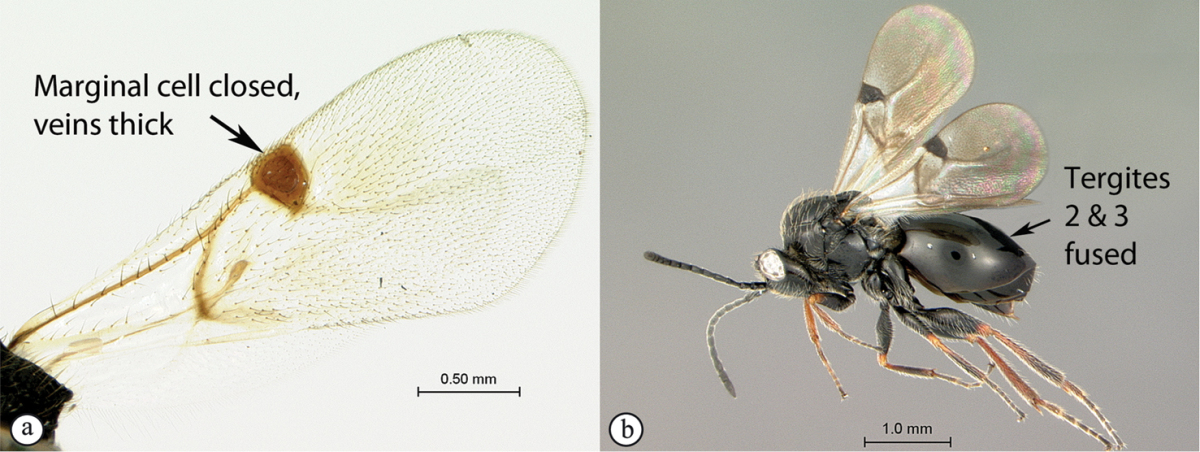	
4	Marginal cell of forewing strongly reduced, closed, its veins thick and heavy. Metasoma with segments 2 and 3 forming a syntergum	**Pycnostigminae**
	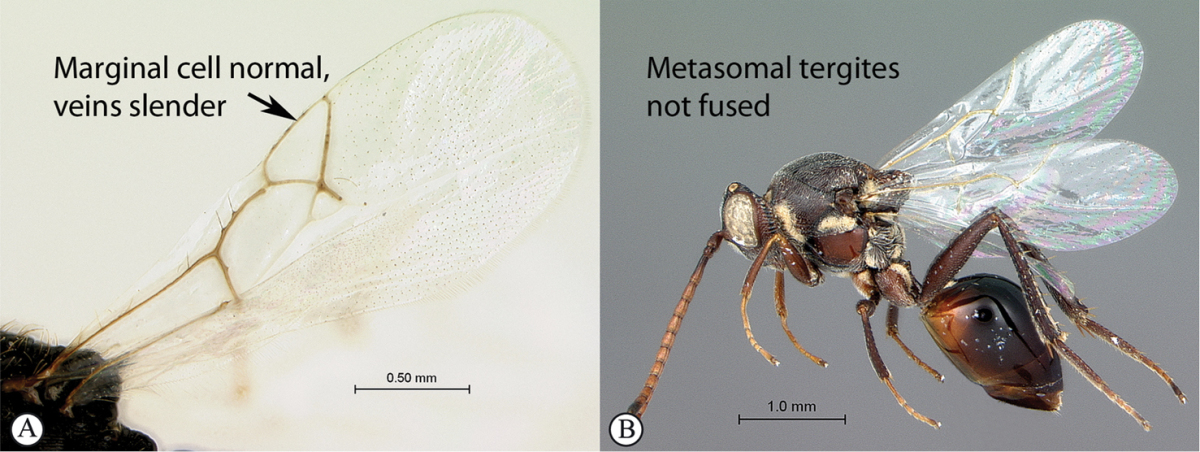	
–	Marginal cell of forewing not reduced, open or closed along anterior margin, veins typically slender (A). Metasomal tergae free (not fused) (B)	**5**
	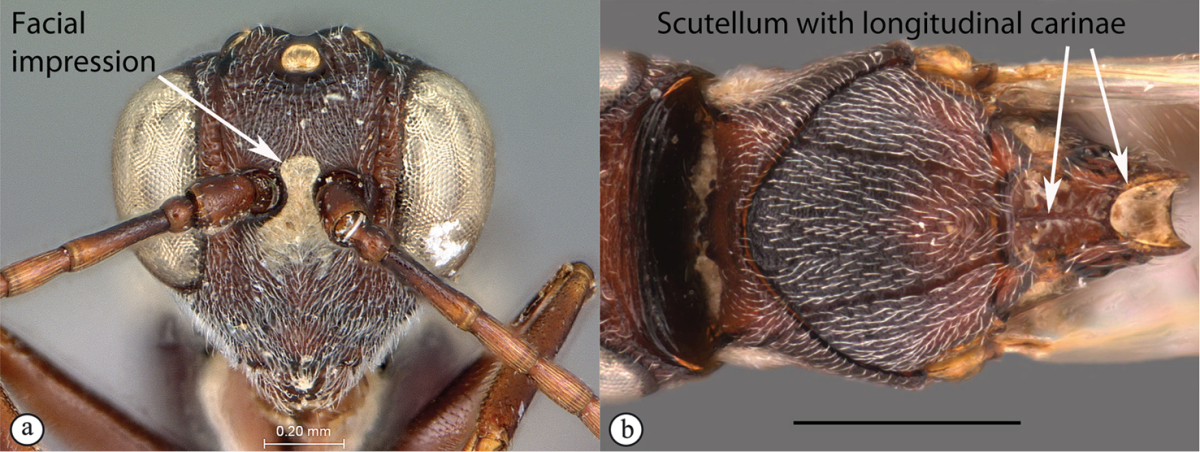	
5	Facial impression usually present between toruli (a). Tergite 2 of metasoma liguliform; hind tibiae in most genera longitudinally ridged or furrowed on outer margins or posteriorly. Scutellum with one or more longitudinal carinae and subapically with either a spine or foveae (b). Mesosoma sculptured, dull (b)	**Aspicerinae**
	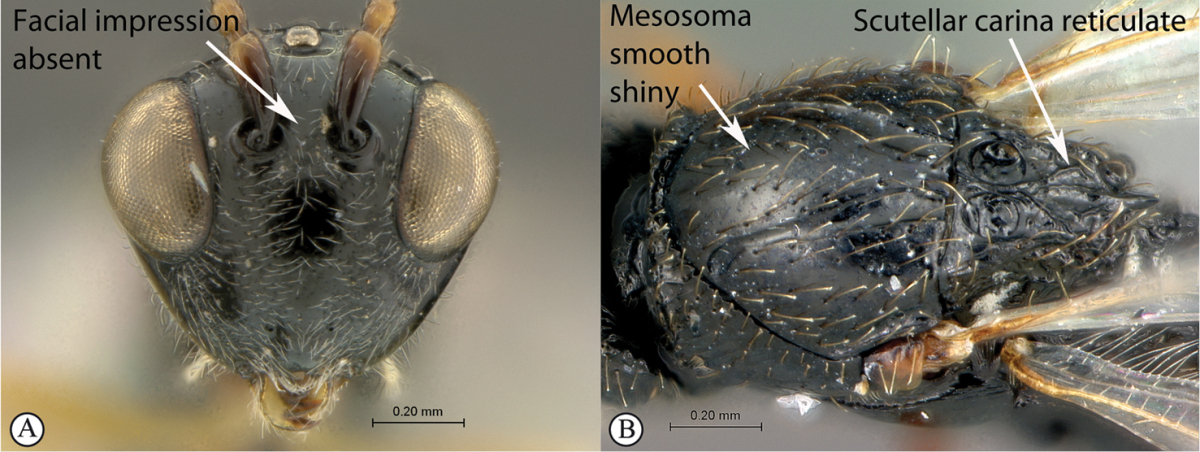	
–	Facial impression absent (A). Tergite 2 of metasoma not liguliform. Hind tibia not longitudinally ridged or furrowed externally or posteriorly, at most with longitudinal carinae or groove internally. Scutellum usually without three longitudinal carinae or subapical fovea, though more frequently produced apically to form a spine (B). Mesosoma generally smooth and shining, with notauli present (B), occasionally dull and sculptured	**6**
	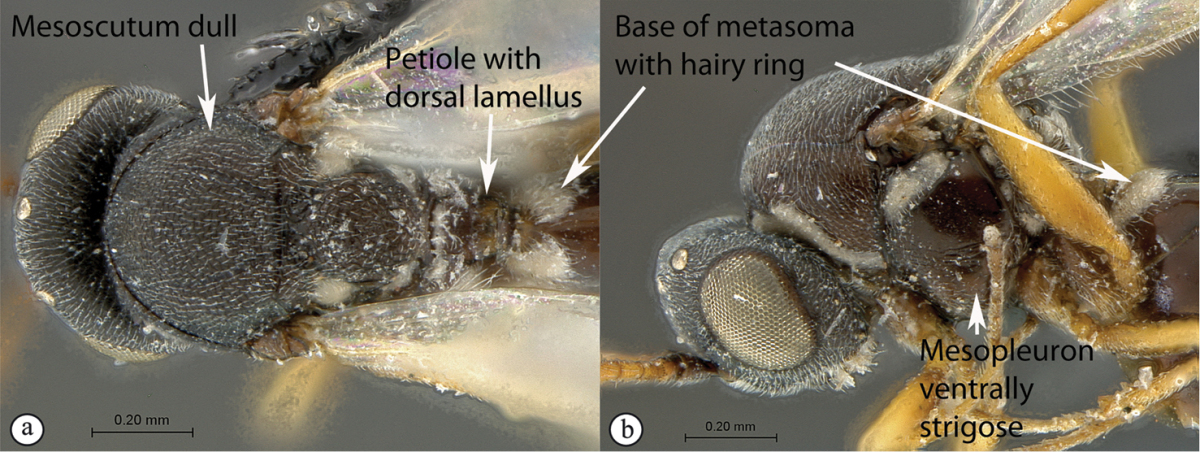	
6	Petiole with dorsal lamellus, which partially covers the junction between the petiole and the nucha (a). Base of metasoma with a complete hairy ring (a, b). Mesoscutum dull, microcoreacious (a, b). Mesopleuron dorsally smooth and shiny, ventrally strigose (b)	***Melanips* (Aspicerinae)**
	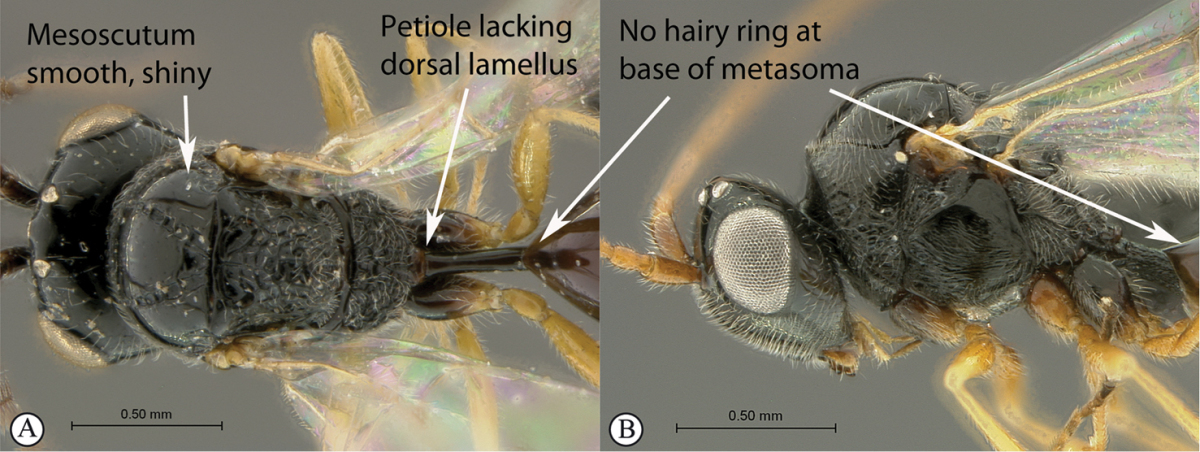	
–	Petiole lacking a dorsal lamellus, junction between petiole and nucha fully exposed (A). Base of metasoma glabrous, lacking a hairy ring (A, B). Mesoscutum lacking microcoreacious sculpture; macrosculptural elements usually present (A). Mesopleuron smooth except for distinct mesopleural line or entirely longitudinally striate (B)	**7**
	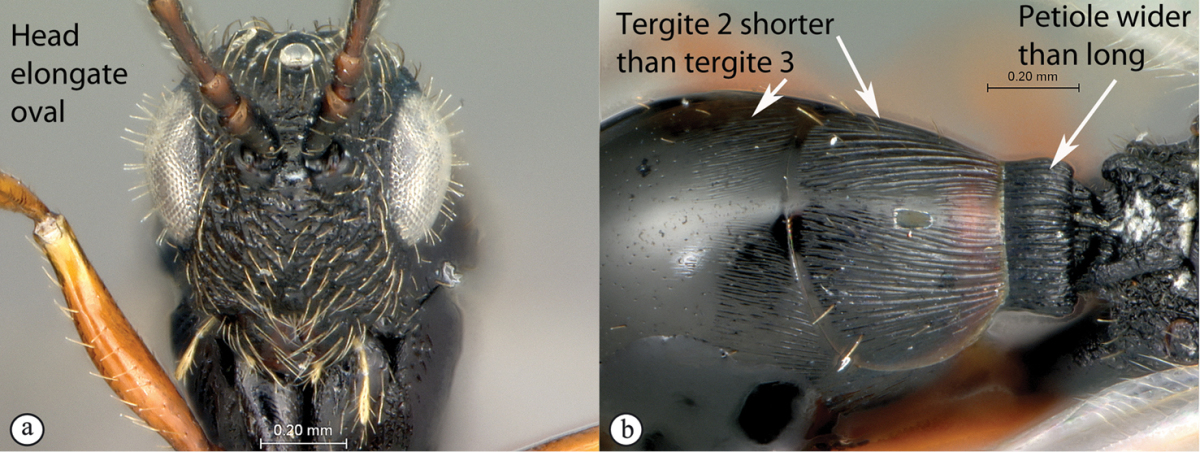	
7	Head, in anterior view, elongate oval to round, as wide as, or narrower than mesosoma (a). Tergite 2 of metasoma shorter than third; petiole never as long as wide (b)	**Figitinae**
	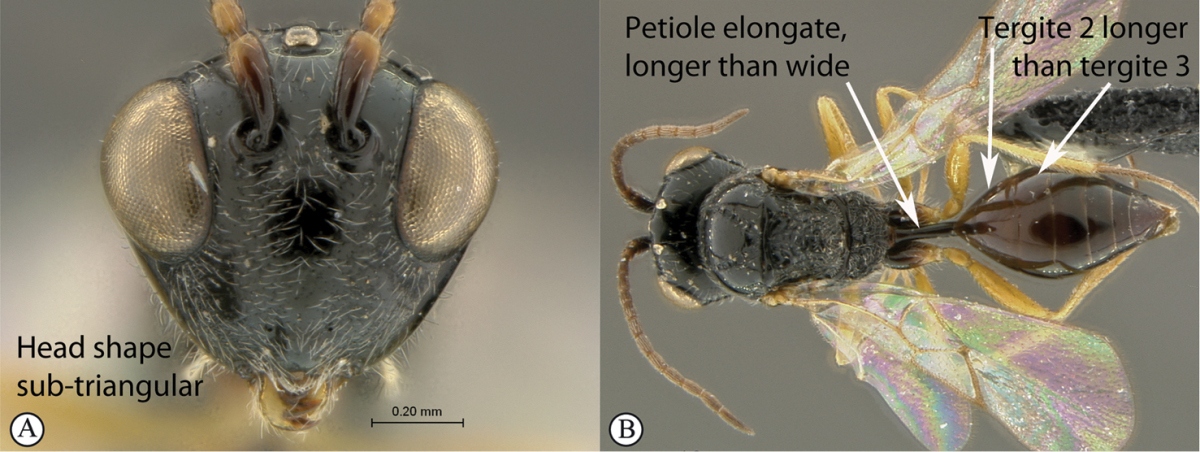	
–	Head, in anterior view, roughly triangular, occasionally wider than mesosoma (A). Tergite 2 of metasoma longer than third; petiole at least as long as wide, generally more than twice as long as wide (B)	**Anacharitinae**

### Anacharitinae

The Anacharitinae are represented in the Afrotropical region by four genera containing 3 described, and at least one undescribed species. Additional undescribed species are present in world collections.

**Biology.** Afrotropical anachritines are primary parasitoids of aphid-hunting Neuroptera larvae (Buffington et al. 2012).

**Distribution.** The subfamily is widespread, with many described species in the Holarctic and Neotropics, and fewer elsewhere. In the Afrotropical and Oceanic regions, there are certainly many species undescribed, and they are likely to be present also in the Oriental regions even though not recorded as such yet.

#### Key to Afrotropical anacharitine genera

**Table:** 

	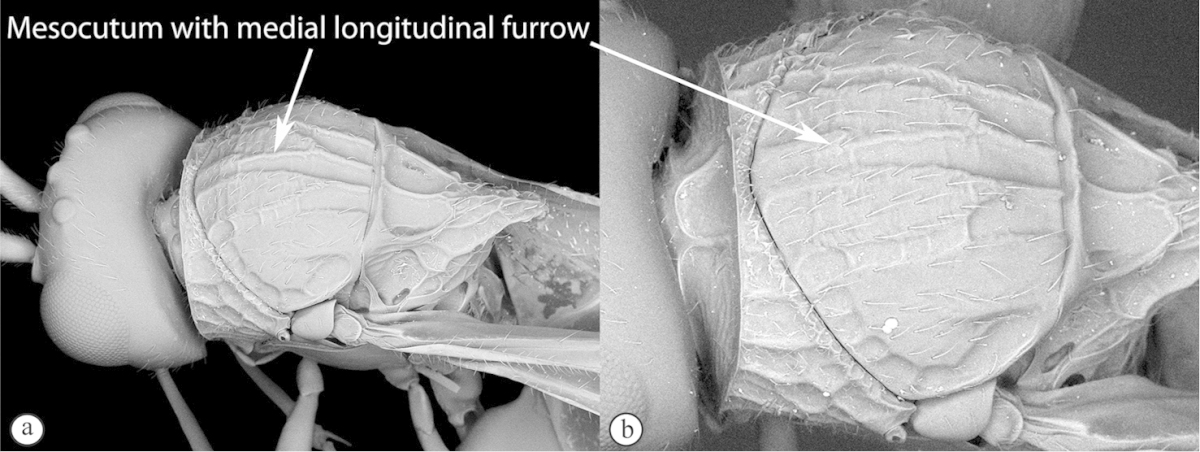	
1	Mesoscutum with medial longitudinal furrow with cross-carinae (a, b) mimicking the adjacent notauli	***Acanthaegilopsis***
	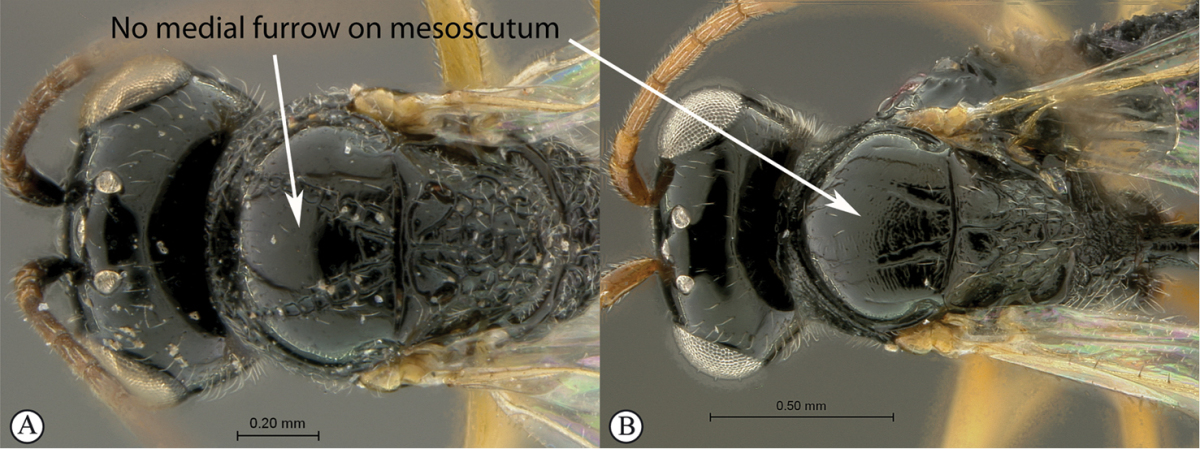	
–	Mesoscutum without medial longitudinal furrow (A, B)	**2**
	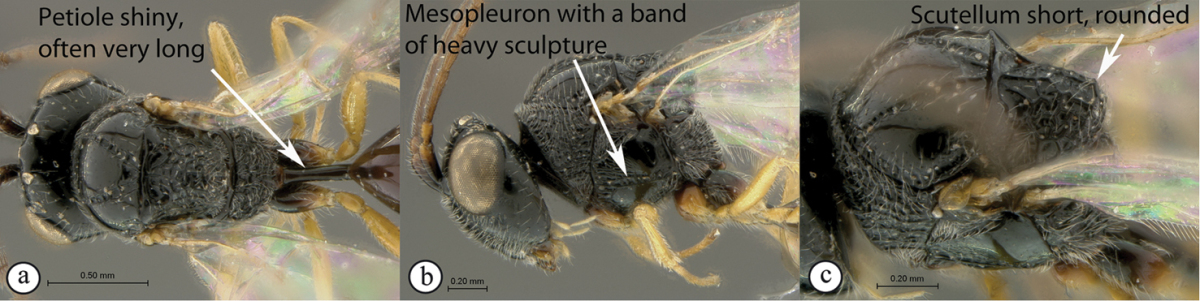	
2	Petiole shiny, often very long (a). Mesopleuron with a band of heavy sculpture (b). Scutellum short, rounded (c)	***Anacharis***
	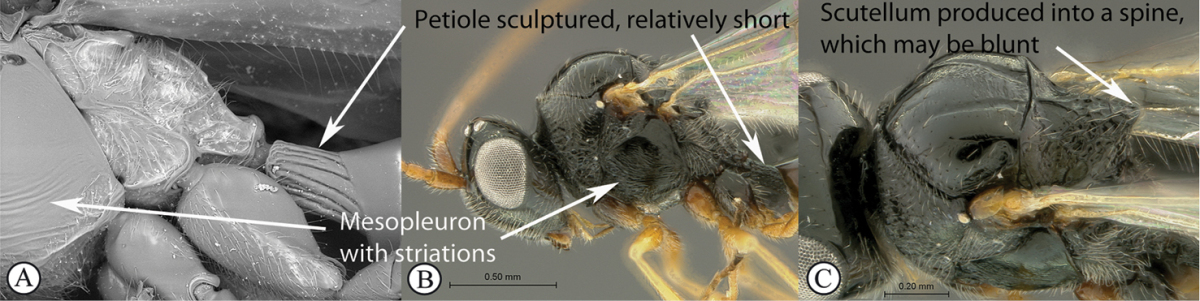	
–	Petiole sculptured, relatively short (A, B). Mesopleuron at most with striations, without a band of heavy sculpture (A, B). Scutellum produced into a spine, which may be blunt (C)	**3**
	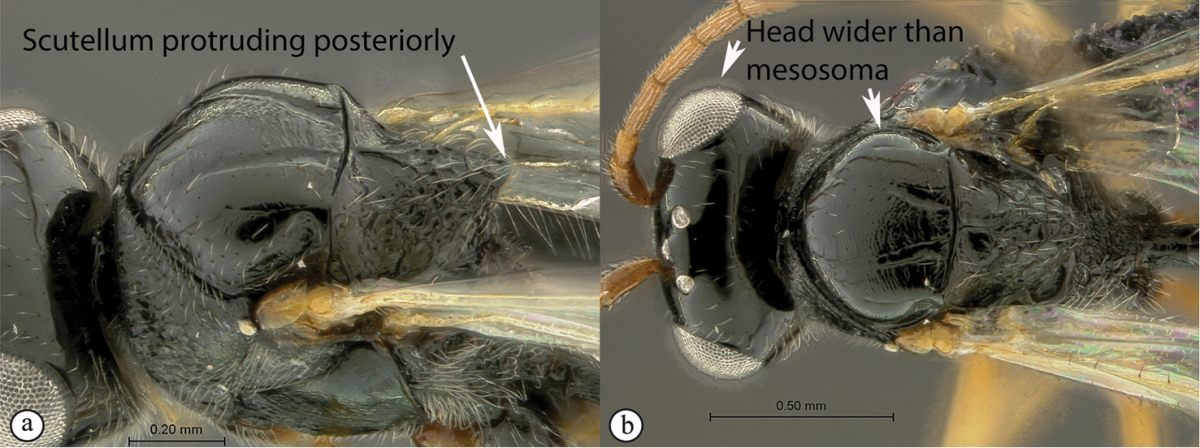	
3	Scutellum protruding posteriorly, overhanging propodeum, sometimes as a spine, with similar reticulate-foveolate sculpture over entire dorsal surface (a). Head transverse, distinctly wider than mesosoma (b)	***Xyalaspis***
	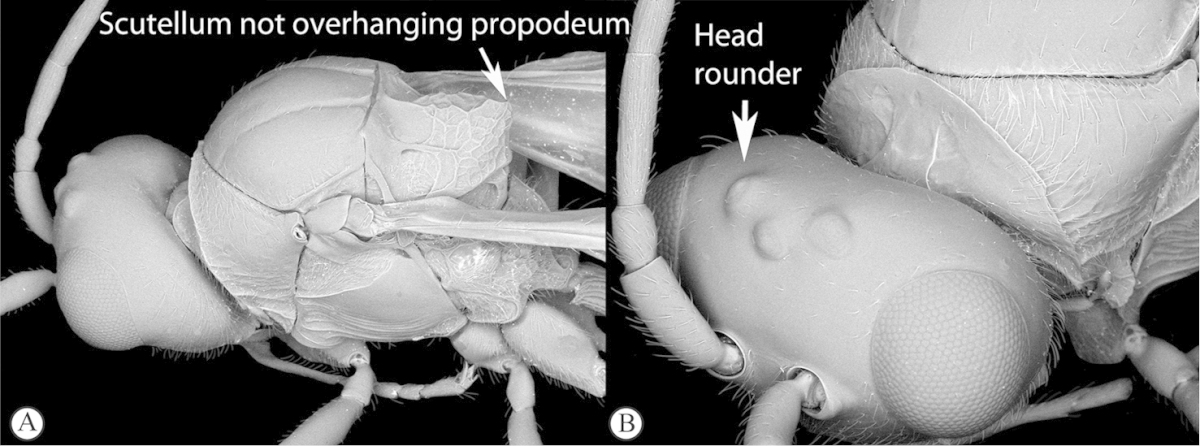	
–	Scutellum usually not overhanging propodeum, never as a distinct spine, smooth or more or less rugose, never rough reticulate (A); typically a circumscutellar carina can be traced around the dorsal circumference of the scutellum. Head rounded, only slightly wider than mesosoma (B)	***Aegilips***

#### Acanthaegilopsis

Taxon classificationAnimaliaHymenopteraFigitidae

Pujade-Villar, 2013

##### Remarks.

Recently described genus endemic to the Afrotropical region.

##### Diagnosis.

Immediately distinguishable from other Afrotropical anacharitines by the presence of a distinct and complete medial longitudinal furrow containing cross-carinae on the mesoscutum. The ventral part of the mesopleuron is coriaceously sculptured, a unique anacharitine character state.

**Figure 5.**
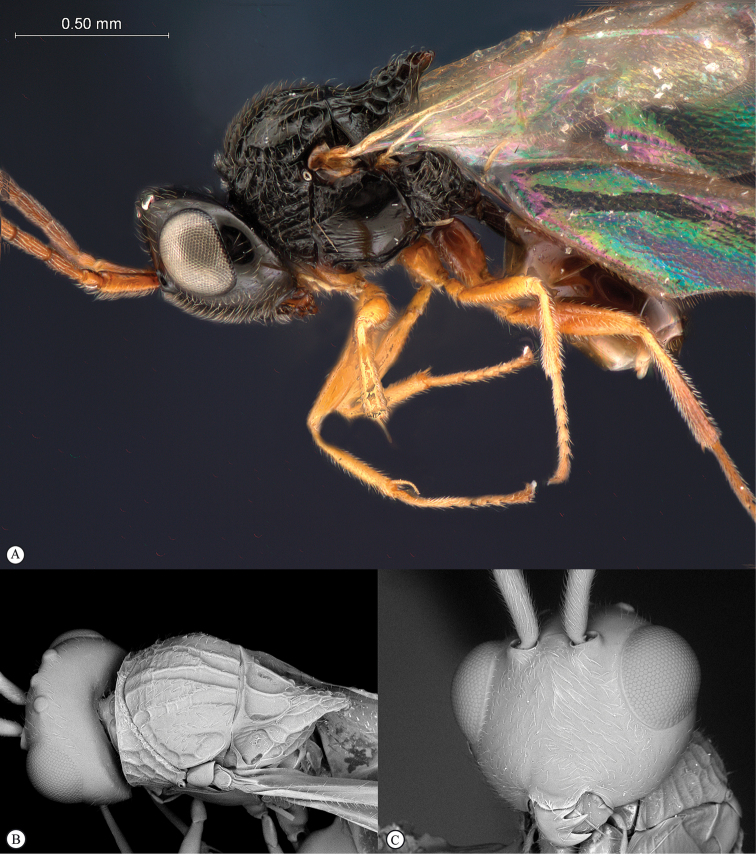
*Acanthaegilopsis* species (Kenya). **A** habitus lateral view **B** head and mesosoma dorsolateral view **C** head, anterior-lateral view.

##### Distribution.

Afrotropical region: Comoros, Madagascar, Uganda, Zimbabwe (Mata-Casanova and Pujade-Villar 2013; Mata-Casanova et al. 2014), Kenya (here).

##### Biology.

Unknown.

##### Species richness.

*Acanthaegilopsis hemicoriaceus* Mata-Casanova & Pujade-Villar, 2014 (Uganda, Zimbabwe)

*Acanthaegilopsis malagasy* Pujade-Villar & Mata-Casanova, 2013 (Comoros, Madagascar)

#### Aegilips

Taxon classificationAnimaliaHymenopteraFigitidae

Walker in Haliday, 1835

##### Remarks.

Rare in Afrotropical region. The genus is often difficult to separate from *Xyalaspis*, and requires revision.

##### Diagnosis.

A variable and rather unsatisfactorily circumscribed genus. Some representatives are quite similar to *Xyalaspis* while some have more of the superficial appearance of small Figitinae. The scutellum may be pointed posteriorly but forms far less of a spine, and is less strongly foveolate so that a circumscutellar carina may follow all the way around the scutellum. Head is less transversal and triangular than in other Anacharitinae.

**Figure 6.**
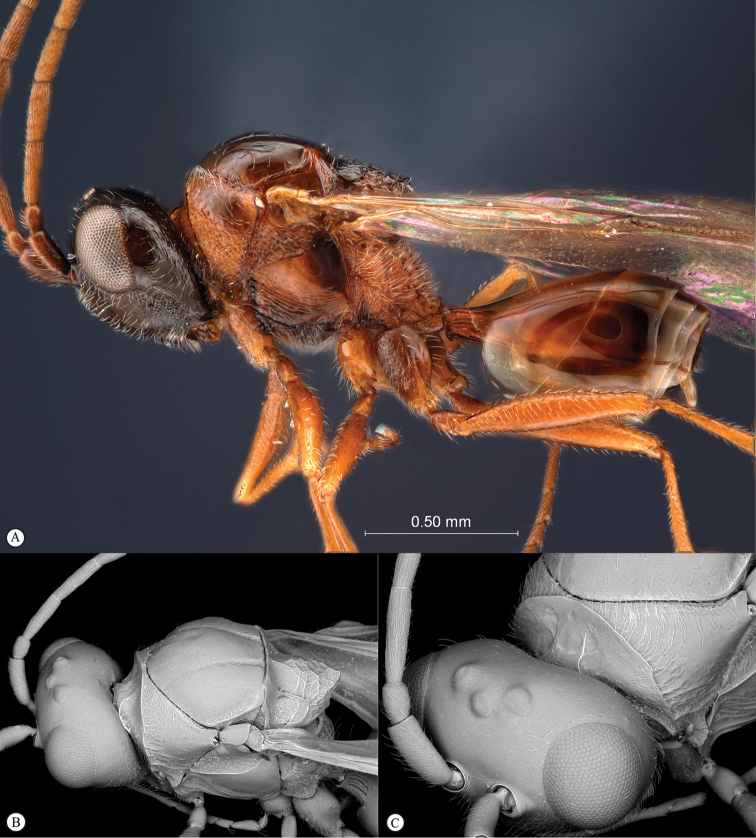
*Aegilips* species (Kenya). **A** habitus lateral view **B** head and mesosoma dorsolateral view **C** head, pronotal plate anteriolateral view.

##### Distribution.

Mainly Holarctic, but present locally also in the Neotropical and Afrotropical regions. Afrotropical records: Democratic Republic of Congo, Kenya, Zimbabwe (here).

##### Biology.

Parasitoids of aphidivorous Neuroptera larvae (Fergusson 1986).

##### Species richness.

Only undescribed species in the Afrotropical region, as Kieffer’s *Aegilips capensis* (at current state of knowledge) is a *Xyalaspis*.

#### Anacharis

Taxon classificationAnimaliaHymenopteraFigitidae

Dalman, 1823

##### Remarks.

Rare in Afrotropical region.

##### Diagnosis.

Characteristic anacharitines with elongate, smooth petiole and distinctly transverse and triangular head. The scutellum does not overhang the propodeum; it has more or less reticulate sculpture (never strongly foveolate) and often has a posterior carina that forms a distinct posterodorsal edge. The mesopleura are typically more sculptured than in other Anacharitinae, and the metasoma ends in a more pointed way (the others typically more abruptly).

**Figure 7.**
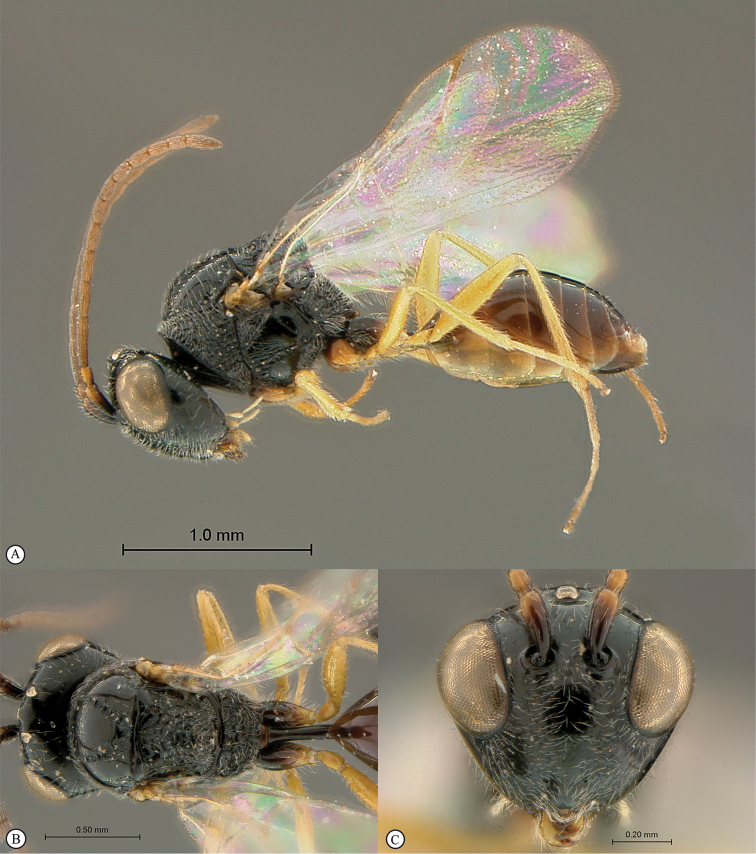
*Anacharis* species (South Africa). **A** habitus lateral view **B** head and mesosoma dorsal view **C** head, anterior view.

##### Distribution.

Mainly Holarctic, but also common in Australia and New Zealand, and locally present in Afrotropical region. Afrotropical records: Madagascar (Pujade-Villar 2012), Democratic Republic of Congo, Kenya, South Africa, Uganda (here).

##### Biology.

Parasitoids of aphidivorous Neuroptera larvae. (Fergusson 1986, Miller and Lambdin 1985, Cave and Miller 1987).

##### Species richness.

*Anacharis madagascarensis* Pujade-Villar, 2012 (Madagascar)

Several undescribed species from elsewhere in Africa.

#### Xyalaspis

Taxon classificationAnimaliaHymenopteraFigitidae

Hartig, 1843

##### Remarks.

Not common in the Afrotropical region. The genus is often difficult to separate from *Aegilips* and requires revision.

##### Diagnosis.

*Xyalaspis* are often easy to recognise by their very distinct scutellar spines, but several taxa have more blunt or moderate spines that are close to character states found in *Aegilips*. In these cases, the scutellum of *Xyalaspis* is characterised by a heavier foveolate sculpture, where no circumscutellar carina is obvious. As currently circumscribed, the genus is somewhat heterogenous in the Afrotropical region, and a distinct species group is characterised by a strongly sculptured mesoscutum with longitudinal carinae as well as strong genal carinae.

**Figure 8.**
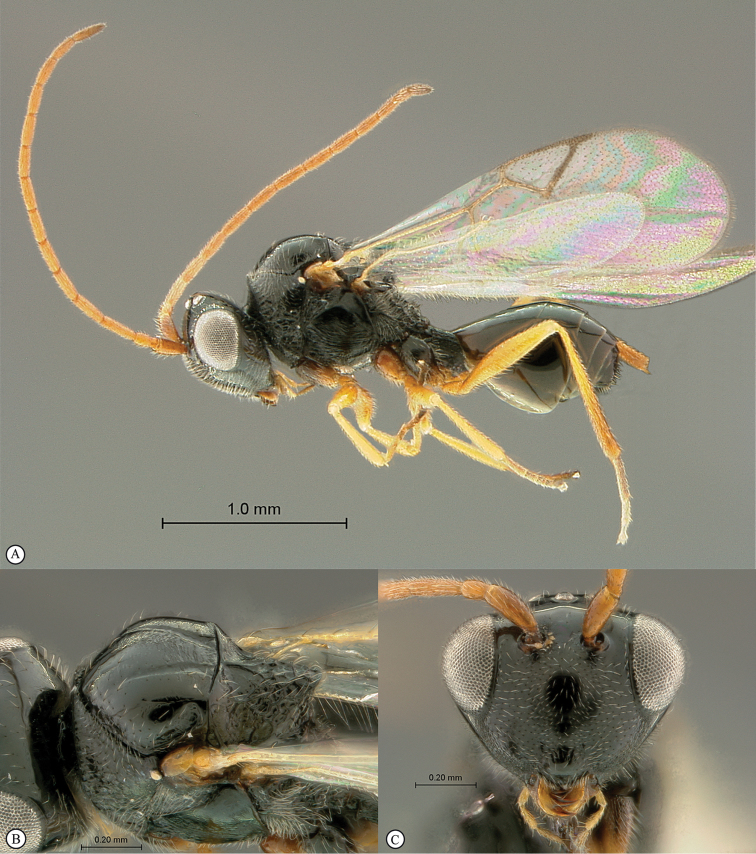
*Xyalaspis* species (South Africa). **A** habitus lateral view **B** head and mesosoma dorsolateral view **C** head, anterior view.

##### Distribution.

Mainly Holarctic, but also present throughout the Old World Tropics. Afrotropical records: South Africa (Kieffer 1912), Nigeria, Uganda (Mata-Casanova et al. 2014), Cameroon, Democratic Republic of Congo, Madagascar, Malawi, Yemen, Zimbabwe (here).

##### Biology.

Parasitoids of aphidivorous Neuroptera larvae (Fergusson 1986, Miller and Lambdin 1985).

##### Species richness.

*Xyalaspis capensis* (Kieffer, 1912), **comb. n.** (*Aegilips*) (South Africa, Zimbabwe) (Type supposedly in ZMBH, but not found there. However, the original description has been deemed sufficient for generic placement here)

*Xyalaspis subsaharica* Mata-Casanova & Pujade-Villar, 2014 (Nigeria, Uganda)

Several undescribed species from elsewhere in the region.

### Aspicerinae

The Aspicerinae are represented in the Afrotropical region by four genera containing 13 described species. A number of undescribed species are present in world collections and await description.

**Biology.** Afrotropical aspicerines are primary parasitoids of aphidophagous Syrphidae and Chamaemyiidae larvae (Diptera) (Buffington and van Noort 2009, Buffington et al. 2012).

**Distribution.** The subfamily is widespread. Although the majority of described species are Palearctic this is a biased distribution with both the Nearctic and Neotropical faunas probably being more diverse than the Palearctic. The species numbers in the Afrotropical and Oriental regions are significantly lower (but with many undescribed species), while the subfamily has not yet been recorded at all in the Oceanic region.

#### Key to Afrotropical aspicerine genera

**Table:** 

	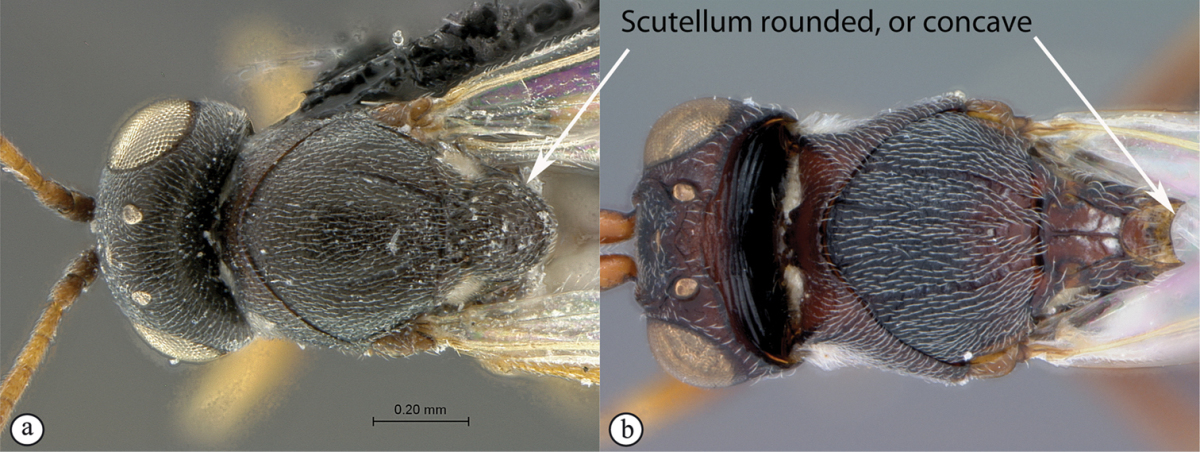	
1	Posterior part of scutellum rounded (a), or concave (b)	**2**
	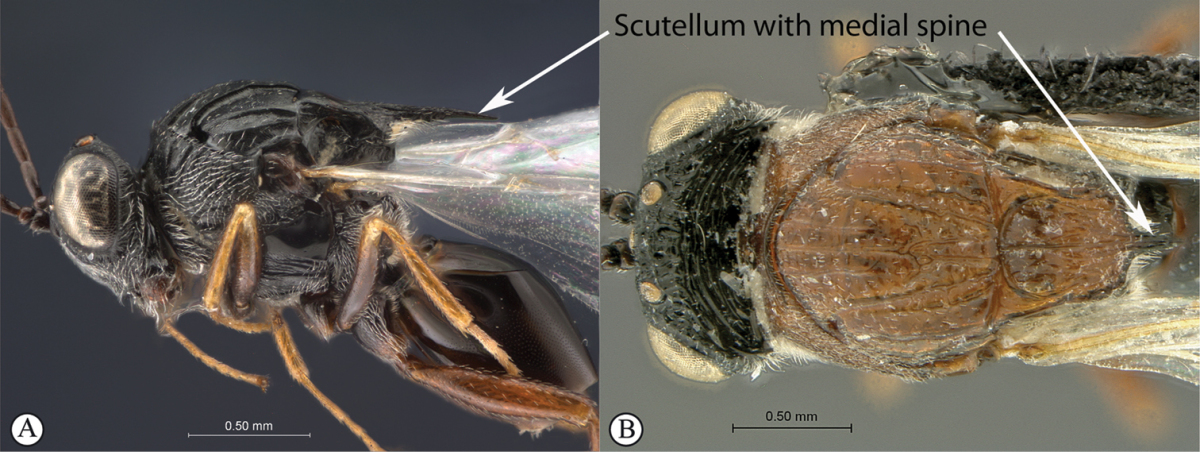	
–	Posterior part of scutellum with a medial spine (A, B)	**3**
	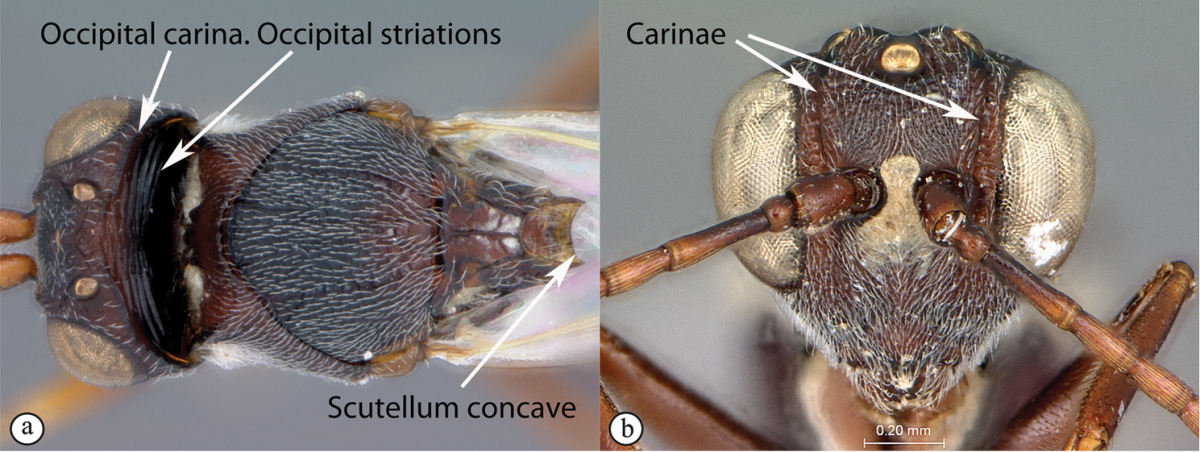	
2	Posterior part of scutellum concave (a); scutellum with lateral ridges (a). Occipital carina present dorsally (medially interrupted) (a); occiput with transverse striations (a). Face with lateral carinae extending from lateral ocelli to antennal scrobes (b)	***Anacharoides***
	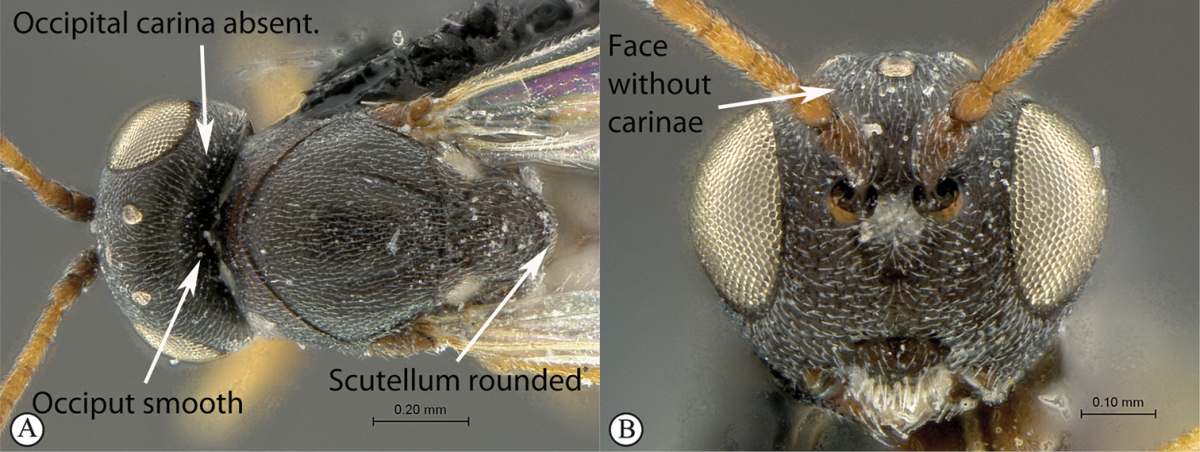	
–	Posterior part of scutellum rounded (A); scutellum smooth without lateral ridges (A). Occipital carina dorsally absent; occiput smooth (A). Face without carinae (B)	***Melanips***
	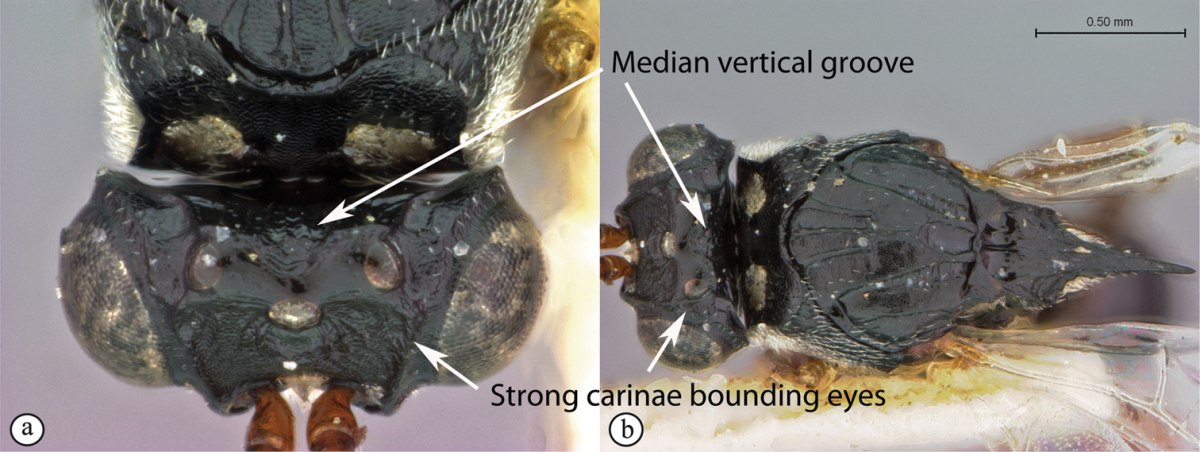	
3	Vertex with a median vertical groove backwards to occiput (a, b). Carinae extend from posterior ocelli forwards to antennae (a). Ocelli raised. Compound eyes bounded by a strong continuous carina (a, b)	***Prosaspicera***
	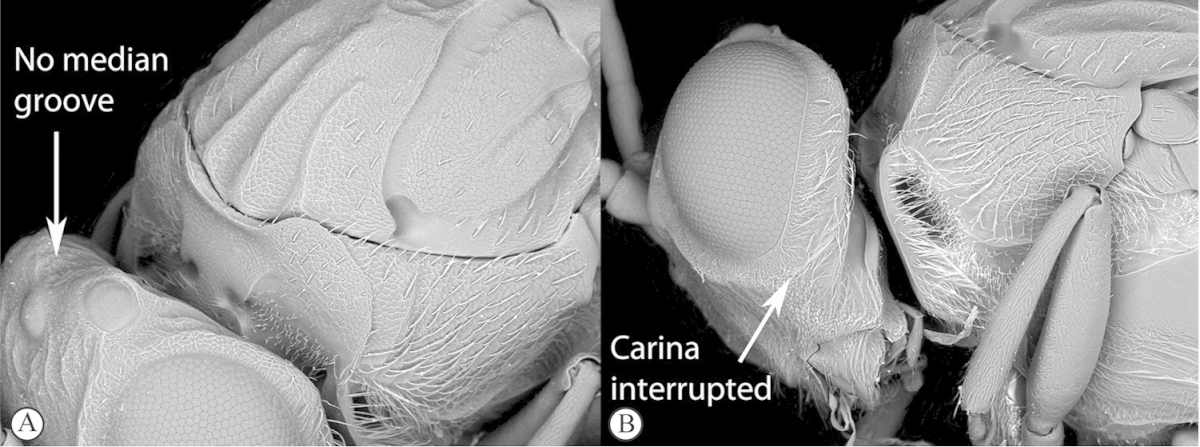	
–	Vertex without a groove (B). Ocelli hardly raised. Carina surrounding compound eyes always interrupted near malar space (A)	***Aspicera***

#### Anacharoides

Taxon classificationAnimaliaHymenopteraFigitidae

Cameron, 1904

Anacharoides (synonym: *Coelonychia* Kieffer, 1910d)

##### Remarks.

Not common. Revised by Buffington and van Noort (2009).

##### Diagnosis.

This genus is immediately separable from all other Figitidae by the distinctive scutellar depression bounded by a pair of sharp, postero-dorsal triangular projections. The elongate petiole is somewhat variable within Aspicerinae, though the state in *Anacharoides* is longer than in most other genera. The only two taxa *Anacharoides* may be confused with are *Callaspidia* and *Pujadella*; both of these latter genera have mesoscutal sculpturing that is remininscent of *Anacharoides*; however, close examination of the scutellar morphology easily separates these taxa (Buffington and van Noort 2009). *Callaspidia* has not been recorded from the Old World Tropics; *Pujadella* has been collected in Thailand (Buffington pers. obs.) and southern China (Ros-Farré 2007).

**Figure 9.**
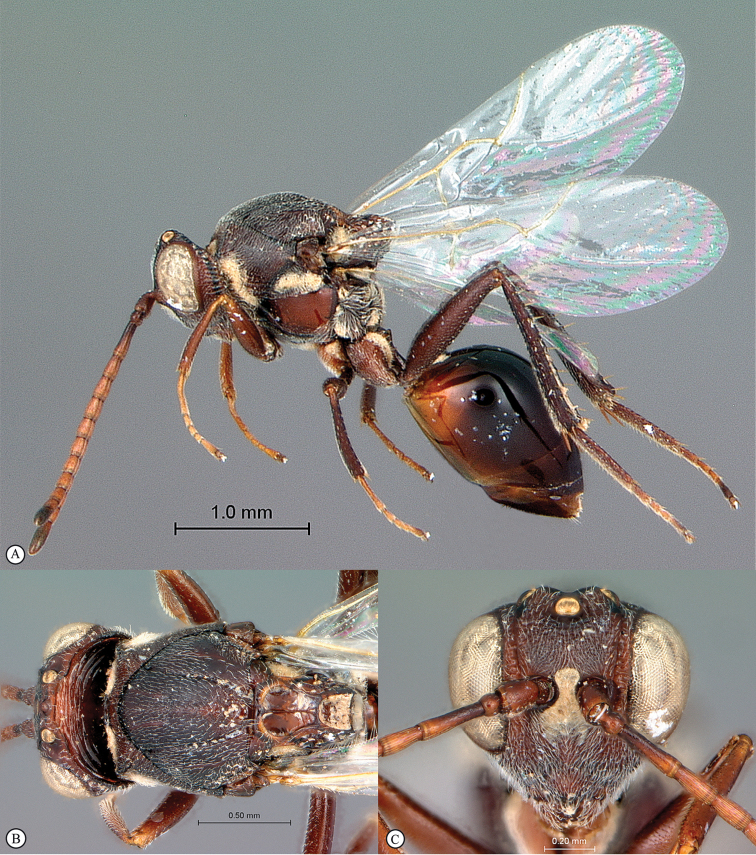
*Anacharoides pallida* (South Africa). **A** habitus lateral view **B** head and mesosoma dorsal view **C** head, anterior view.

##### Distribution.

Almost endemic to the Afrotropical region with extralimital distribution in the Canary Islands. Afrotropical records: Angola, Democratic Republic of Congo, Ethiopia, Ghana, Kenya, Madagascar, Nigeria, Rwanda, Senegal, Sierra Leone, South Africa, Tanzania, Uganda, Yemen, Zimbabwe (Buffington and van Noort, 2009), Central African Republic, Guinea-Bissau, Malawi (here).

##### Biology.

Parasitoids of aphidivorous Brachycera larvae (Buffington and van Noort 2009).

##### Species richness.

*Anacharoides nicknacki* Buffington & van Noort, 2009 (Cameroon, Kenya, Malawi, Rwanda, South Africa, Uganda, Zimbabwe)

*Anacharoides pallida* Quinlan, 1979 (Ethiopia, South Africa; extralimital distribution in the Canary Islands)

*Anacharoides paragi* Benoit, 1956c (Democratic Republic of Congo, Ethiopia, Ghana, Nigeria, Sierra Leone, Uganda, Zimbabwe)

*Anacharoides quadrus* Quinlan, 1979 (Ethiopia, Uganda, Zimbabwe)

syn *Anacharoides astrida* Quinlan, 1979

*Anacharoides rufus* (Kieffer, 1912) (*Coelonychia*) (Ethiopia, South Africa) (identity uncertain)

*Anacharoides striaticeps* Cameron, 1904 (Angola, Ethiopia, Kenya, Nigeria, Rwanda, Senegal, South Africa, Uganda, Yemen, Democratic Republic of Congo, Zimbabwe)

syn *Anacharoides arcus* Quinlan, 1979

syn *Anacharoides decellius* Quinlan, 1979

syn *Anacharoides elongaticornis* Benoit, 1956c

syn *Anacharoides eurytergis* Benoit, 1956c

syn *Anacharoides gibbosus* Benoit, 1956c

syn *Anacharoides nigra* Quinlan, 1979

syn *Anacharoides sanitas* Quinlan, 1979

syn *Coelonychia spinosipes* Kieffer, 1910d

syn *Anacharoides suspensus* Quinlan, 1979

*Anacharoides stygius* Benoit, 1956c (Democratic Republic of Congo, Ethiopia, Madagascar, Nigeria, Tanzania)

#### Aspicera

Taxon classificationAnimaliaHymenopteraFigitidae

Dahlbom, 1842

##### Remarks.

Rare in the Afrotropical region.

##### Diagnosis.

This taxon can be difficult to seperate from *Prosaspicera*. *Aspicera hartigi*, the only species recorded from the Afrotropical region thus far, has a much shorter scutellar spine than *Prosaspicera*. Additionally, *Aspicera* lacks the characteristic inner-orbital carina that *Prosaspicera* has. Finally, *Aspicera hartigi* (from Yemen) is distinctly bi-chromatic, with a orange-brown mesoscutum and black mesopleuron; African *Prosaspicera*, to our knowledge, are all black. *Neralsia* and *Xyalophora* are Figitinae and lack the characteristic ligulate T2 of the metasoma that aspicerines have. Furthermore, *Neralsia* and *Xyalophora* lack the setiferous pit on the frons, a putative defining feature of Aspicerinae (Ros-Farré et al. 2000).

**Figure 10.**
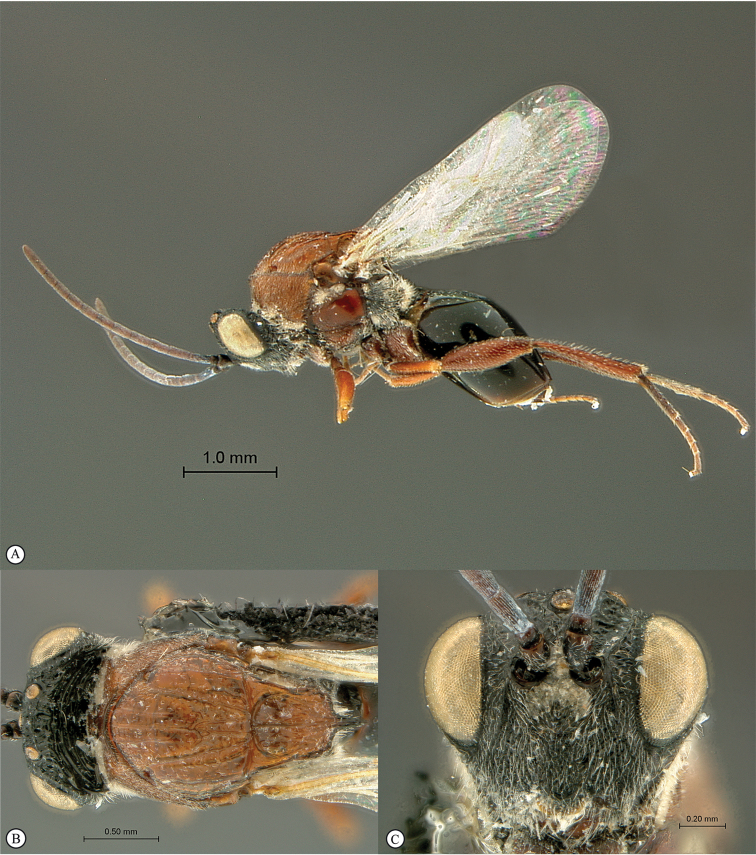
*Aspicera hartigi* (Yemen). **A** habitus lateral view **B** head and mesosoma dorsal view **C** head, anterior view.

##### Distribution.

Mainly Holarctic, but marginally present in the Afrotropical region. Afrotropical records: Yemen (here).

##### Biology.

Ros-Farré and Pujade-Villar (2013) report, based on label data, that *Aspicera dianae* Ros-Farré, 2013 emerged from the syrphid *Metasyrphus vinelandi* (Curran, 1921). Ronquist (1999) and Weld (1952) report *Aspicera* species are parasitoids of aphidivorous Syrphidae and Chamaemyiidae, but these rearing records await confirmation.

##### Species richness.

*Aspicera hartigi* Dalla Torre, 1889 (Yemen). This species has also been reported from the UAE (Buffington 2010 as *Aspicera* sp.), Saudi Arabia and Algeria (here)

#### Melanips

Taxon classificationAnimaliaHymenopteraFigitidae

Walker in Haliday, 1835

##### Remarks.

Rare in the Afrotropical region; likely non-native to the region. The classification of this taxon is unstable. Recently, Buffington et al. (2007) moved it to Aspicerinae from Figitinae. As reflected in the key to subfamilies, the taxon does not neatly fit into either subfamily, and possesses a plesiomorphic morphotype reminiscent of Thrasorinae (Australasian), Plectocynipinae (Neotropical) and some Cynipidae. Rearing records (summarized below) as well as phylogenetic analyses suggest this taxon is more closely related to the genera in Aspicerinae, and we maintain that classification here.

##### Diagnosis.

Distinguished from other Figitidae by the characteristic ‘clam-shell’ petiolar lamina present on the dorsal half of the petiole. This lamina can often cover the junction between the petiole and the nucha. Superficially, *Melanips* appears to be a cynipid, but can be distinguished from the Afrotropical cynipids by lacking an areola in the forewing, and by having a dorsally smooth mesopleuron. In addition, *Melanips* has a setose mesoscutum.

**Figure 11.**
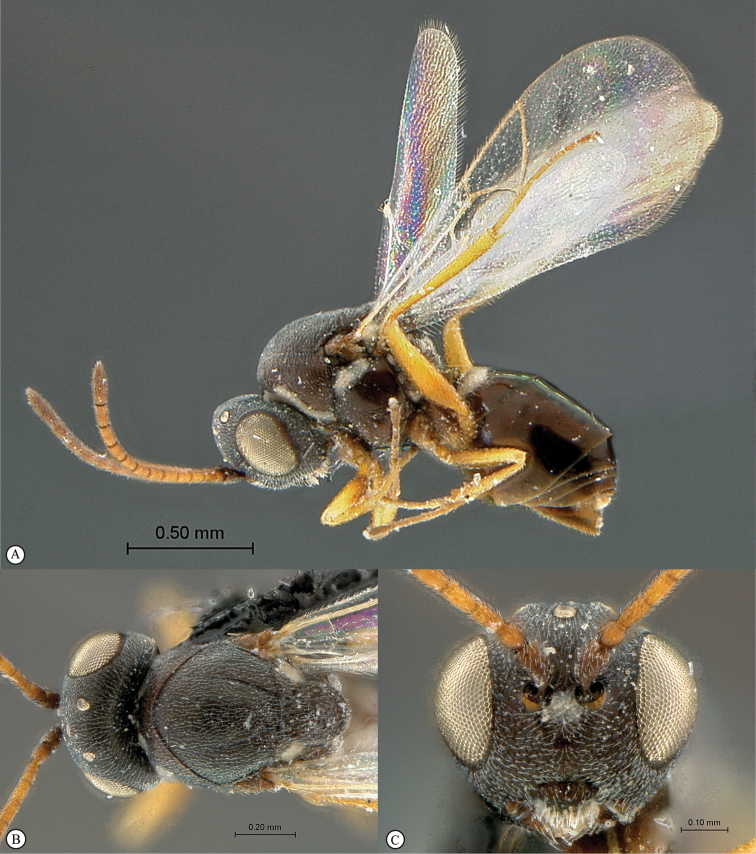
*Melanips alienus* (Yemen). **A** habitus lateral view **B** head and mesosoma dorsal view **C** head, anterior view.

##### Distribution.

Mainly Holarctic but transgressing into the Old World Tropics; in the Afrotropical region found so far only in Kenya and Yemen (here) but expected to show up elsewhere.

##### Biology.

Parasitoid of aphidivorous Brachycera larvae (Evenhuis 1968, Fergusson 1986, Buffington et al. 2012); label data of several specimens in BMNH (from Kenya and India) records them as reared from *Lecopis* (Chamaemyiidae), some with host remains.

##### Species richness.

*Melanips alienus* Giraud, 1860 (Kenya, Yemen; extralimital distribution: widespread in Europe and North Africa)

#### Prosaspicera

Taxon classificationAnimaliaHymenopteraFigitidae

Kieffer, 1907

##### Remarks.

Revised by Díaz (1979) and by Ros-Farré and Pujade-Villar (2006).

##### Diagnosis.

This taxon is most easily confused with Afrotropical *Aspicera*, but can be distinguished from that taxon by having a much longer scutellar spine, easily as long as the petiole and distinctly overhanging it (much shorter in *Aspicera*, not overhanging the petiole). Further, *Aspicera* has not yet been recorded from equatorial Africa, and appears to be restricted to arid portions of Mediterranean Africa and the southern Arabian Peninsula (here). *Prosaspicera* can, to a lesser extent, be confused with the figitines *Neralsia* and *Xyalophora* (all having reasonably well-developed scutellar spines); however, figitines lack the ligulate metasomal T2, and well as the facial impression, and these two characters separate *Prosaspicera* from figitines with scutellar spines.

**Figure 12.**
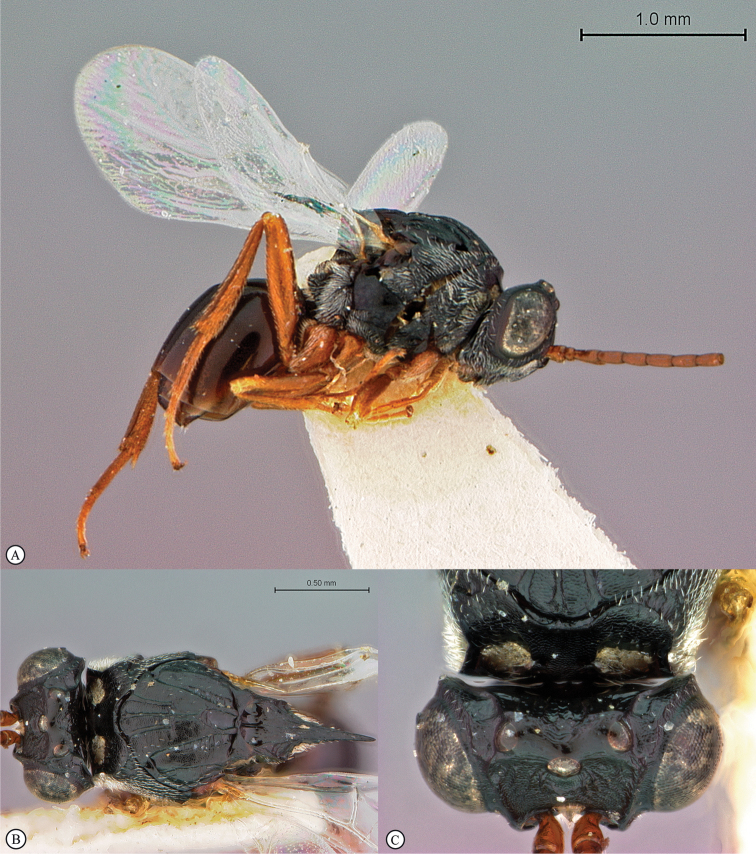
*Prosaspicera* species (South Africa). **A** habitus lateral view **B** head and mesosoma dorsal view **C** head, dorsal view.

##### Distribution.

Mainly pantropical, but extending into the southern Nearctic and the southeastern Palearctic. Afrotropical records: Democratic Republic of Congo, Ethiopia, Ghana, Malawi, Nigeria, Sierra Leone, Zimbabwe (Ros-Farré and Pujade-Villar 2006); South Africa (here).

##### Biology.

Parasitoid of aphidivorous Brachycera larvae (Syrphidae: Ros-Farré and Pujade-Villar 2006). USNM has two specimens from Nigeria reared from *Paragus* (Syrphidae) on cotton.

##### Species richness.

*Prosaspicera antennata* (Benoit, 1956c) (*Aspicera*) (Democratic Republic of Congo, Ethiopia)

*Prosaspicera optiva* Quinlan, 1979 (Democratic Republic of Congo, Ethiopia)

*Prosaspicera paragicida* (Benoit, 1956c) (*Aspicera*) (Democratic Republic of Congo, Ethiopia)

*Prosaspicera tropica* (Kieffer, 1910d) (*Aspicera*) (Democratic Republic of Congo, Ethiopia, Ghana, Malawi, Nigeria, Sierra Leone, South Africa, Zimbabwe)

syn *Aspicera africana* Kinsey, 1919

syn *Aspicera kisantua* Benoit, 1956c

### Charipinae

The Charipinae are represented in the Afrotropical region by four genera containing 19 described species. The African fauna was reviewed by Ferrer-Suay et al. (2013). A number of undescribed species are present in world collections and await description.

**Biology.** Afrotropical charipines are hyperparasitoids, attacking braconids and aphelinids in aphids, or encyrtids in psyllids (Ferrer-Suay et al. 2012, Buffington et al. 2012).

**Distribution.** The subfamily is represented in all biogeographical regions with the majority of species occurring in the Holarctic (Ferrer-Suay et al. 2012).

#### Key to Afrotropical charipine genera

**Table:** 

	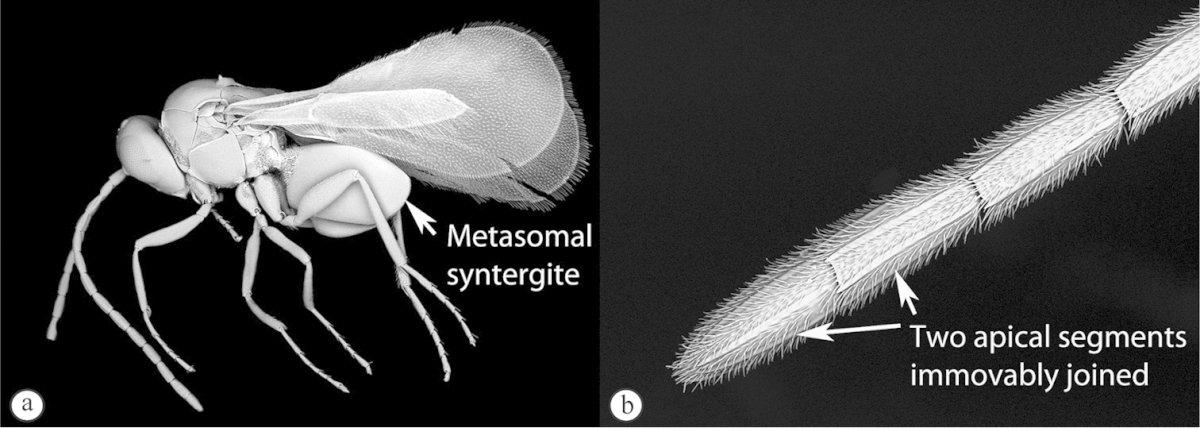	
1	Metasoma with a syntergite (sometimes with second tergite separate as a small anterior ring, but no posterior tergites visible normally) (a). Two apical segments of female antennae immovably conjoined (b). Wing veins do not reach wing edge (a)	**2**
	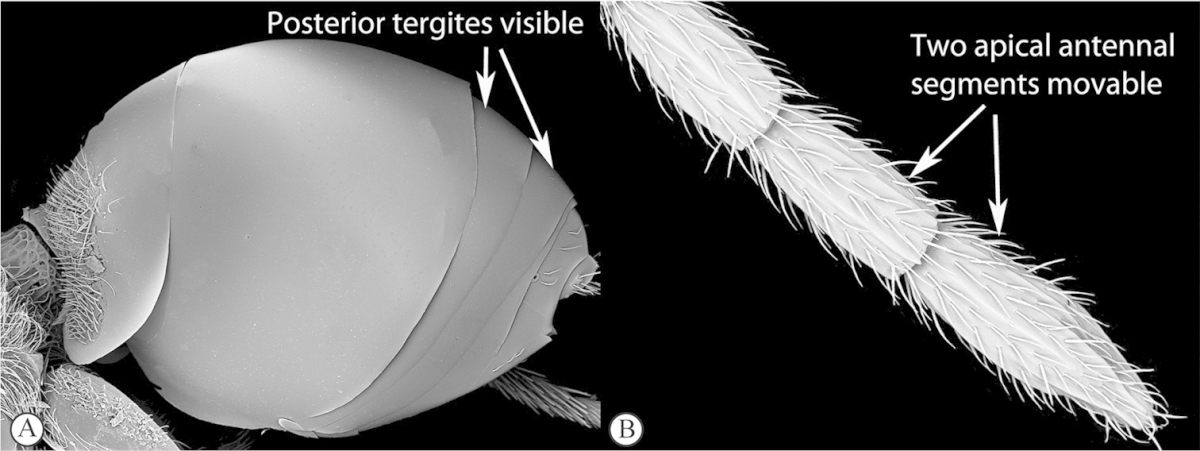	
–	Metasoma with posterior tergites visible (A). The two apical segments of antennae always movable (B). Wing veins reach wing edge	**3**
	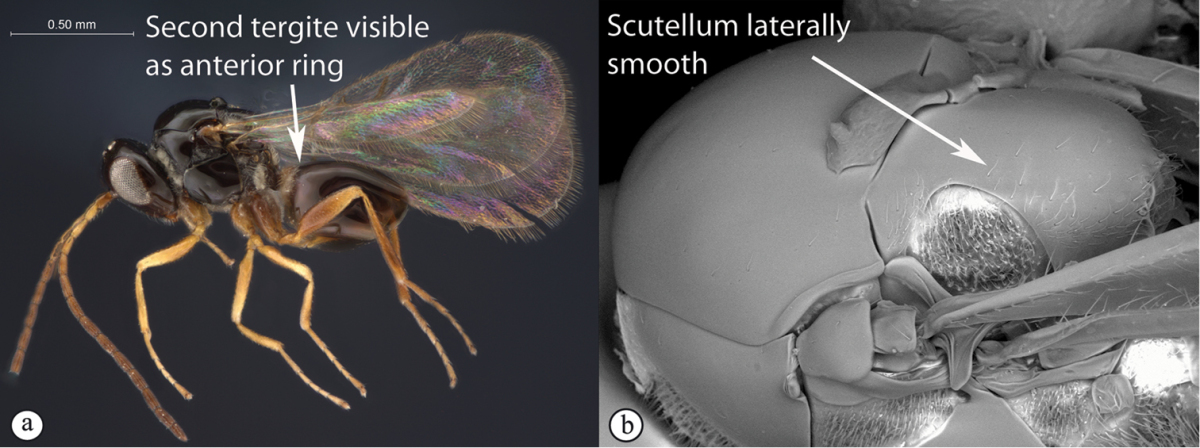	
2	Second tergite separate as a small anterior ring (a). Scutellum laterally smooth; apical ridge small (b). Subcosta running near wing edge. Common	***Dilyta***
	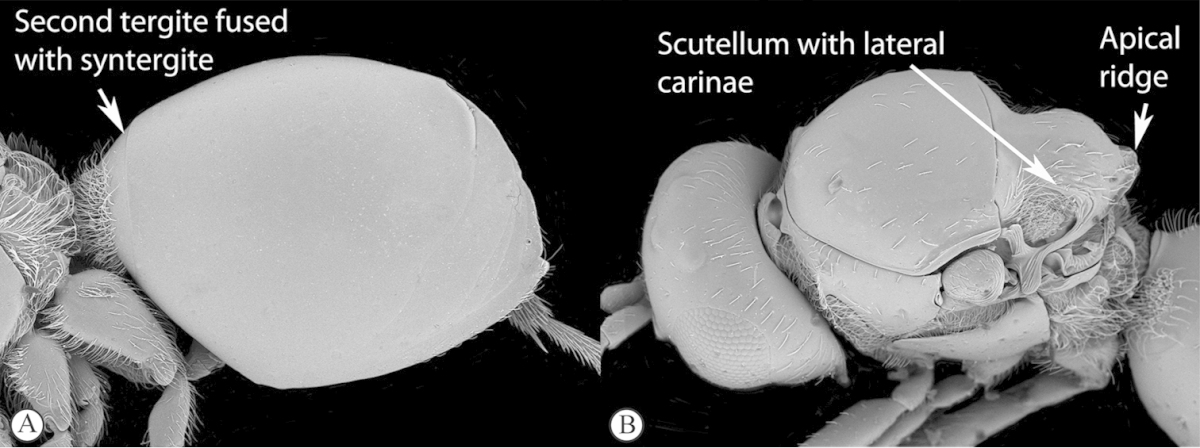	
–	Second tergite merged with syntergite (A). Scutellum with lateral ridges (B) and pronounced apical ridge (B). Subcosta running near middle of wing. Rare	***Apocharips***
	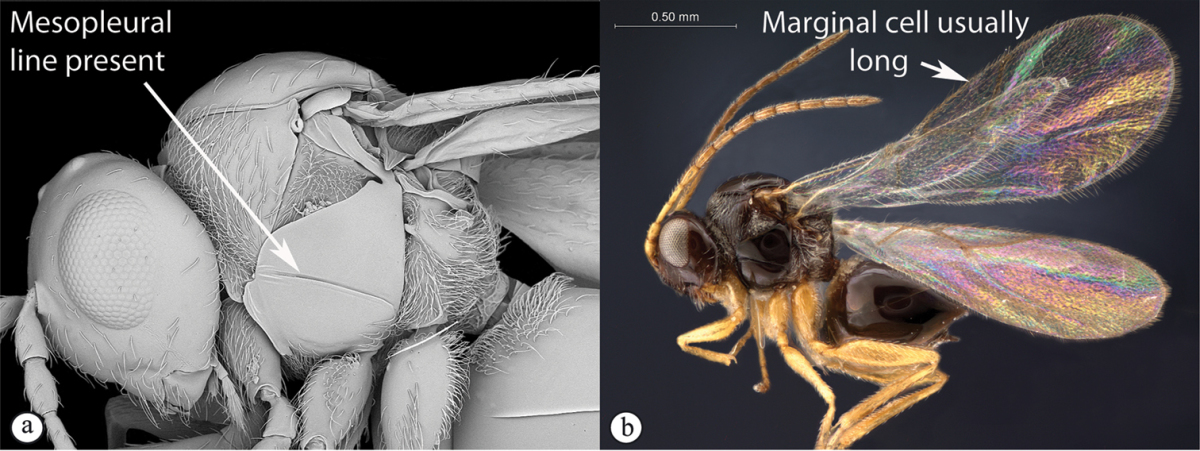	
3	With mesopleural line (a). Variable but usually more “figitid-looking”, darkly colored, with a long marginal cell (b)	***Phaenoglyphis***
	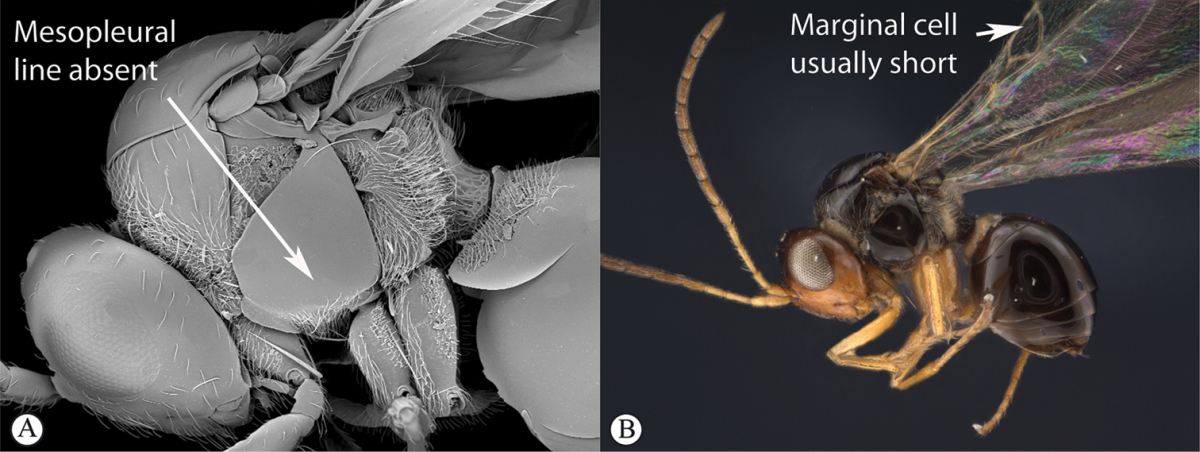	
–	Without mesopleural line (A). Usually tiny, pale, with a short marginal cell (B)	***Alloxysta***

#### Alloxysta

Taxon classificationAnimaliaHymenopteraFigitidae

Förster, 1869

##### Remarks.

Commonly collected. Reviewed for Africa by Ferrer-Suay et al. (2013).

##### Diagnosis.

These are the characteristic tiny pale charipines, but in fact they vary a lot in size and colour (also within species!). Colours vary from very dark brown through middle browns and reds to pale yellow, frequently with the head in a paler hue than the rest of the body. The absence of a mesopleural line is the best way to separate them from the otherwise often similar *Phaenoglyphis*. *Dilyta* and *Apocharips* also lack the mesopleural line, but can be separated by their conjoined two apical antennal articles, or by their characteristic metasoma, which is mostly covered by a syntergite and is oval in shape. The metasoma of a representative of *Alloxysta* typically shows the posterior tergites separate, and is truncated at the end, often with a (cynipid-like) oblique slash. Furthermore, most *Alloxysta* are larger, paler and more pubescent (on the pronotum, metapleura and coxae) than most *Dilyta*, and the mesoscutum is smoothly convex (rather than the hump of *Dilyta*).

**Figure 13.**
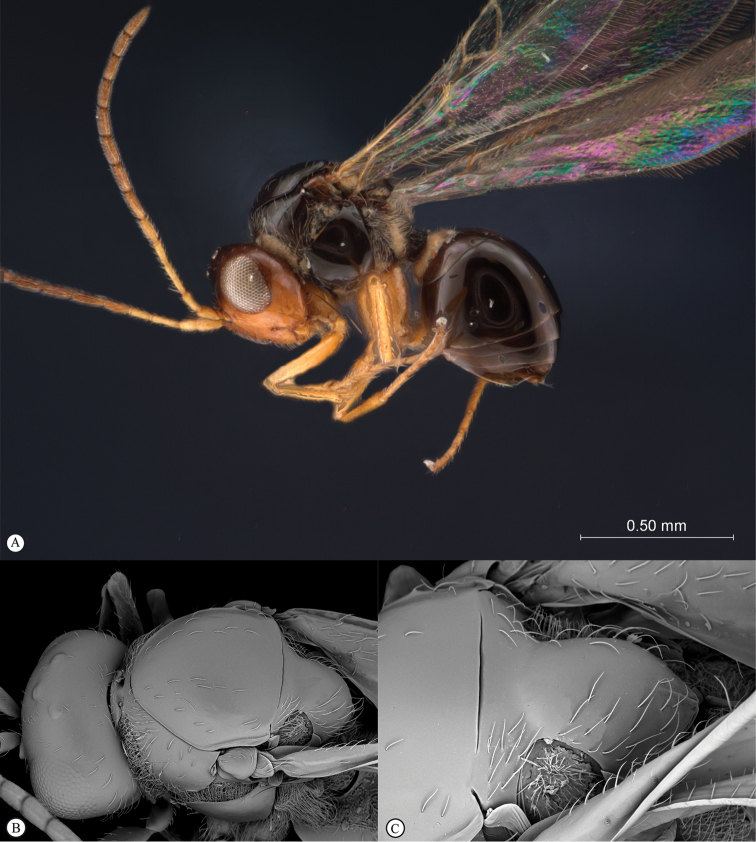
*Alloxysta* species (Kenya). **A** habitus lateral view **B** head and mesosoma dorsal view **C** scutellum, dorsal view.

##### Distribution.

Worldwide, but most abundant in the Holarctic region. Afrotropical records: Democratic Republic of Congo (Benoit 1956e), Kenya (Evenhuis 1974), Madagascar (Ferrer-Suay et al. 2012), Burundi, Rwanda, South Africa, Uganda, Zimbabwe (Ferrer-Suay and Pujade-Villar 2013) Ethiopia, Ghana, Namibia, Yemen, Zambia (here). Apparently some species are synanthropically widespread, but there is no reason to assume that none of the forms present in Africa are native.

##### Biology.

Hyperparasitoids attacking aphelinid and aphidiine wasps on aphids. (Gutierrez and van den Bosch 1970, Evenhuis passim, Andrews 1978, Fergusson 1986).

##### Species richness.

*Alloxysta antananarivoi* Ferrer-Suay & Pujade-Villar, 2012 (Burundi, Kenya, Madagascar, Rwanda, Zimbabwe)

*Alloxysta antsirananae* Ferrer-Suay & Pujade-Villar, 2012 (Madagascar, Zimbabwe)

*Alloxysta arcuata* (Kieffer, 1902) (*Allotria*) (Kenya, South Africa, Zimbabwe; also Palearctic and Neotropical regions)

*Alloxysta brevis* (Thomson, 1862) (*Allotria*) (Zimbabwe; also Palearctic and Neotropical regions)

*Alloxysta citripes* (Thomson, 1862) (*Allotria*) (South Africa; cosmopolitan species described from the Palearctic)

*Alloxysta fuscicornis* (Hartig, 1841) (*Xystus*) (Kenya, South Africa; cosmopolitan species described from the Palearctic)

*Alloxysta hendrickxi* (Benoit, 1956e) (*Charips*) (Democratic Republic of Congo, Kenya, Zimbabwe)

*Alloxysta mullensis* (Cameron, 1883) (*Allotria*) (Kenya, Madagascar, South Africa, Uganda, Zimbabwe; also Palearctic and Neotropical regions)

*Alloxysta pilipennis* (Hartig, 1840) (*Xystus*) (Zimbabwe; also Palearctic and Neotropical regions)

#### Apocharips

Taxon classificationAnimaliaHymenopteraFigitidae

Fergusson, 1986

##### Remarks.

Rare.

##### Diagnosis.

Similar to *Dilyta*, and most easily separated by wing ventation characters: an elongate marginal cell, a subcosta running near mid-width of wing in basal part and having a distinct curve near the basal cross-vein. Other distinguishing characters, easily observed in larger specimens but hardly seen in smaller, is the scutellum with distinct lateral and posterior carinae, and the syntergite where T2 is barely visible as a separate small anterior sclerite.

**Figure 14.**
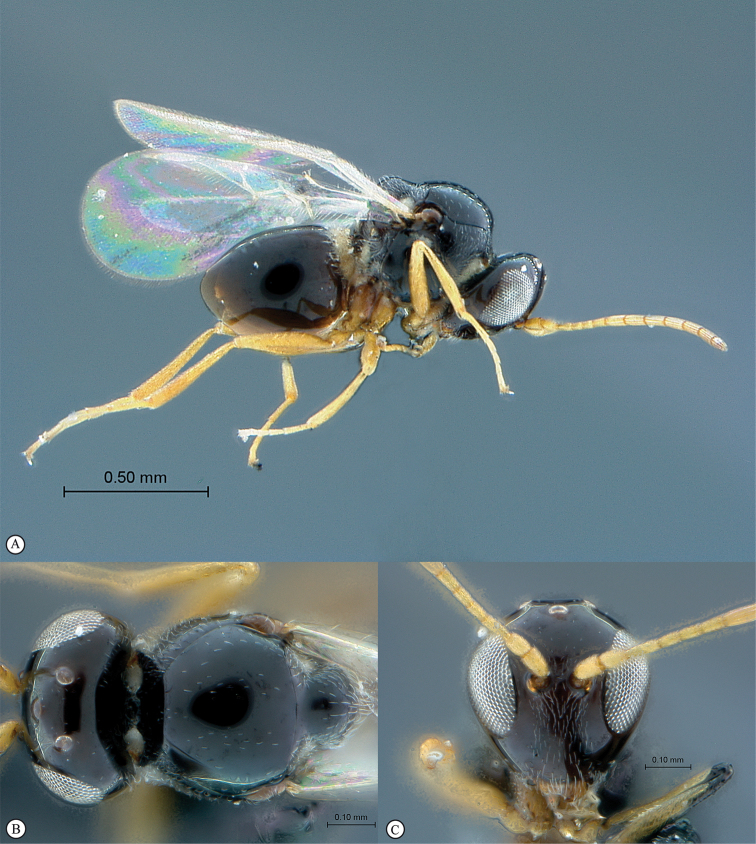
*Apocharips trapezoidea* (Ghana). **A** habitus lateral view **B** head and mesosoma dorsal view **C** head, anterior view.

##### Distribution.

Palearctic, Neotropical and Afrotropical. Afrotropical records: Ethiopia (Silvestri 1915), Kenya (Ferrer-Suay and Pujade-Villar 2013), Ghana, Uganda, Zimbabwe (here).

##### Biology.

Hyperparasitoids attacking wasps on psyllids (Vasileva-Sumnalieva 1976, Menke 1993). A specimen from Uganda in BMNH was reared from the psyllid *Pseudoeriopsylla laingi* Hollis & Broomfield, 1989 on *Ficus natalensis* Hochst. (label data).

##### Species richness.

*Apocharips trapezoidea* (Hartig, 1841) (*Xystus*) (Ethiopia, Ghana, Kenya; Palearctic species)

syn *Alloxista peraptera* Silvestri, 1915. Synonymy implied in Ferrer-Suay et al. (2013), but apparently never published as an explicit new synonymy

#### Dilyta

Taxon classificationAnimaliaHymenopteraFigitidae

Förster, 1869

##### Remarks.

Common. Revised for the region by Paretas-Martinez et al. (2009).

##### Diagnosis.

Mostly tiny and dark brown charipines. Separated from *Apocharips* by lacking lateral ridges on scutellum, having at most a small posterior ridge, and by usually having T2 discernable as a separate sclerite. May be confused with *Alloxysta*, but has an oval-rounded metasoma covered in the major part by a syntergite (no posterior tergites visible) and has the two apical articles of the female antenna immovable conjoined. Furthermore, *Alloxysta* are usually larger, paler, has more pubescence, and have a more smoothly convex mesoscutum; *Dilyta* have a characteristic anterior hump in lateral view.

**Figure 15.**
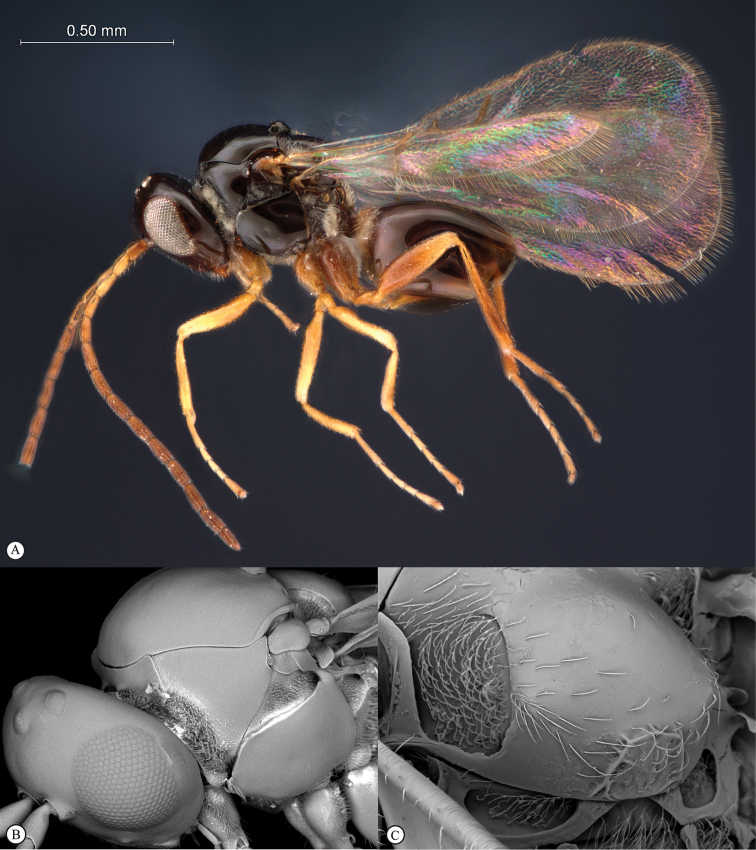
*Dilyta* species (Madagascar). **A** habitus lateral view **B** head and mesosoma dorso-lateral view **C** scutellum, posterio-dorsal view.

##### Distribution.

Holarctic and Old World Tropics. Afrotropical records: Cameroon (Lebel and Modesta 2007), Democratic Republic of Congo (Benoit 1956e), Botswana, Ghana, Kenya, Somalia, South Africa (Paretas-Martinez et al. 2009), Madagascar (Pujade-Villar and Ferrer-Suay 2012), Rwanda, Zimbabwe (Ferrer-Suay et al. 2013), Central African Republic, Nigeria, Tanzania, Uganda, Yemen (here).

##### Biology.

Hyperparasitoids attacking encyrtid wasps on psyllids (Menke and Evenhuis 1991).

##### Species richness.

*Dilyta africana* (Benoit, 1956e) (*Alloxysta*) (Democratic Republic of Congo, Rwanda, Tanzania, Zimbabwe)

*Dilyta australafricana* Paretas-Martínez & Pujade-Villar, 2009 (South Africa)

*Dilyta camerounensis* Lebel & Modeste, 2007 (Cameroon)

*Dilyta ghanana* Paretas-Martínez, Pujade-Villar & Melika, 2009 (Central African Republic, Ghana)

*Dilyta kenyana* Paretas-Martínez & Pujade-Villar, 2009 (Kenya)

*Dilyta paretasmartinezi* Pujade-Villar & Ferrer-Suay, 2012 (Madagascar)

*Dilyta somaliana* Paretas-Martínez, Pujade-Villar & Evenhuis, 2009 (Botswana, Somalia, South Africa)

*Dilyta subclavata* Förster, 1869 (Madagascar; a European species)

#### Phaenoglyphis

Taxon classificationAnimaliaHymenopteraFigitidae

Förster, 1869

##### Remarks.

Not uncommon in South Africa.

##### Diagnosis.

Similar to *Alloxysta*, but usually with a habitus more resembling other figitids. Easily recognised among charipines through the possession of a mesopleural carina.

**Figure 16.**
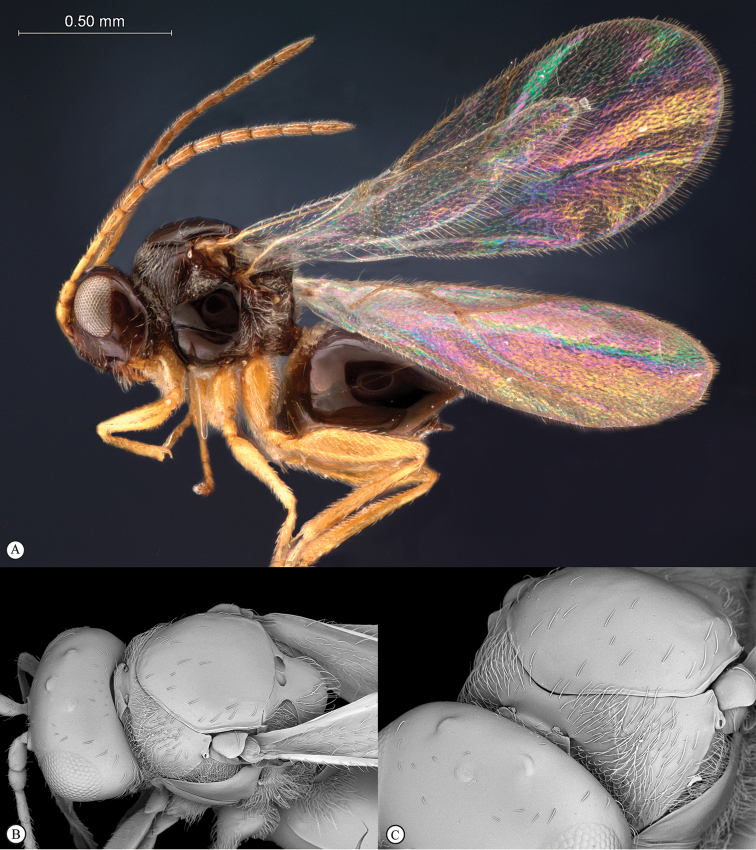
*Phaenoglyphis* species (South Africa). **A** habitus lateral view **B** head and mesosoma dorso-lateral view **C** pronotum, dorso-anterior view.

##### Distribution.

Worldwide, but most abundant in Holarctic. Afrotropical records: South Africa (Gaston et al. 2003). Seemingly introduced.

##### Biology.

Hyperparasitoids attacking aphelinid and aphidiine wasps on aphids (Kierych 1979; Quinlan and Evenhuis 1980; Fergusson 1986).

##### Species richness.

*Phaenoglyphis villosa* (Hartig, 1841) (*Xystus*) (South Africa; this species is widespread throughout the world (Pujade-Villar et al. 2007)

### Emargininae

The Emargininae are represented in the Afrotropical region by a single genus containing 12 described species. A number of undescribed species are present in world collections.

**Biology.** Unknown (Buffington et al. 2012), possibly associated with ants (Weld 1960, Díaz 1978).

**Distribution.** The subfamily is represented in all biogeographical regions except the Antarctic, but with very few species in the Holarctic and the majority of species occurring in the Afrotropical region (present study).

#### Thoreauella

Taxon classificationAnimaliaHymenopteraFigitidae

Girault, 1930

Thoreauella (synonyms: *Bothrocynips* Díaz, 1978, **syn. n.**, *Emargo* Weld, 1960, **syn. n.**, *Weldiola* Kovalev, 1994, **syn. n.**, *Quinlania* Kovalev, 1994, **syn. n.**)

##### Remarks.

*Emargo* and *Thoreauella* (and *Bothriocynips*) were described at different times in different groups that are now considered different figitid subfamilies, obviously with the authors being unaware of the other generic names (Díaz 1978, Girault 1930, Quinlan 1960). The Neotropical *Bothriocynips* was made a junior synonym of the Neotropical and Afrotropical *Emargo* by Pujade-Villar et al (2002). However nothing has been suggested to distinguish *Emargo* from *Thoreauella* except distribution (*Emargo* Neotropical and Afrotropical, *Thoreauella* Australian), and there is no morphological evidence to support keeping them separate. Kovalev (1994), within a consideration of broad evolutionary trends in the Cynipoidea, found that some of the characters separating some of the Afrotropical *Emargo* species in Quinlan’s key (Quinlan 1988) were important enough to merit separation on a higher taxonomic level and erected not just new genera but new tribes for two of Quinlan’s species from Madagascar (and a new family for the whole group). Ronquist (1995) changed the status of Kovalev’s Emarginidae into a subfamily, and made Kovalev’s Weldiolini and Quinlaniini synonyms of it, but Kovalev’s genera have remained to this date, in spite of being raised in the absence of an actual morphological study as well as of consideration of the global variation. Here we consider that at the current level of knowledge they constitute mere recognisable species or species groups among others within a single genus.

Thus, here we synonymise all genera of Emargininae, and the senior name is *Thoreauella* Girault. Most of the resulting new combinations are species hitherto classified as *Emargo* Weld, including the Neotropical type species *Thoreauella eciton* (Weld, 1960), **comb. n.** *Bothriocynips* was already made a synonym of *Emargo* but is a new synonym of *Thoreauella*, and its Neotropical type species is now *Thoreauella recisa* (Díaz, 1978), **comb. n.** *Quinlaniana* and *Weldiola* were both monotypical for taxa from Madagascar and cited in the new combinations *Thoreauella pexa* and *Thoreauella capito* respectively below.

##### Diagnosis.

Members of the subfamily Emargininae, now coinciding with the genus *Thoreauella*, uniformly possess an emarginate apical margin of the forewing. The only other cynipoids to have such a character are species of *Kleidotoma*, but being eucoilines, the latter have a distinct scutellar plate with posterior midpit. Some emarginines have what appears to be a raised scutellar plate on the scutellum, but in these species, there is clearly an entire lack of a glandular pit. Emarginines also typically have: a very abrupt, compact marginal cell in the forewing; distinct notauli; dense ‘foamy’ setae on the propodeum and base of the metasoma; and large, setiferous pits on the flagellomeres of the male antenna.

Much species level work remains to be conducted on the Afrotropical emarginines. However, we have noted the following characteristics of currently unnamed species groups:

Species group A, which appears to be common in Madagascar, comprises species that possess notauli, and have a thin, complete, lamella along the posterior margin of the scutellum. The lamellae enclose a rather deep ‘trough’; further, the lamella is so thin that without significant magnification, it will not be visible. The presence of this resulting ‘trough’ may have led Quinlan (1988) to erroneously conclude Emargininae are actually Eucoilinae.

Species group B also possesses notauli, as well as having rather spectacular projections off the posterior margin of the scutellum. Two sub-groups can be recognized; one with a simply bifurcate scutellar margin; and a second group possessing a single projection off the posterior margin of the scutellum. Both subgroups contain the largest physical specimens of *Thoreauella*, with some reaching nearly 1.5 mm; further, nearly all members of this group have dark, dusky wings. Quinlan’s *Thoreauella pexa*, considered by Kovalev to constitute the tribe Quinlanianini, belongs in this species group. Species group B appears to be endemic to Madagascar.

Species group C possesses notauli, and is similar in overall appearance to species group A, however, species in group C lack the posterior lamella on the scutellum. Nevertheless, group C species do have a deep, marked depression on the posterior margin of the scutellum. Quinlan's *Thoreauella capito*, considered by Kovalev to constitute the tribe Weldiolini, belongs in this species group. Species group C is common in continental Africa (throughout the tropical belt), as well as Madagascar.

Species group D is the most widespread of the four genus groups, found equally common in Madagascar and continental Africa. The distinguishing feature of this group is the general lack of clear notauli. In some species, it is clear that the mesoscutum is perfectly smooth; in others, there are faint traces of notauli, but never as clearly indicated as in species groups A–C. The posterior margin of the scutellum in these species is rounded, lacking any remarkable morphology. *Thoreauella laverna*, *Thoreauella micipsa*, *Thoreauella palloris*, and *Thoreauella vacuna* would be included in this group.

**Figure 17.**
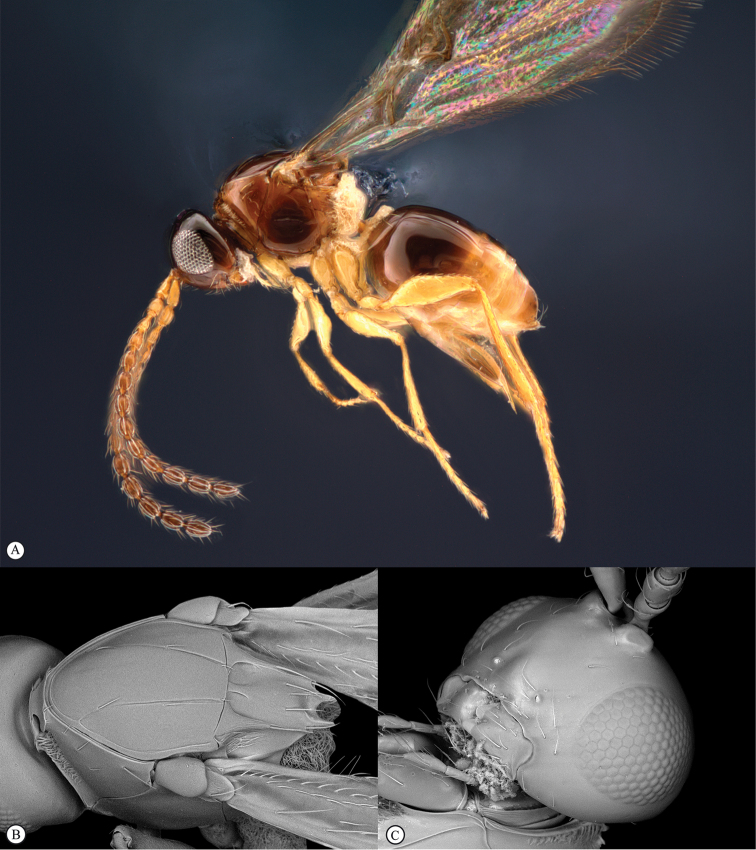
*Thoreauella* species (Madagascar). **A** habitus lateral view **B** head and mesosoma dorsal view **C** head, anterior view.

##### Distribution.

Pantropical, apparently especially diverse in Madagascar. Presence in the Oriental region has not been published hitherto but is confirmed here. Afrotropical records: Cameroon, Democratic Republic of Congo, Madagascar, South Africa, Zambia, Zimbabwe (Quinlan 1988), Central African Republic, Ghana, Nigeria, Rwanda, Tanzania, Uganda (here).

##### Biology.

Hosts unknown. Appear to be associated with ants (Weld 1960, Díaz 1978).

##### Species richness.

Species group A

*Thoreauella numa* (Quinlan, 1988), **comb. n.** (*Emargo*) (Zimbabwe)

Species group B

*Thoreauella pexa* (Quinlan, 1988), **comb. n.** (*Emargo*) (Madagascar)

Species group C

*Thoreauella ascia* (Quinlan, 1988), **comb. n.** (*Emargo*) (Madagascar, Zambia)

*Thoreauella cantus* (Quinlan, 1988), **comb. n.** (*Emargo*) (Democratic Republic of Congo, Zimbabwe)

*Thoreauella capito* (Quinlan, 1988), **comb. n.** (*Emargo*) (Madagascar)

*Thoreauella matius* (Quinlan, 1988), **comb. n.** (*Emargo*) (Cameroon, Democratic Republic of Congo, Kenya, Zimbabwe)

*Thoreauella peleus* (Quinlan, 1988), **comb. n.** (*Emargo*) (Cameroon, Madagascar)

*Thoreauella themis* (Quinlan, 1988), **comb. n.** (*Emargo*) (Cameroon, Madagascar)

Species group D

*Thoreauella laverna* (Quinlan, 1988), **comb. n.** (*Emargo*) (Kenya, Zimbabwe)

*Thoreauella micipsa* (Quinlan, 1988), **comb. n.** (*Emargo*) (Cameroon, Democratic Republic of Congo, Madagascar)

*Thoreauella palloris* (Quinlan, 1988), **comb. n.** (*Emargo*) (Democratic Republic of Congo)

*Thoreauella vacuna* (Quinlan, 1988), **comb. n.** (*Emargo*) (South Africa)

### Eucoilinae

The Eucolinae are represented in the Afrotropical region by 30 genera containing 176 described species. Numerous undescribed species are present in world collections awaiting description.

**Biology.** Afrotropical eucoilines are koinobiont endoparasitoids of Muscomorpha Diptera larvae (Buffington et al. 2012). A large group of Eucoilinae are parasitoids of Agromyzidae, mostly leafminers in the canopy, but the majority attack various families of flies typically in decomposing habitats (carrion, dung, fruit, leaf litter, sea wrack etc) but also in living plants, mushrooms and algae (Drosophilidae, Phoridae, Sepsidae, Ephydridae, Muscidae, Calliphoridae etc). However, it must be noted that the biology of the majority of eucoiline species remains unknown (Forshage and Buffington pers. obs.).

**Distribution.** The subfamily is represented in all biogeographical regions (including subantarctic islands). The majority of described species are Palearctic, and indeed there is a striking diversity in some genera throughout the Holarctic, but species diversity in the tropical regions is far richer though mostly yet undescribed, and possibly the Neotropics has the largest diversity of all regions (Buffington and Forshage pers. obs.).

#### Key to Afrotropical eucoiline tribes and genera

**Table:** 

	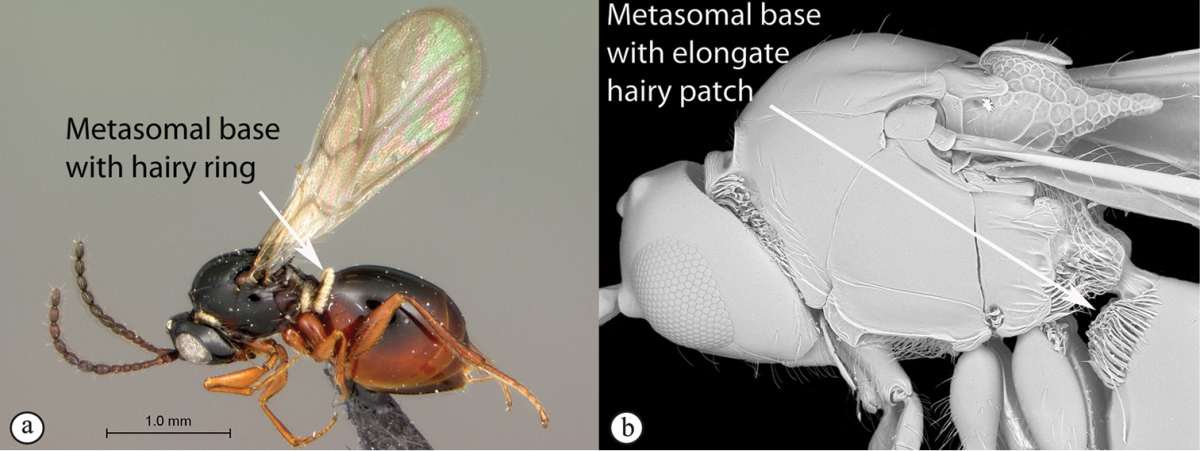	
1	Metasomal base with hairy ring (a) or elongate hair patches laterally (b)	**2**
	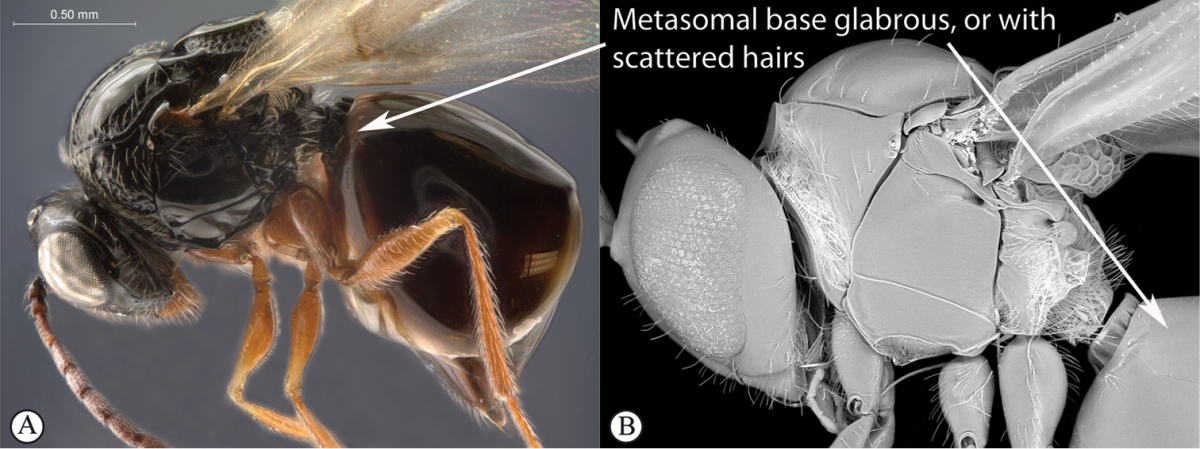	
–	Metasomal base glabrous (A) or with scattered hairs only (B)	**24**
	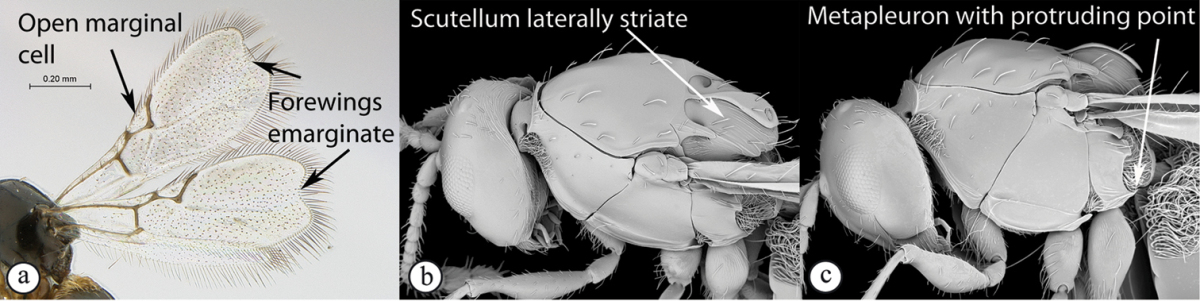	
2	Forewing emarginated (a); reduced wing venation with open marginal cell (a); usually with partial reduction of wing pubescence. Scutellum longitudinally striate with a narrow scutellar plate (b). Posteroventral corner of metapleuron pointedly protruding (c). Head bulbous with relatively small eyes (c)	***Kleidotoma* (Kleidotomini)**
	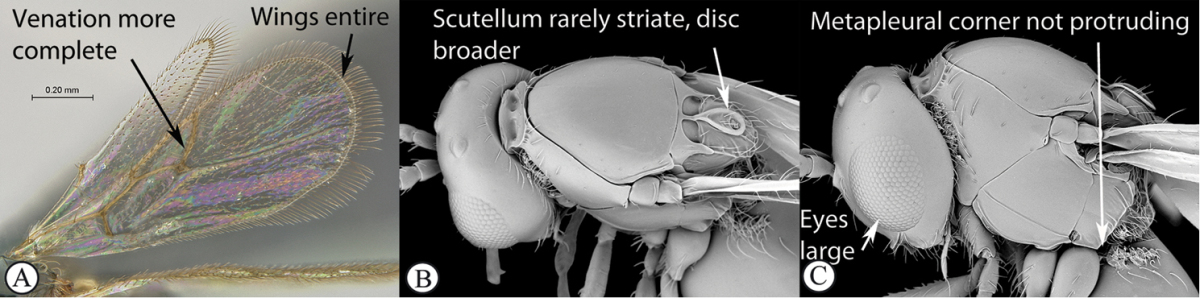	
–	Wings not distinctly emarginated (A); wing venation more complete, marginal cell open, partially closed, or completely closed (A). Scutellum variable, but very rarely with a striate scutellum and narrow scutellar disc (B). Metapleural corner not protruding (C). Head variable, but rarely bulbous with small eyes	**3**
	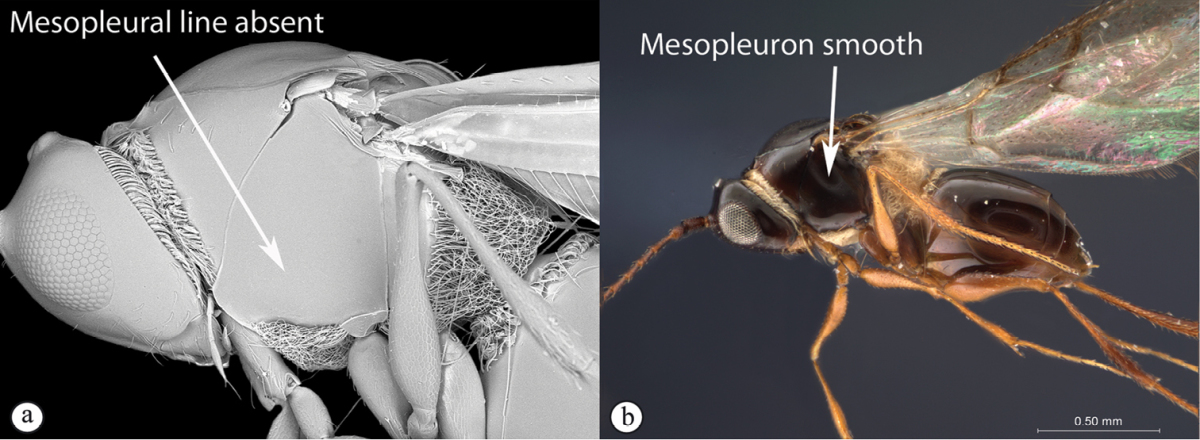	
3	Mesopleural line absent (a, b); mesopleuron entirely smooth (a, b)	**4**
	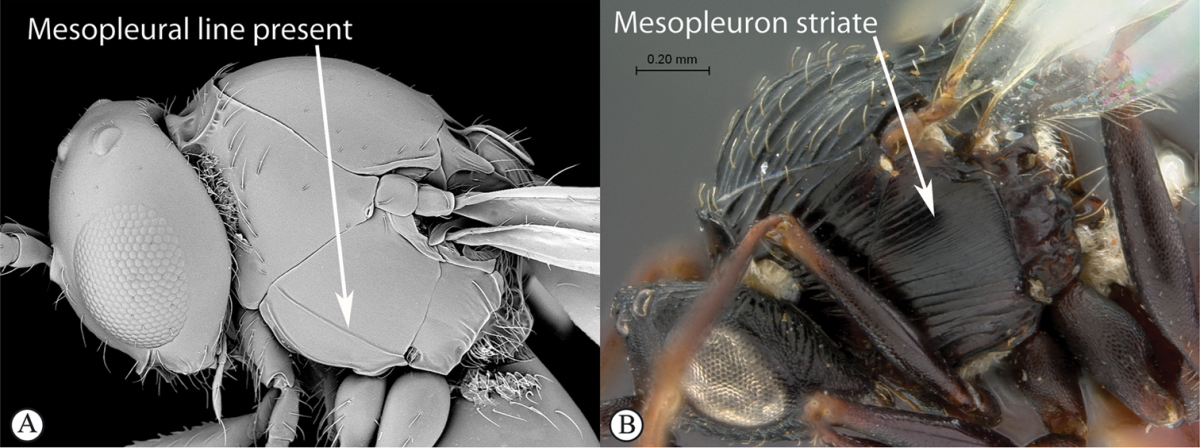	
–	Mesopleural line present (A), or mesopleuron heavily striate (B)	**6**
	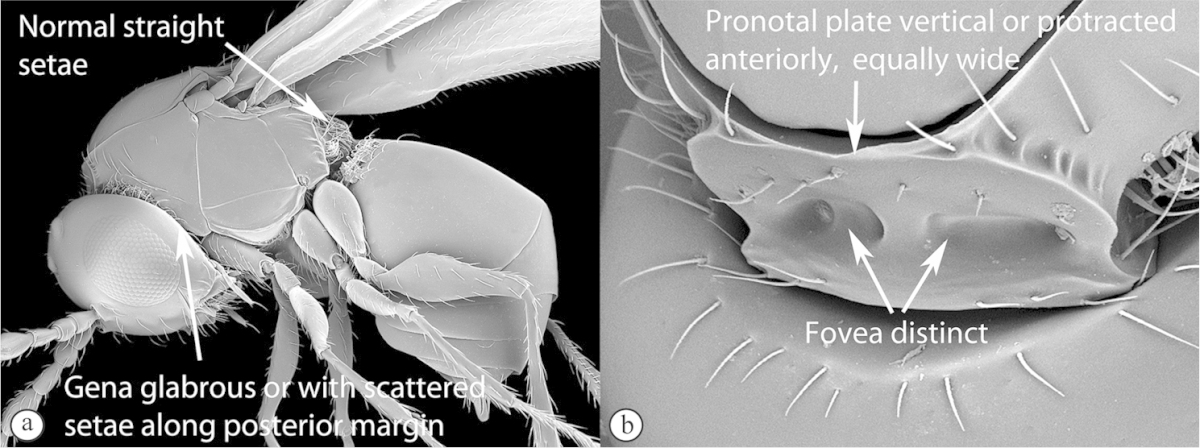	
4	Foamy setae absent on propodeum and metasomal base; normal straight setae present (a), or glabrous. Gena glabrous, or with a few scattered setae along posterior margin (a). Pronotal plate either vertical or protracted anteriorly, with anterior and posterior halves roughly the same width (b); fovea present, distinct (b)	**5**
	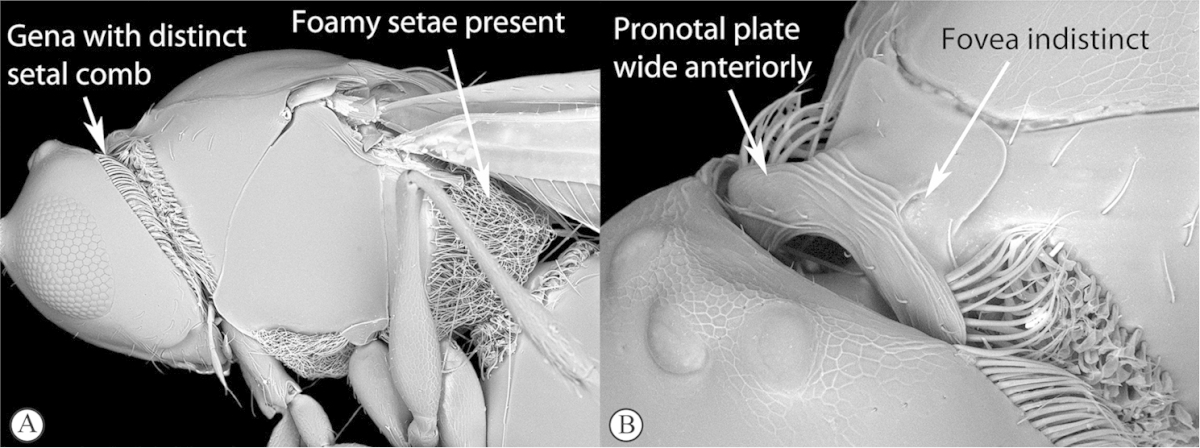	
–	‘Foamy’, reticulate setae present on propodeum and anterior base of metasoma (A). Distinct setal comb present along posterior margin of gena (A). Pronotal plate protracted anteriorly, anterior half distinctly wider than posterior half (B), lateral fovea shallow, nearly indistinct (B)	***Leptolamina*** (*Leptolamina* group, tribe uncertain)
	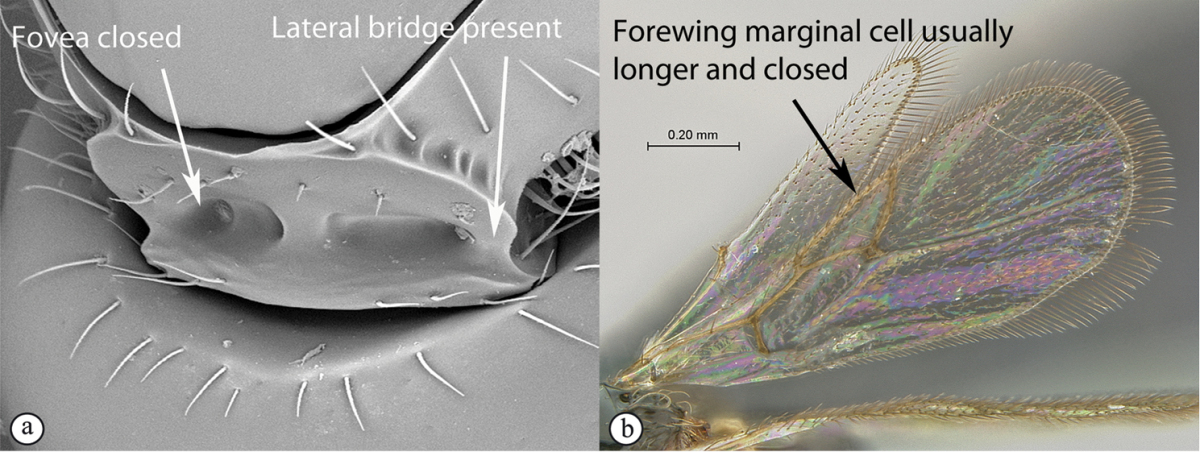	
5	Fovea on pronotal plate closed, lateral bridge present (a). Forewing marginal cell usually longer and closed (b)	***Rhoptromeris*** (in part) (Trichoplastini)
	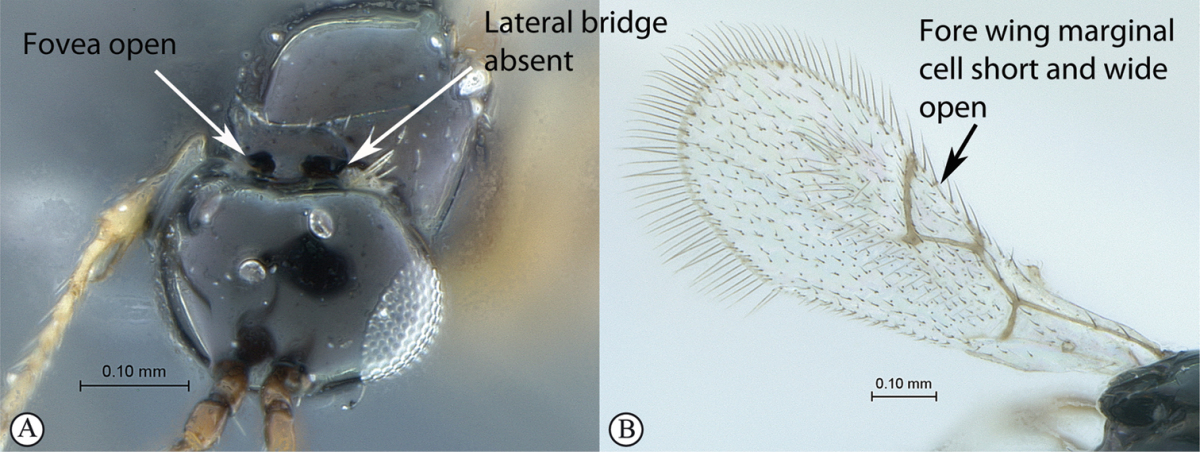	
–	Fovea on pronotal plate open, lateral bridge absent (A). Fore wing marginal cell short and wide open (B). Tiny wasps	***Micreriodes*** (*Leptolamina* group, tribe uncertain)
	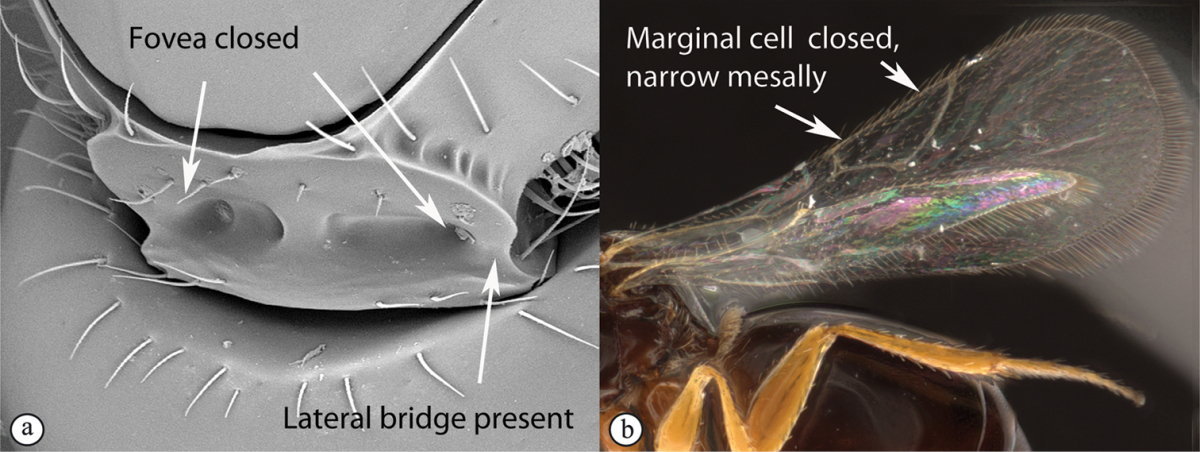	
6	Pronotal plate with closed lateral foveae, lateral bridges present (a). Forewings narrow (b); marginal cell closed, distinctly narrow mesally (b). Species typically more or less laterally compressed	**Trichoplastini 7**
	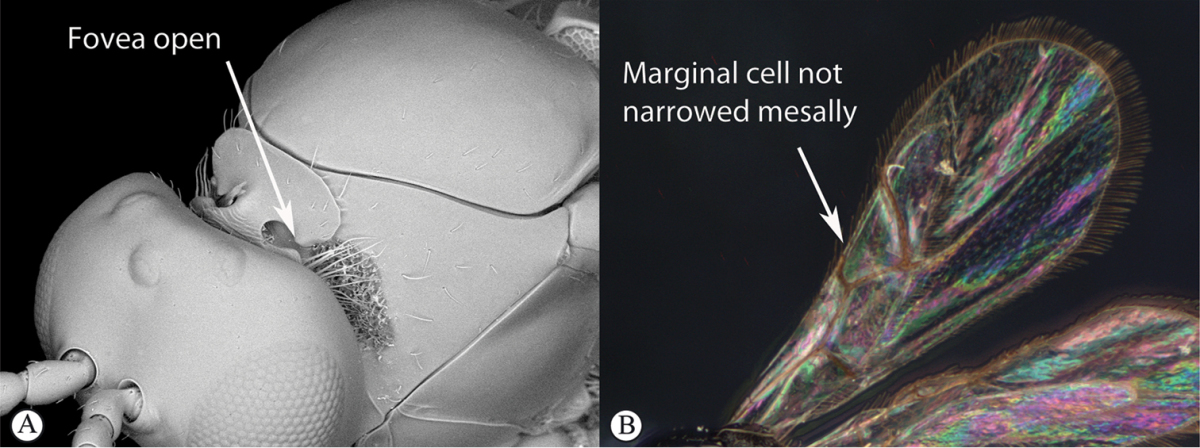	
–	Pronotal plate with lateral foveae open (A), or fovea too shallow to see. Forewings narrow to broad; marginal cell open, partially open, or closed; shape of marginal cell more symmetrical, not narrowed mesally (B)	**12**
	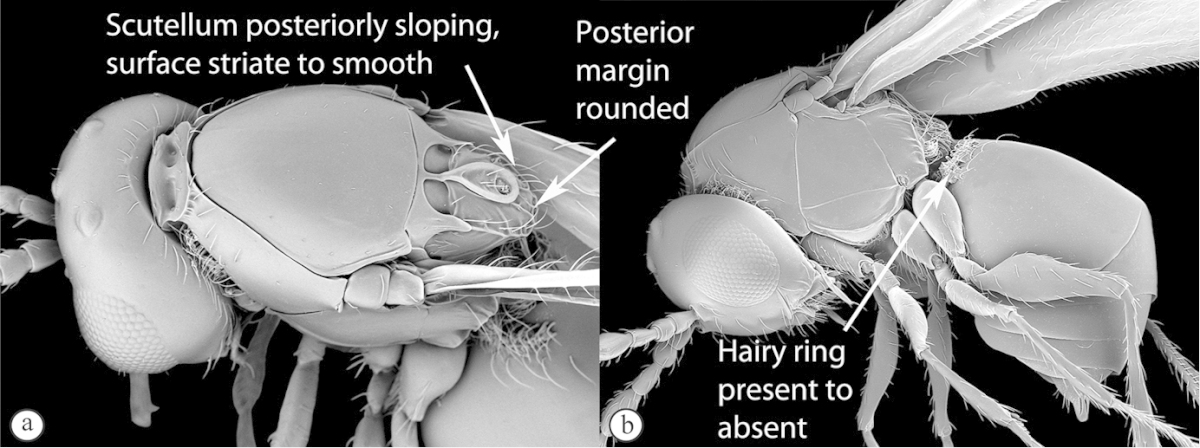	
7	Posterior part of scutellum strongly sloping, rarely overhanging propodeum (a); dorsal surface of scutellum distinctly longitudinally striate to smooth, occasionally foveate (a); posterior margin of scutellum rounded, not drawn out into a spine (a). Posterior margin of metapleuron occasionally with a distinct cavity. Hairy ring of metasoma ranging from entire to absent (b)	**8**
	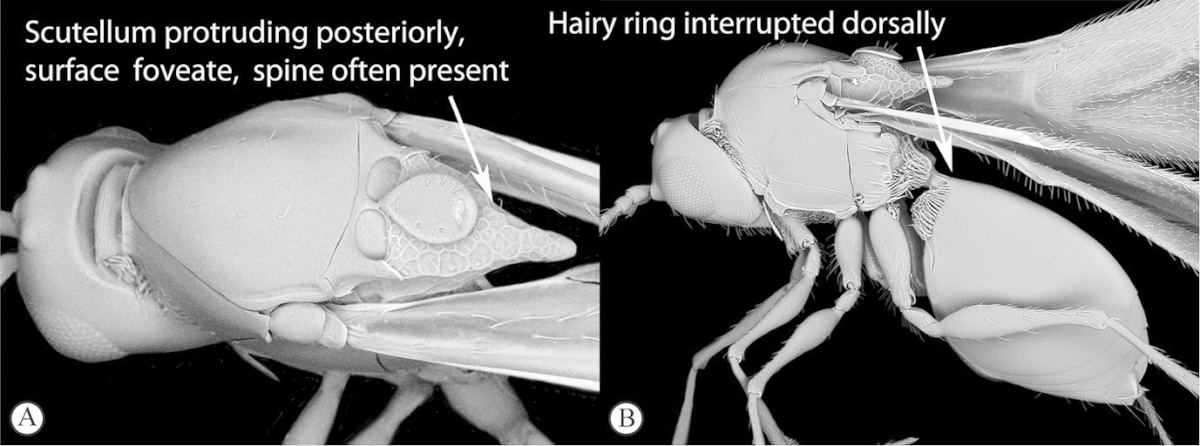	
–	Posterior part of scutellum protruding posteriorly, overhanging propodeum (A); dorsal surface of scutellum foveate (A), never striate or smooth; posterior margin of scutellum often drawn out to a distinct spine (A, B). Posterior margin of metapleuron always entire. Hairy ring at base of metasoma usually broadly interrupted dorsally (B)	**10**
	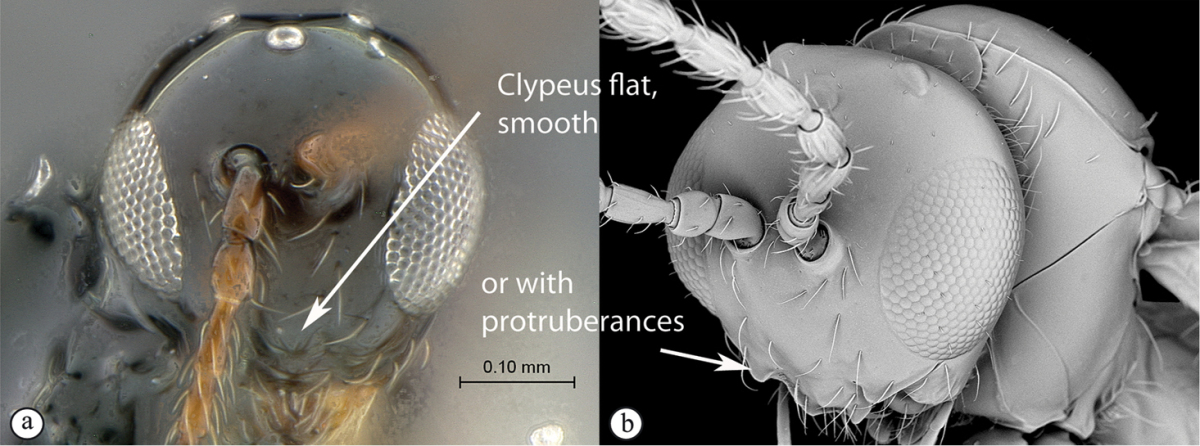	
8	Clypeus typically flat and smooth (a); rarely bifurcate with single small medial conical protuberance (b). Mandibles of normal cynipoid appearance, triangular, not enlarged. All sizes, usually small or medium sized. Very common	**9**
	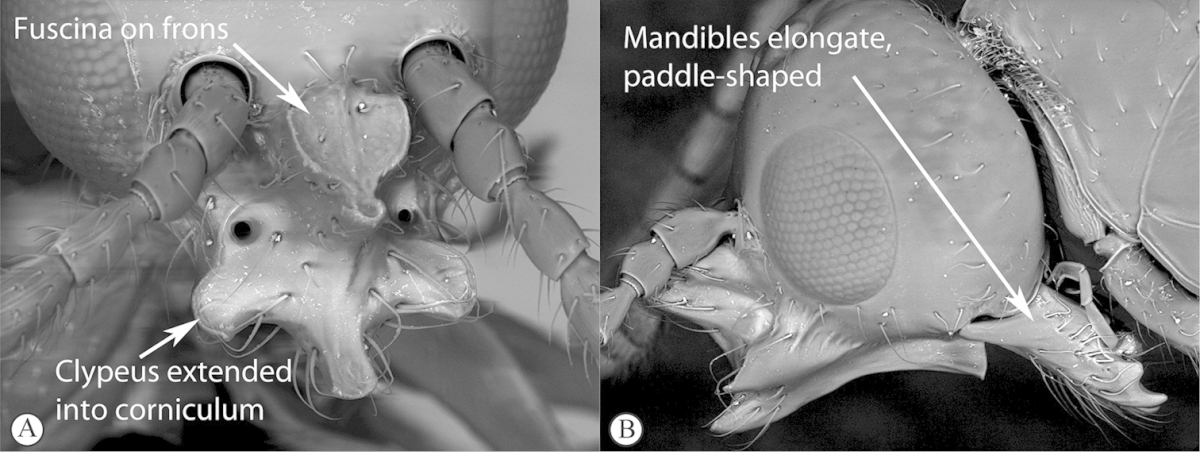	
–	Clypeus extended into a massive corniculum, with a fuscina on frons (A); mandibles elongate, paddle-shaped (B). Very rare	***Nanocthulhu***
	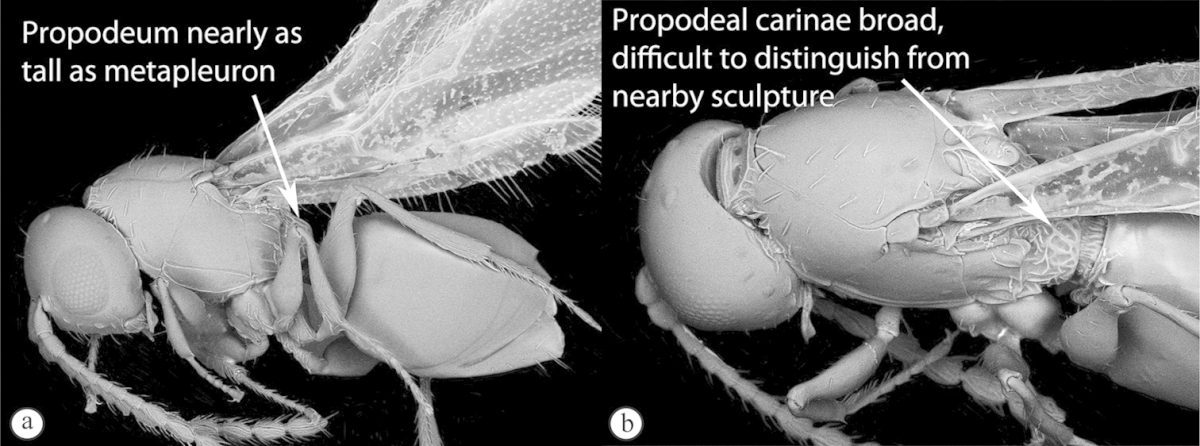	
9	Propodeum, in lateral view, nearly as tall as metapleuron (a), nearly parallel-sided, hardly tapering posteriorly; propodeal carinae broad, difficult to distinguish from nearby sculpture (b), gently divergent; rare	***Garudella*** (tribe uncertain)
	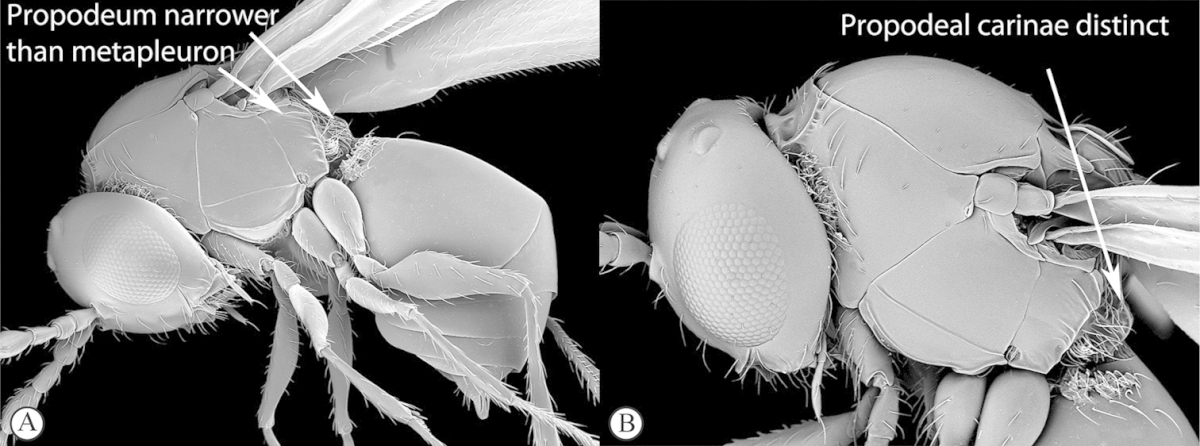	
–	Propodeum, in lateral view, much narrower than metapleuron (A), distinctly tapering posteriorly; propodeal carinae very distinct from nearby sculpture (B), nearly parallel; very common	***Rhoptromeris***
	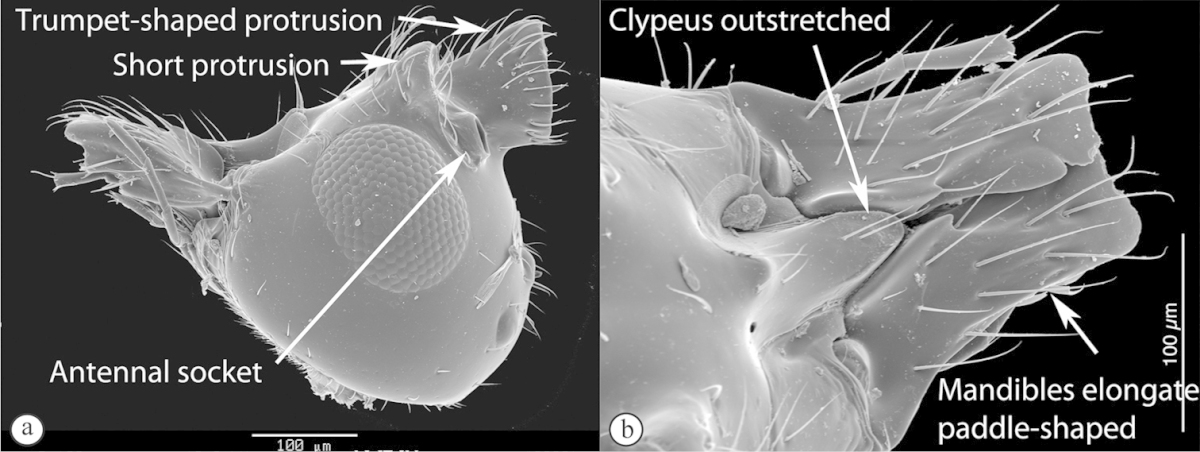	
10	Face with trumpet-shaped anterior-ventral protrusion between toruli (a); short paired anterior protrusions present under antennal sockets (a). Clypeus ventrally outstretched (b); mandibles elongate, paddle-shaped (b)	***Stentorceps***
	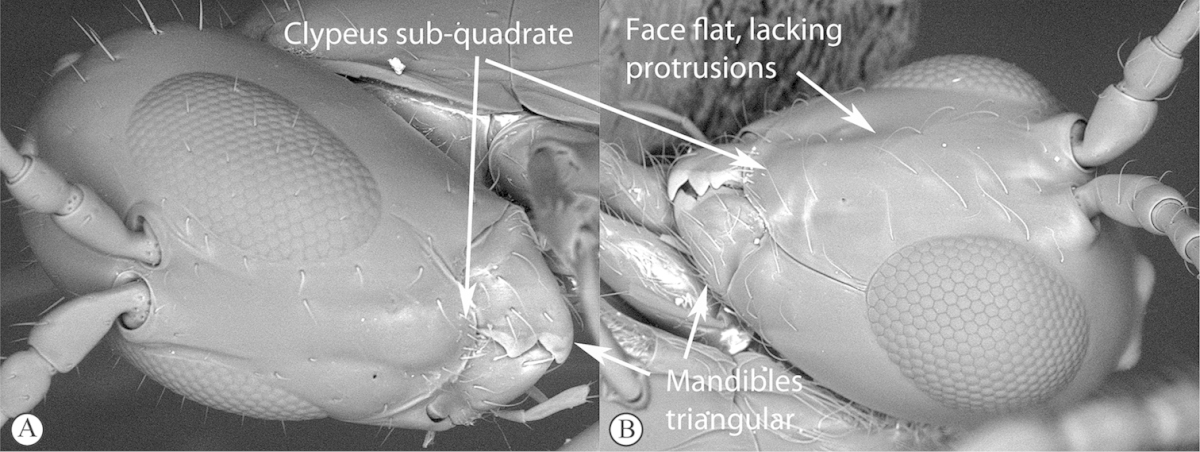	
–	Face flat, lacking protrusions (A, B); protrusions from ventral margin of toruli absent (B). Clypeus sub-quadrate, not outstretched (A, B); mandibles triangular (A, B)	**11**
	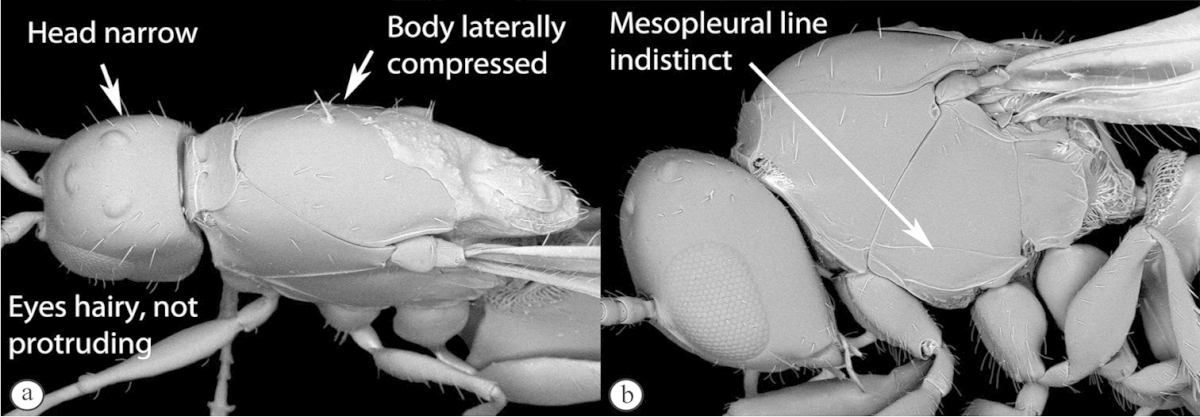	
11	Entire wasp laterally compressed, narrow, with a protruding head (a); head more than twice as long as wide, eyes hairy and scarcely protruding from outline of head capsule (a). Mesopleural line indistinct and very low on metapleuron (b)	***Angustacorpa***
	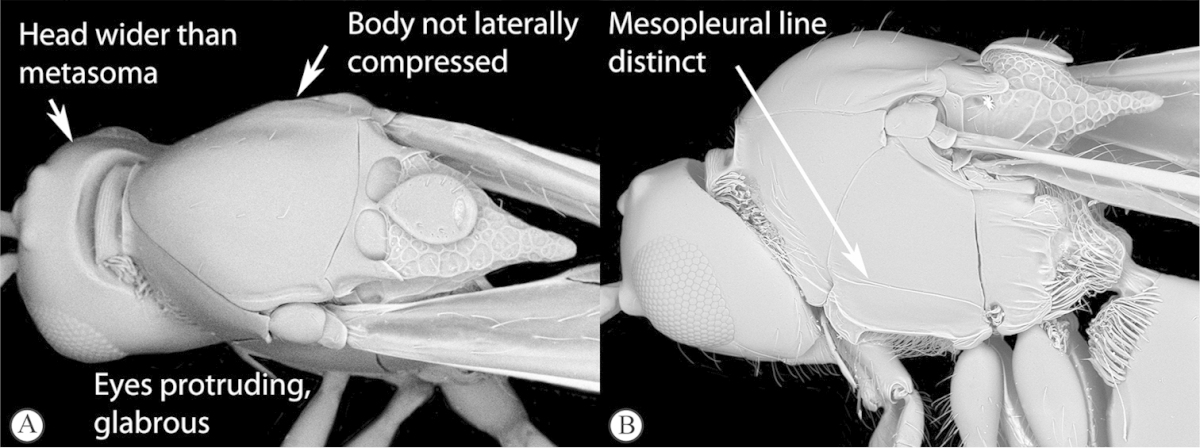	
–	Normally shaped, or rarely laterally compressed, head normally transversal. Head about as long as wide, occasionally slightly longer than wide (A). Eyes always protruding from outline of head capsule, head always wider than metasoma, eyes glabrous (A). Mesopleural line distinct (B)	***Trichoplasta***
	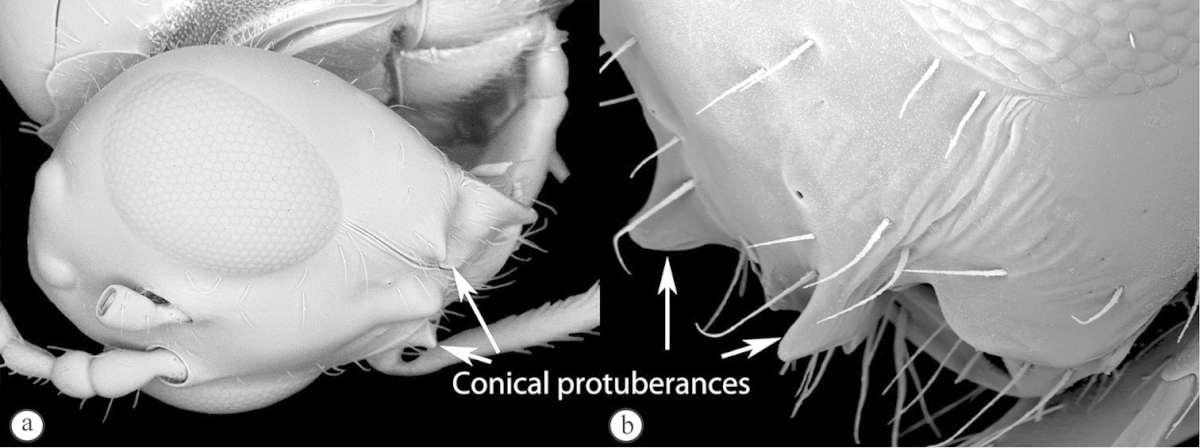	
12	Conical protuberances on clypeus and malar space (a, b)	**13**
	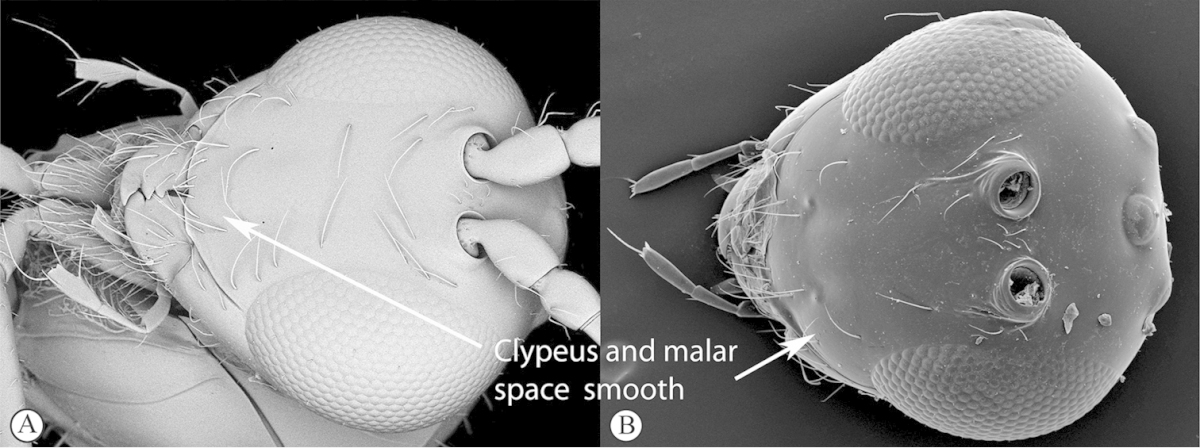	
–	Protuberances on clypeus and malar space absent (A, B)	**14**
	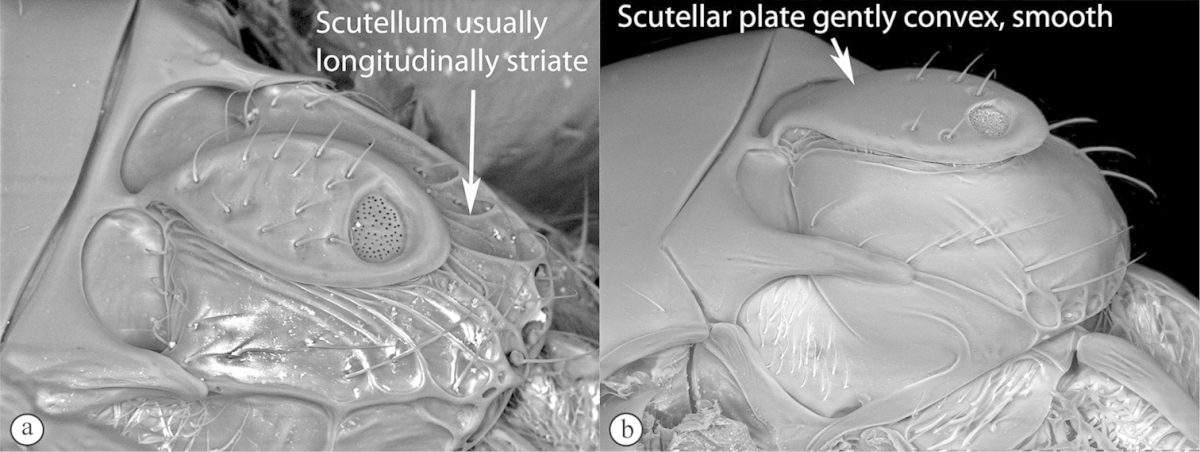	
13	Dorsal surface of the scutellum usually longitudinally striate (a). Scutellar plate gently convex, smooth (b). Commonly collected	***Hexacola*** (in part) **(Ganaspini)**
	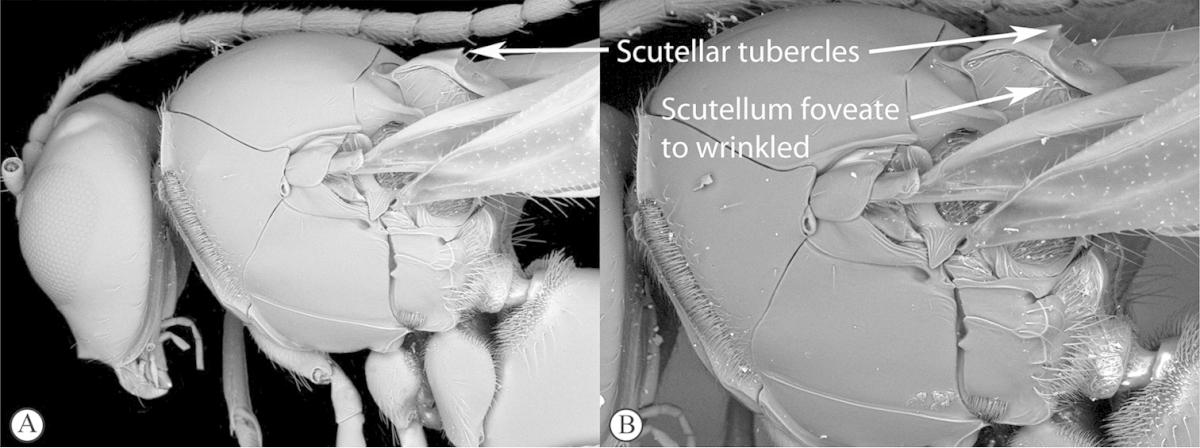	
–	Dorsal surface of scutellum foveate to wrinkled, not striate (B). Scutellar plate flat with a pair of tubercles present just anterior to the glandular pit (A, B). Rare	***Ganaspidium* (Diglyphosematini)**
	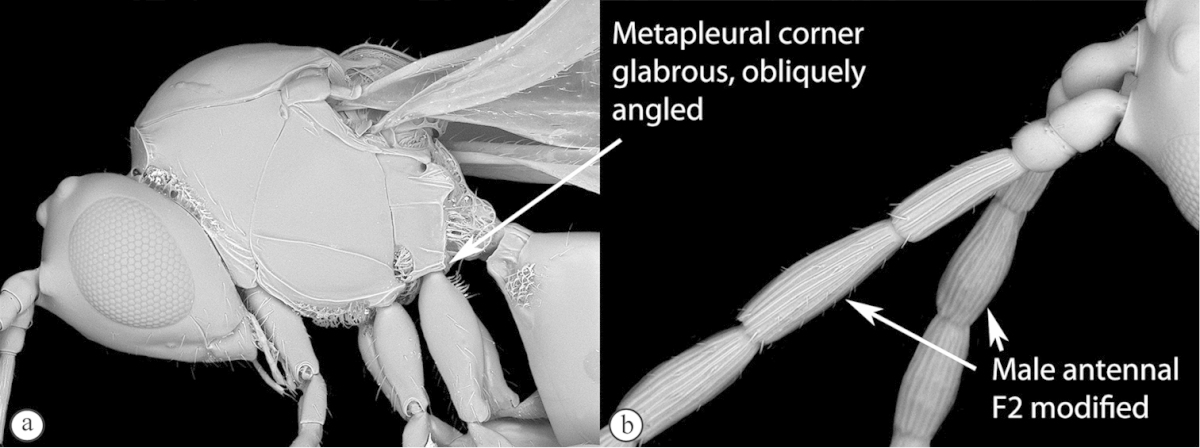	
14	Posteroventral corner of metapleuron glabrous, obliquely angled, corner often raised and forming a more or less triangular surface facing posterolaterally (a). Antennal F2 modified in males, more or less asymmetric, more so than F1 (or rarely only as much as F1) (b)	**15 (Eucoilini)**
	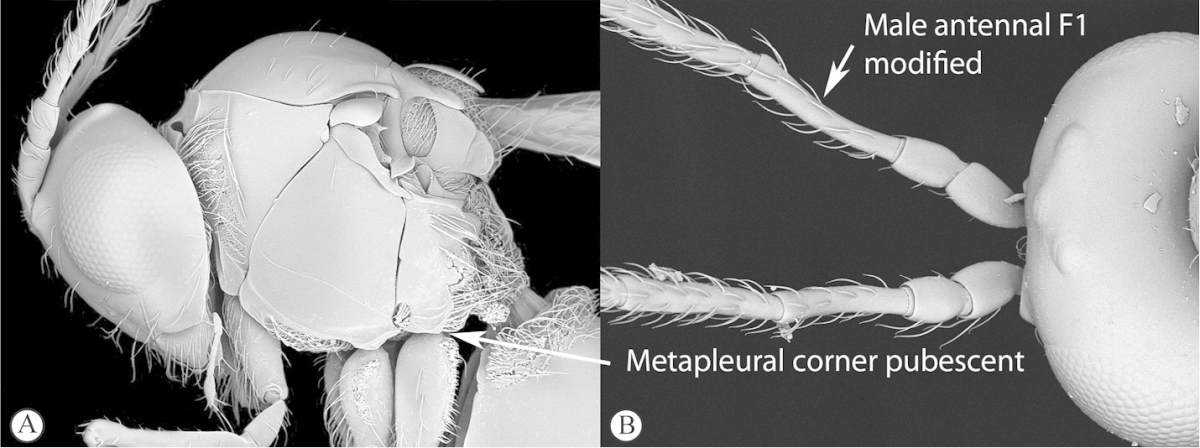	
–	Posteroventral corner of metapleuron always pubescent, angle often rectilinear or pointed (rarely oblique), never raised or forming a particular surface (A). Antennal F1 modified in males, asymmetric and more or less strongly curved (B), while F2 is not modified	**19 (Ganaspini)**
	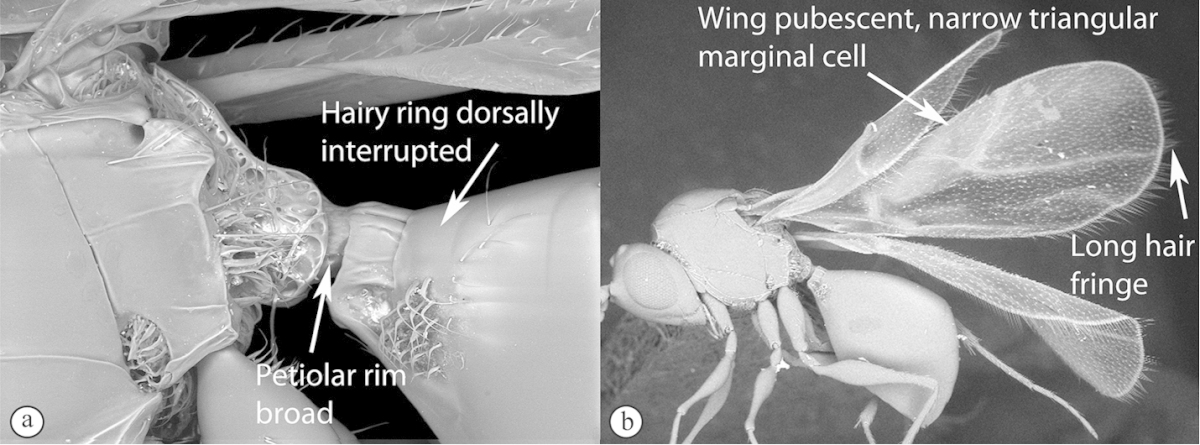	
15	Compact wasps, usually small, typically less than 2 mm long. Petiolar rim broad, hairy ring of metasoma dorsally interrupted (a). Metapleural triangle and subalar pit moderately developed (b). Wing always pubescent, usually with a rather narrow triangular marginal cell and a long hair fringe (b)	***Leptopilina***
	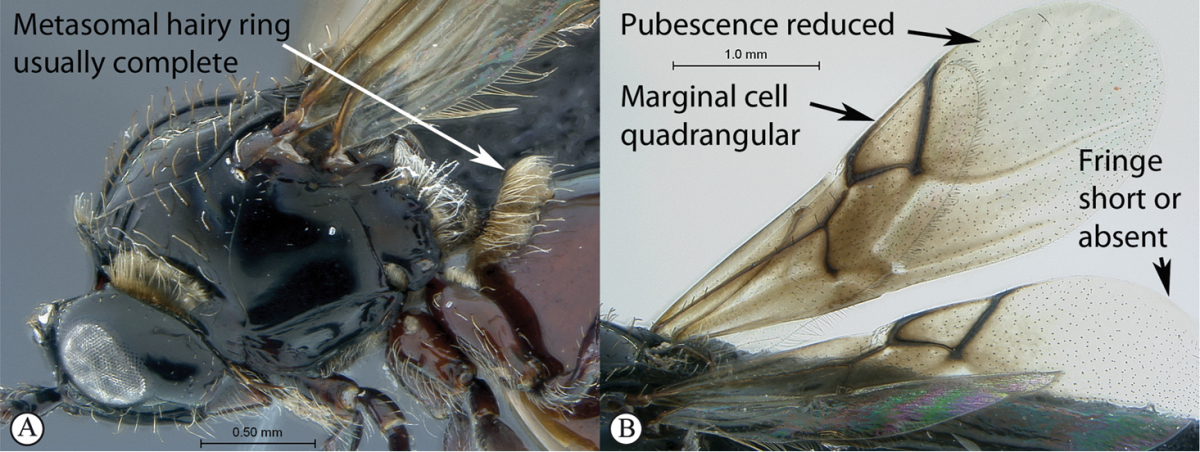	
–	Often large wasps, commonly over 2 mm in length. Petiolar rim indistinct, hairy ring of metasoma usually complete (A), occasionally briefly interrupted dorsally. Metapleural triangle and subalar pit well developed. Wing often with reduced pubescence (B), usually with a deep and long quadrangular marginal cell (B), and a short hair fringe (or no hair fringe) (B)	**16**
	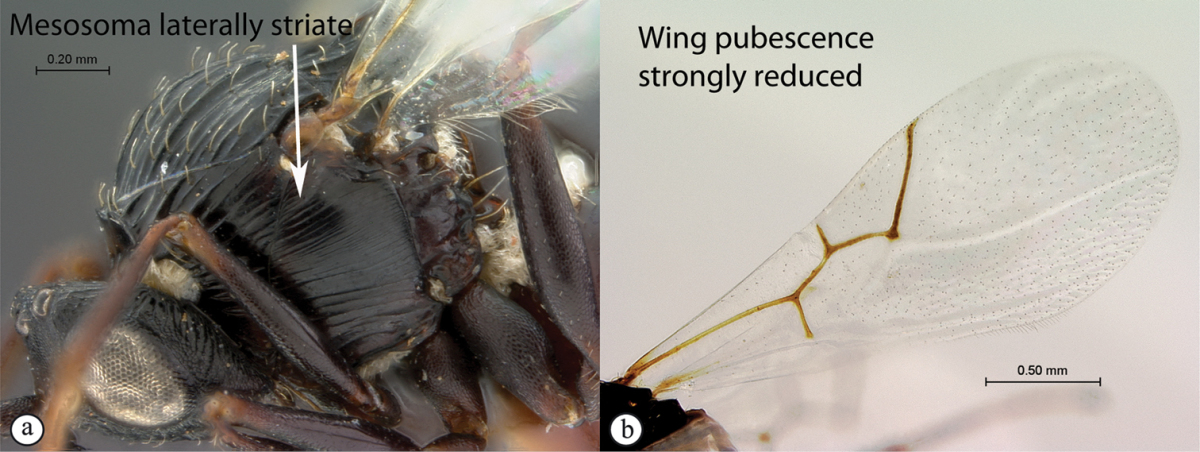	
16	Sides of mesosoma with strongly striate sculpture (a). Strongly reduced pubescence on wing membrane, usually more or less hairless (b)	**17**
	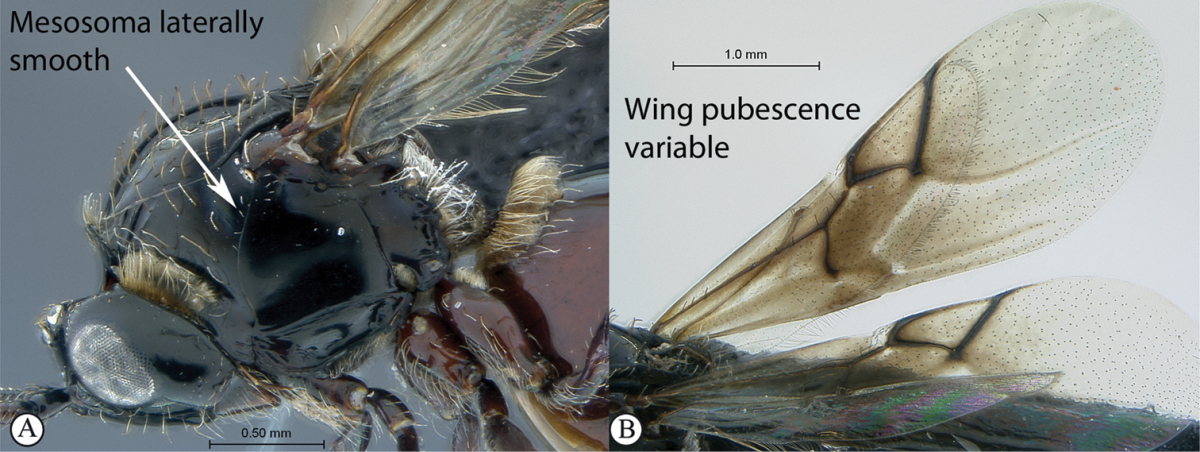	
–	Sides of mesosoma without striate sculpture (A). Wing pubescence variable (B)	**18**
	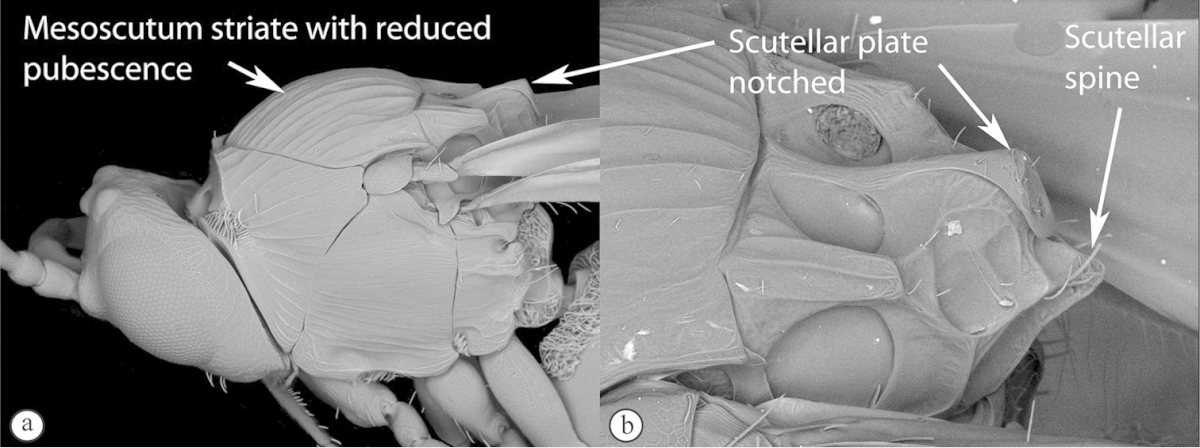	
17	Mesoscutum striate (a). Scutellum with a posterior spine (b), scutellar plate notched. Pronotum and mesoscutum with strongly reduced pubescence, almost absent (a, b)	***Afrodontaspis***
	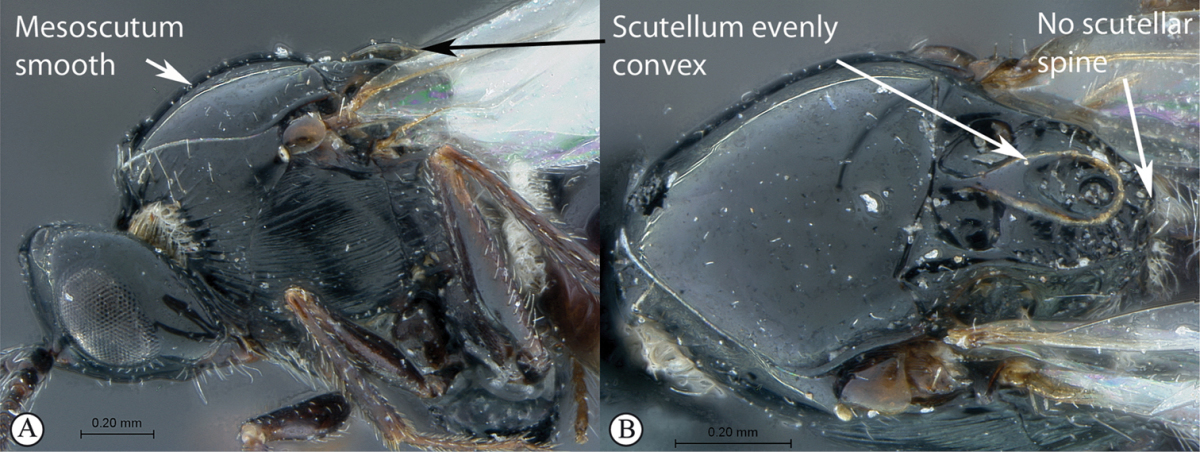	
–	Mesoscutum smooth (A, B). Scutellum without a posterior spine, scutellar plate weakly convex in lateral view (A, B). Pronotum and mesoscutum with scattered setae (A, B)	***Linoeucoila***
	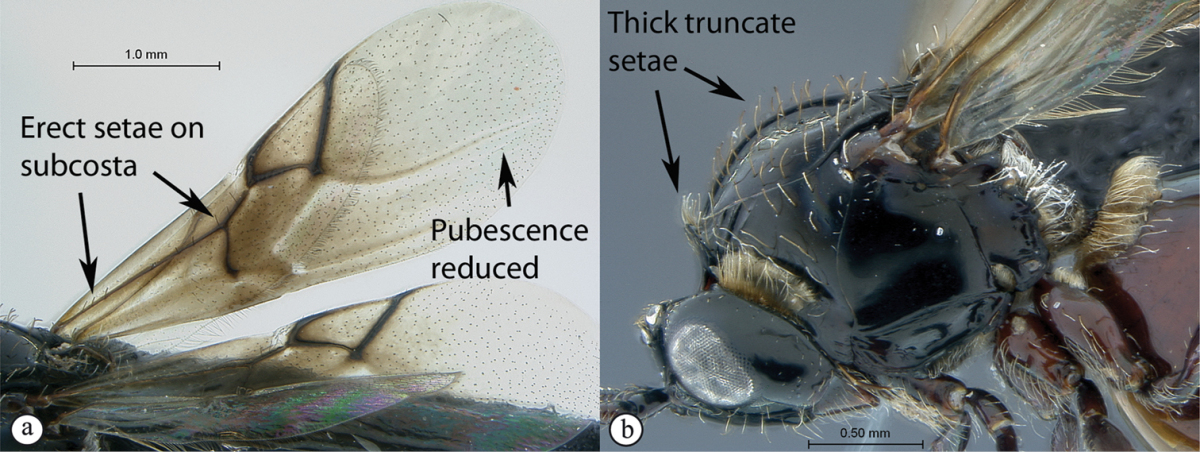	
18	Wing with erect setae on subcosta (a), and often with strongly reduced pubescence (a). Pronotum (and often mesoscutum) with thick truncate setae (b). Coxae often with reticulate-vermiculate sculpture. Scutellar plate convex or even notched, scutellar foveae usually very large	***Bothrochacis***
	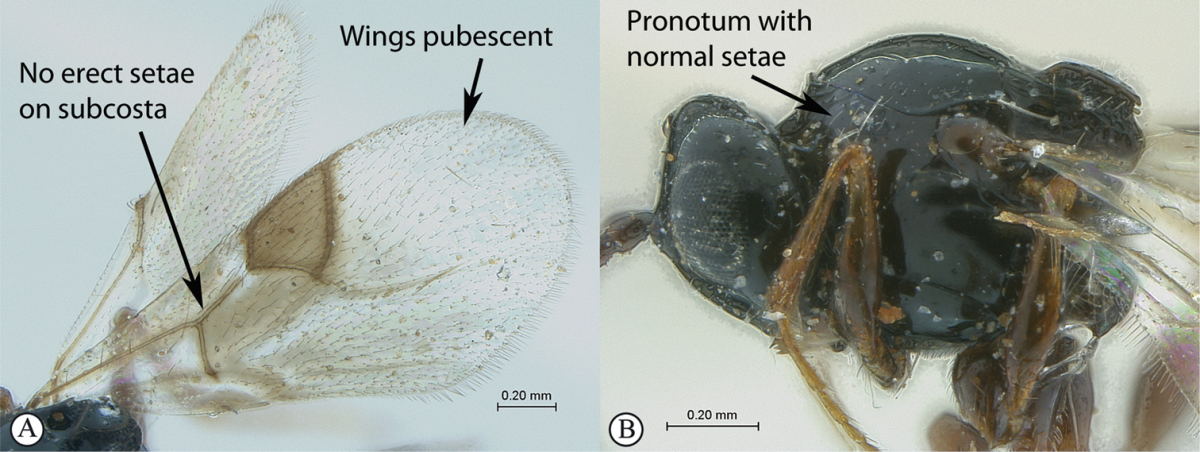	
–	No erect setae on subcosta (A). Wing usually normally pubescent (A), sometimes with reduced pubescence. Pronotum with at most a few thick truncate setae among a majority of normal, thin and pointed setae (B). Coxae always smooth. Scutellar plate straight or convex but never notched, scutellar foveae not very large	***Trybliographa***
	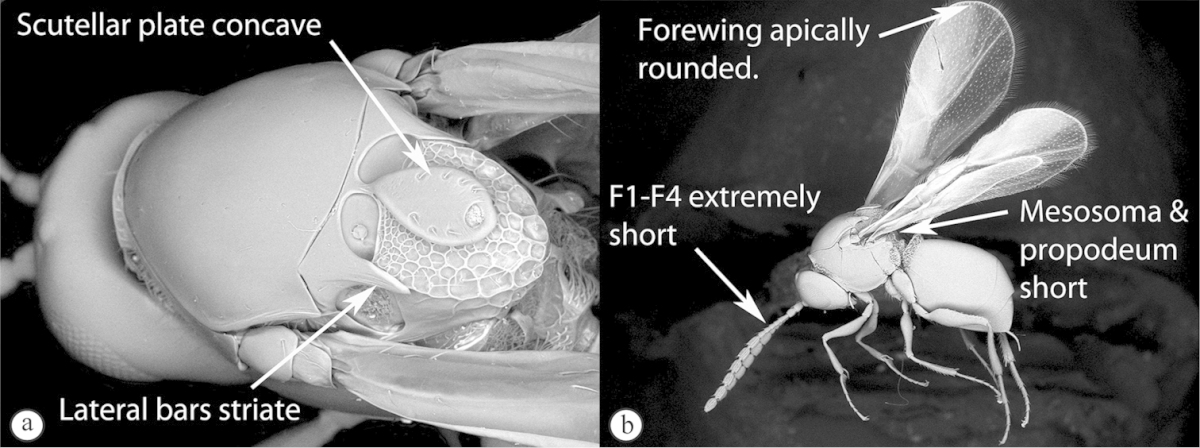	
19	Surface of scutellar plate concave, relatively narrow (a). Mesosoma short, only slightly longer than high (b); propodeum short, not protruding (b). Marginal cell typically distinctly half-closed (R1 vein along fore margin either ends or goes abruptly from pigmented to transparent at half length of marginal cell); occasionally indistinctly so (and sometimes entirely open in very small specimens). Lateral bars of scutellum typically striate (a). Head typically transverse (globular in very small specimens). Forewing shape apically rounded (b). Female antenna with F1–F4 extremely short, annelliform, resulting in a very striking clava (b)	**20**
	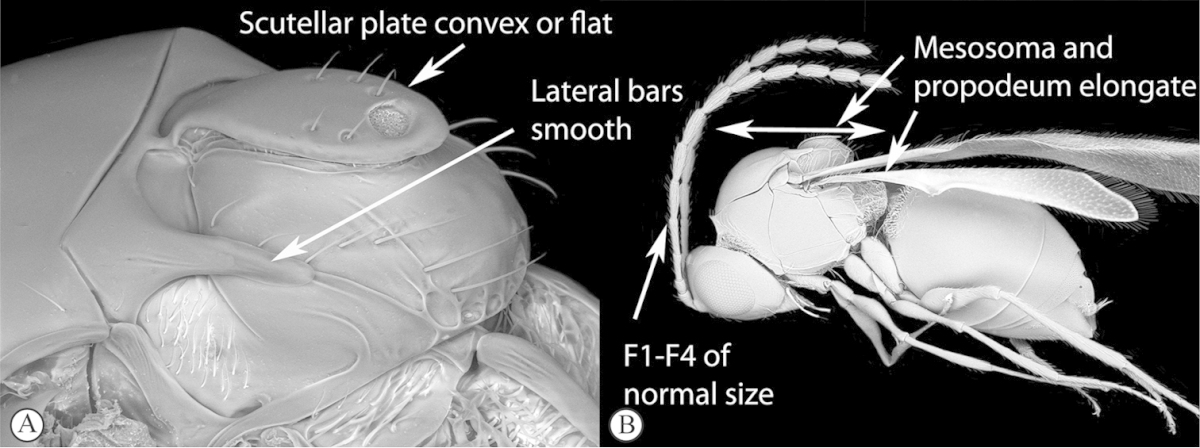	
–	Surface of scutellar plate convex or flat, often very large (A). Mesosoma elongate, clearly longer than high (B); propodeum protruding. Marginal cell variable, open or closed or indistinctly half-closed. Lateral bars of scutellum typically smooth (A). Head typically deep, often globular. Forewing shape typically with apex more triangular, truncate or faintly incised. Female antenna with F1–F4 of normal size (B), clava indistinct or distinct	**21**
	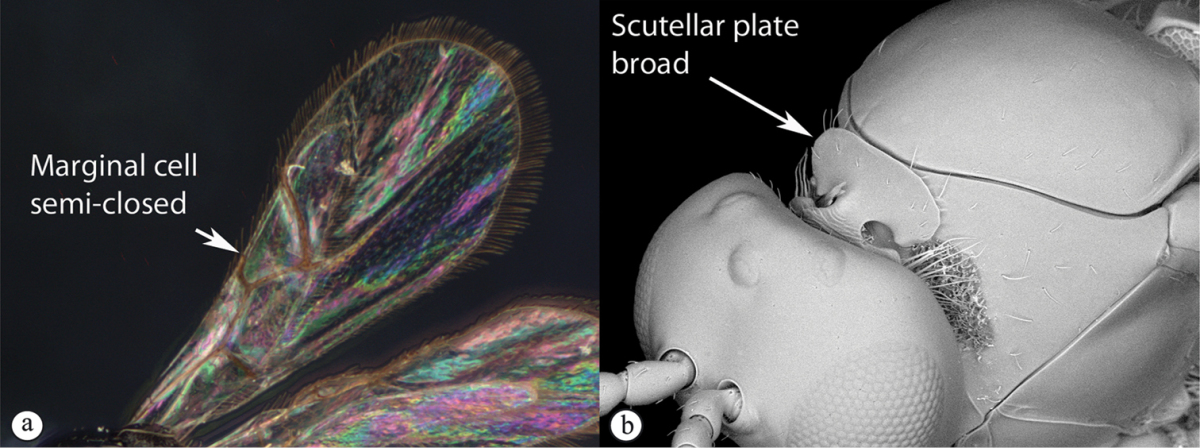	
20	Marginal cell of fore wing semi-closed (a). Head transverse. Scutellar plate about as wide as long, not narrow (b). Apical hair fringe of fore wing variable, typically short (a). Size variable	***Didyctium***
	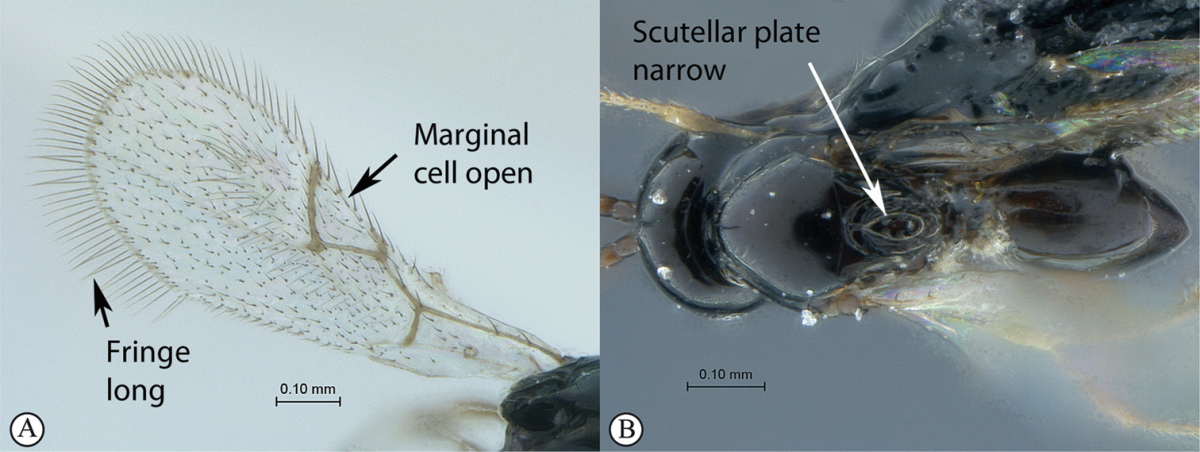	
–	Marginal cell of fore wing open (A). Head globular, about as deep as tall. Scutellar plate narrow, distinctly longer than wide (B). Apical hair fringe on fore wing always distinctly long (A). Always tiny (less than 1 mm) (B)	***Endecameris***
	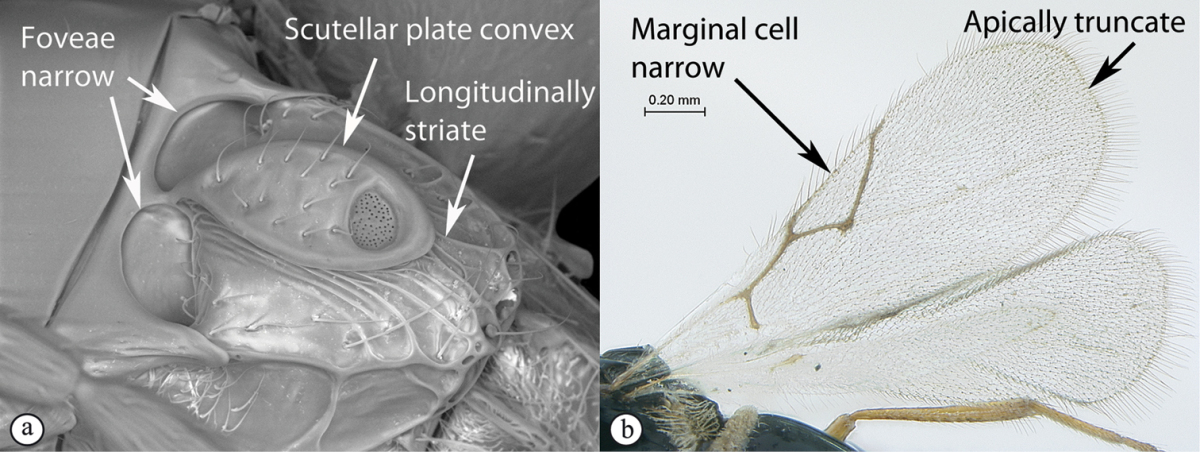	
21	Scutellum strongly convex, surface of scutellar plate convex and usually covering most of scutellum (a); scutellar foveae oriented obliquely relative to midline (a); dorsal surface of scutellum typically longitudinally striate (a). Marginal cell narrow, elongate-triangular (2r and RS straight and of equal length, with an open angle between them), typically closed (b). Wing elongate, relatively narrow, apically truncate (b). Metacoxae with a small tuft of hairs	***Hexacola***
	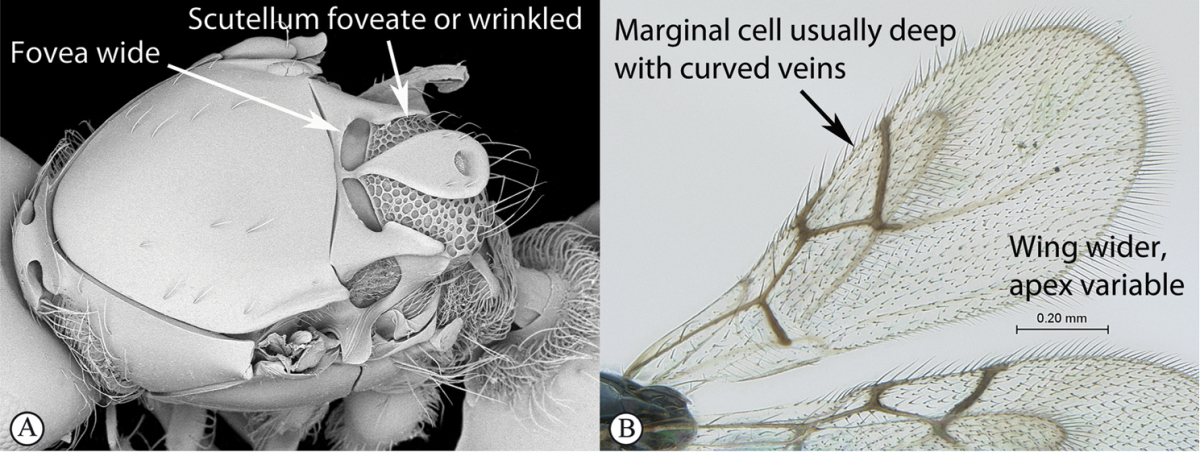	
–	Scutellum gently convex, surface of scutellar plate flat or convex, of variable size and width (A); scutellar foveae nearly perpendicular relative to midline; dorsal surface of scutellum variable, foveate or gently wrinkled, very rarely longitudinally striate (A). Marginal cell variable, usually deep with curved veins, open or closed (B). Wing shape wider, apex variable (B). Metacoxae usually with long hairline (occasionally short)	**22**
	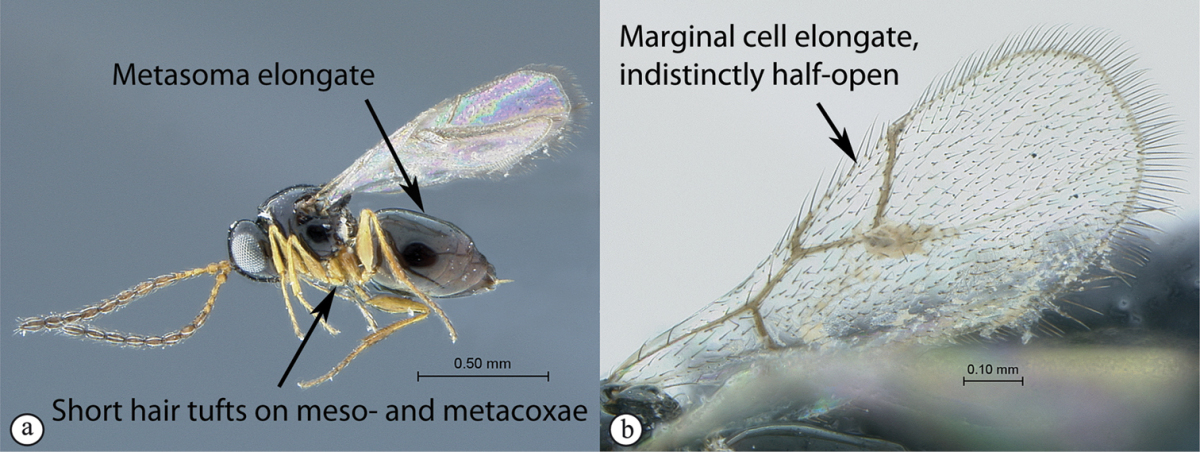	
22	Female metasoma extended, elongate (a). Short hair tufts on meso- and metacoxae. Marginal cell elongate, indistinctly half-open (b)	***Gastraspis***
	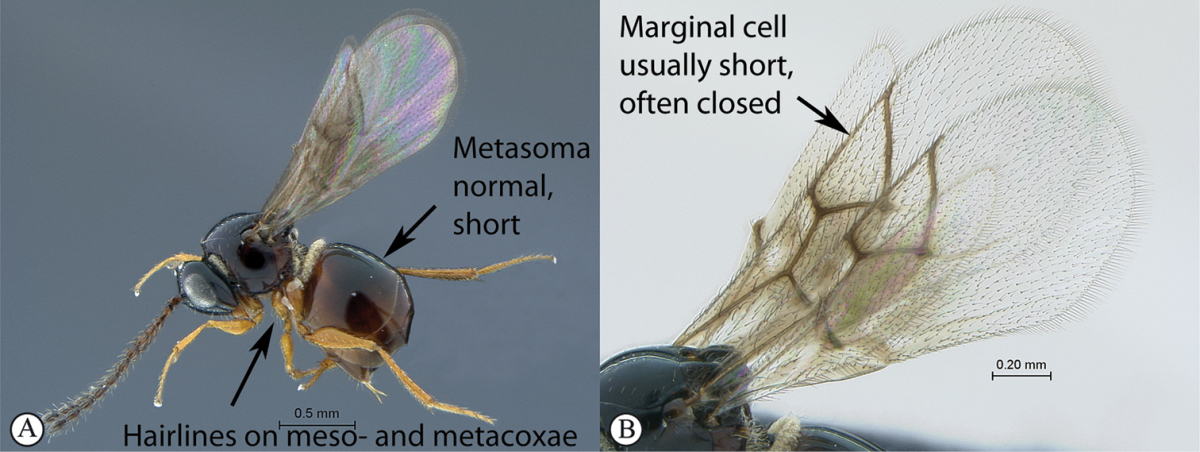	
–	Female metasoma normal, short (A). Usually hairlines on meso- and metacoxae (A), sometimes short hair tufts. Marginal cell variable, usually relatively short and often distinctly closed (B)	**23**
	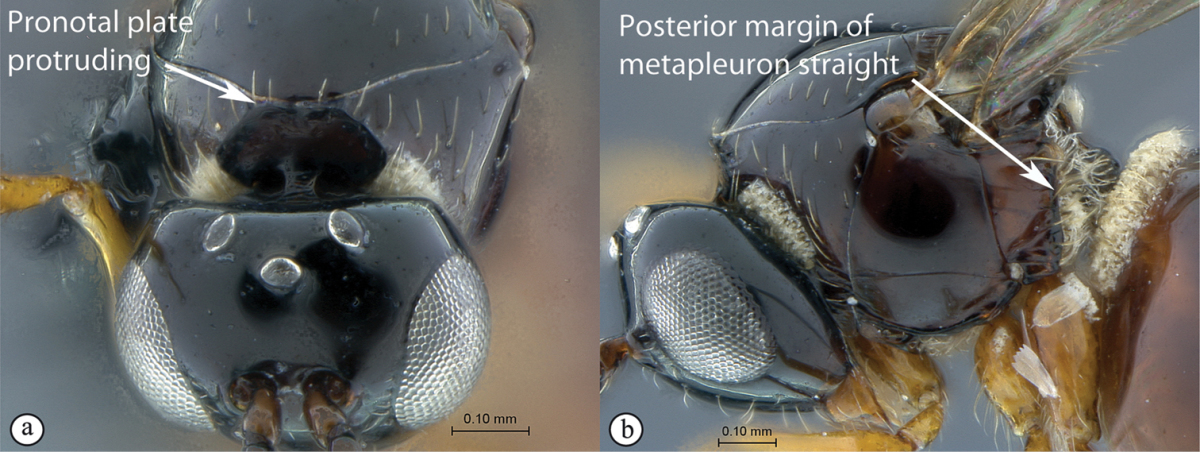	
23	Large wasps (2–3 mm), strongly built, black or dark brown wasps with dark appendages. Pronotal plate more or less protruding over pronotal-mesoscutal suture (a). Posterior margin of metapleuron straight (b). Rarely collected	***Aganaspis***
	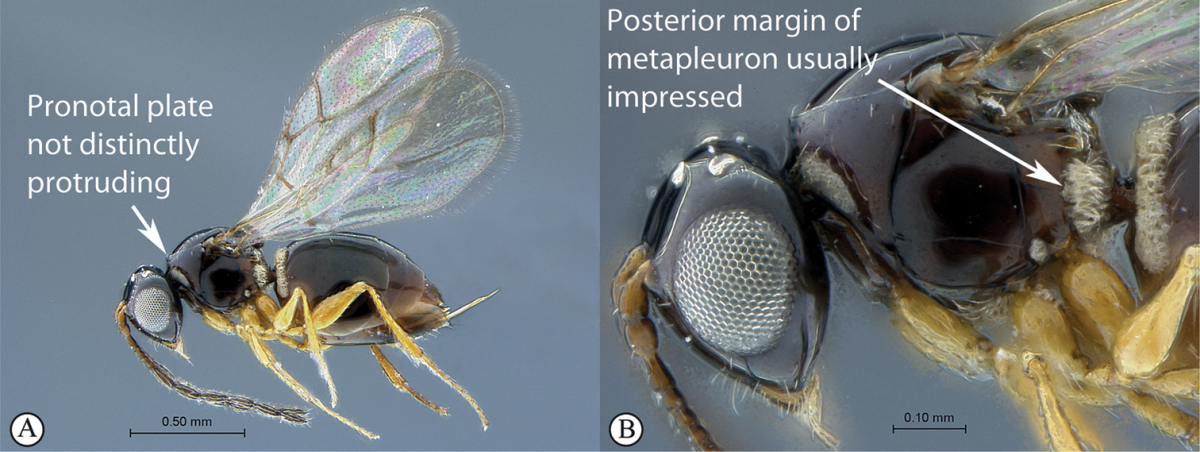	
–	Typically small, compact or elongate but not strongly built, brown wasps with usually yellow appendages (sometimes with bi- or tricolored antennae). Pronotal plate not distinctly protruding over pronotal-mesoscutal suture (A). Posterior margin of metapleuron usually with a circular or elongate incision (B), rarely straight. Common	***Ganaspis***
	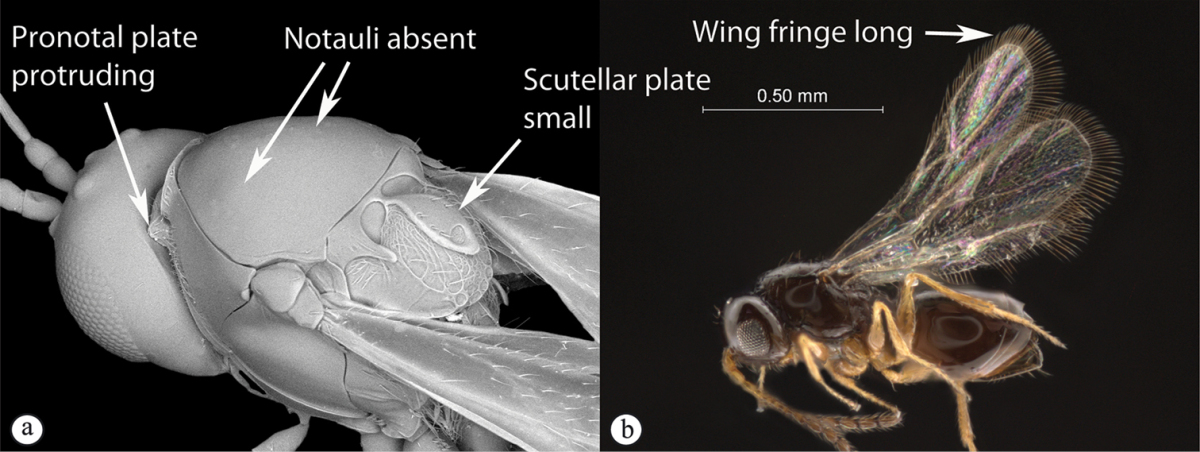	
24	Elongate or slender wasps (a, b). Notauli absent (a). Protrusions from lower face absent. Lateral pronotal carina absent. Antennal F2 modified in male. Hair fringe on wings long (b). Scutellar plate typically small, thinly elongate or teardrop-shaped, covering less than half of the dorsal surface of the scutellum (a). Anterior part of pronotal plate protruding (a)	**25**
	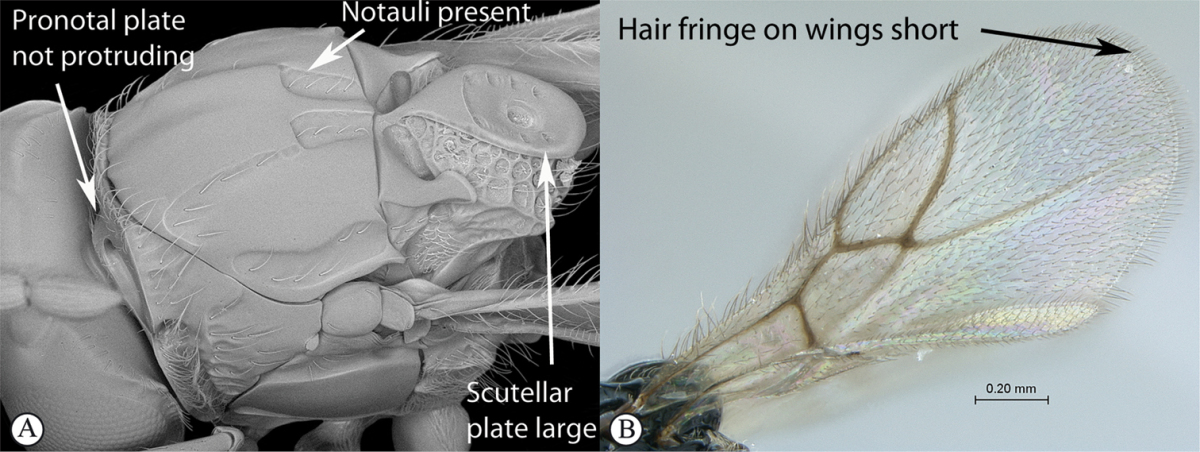	
–	Compact wasps. Notauli present, often incomplete mesally, rarely reduced to mere anterior impressions of the mesoscutum (A). Clypeal and malar protrusions on face often present. Lateral pronotal carina present to absent. Antennal F1 modified in male. Hair fringe on wings short (B). Scutellar plate often large, elongate, covering over half of the dorsal surface of the scutellum (A). Anterior part of pronotal plate not protruding (A)	**27 (Diglyphosematini)**
	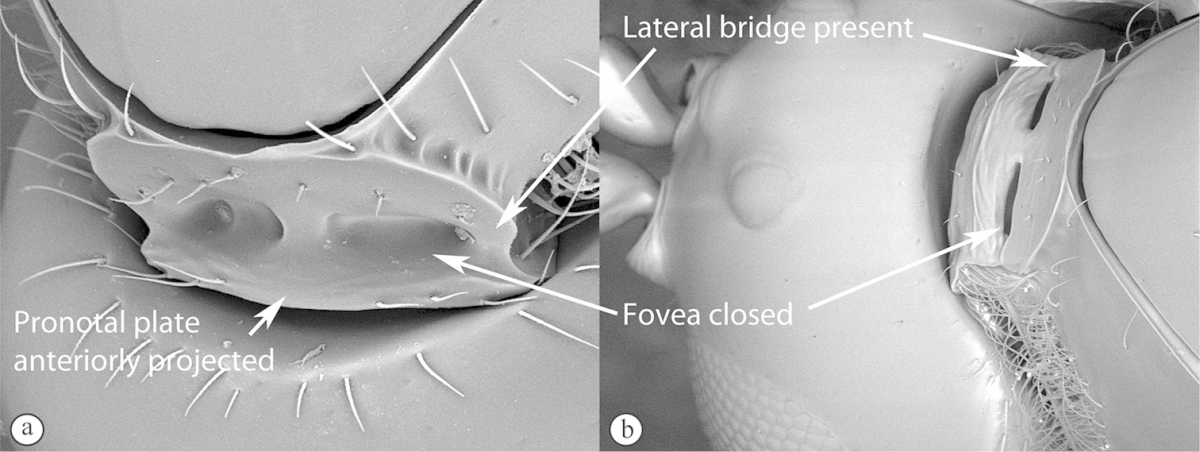	
25	Lateral foveae of the pronotal plate closed, lateral bridges complete (a, b); pronotal plate projected anteriorly (a)	***Rhoptromeris*** (in part) **(Trichoplastini)**
	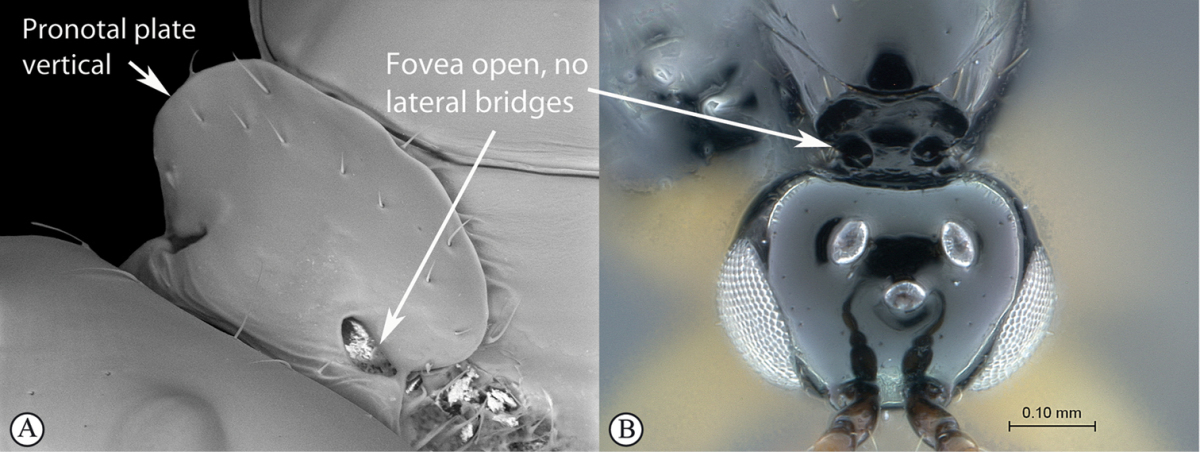	
–	Lateral fovea of the pronotal plate open, lateral bridges absent (A, B); pronotal plate typically oriented vertically (A), not projected anteriorly	**26**
	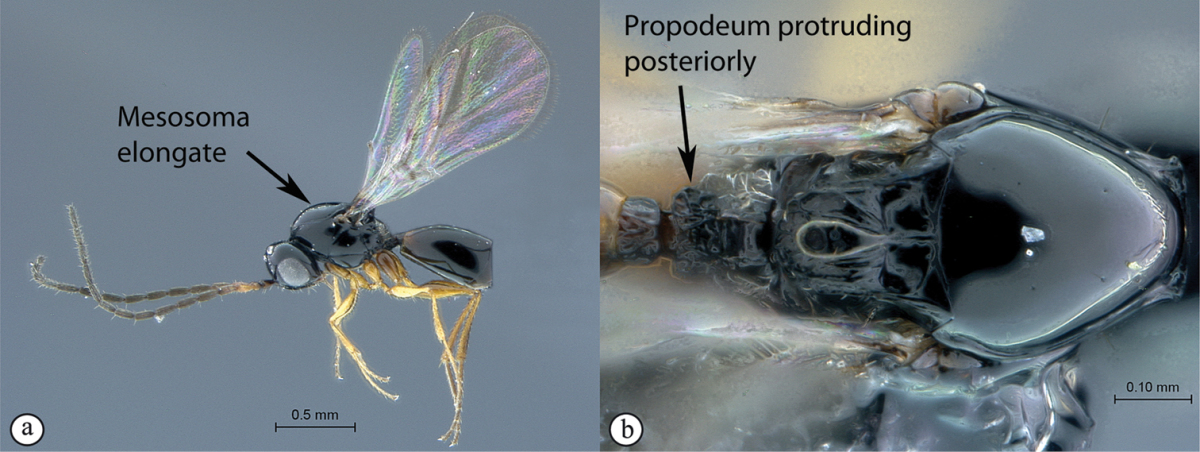	
26	Elongate wasps, “diggers”, with deep rounded heads, small eyes, strong legs, and short wings (a). Mesosoma remarkably elongate, much longer than high (a), with an almost flat mesoscutum (a); propodeum distinctly protruding posteriorly (b). Rare	***Cothonaspis* (Kleidotomini)**
	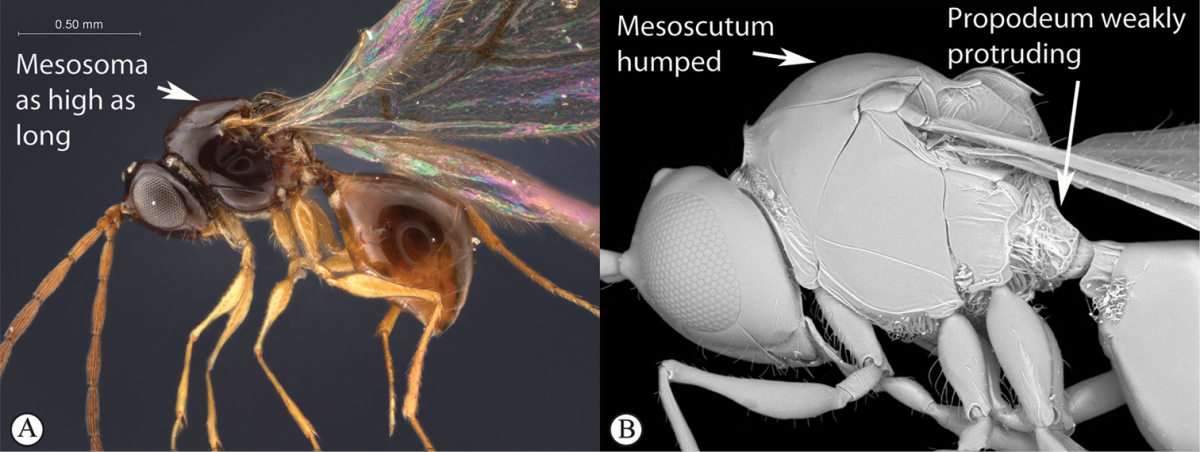	
–	More or less slender wasps, “flyers”, with more or less transverse heads, large eyes, long legs, and large wings. Mesosoma as high as long (A), mesoscutum obviously humped (B); propodeum weakly protruding posteriorly (B). Very common	***Leptopilina*** (in part) **(Eucoilini)**
	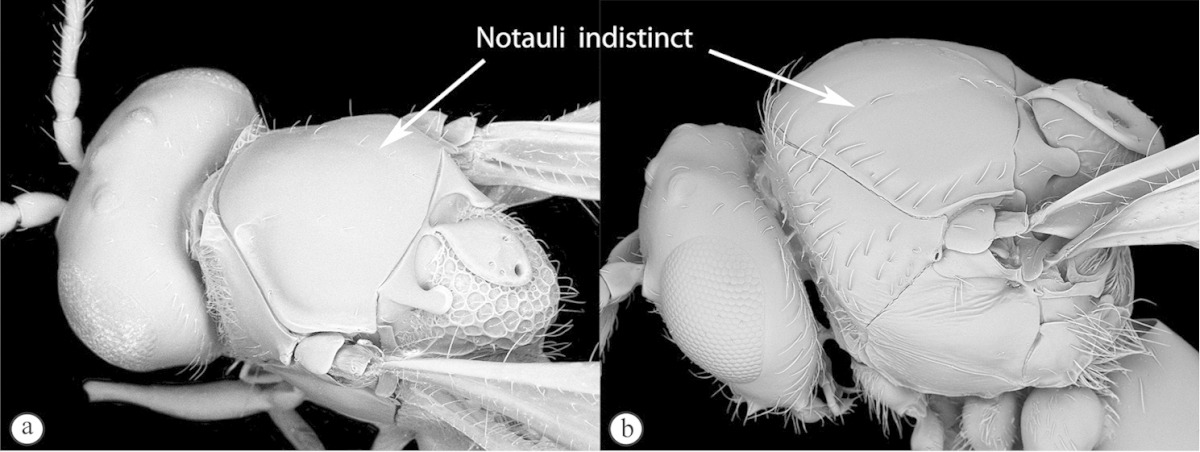	
27	Notauli reduced; shortened, shallow and/or indistinct (a, b)	**28**
	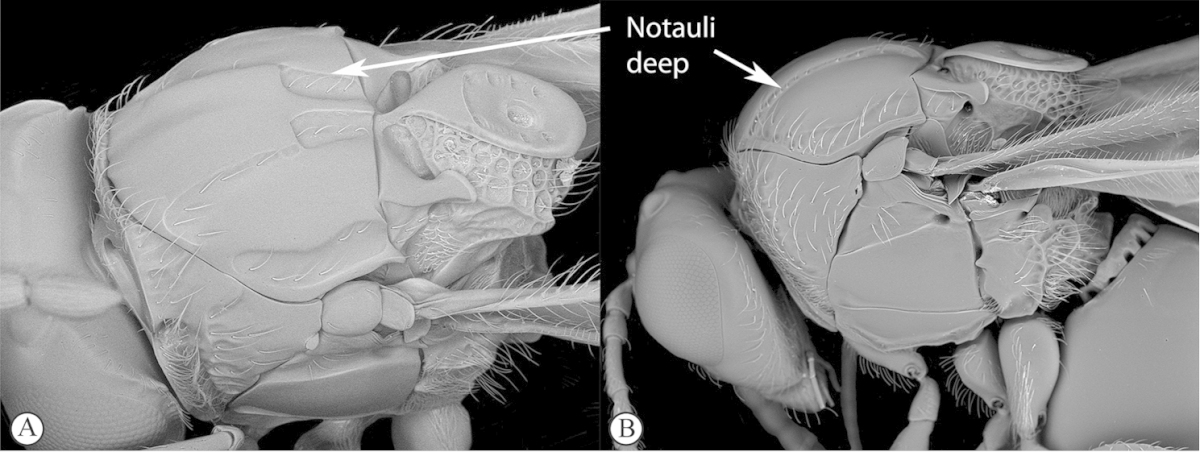	
–	Notauli well-developed; deep, wide and often sculptured (A, B)	**29**
	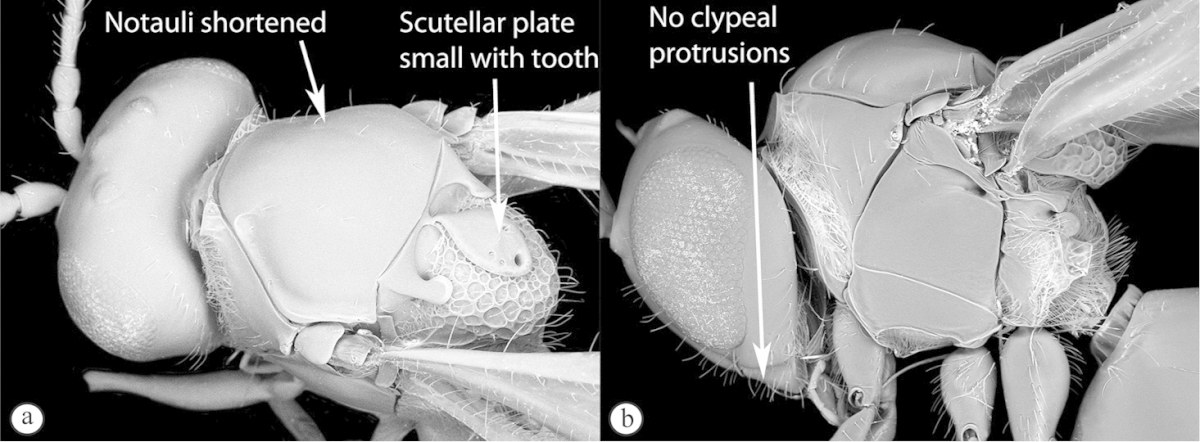	
28	Notauli shortened, only anteriormost part visible, indistinct over the rest of the mesoscutum (a). Face without protrusions (b). Scutellar plate small, with tooth just anterior to glandular pit (a); scutellum broadly rounded posteriorly, distinct posterior face absent (a)	***Ealata***
	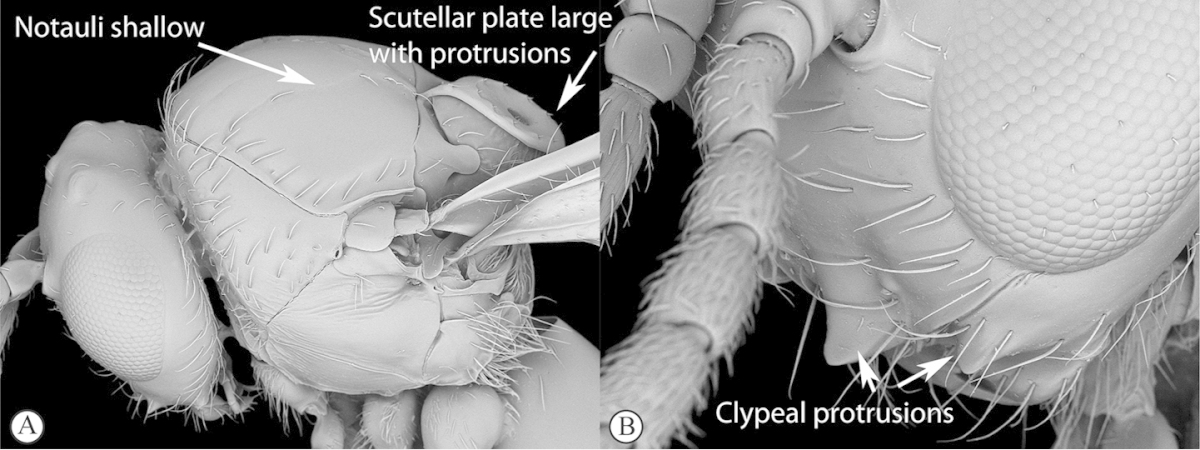	
–	Notauli shallow and indistinct for their entire length, often not visible in middle of mesoscutum (A). Clypeus and malar spaces each with a single conical or pyramidal protrusion, sometimes reduced (B). Scutellar plate large, flat, often with several small paired protuberances along the perimeter; scutellum with distinct posterior face, ventral to gentle ledge along posterior margin of scutellum	***Nordlanderia***
	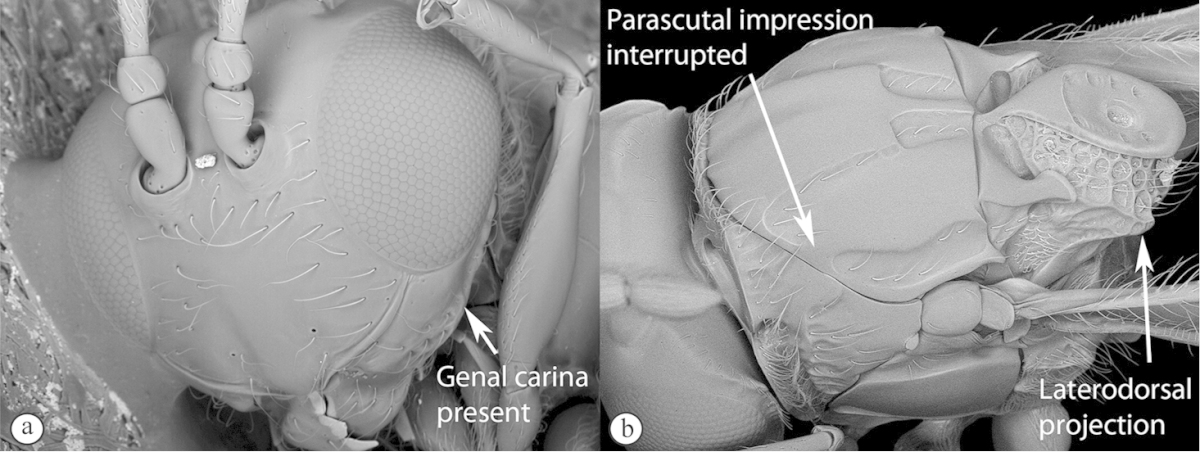	
29	Genal carina present (a). Scutellum with faint laterodorsal projections (b). Parascutal impression interrupted near origin of notauli (b)	***Paradiglyphosema***
	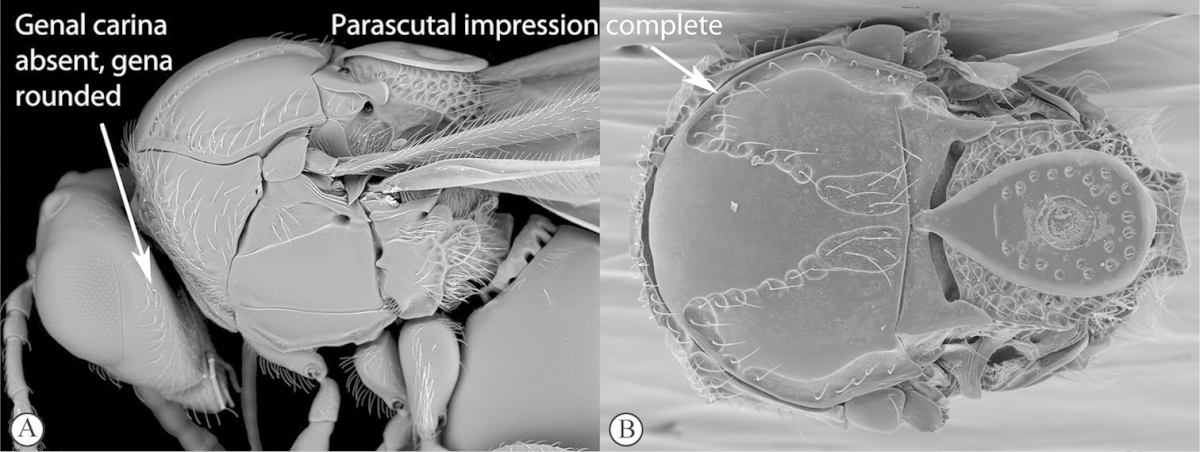	
–	Genal carina absent, gena rounded (A). Scutellum without laterodorsal projections. Parascutal impression complete, not interrupted (B)	**30**
	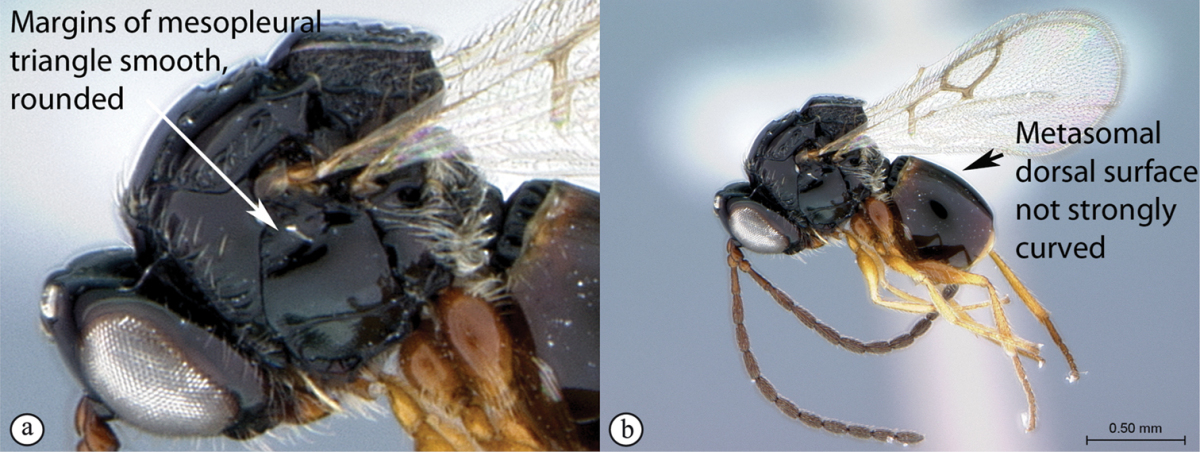	
30	Dorsal and ventral margins of mesopleural triangle smooth, rounded, indistinct (a). Female metasoma directed posteriorly, dorsal surface not strongly curved downward (b)	***Gronotoma***
	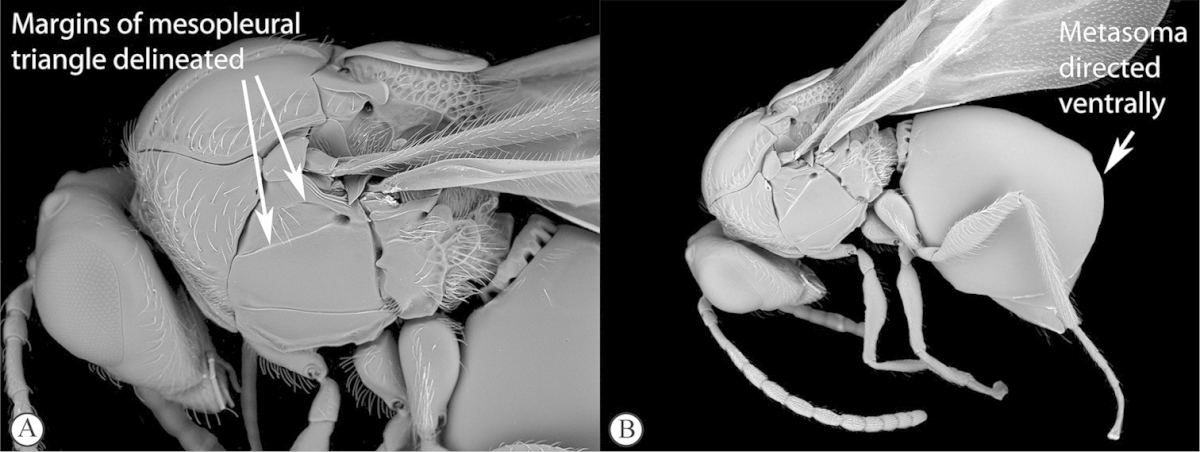	
–	Dorsal and ventral margins of mesopleural triangle cleft, distinctly delineated (A). Female metasoma directed ventrally, dorsal surface strongly curved downward (B)	***Afrostilba***

#### Diglyphosematini

Diglyphosematini is a characteristic and rather easily circumscribed tribe, which was overviewed and revised on the generic level by Buffington (2011). Species of this tribe can be locally abundant, especially *Afrostilba*. According to all vetted records, species are parasitoids of leaf-mining Diptera (Agromyzidae) (Buffington 2011).

##### Afrostilba

Taxon classificationAnimaliaHymenopteraFigitidae

Benoit, 1956a stat. n.

Afrostilba (synonym *Amphiglyphosema* Benoit, 1956a, **syn. n.**)

###### Remarks.

Commonly collected genus of eucoilines throughout Africa, but particularly plentiful in equatorial Africa. The genus is common throughout the Old World Tropics as well as Mediterranean Africa. Quinlan (1986) made it a junior synonym of *Gronotoma* Förster, and Buffington (2002, 2011) followed this synonymy. The phylogenetic support for the inclusion of *Gronotoma nitida* Benoit (the only representative of *Afrostilba* in Buffington 2011) in *Gronotoma* s.str. was very weak; in that same work, it was noted that there is a distinct lineage of African species. After examination of much more recently collected material, it is clear that the African species reliably cluster with Benoit’s (1956a) generic concept of *Afrostilba*. Examination of the type specimens of Quinlan’s (1986) *Eucoilidea* species, as well as *Diglyphosema sensu* Quinlan, has allowed us to make the new combinations below. Furthermore, examination of the type specimen of *Amphiglyphosema*, *Afrostilba latesulcatum* Benoit, allows us to synonymize *Amphiglyphosema* with *Afrostilba*. However, there are still species of Afrotropical *Gronotoma* of which we have not yet been able to examine the types, which may be *Afrostilba* or true *Gronotoma*, and these species are still listed under *Gronotoma* below.

###### Diagnosis.

Mesopleural triangle distinctly impressed, with both dorsal and ventral margins cleft, delimited by a sharp edge. Lateral pronotal carina present. Notauli present, well developed in nearly all species. Scutellar plate large, glandular pit in center. Hairy ring at base of syntergum absent; metasoma downturned towards ventral position. Most easily confused with *Diglyphosema* and *Gronotoma*; distinguished from these genera by the presence of a distinctly impressed mesopleural triangle, and downturned metasoma. In both *Diglyphosema* and *Gronotoma*, the mesopleural triangle is present, but the dorsal and ventral margins are gently rounded, and the metasoma is directed more posteriorly. Distinguished from *Paradiglyphosema* by the possession in the latter of a genal carina and postero-lateral projections on the scutellum.

Five species groups are recognized within the genus, all based on the morphology of the scutellum. This character system provides a wealth of taxonomic information, and from the hundreds of specimens examined for this paper, the characters appear to be stable through space and time. The most commonly encountered species group is the *Afrostilba nitida* group, characterized by having a distinctly concave dorsal surface of the scutellar plate. This is best seen in lateral view, and can be characterized as reminiscent of a gentle ‘wave’ or ‘ski jump’. From our examination of the type series of all of Quinlan’s (1986) species, several species in this group will be synonymized in a more thorough treatment of the genus. The *Afrostilba dubia* species group is characterized by having an extremely short, narrow scutellar plate, revealing much of the dorsal surface of the scutellum. Some specimens in this group approach an *Ealata* in appearance, but lack other characters of that genus. The *Afrostilba bucca* species group is second in diversity to the *Afrostilba nitida* species group, and superficially looks similar. However, in the *Afrostilba bucca* species group, the scutellar plate, in profile, is perfectly flat (wave or ski-jump shaped in the *Afrostilba nitida* species group), with a large, deep glandular release pit. The last species group to be recognized here is the *Afrostilba fercula* species group. On first glance, these species look similar to the *Afrostilba bucca* species group, however, species in the *Afrostilba fercula* group possess a very small, shallow, glandular release pit. The appearance of the scutellar plate is remarkable in that the rim of the plate appears enormous, when in fact, the rim is of normal width; it is the small glandular release pit contributing to this illusion.

**Figure 18.**
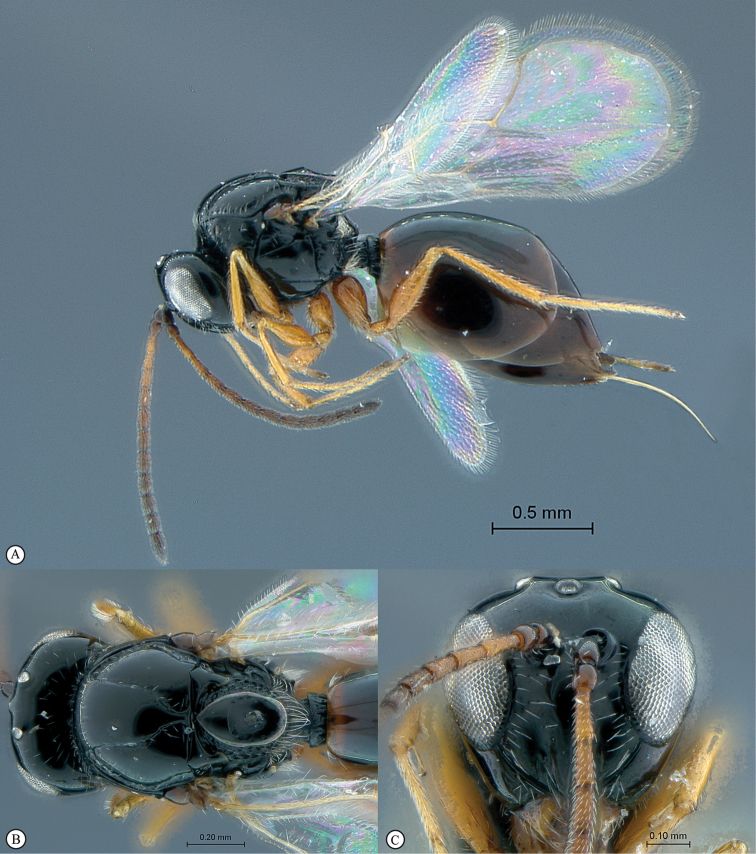
*Afrostilba* species (Tanzania). **A** habitus lateral view **B** head and mesosoma dorsal view **C** head, anterior view.

###### Distribution.

Endemic to Old World Tropics. Afrotropical records: Burundi, Democratic Republic of Congo, Rwanda, (Benoit 1956), Cameroon, Ethiopia, Ghana, Kenya, Madagascar, Mauritius, Nigeria, Senegal, South Africa, Tanzania, Uganda, Yemen, Zambia, Zimbabwe (Quinlan 1986, 1988), Botswana, Cape Verde, Central African Republic, Gabon, Ivory Coast, Niger, Republic of Congo, Somalia, Tanzania (here).

###### Biology.

Parasitoids of leaf-mining Agromyzidae (Buffington 2002, 2011, Greathead 1969).

##### *Afrostilba bucca* species group

*Afrostilba advena* (Quinlan, 1986), **comb. n.** (*Eucoilidea*) (Cameroon, Democratic Republic of Congo, Kenya, South Africa, Uganda, Yemen, Zambia, Zimbabwe)

*Afrostilba bucca* (Quinlan, 1986), **comb. n.** (*Eucoilidea*) (Democratic Republic of Congo, Ethiopia, Kenya, South Africa, Uganda, Yemen, Zambia)

*Afrostilba fetura* (Quinlan, 1986), **comb. n.** (*Eucoilidea*) (Cameroon, Democratic Republic of Congo, Nigeria, Senegal, South Africa, Uganda, Zimbabwe)

*Afrostilba pallida* (Quinlan, 1986), **comb. n.** (*Eucoilidea*) (Democratic Republic of Congo, South Africa, Yemen)

##### *Afrostilba dubia* species group

*Afrostilba dubia* (Quinlan, 1986), **comb. n.** (*Eucoilidea*) (Democratic Republic of Congo, South Africa)

##### *Afrostilba furcula* species group

*Afrostilba furcula* (Quinlan, 1986), **comb. n.** (*Eucoilidea*) (Democratic Republic of Congo, Ethiopia, Kenya, South Africa, Uganda, Zimbabwe)

*Afrostilba urundiensis* (Benoit, 1956a) **comb. n.** (*Eucoilidea*) (Burundi, Uganda, Zimbabwe)

##### *Afrostilba nitida* species group

*Afrostilba compressa* (Quinlan, 1986), **comb. n.** (*Eucoilidea*) (Democratic Republic of Congo, Kenya, South Africa, Uganda, Zimbabwe)

*Afrostilba conversa* (Quinlan, 1986), **comb. n.** (*Eucoilidea*) (Cameroon, Democratic Republic of Congo, Ghana, Kenya, Madagascar, Mauritius, Nigeria, South Africa, Tanzania, Uganda, Zimbabwe)

*Afrostilba lacerta* (Quinlan, 1986), **comb. n.** (*Eucoilidea*) (Democratic Republic of Congo, Ivory Coast)

*Afrostilba lana* (Quinlan, 1986), **comb. n.** (*Eucoilidea*) (Democratic Republic of Congo)

*Afrostilba marcellus* (Quinlan, 1986), **comb. n.** (*Eucoilidea*) (Democratic Republic of Congo, Madagascar, Mauritius)

*Afrostilba nitida* Benoit, 1956, **comb. reinst.** (Burundi, Democratic Republic of Congo, Gabon, Ghana, Kenya, South Africa, Zambia, Zimbabwe)

*Afrostilba parma* (Quinlan, 1986), **comb. n.** (*Eucoilidea*) (Democratic Republic of Congo, Madagascar, Nigeria)

*Afrostilba perangusta* (Quinlan, 1986), **comb. n.** (*Eucoilidea*) (Democratic Republic of Congo, Zambia, Zimbabwe)

*Afrostilba trulla* (Quinlan, 1986), **comb. n.** (*Eucoilidea*) (Democratic Republic of Congo, South Africa)

*Afrostilba tyrus* (Quinlan, 1986), **comb. n.** (*Eucoilidea*) (Cameroon)

##### Unplaced *Afrostilba* species

*Afrostilba latesulcatum* (Benoit, 1956a), **comb. n.** (*Amphiglyphosema*) (Rwanda)

*Afrostilba utica* (Quinlan, 1988), **comb. n.** (*Diglyphosema*) (Ivory Coast, Nigeria)

###### Ealata

Taxon classificationAnimaliaHymenopteraFigitidae

Quinlan, 1986

####### Remarks.

Rare, mostly in East Africa.

####### Diagnosis.

Protuberances absent on malar space. Dorsal margin of pronotal plate with a distinct emargination. Notauli reduced, only present anteriorly and sometimes posteriorly. Scutellar plate small, with a mound like protuberance anterior of glandular pit. Dorsal surface of scutellum broadly rounded both laterally and posteriorly, distinct posterior aspect of scutellum absent. Separated from all other Diglyphosematini by the presence of a single, broad protuberance anterior of the glandular pit of the scutellum.

**Figure 19.**
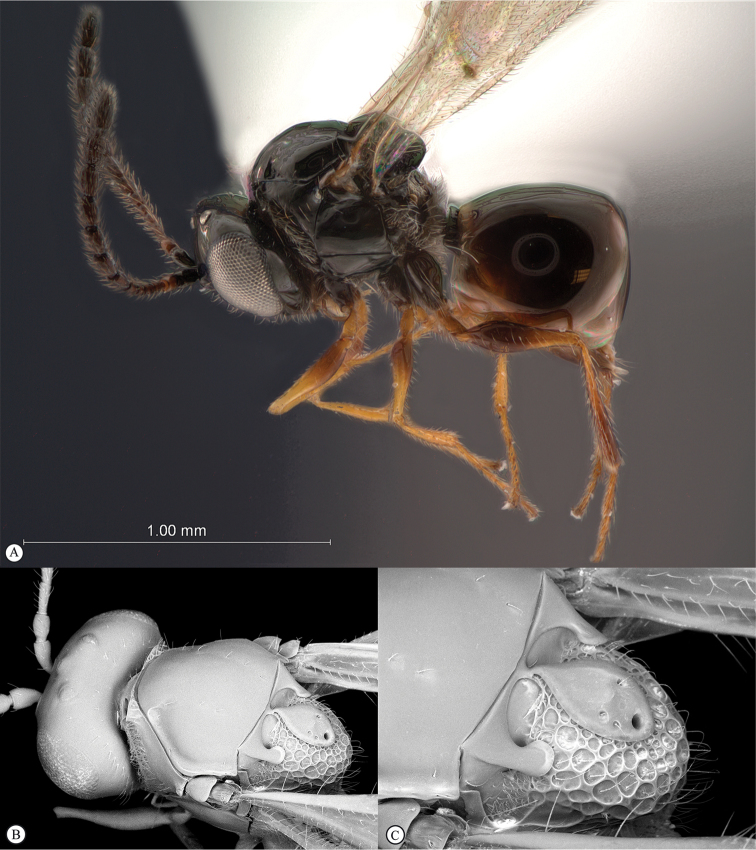
*Ealata* species (Kenya). **A** habitus lateral view **B** head and mesosoma dorsal view **C** scutellum, dorso-lateral view.

####### Distribution.

Found in the Oriental and Afrotropical regions. Afrotropical records: Cameroon, Democratic Republic of Congo, Kenya, Mauritius, Nigeria, Principé, South Africa, Uganda, Zimbabwe (Quinlan 1986), Botswana, Central African Republic, Ivory Coast, Madagascar, Republic of Congo, Somalia, Tanzania (here).

####### Biology.

Controversial, reviewed in Buffington (2011). Putatively reared from Tephritidae (unlike the rest of the Diglyphosematini, which attack leaf-mining Agromyzidae), but there are no isolated rearings to confirm this.

####### Species richness.

*Ealata clava* Quinlan, 1986 (Cameroon, Democratic Republic of Congo, Kenya, Mauritius, Principé, South Africa, Uganda)

*Ealata marica* Quinlan, 1986 (Democratic Republic of Congo)

*Ealata saba* Quinlan, 1986 (Democratic Republic of Congo, Nigeria, South Africa, Uganda, Zimbabwe)

###### Ganaspidium

Taxon classificationAnimaliaHymenopteraFigitidae

Weld, 1955

####### Remarks.

A New World genus that is rare in South Africa. Included here based on two individuals taken in the Western Cape.

####### Diagnosis.

Malar space and ventral clypeal margin with distinct conical protuberances. Notauli absent. Parascutal impression incomplete. Setal band at base of syntergum of metasoma complete. Superficially similar to *Nordlanderia*, but readily distinguished based on notauli being absent and the hairy ring of syntergum present; most similar to the New World genus *Banacuniculus* Buffington, but separated by the presence of two distinct tubercles anterior of the scutellar glandular pit (surrounded by a series of tubercles in *Banacuniculus*).

**Figure 20.**
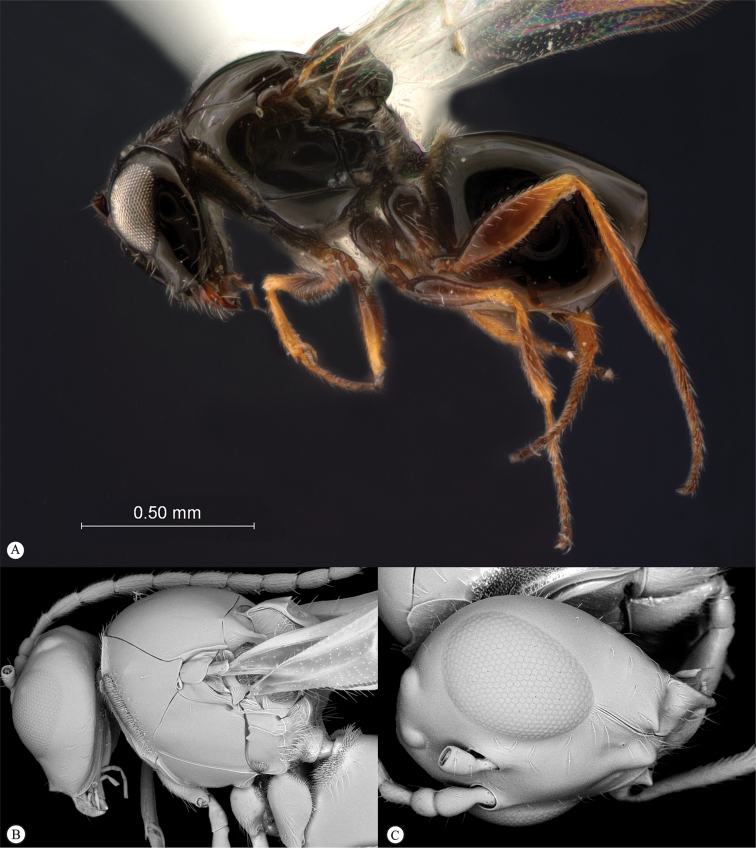
*Ganaspidium* (South Africa). **A** habitus lateral view **B** head and mesosoma lateral view **C** head, anterior-lateral view.

####### Distribution.

A New World genus: Western United States, southwestern Canada, and Northern Mexico (Buffington 2011). Here reported from the Old World for the first time based on two specimens from South Africa. Species of this genus are restricted to arid to semi-arid environments (Buffington 2011).

####### Biology.

Attacks leaf-mining Agromyzidae in arid habitats (Buffington 2011).

####### Species richness.

One undescribed species found in South Africa.

###### Gronotoma

Taxon classificationAnimaliaHymenopteraFigitidae

Förster, 1869

Gronotoma (synonym *Eucoilidea* Ashmead, 1887)

####### Remarks.

Rarely collected in Africa. Many African species previously listed in Buffington (2011) are currently moved to *Afrostilba* Benoit. Holotypes for species listed below have not yet been examined, hence we prefer to leave them in *Gronotoma* for the time being.

####### Diagnosis.

Mesopleural triangle gently impressed, dorsal and ventral margins rounded. Lateral pronotal carina present. Notauli present, well developed in most species. Scutellar plate large, glandular pit in center. Hairy ring at base of syntergum absent; metasoma downturned towards ventral position. Most easily confused with *Afrostilba* and *Paradiglyphosema*; distinguished from these genera by the absence of a distinctly impressed mesopleural triangle, and downturned metasoma. Further distinguished from *Paradiglyphosema* by the possession in the latter of a genal carina and postero-lateral projections on the scutellum.

**Figure 21.**
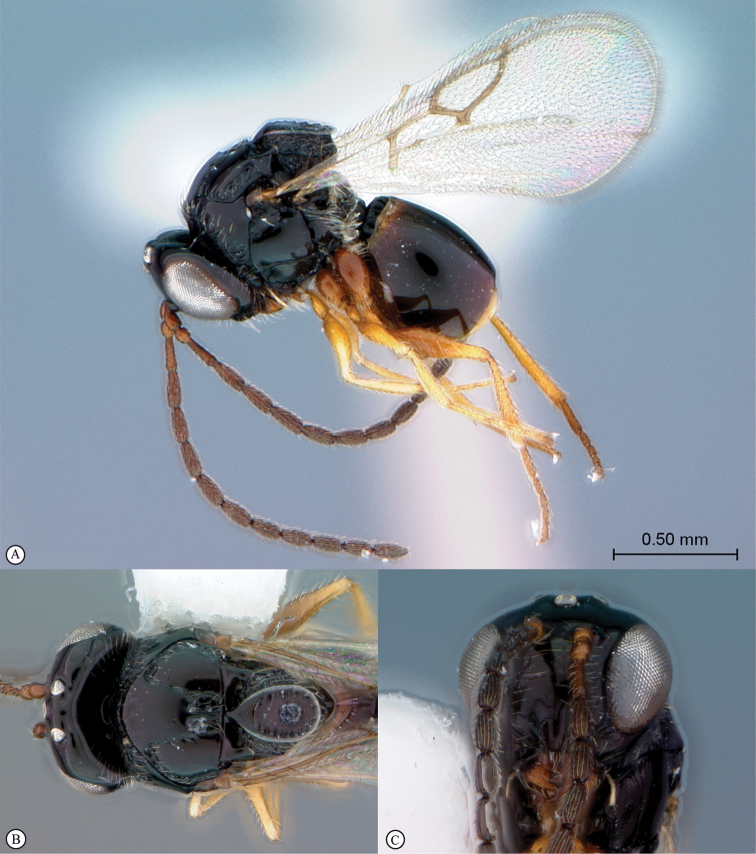
*Gronotoma* species (Kenya). **A** habitus lateral view **B** head and mesosoma dorsal view **C** head, anterior view.

####### Distribution.

Worldwide but mainly Holarctic. Afrotropical records: Cameroon, Madagascar, Seychelles, South Africa (Quinlan 1986), Kenya (here).

####### Biology.

Parasitoids of leaf-mining and stem-mining Agromyzidae (Buffington 2011).

####### Species richness.

(for all the proper species below, types have not been scrutinised, and it is uncertain whether they are true *Gronotoma* or *Afrostilba*.)

*Gronotoma extraria* (Quinlan, 1986) (*Eucoilidea*) (Madagascar)

*Gronotoma leptis* (Quinlan, 1986) (*Eucoilidea*) (Cameroon)

*Gronotoma mauri* (Quinlan, 1986) (*Eucoilidea*) (South Africa)

*Gronotoma parvula* Kieffer, 1910c (Madagascar)

*Gronotoma seychellensis* Kieffer, 1911a (Seychelles)

*Gronotoma* sp. (Madagascar, Kenya)

###### Nordlanderia

Taxon classificationAnimaliaHymenopteraFigitidae

Quinlan, 1986

####### Remarks.

Locally common in the Afrotropical region, particularly in arid regions.

####### Diagnosis.

Ventral margin of malar space and clypeus with distinct protuberances. Lateral pronotal carina present. Notauli faint, often only present anteriorly and posteriorly on mesoscutum. Ventral border of mesopleural triangle distinct, not rounded (at least posteriorly). Base of syntergum of metasoma glabrous. Most easily confused with *Tobiasiana* Kovalev (not an Afrotropical taxon), but differing by the conical protuberance on the ventral clypeal margin (spatulate in *Tobiasiana*). Within the Afrotropical region, *Nordlanderia* most closely resembles *Ealata* by having reduced notauli and a smaller scutellar plate; can be distinguished from *Ealata* by the presence of numerous tubercles along the rim of the scutellar plate (single central tooth in *Ealata*), and typically more complete notauli.

**Figure 22.**
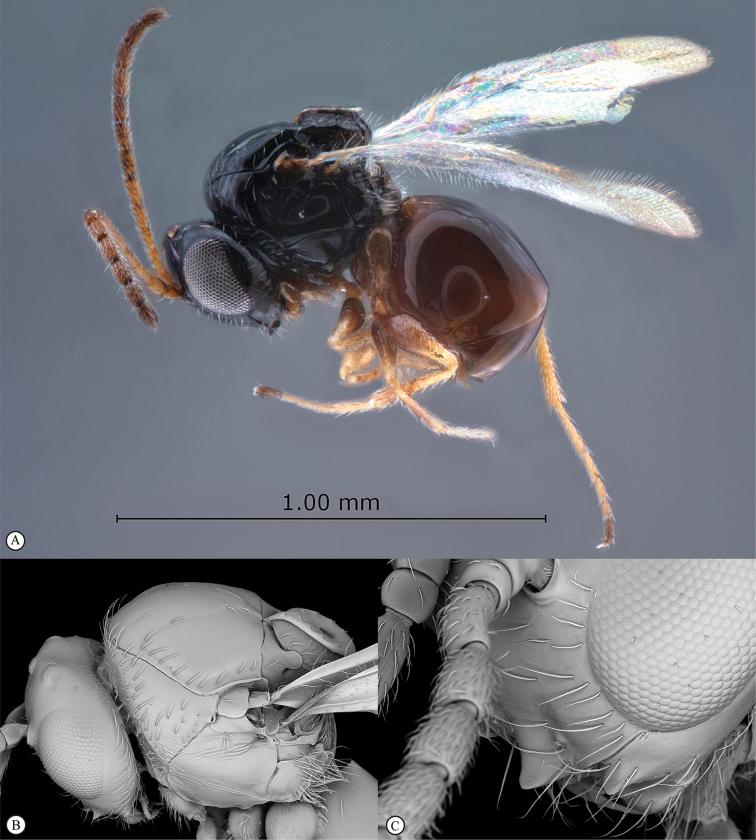
*Nordlanderia* species (South Africa). **A** habitus lateral view **B** head and mesosoma dorso-lateral view **C** head, anterior-lateral view.

####### Distribution.

Mainly Afrotropical, but extending to the Oriental region and the southern Palearctic. Afrotropical records: Comoros, Democratic Republic of Congo, Ghana, Namibia, Nigeria, South Africa, Zambia, Zimbabwe (Quinlan 1986), Botswana, Burkina Faso, Cameroon, Central African Republic, Gabon, Ivory Coast, Kenya, Madagascar, Mauritius, Mozambique, Niger, Senegal, Sierra Leone, Somalia, Tanzania, Uganda, Yemen (here). Particularly species-rich in South Africa.

####### Biology.

Parasitoids of leaf-mining Agromyzidae (Buffington 2011).

####### Species richness.

*Nordlanderia acis* Quinlan, 1986 (Namibia, South Africa)

*Nordlanderia pallida* Quinlan, 1986 (Ghana, South Africa)

*Nordlanderia phaedrae* Buffington, 2010 (Yemen)

*Nordlanderia plowa* Quinlan, 1986 (Comoros, Democratic Republic of Congo, Nigeria, Zambia, Zimbabwe)

Several undescribed species from South Africa.

###### Paradiglyphosema

Taxon classificationAnimaliaHymenopteraFigitidae

Lin, 1988

####### Remarks.

Rare.

####### Diagnosis.

Genal carina present. Lateral pronotal carina present (at least ventrally). Parascutal impression complete, with a distinct interruption anteriorly. Notauli present and well developed. Laterodorsal projections of scutellum present. Dorsal and ventral margins of mesopleural triangle distinct. This genus can be distinguished from all other Diglyphosematini by the presence of laterodorsal projections on the scutellum and a complete genal carina. These characters are only shared with some Zaeucoilini (found in New World tropics), but the latter group is not found in the Afrotropical or Oriental regions, nor do Zaeucoilini possess notauli (Buffington 2009).

**Figure 23.**
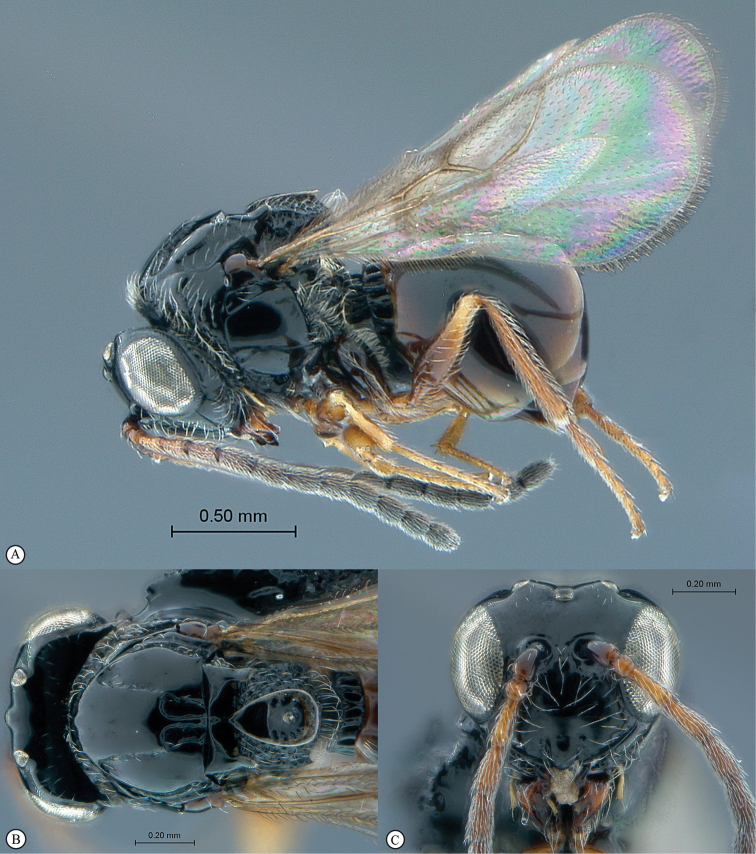
*Paradiglyphosema* species (Central African Republic). **A** habitus lateral view **B** head and mesosoma dorsal view **C** head, anterior view.

####### Distribution.

Mainly Oriental but also extending into equatorial Africa. Afrotropical records: Kenya, Somalia (Buffington 2011), Cameroon, Central African Republic, Uganda (here).

####### Biology.

Hosts not observed, but hypothesized to be leaf-mining Agromyzidae (Buffington 2011).

####### Species richness.

Two undescribed species: one in Cameroon, Central African Republic and Uganda, one in Kenya and Somalia.

#### Eucoilini

Current evidence suggests that Eucoilini is possibly not monophyletic. The tribe may be paraphyletic *visavis* the Trichoplastini and the two tribes may eventually have to be merged. It includes a majority of the larger-sized representatives of the subfamily.

##### Afrodontaspis

Taxon classificationAnimaliaHymenopteraFigitidae

Weld, 1961

###### Remarks.

Rare. *Afrodontaspis* was first characterised as superficially similar to the Neotropical *Trissodontaspis* Ashmead, 1903 (Weld 1962) and then Quinlan (1986) noted it had affinities with *Bothrochacis*. In fact *Afrodontaspis* shares most of the systematically important character states with *Bothrochacis*, and may possibly turn out to be an ingroup there (and accordingly a junior synonym); however, preliminary phylogenetic analyses (Baião and Forshage, unpublished) have not confirmed this, and we maintain it as a separate genus.

###### Diagnosis.

Large, black or reddish wasps with very little pubescence on body and wings; unmistakable in their heavy striation and scutellar spine, yet sharing most of the systematically informative characters with *Bothrochacis*. Like *Bothrochacis* with striate vertex, and like some *Bothrochacis* with vermiculate coxae and notched scutellum, but *Bothrochacis* always has many setae and no striation on the mesosoma. *Linoeucoila* are similar but smaller, without a scutellar spine, and with many setae on the mesosoma. The scutellar spine is thinner and more pointed than in *Trichoplasta*. General appearance may also approach members of Aspicerinae and Figitinae with striate sides and reduced wing pubescence, but these lack the characteristic eucoiline scutellum.

**Figure 24.**
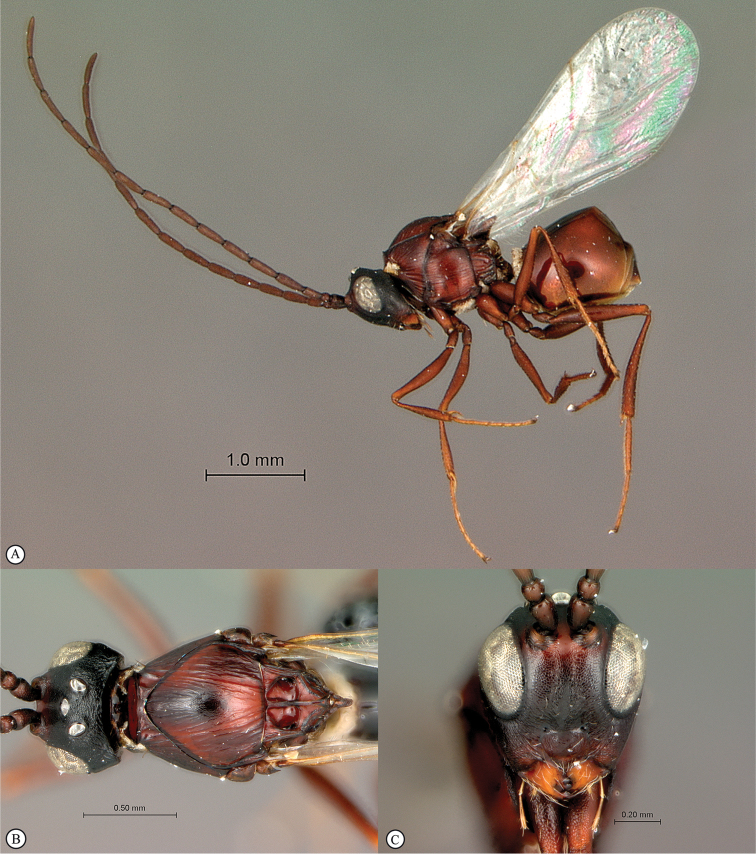
*Afrodontaspis striatissima* (Uganda). **A** habitus lateral view **B** head and mesosoma dorsal view **C** head, anterior view.

###### Distribution.

Endemic to the Afrotopical region: Burundi, Democratic Republic of Congo (Benoit 1956a), Kenya, Uganda (Quinlan 1986), Burkina Faso, Cameroon, Central African Republic, Gambia, Rwanda, South Africa, Tanzania, Zambia (here).

###### Biology.

Unknown, but like its close relatives, and like other species with glabrous wing membranes and striated body, it can be expected to attack dung-dwelling flies.

###### Species richness.

*Afrodontaspis lanata* Quinlan, 1986 (Democratic Republic of Congo, Kenya)

*Afrodontaspis striatissima* (Benoit, 1956a) (*Coneucoila*) (Burkina Faso, Burundi, Democratic Republic of Congo, Gambia, Kenya, Rwanda, Tanzania, Uganda)

##### Bothrochacis

Taxon classificationAnimaliaHymenopteraFigitidae

Cameron, 1904

Bothrochacis (synonyms *Ditrupaspis* Kieffer, 1910d, *Salpictes* Kieffer, 1913b, *Stirencoela* Cameron, 1910)

###### Remarks.

Not uncommon. *Bothrochacis* has been a small and easily recognisable genus since its original description. Quinlan’s Afrotropical *Eucoila* species have been known for some time to not fit into *Eucoila*, but nothing has been published about them since their original description. Here we note that they share all the important characteristics of *Bothrochacis* except the very peculiar notched scutellum, and include them here. Most of the characteristics of the genus in the wider sense are also found in the genera *Afrodontaspis* and *Linoeucoila*, but preliminary phylogenetic analyses (Baião and Forshage, unpubl.) have not supported their merging into one genus.

###### Diagnosis.

Most of the large and dark (black or distinctly bicolored) eucoilines in the Afrotropical region belong to *Bothrochacis*. They can be distinguished from the few others (*Afrodontaspis*, *Linoeucoila*, *Aganaspis*, *Trybliographa* and perhaps some *Trichoplasta*) by having plenty of large truncate setae on pronotum and mesoscutum, and stout setae on the subcosta of the wing. Like other genera of Eucoilini, they have subalar pits and glabrous oblique metapleural corners. They have a striate vertex, often have vermiculate-reticulate sculpture on the coxae and large scutellar foveae, usually strong reduction of wing pubescence, and sometimes a notched scutellum.

The core group are the “*erythropoda* species group”, coinciding with *Bothrochacis* sensu Quinlan, the species possessing a notched scutellar plate. These are large, typically bicolored, have hairless wings, smooth coxae, and large scutellar foveae.

Appearing like a less modified stem group there is the “*veleda* species group”, consisting of species described by Quinlan in *Eucoila*, typically of smaller size, with an evenly arched scutellar plate and smaller scutellar foveae, often with some pubescence on wings. Specimens belonging to this group are sometimes quite similar to *Trybliographa*, rare in the region, common elsewhere, but easily separated by the characters listed above.

**Figure 25.**
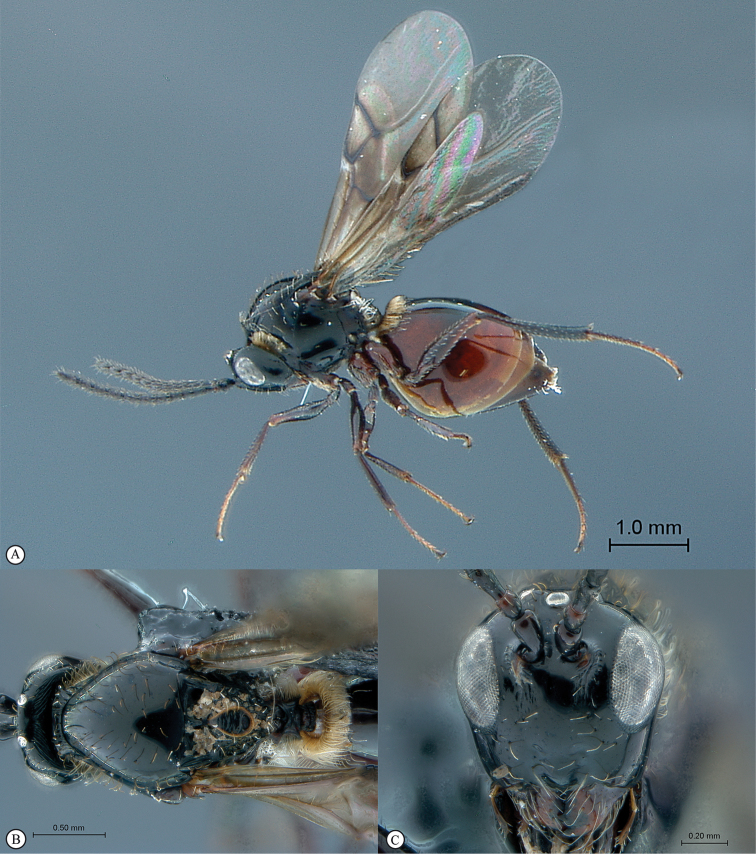
*Bothrochacis marina* (Uganda). **A** habitus lateral view **B** head and mesosoma dorsal view **C** head, anterior view.

###### Distribution.

Mainly Afrotropical but also occuring in the Oriental and Oceanic regions (hitherto unpublished). Afrotropical records: Kenya (Kieffer 1913b), Rwanda (Benoit 1956a), Ethiopia (Belizin 1973), Botswana, Democratic Republic of Congo, Malawi, Nigeria, South Africa, Uganda, Zimbabwe (Quinlan 1988), Burkina Faso, Cameroon, Central African Republic, Gabon, Gambia, Ivory Coast, Madagascar, Namibia, Somalia, Tanzania, Zambia (here).

###### Biology.

One species is recorded as attacking Muscidae in dung (Bridwell 1919), and due to phylogenetic inference as well as inference based on parallell occurrence of morphological particularities (elsewhere in the Eucoilinae as well as in the Figitinae) it seems reasonable to assume that most of them have similar habits.

###### Species richness.

***Bothrochacis erythropoda* species group**

*Bothrochacis erythropoda* Cameron, 1904 (Democratic Republic of Congo, Kenya, Malawi, South Africa, Tanzania)

syn *Ditrupaspis semirufa* Kieffer, 1910d

syn *Bothrochacis stercoraria* Bridwell, 1919

syn *Stirencoela striaticollis* Cameron, 1910

*Bothrochacis rufiventris* (Kieffer, 1913b) (*Salpictes*) (Kenya)

*Bothrochacis septenaria* Belizin, 1973 (Ethiopia)

*Bothrochacis serratepilosa* Benoit, 1956a (Rwanda)

***Bothrochacis veleda* species group**

*Bothrochacis bantia* (Quinlan, 1988), **comb. n.** (*Eucoila*) Type in BMNH studied by MF (Botswana, Democratic Republic of Congo, Gambia, Kenya, Madagascar, South Africa, Zimbabwe)

*Bothrochacis erinna* (Quinlan, 1988), **comb. n.** (*Eucoila*) Type in RMCA studied by MB (Democratic Republic of Congo, Kenya)

*Bothrochacis marina* (Quinlan, 1988), **comb. n.** (*Eucoila*) Type in BMNH studied by MF (Burkina Faso, Democratic Republic of Congo, Kenya, Namibia, South Africa, Zimbabwe)

*Bothrochacis veleda* (Quinlan, 1988), **comb. n.** (*Eucoila*) Type in BMNH studied by MF (Botswana, Burkina Faso, Democratic Republic of Congo, Kenya, Nigeria, South Africa, Tanzania, Zimbabwe)

##### Leptopilina

Taxon classificationAnimaliaHymenopteraFigitidae

Förster, 1869

###### Remarks.

Very common throughout Africa.

###### Diagnosis.

Usually medium-sized and rather stout eucoilines, with a more or less reduced hairy ring. Often superficially similar to *Ganaspis*. Easily separated from the former in males, since *Leptopilina* males have antennal F1 hardly modified and F2 moderately modified, while *Ganaspis* have F1 distinctly modified and F2 not at all; but for females (or males with antennae not visible) it is important to examine the metapleural corner: in *Leptopilina* it is oblique and hairless, in *Ganaspis* rectangular and hairy. Many *Leptopilina* which have a narrow marginal cell may be confused with *Rhoptromeris*, which have the male F2 more strongly modified and only rarely have a reduced hairy ring, and which are easy to separate by having lateral bridges on the pronotum (open in *Leptopilina*, closed in *Rhoptromeris*).

Species groups have been suggested by Nordlander (1980) and Allemand et al. (2002), but these have little consequence for the identification of the genus as such.

**Figure 26.**
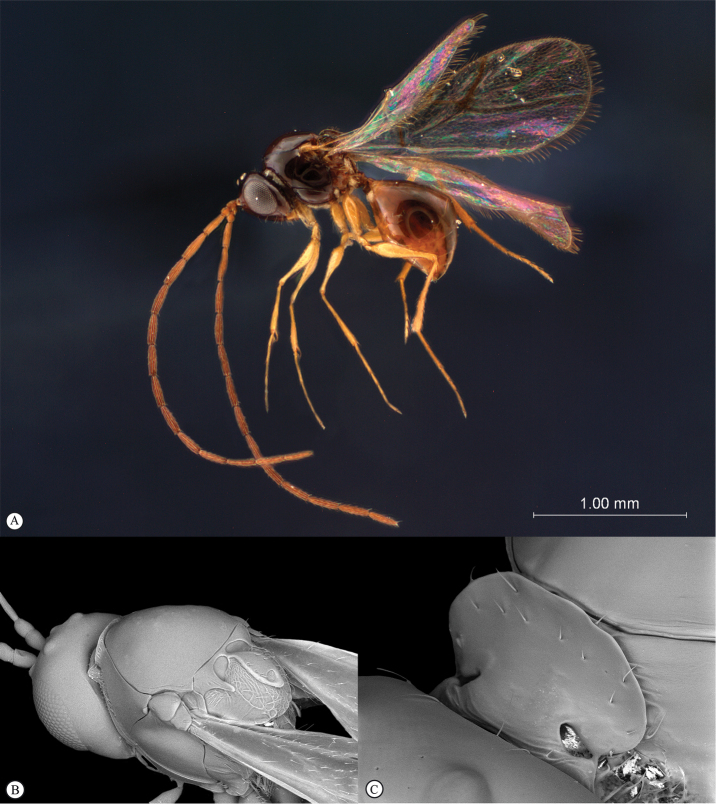
*Leptopilina* species (Kenya). **A** habitus lateral view **B** head and mesosoma dorso-lateral view **C** pronotal plate.

###### Distribution.

Worldwide. Afrotropical records: Cameroon, Democratic Republic of Congo, Madagascar, Nigeria, Seychelles, South Africa, Uganda, Zambia, Zimbabwe (Quinlan 1986), St Helena (Notton 2008), Benin, Comoros, Gambia, Ivory Coast, Kenya, Mauritius, Republic of Congo, Reunion, Sao Tomé (Allemand et al. 2002), Botswana, Burkina Faso, Burundi, Central African Republic, Ethiopia, Gabon, Ghana, Malawi, Rwanda, Tanzania, Yemen (here).

###### Biology.

Well-known for attacking Drosophilidae in various habitats (van Alphen et al. 1991, Buffington 2007; Buffington et al. 2012, Lee et al. 2009, Novkovic et al. 2011), including in Africa (Allemand et al. 2002, Nordlander 1980).

###### Species richness.

*Leptopilina africana* (Kieffer, 1911b), **comb. n.** (*Eucoila*) (replacement name for *Eucoila afra* Kieffer, 1910d nec Kieffer, 1904) Type ZMBH seen by MF (Rwanda)

*Leptopilina apella* Quinlan, 1988 (Democratic Republic of Congo, Madagascar)

*Leptopilina atraticeps* (Kieffer, 1911b) (*Ectolyta*) (Nigeria, Seychelles)

*Leptopilina boulardi* (Barbotin, Carton & Kelner-Pillault, 1979) (*Cothonaspis*) (Democratic Republic of Congo, Ivory Coast, Kenya, Madagascar, Republic of Congo, Seychelles, South Africa, Zambia, Zimbabwe; worldwide distributed species)

syn *Charips mahensis* Kieffer, 1911c, a secondary homonym in *Leptopilina*

*Leptopilina cavernicola* (Kieffer, 1913b), **comb. n.** (*Eucoila*) Type not found, apparently missing in MNHN, placement tentative based on original description (Tanzania)

*Leptopilina drosophilae* (Kieffer, 1913c), **comb. n.** (*Eucoila*) Type not located (possibly in coll Silvestri), placement based on original description (Guinea)

*Leptopilina dulcis* (Quinlan, 1988), **comb. n.** (*Cothonaspis*) (Democratic Republic of Congo, Madagascar)

*Leptopilina fannius* Quinlan, 1988 (Democratic Republic of Congo)

*Leptopilina faunus* Quinlan, 1988 (Democratic Republic of Congo)

*Leptopilina fenerivae* (Kieffer, 1910c), **comb. n.** (*Psilosema*) Type in ZMUH seen in 1980s by Göran Nordlander and generic placement assessed by him (pers. comm.), but not found upon more recent enquiry (Ralph Peters pers. comm.) (Madagascar)

*Leptopilina fimbriata* (Kieffer, 1901a) (*Eucoela*) (Palearctic species, also recorded from Afrotropical region)

*Leptopilina freyae* Allemand & Nordlander, 2002 (Benin, Gambia, Kenya)

*Leptopilina guineaensis* Allemand & Nordlander, 2002 (Benin, Cameroon, Republic of Congo, Reunion, South Africa, Sao Tomé)

*Leptopilina heterotoma* (Thomson, 1862) (*Eucoila*) (Democratic Republic of Congo, Madagascar, St Helena; worldwide distributed species)

syn *Pseudeucoila bochei* Weld, 1944

*Leptopilina itys* Quinlan, 1988 (Democratic Republic of Congo, Zimbabwe)

*Leptopilina mahensis* (Kieffer, 1911c) (*Erisphagia*) (Seychelles)

*Leptopilina misensus* Quinlan, 1988 (Democratic Republic of Congo, Uganda)

*Leptopilina orientalis* Allemand & Nordlander, 2002 (Comoros, Madagascar, Reunion)

*Leptopilina pisonis* Quinlan, 1988 (Democratic Republic of Congo)

*Leptopilina syphax* Quinlan, 1988 (Democratic Republic of Congo)

*Leptopilina thetus* Quinlan, 1988 (Democratic Republic of Congo, South Africa)

*Leptopilina vesta* Quinlan, 1988 (Cameroon, Democratic Republic of Congo)

*Leptopilina victoriae* Nordlander, 1980 (Democratic Republic of Congo, Ivory Coast, Madagascar, Mauritius, Seychelles, South Africa, Uganda)

##### Linoeucoila

Taxon classificationAnimaliaHymenopteraFigitidae

Lin, 1988

###### Remarks.

Rare in the Afrotropical region. *Linoeucoila* was first described with a number of species from Taiwan (Lin 1988), and considered close to *Trybliographa*, mainly differing by their aciculate, vermiculate or striate body sculpture. The name has only been used later when the genus was included in the tribe Eucoilini (Forshage et al. 2007). Type specimens have not been available for loan, but specimens from Taiwan corresponding to the original description of the genus (mostly undescribed species) have been studied, and it has been considered that this is a taxon present throughout the Oriental region and also present in Africa. However, it has been difficult to decide how to circumscribe the genus or to separate it from *Trybliographa* except by the body sculpture, and it may well turn out to be one or several lineages within *Trybliographa*. Despite conforming to the hitherto known diagnostic characters of *Linoeucoila*, African specimens have some differences from the Oriental species, and may eventually have to form a genus of their own.

###### Diagnosis.

Large and dark (bicolored) eucoilines, most similar to *Bothrochacis*, but lacking the characteristic subcostal setae and pentiful truncate mesosomal setae of that genus, and having striation on sides of mesosoma. Striation of vertex is reticulate or lateral, not radiate as in *Bothrochacis*. Very similar to *Trybliographa* except for the sculpture of the integument. With subalar pits, an oblique and glabrous metapleural corner, more or less reduced wing pubescence, large scutellar foveae, and vermiculate sculpture on coxae.

**Figure 27.**
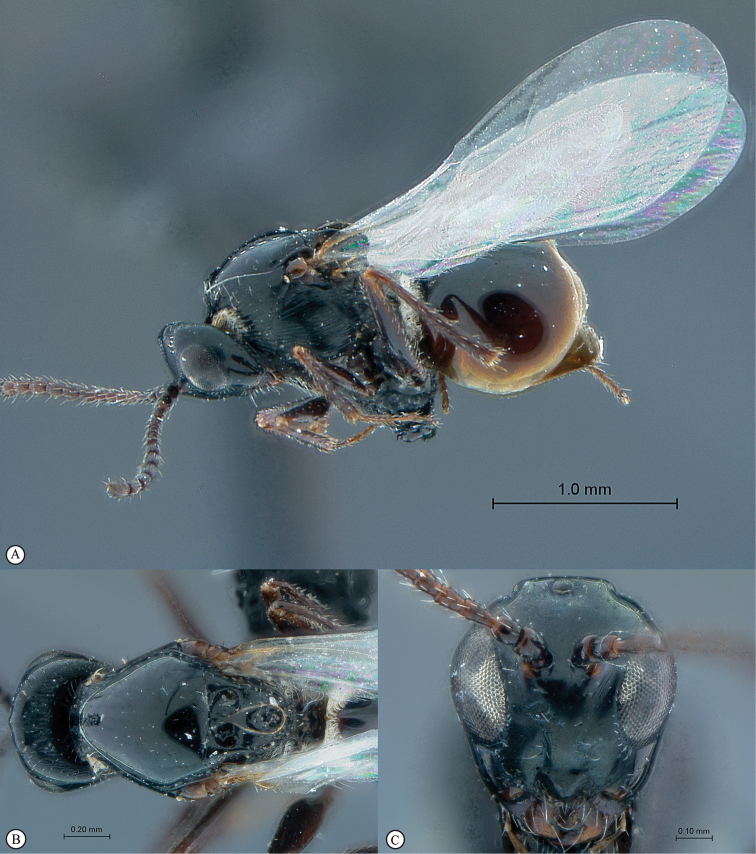
*Linoeucoila* species (South Africa). **A** habitus lateral view **B** head and mesosoma dorsal view **C** head, anterior view.

###### Distribution.

Mainly Oriental, but also occuring in the Afrotropical region (hitherto unpublished). Afrotropical records: South Africa, Uganda (here).

###### Biology.

Unknown, but many close relatives, and many figitids with striate sides and reduced wing pubescence are parasitoids of dung-breeding flies.

###### Species richness.

Undescribed species only.

##### Trybliographa

Taxon classificationAnimaliaHymenopteraFigitidae

Förster, 1869

###### Remarks.

Rare in the Afrotropical region. Only one species encountered so far, but more are to be expected.

###### Diagnosis.

Characteristically large eucoilines, dark and mostly strongly built, separated from several other genera with which confusion is otherwise possible by the possession of distinct subalar pit, as well as a metapleural corner which is hairless, oblique, and more or less upturned (forming a small, more or less triangular glabrous posterolateral surface called the metapleural triangle). *Aganaspis* which is often superficially similar always has a distinct tuft of hairs on the metapleural corner. Furthermore, in males, *Aganaspis* just like other Ganaspini have the antennal F1 modified, while *Trybliographa* have F2 modified. In Africa, the major confusion risk is in fact those specimens of the closely related *Bothrochacis* that have less reduced wing pubescence. Unlike *Trybliographa*, they typically have stout setae on the subcosta, truncate setae on the pronotum and mesoscutum, large scutellar foveae, and vermiculate sculpture on the coxae. The single *Trybliographa* species encountered so far in the Afrotropical region though, is very easy to recognise by the fuscate marginal cell.

**Figure 28.**
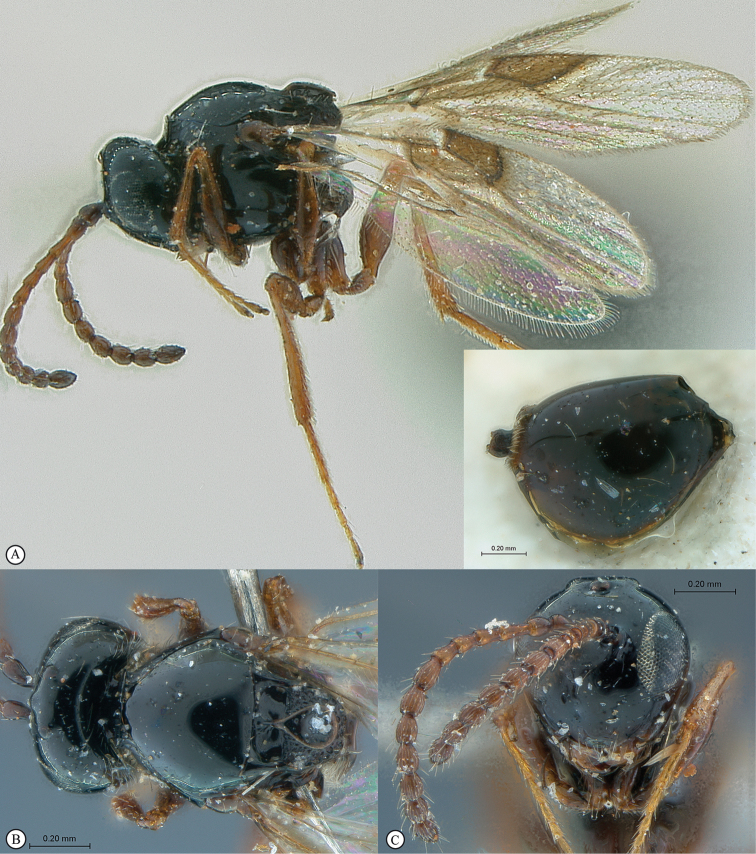
*Trybliographa australiensis* (Madagascar). **A** habitus lateral view **B** head and mesosoma dorsal view **C** head, anterior view.

###### Distribution.

Worldwide, but by far most diverse in the Holarctic. Afrotropical region: Madagascar (here).

###### Biology.

Attack Anthomyiidae and occasionally other calyptrate flies in dung, fungi, debris, etc (Belizin 1963, Block et al. 1987, Griffiths 1993, Harukawa and Kikamashiro 1939, Hemachandra et al. 2007, Hennig 1976, Hummel et al. 2010, Jones and Hassell 2008, Kerrich and Quinlan 1960, Kieffer 1913a, Makarenko 1968, Neveu et al. 1996, 2000, Nilsson et al. 2011, 2012, Nordlander 1981, Quinlan 1978, Tamer 1994, Wilkes and Wishart 1953, Wishart and Monteith 1954, and plenty of others, plus lots of additional label data). The host of the African species is not known.

###### Species richness.

*Trybliographa australiensis* Ashmead, 1900 (Madagascar)

#### Ganaspini

This is the largest and most difficult tribe of Eucoilinae. The generic limits between the major genera are not clear and a small selection of autapomorphic forms currently have generic status which could just as well be moved into the major genera. We have considered a major generic-level revision of this group beyond the scope of this work, and probably impossible without a thorough phylogenetic analysis.

##### Aganaspis

Taxon classificationAnimaliaHymenopteraFigitidae

Lin, 1987

###### Remarks.

Rare. *Aganaspis* has been widely confused with *Trybliographa*; superficially strikingly similar morphologically, but not closely related (Fontal-Cazalla et al. 2002, Buffington et al. 2007). On the other hand, it remains uncertain how to delineate *Aganaspis* from *Ganaspis*.

###### Diagnosis.

Large, strongly built, black or darkly brown eucoilines. The genus was originally erected (Lin 1987) for Oriental species with a very large scutellar plate reaching the posterior end of the scutellum, and a high pronotal plate with an emarginate (bilobed) dorsal rim protruding well over the pronotal-mesoscutal suture. But the exploration of apparently closely related forms, especially in South America, has made the genus far more difficult to circumscribe in terms of unambiguous diagnostic characters, and indeed, blurred the boundaries towards the heterogenous *Ganaspis*. Most *Aganaspis*, however, are large and resemble *Trybliographa* in general habitus, but are easy to distinguish from the latter based on the distinct hairtuft on the metapleural corner, the small and shallow subalar pit, and the modified antennal F1 in males. The posterior metapleural margin is uninterrupted (but sometimes depressed in the middle) and usually somewhat oblique in the ventral part; the metacoxae usually have semi-long hairlines (but sometimes only small tufts); the sctutellar plate is usually very large and the scutellar foveae usually large (but sometimes far more normal sized).

**Figure 29.**
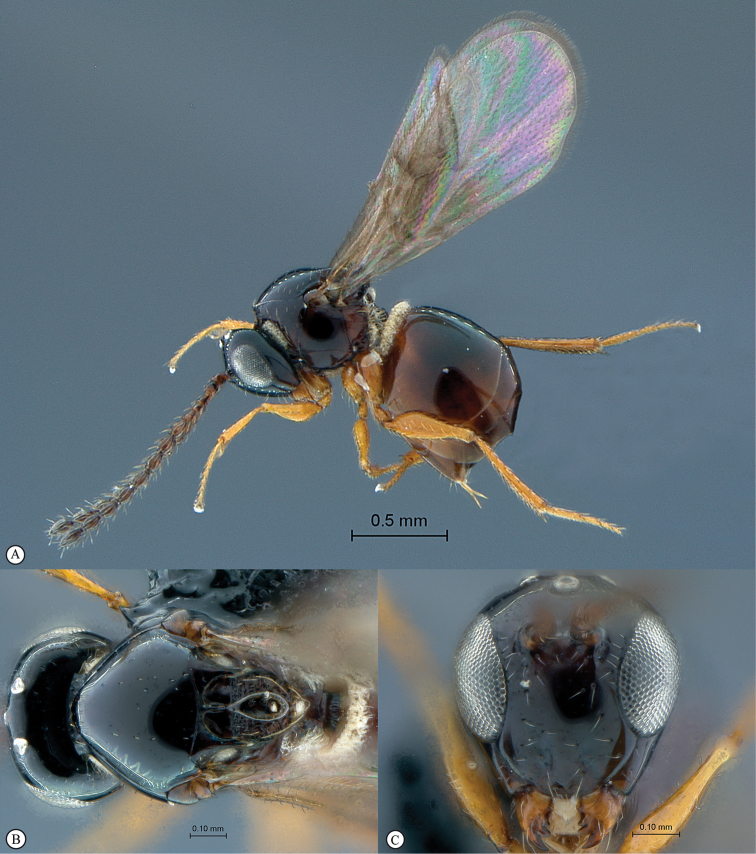
*Aganaspis* species (Central African Republic). **A** habitus lateral view **B** head and mesosoma dorsal view **C** head, anterior view.

###### Distribution.

Widespread, but primarily East Asian and Neotropical. Afrotropical records: Central African Republic, Democratic Republic of Congo, Kenya, Reunion, South Africa, Tanzania (here).

###### Biology.

Attacks Tephritidae and other fruit-infesting flies (Wharton et al. 1998, Guimarães et al. 2003).

###### Species richness.

*Aganaspis daci* (Weld, 1951) (*Trybliographa*) (widely distributed species, synanthropically spread with an assumed origin in the Oriental region).

Several undescribed species in the region.

##### Didyctium

Taxon classificationAnimaliaHymenopteraFigitidae

Riley, 1879

###### Remarks.

Among the most common genera of eucoilines in the Afrotropical region (yet not recorded by Quinlan). Not always certainly distinguished from *Ganaspis*, *Hexacola* and *Endecameris*.

###### Diagnosis.

*Didyctium* are usually recognisable through the combination of a concave scutellar plate and the characteristically half-open marginal cell. Furthermore, unlike in typical representatives of the closely related *Ganaspis*, the head is transverse (not deep), the mesosoma is short (propodeum not extended), the coxae bear small hair tufts (not elongate hairlines), the scutellar plate is relatively small, the lateral bars of the scutellum are striate, and the posterior margin of the metapleuron is straight (not with a circular or elongate incision). However, the diverse *Ganaspis* may vary in all these traits. *Didyctium* females usually have very unusual antennae, where the flagellomeres are strongly differentiated into very short annelli and very long club articles. Very small specimens of *Didyctium* approach the character states of *Endecameris*, and the boundary between the two genera is uncertain.

**Figure 30.**
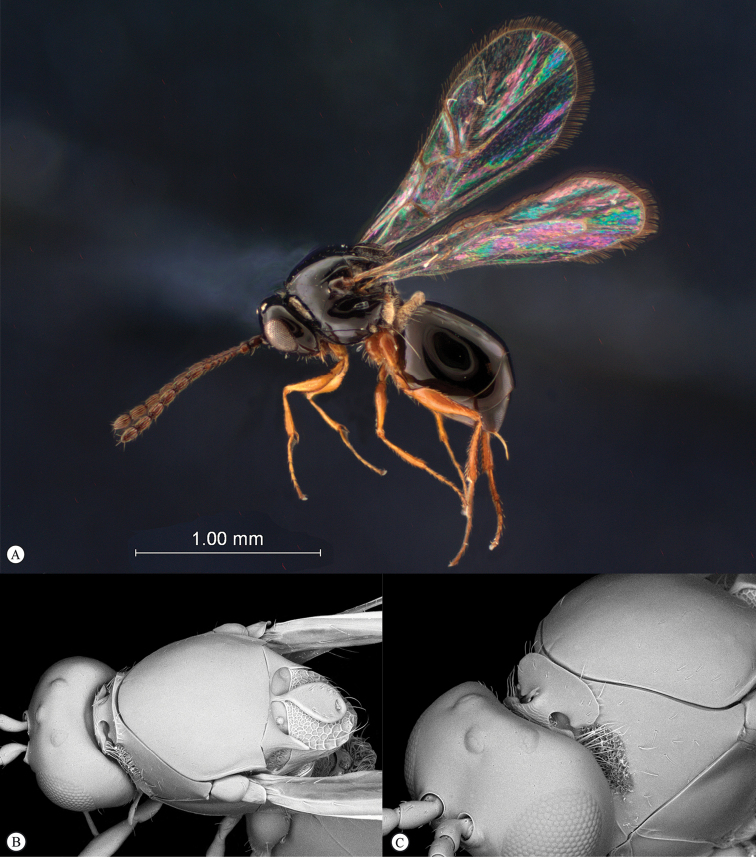
*Didyctium* species (Kenya). **A** habitus lateral view **B** head and mesosoma dorsal view **C** propodeal plate anterior dorsal view.

###### Distribution.

Worldwide. Afrotropical records: Burkina Faso, Cameroon, Central African Republic, Comoros, Gabon, Gambia, Ghana, Ivory Coast, Kenya, Madagascar, Republic of Congo, Sierra Leone, South Africa, Tanzania, Uganda, Zambia, Zimbabwe (here).

###### Biology.

Attacks Phoridae and other flies in concealed habitats (Beardsley 1989, Riley 1879, label data); no host records are from Africa.

###### Species richness.

*Didyctium naivashae* (Kieffer, 1913b), **comb. n.** (*Cothonaspis*) Type in MNHN studied by MF (Kenya, Rwanda)

Numerous African species remain to be described.

##### Endecameris

Taxon classificationAnimaliaHymenopteraFigitidae

Yoshimoto, 1963

###### Remarks.

Rare.

###### Diagnosis.

Tiny eucoilines with round heads, short antennae and characteristically modified wings: narrow triangular wings with very long hair fringe, and a short wide open marginal cell. A similar habitus and similar wings may occur in other “dwarfified” eucoilines of extremely small size – the rare *Micreriodes* and some species of *Rhoptromeris*. The combination of a pronotal plate with laterally open foveae and presence of a mesopleural line separates *Endecameris* from *Micreriodes* and *Rhoptromeris*. In *Endecameris*, the scutellar plate is narrow, there is often a reduction in wing pubescence, and there is often a remarkable reduction of the number of antennomeres (the latter not yet observed in African specimens). The antennae of female *Endecameris* are similar to those of *Didyctium*, with a strong differentiation of the flagellomeres into proximal very short articles (more or less annelli) and distal elongate club articles with distinct white rhinaria.

**Figure 31.**
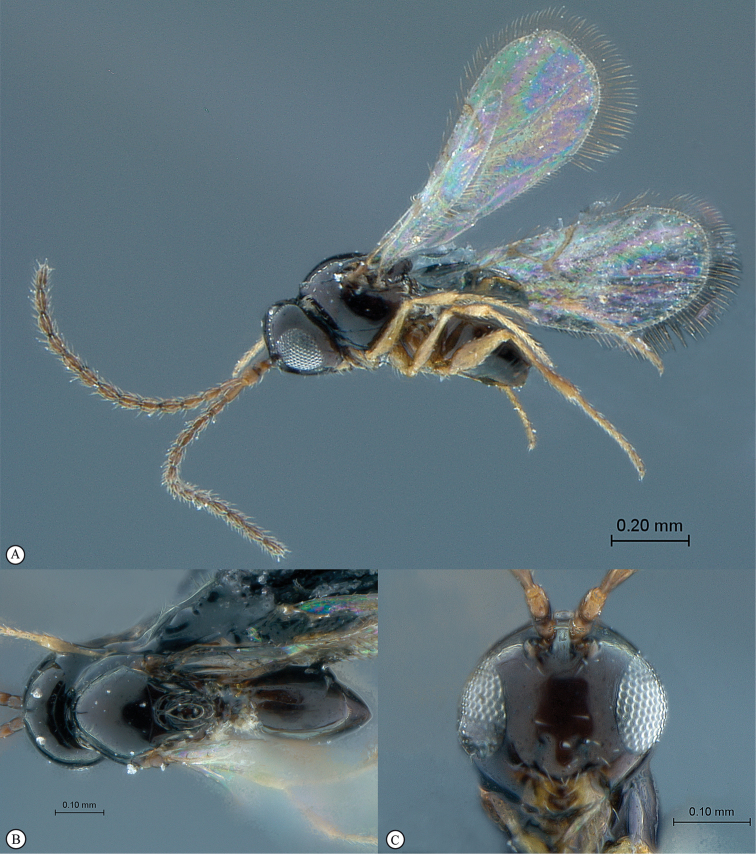
*Endecameris* species (Central African Republic). **A** habitus lateral view **B** head and mesosoma dorsal view **C** head, anterior view.

###### Distribution.

Mainly in the Oriental and Oceanic regions, but also occuring in the eastern Palearctic and in the Afrotropical region. Afrotropical records: Cameroon, Central African Republic, Gambia, Guinea-Bissau, Madagascar, Mauritius, Sierra Leone, South Africa, Uganda, Yemen, Zimbabwe (here).

###### Biology.

Host unknown.

###### Species richness.

Several species that are probably undescribed are present in Africa.

##### Ganaspis

Taxon classificationAnimaliaHymenopteraFigitidae

Förster, 1869

###### Remarks.

Common in Africa as elsewhere, yet only recorded in the key to genera in Quinlan (1986). The genus is very difficult to circumscribe, not always certainly distinguished from *Aganaspis*, *Didyctium* and *Hexacola*. At the same time, many smaller genera of the Ganaspini may actually be ingroups in the genus as currently conceived. Thus, a thorough analysis of the boundaries of this genus will most likely highlight the need to either synonymise a number of apomorphic small genera back into *Ganaspis*, or to recognise species groups of *Ganaspis* as separate genera. However, the difficulty to circumscribe the latter and to delineate them from the other major genera of Ganaspini suggests that this cannot be done without a thorough phylogenetic analysis. Of the several genera that Lin (1988) described in his revision of Taiwanese eucoilines, we have recognised *Gastraspis* (easily recognisable at least in females), but we have not been able to establish whether the plentiful African *Ganaspis* with very wide scutellar plates could be assigned to his *Epochresta*, and thus we have not treated the latter as an African genus.

###### Diagnosis.

*Ganaspis* is a vast and rather morphologically heterogenous genus, and can be regarded as currently comprising all of the typical Ganaspini that are not “different” enough to warrant a genus of their own. Typical *Ganaspis* are small, somewhat pale, compactly built eucoilines. Other characteristics include: a deep, more or less globular, head; a rather elongate mesosoma; more or less long hairlines on meso- and metacoxae; scutellum with a foveolate dorsal surface; a large, flat or convex scutellar plate; smooth lateral bars; a posterior metapleural margin with a circular or elongate excision; a narrow but distinct petiolar rim; broad wings with a rather truncate or faintly excised apex; and a deep fore wing marginal cell with curved sides – but most or all of these characters may vary within the genus. The typical *Ganaspis* are very often superficially similar to *Leptopilina* (also common parasitoids of Drosophilidae), but usually rather easily separated from them by having a modifed F1 in male antennae, and a distinct hair tuft on the metapleural corner. In order to facilitate recognition and sorting of *Ganaspis* in the Afrotropical region, we recognise the following morphological types of *Ganaspis* as characteristic within the Afrotropical fauna.

The apparently most common appearance of *Ganaspis* is one rather close to the European type, with long coxal hairlines, a circular or elongate excision of posterior pronotal margin, a wide scutellar plate not reaching the posterior end of scutellum, rather pale colour (middle brown body and yellow or pale brown legs). In some species, including most of the African taxa there are distinct patches of dense white pubescence on the pronotum and axillulae; marginal cells of wings are relatively homogenous with stong dominance for a short deep closed type with curved sides; some forms have a striking tooth on the metapleural edge, distinctly bi- or tricolored antennae, or large scutellar foveae.

A similar morphological type can also be seen in forms with an even larger scutellar plate, that is distinctly convex, but with a posterior depression and often an elongate-oval glandular release pit. These species are often relatively large in size. They may coincide with Lin’s (1988) genus *Epochresta* (but types in TARI have not been available for loan).

Occasional specimens, usually of small size, lack the coxal hairlines and have only short hair tufts.

Many undescribed species, especially from Madagascar, are rather large and remarkably slender-elongate in build and pale in color, somewhat approaching the habitus of *Chrestosema* and related genera. Occasionally these may have very unusual features such as an elongated petiolar rim. Some of these are similar in appearance to the characteristic Neotropical *Ganaspis* “*neotropica*-group”.

Other morphological types conform to the most common type in most respects, but differ, for example, in scutellar morphology. Some tiny specimens resemble *Endecameris* or *Didyctium*, others *Hexacola*, many *Aganaspis*.

**Figure 32.**
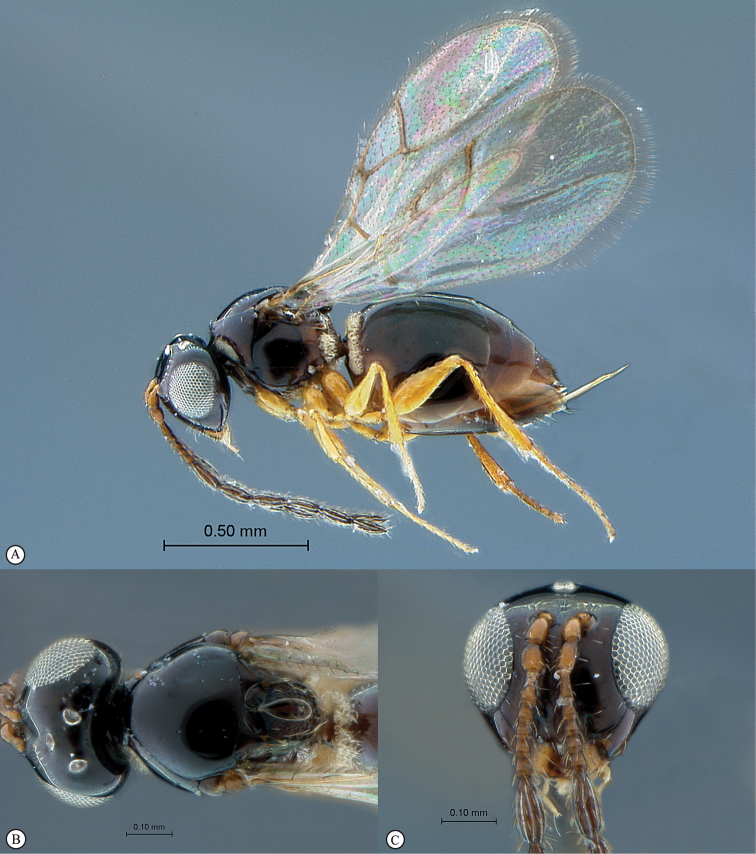
*Ganaspis* species (Central African Republic). **A** habitus lateral view **B** head and mesosoma dorsal view **C** head, anterior view.

###### Distribution.

Worldwide. Afrotropical records: Seychelles, Tanzania (Kieffer 1911, 1913), Rwanda (Benoit 1956a), Burkina Faso, Cameroon, Cape Verde, Central African Republic, Democratic Republic of Congo, Gabon, Gambia, Ivory Coast, Kenya, Madagascar, Mauritius, Nigeria, Sierra Leone, South Africa, Uganda, Zambia, Zimbabwe, Yemen (here).

###### Biology.

Usually attacking Drosophilidae in various habitats, but sometimes also fruit-infesting Tephritidae or other flies (Nordlander and Grijpma 1991, Melk and Govind 1999, Vass and Nappi 2000). None of the host records are from Africa.

###### Species richness.

*Ganaspis kilimandjaroi* (Kieffer, 1913b), **comb. n.** (*Eucoila*) Type MNHN studied by MF (Tanzania)

*Ganaspis mahensis* Kieffer, 1911c (Seychelles)

*Ganaspis ruandana* (Benoit, 1956a) (*Pseudeucoila*) (Rwanda)

*Ganaspis xanthopoda* (Ashmead, 1896) (*Trybliographa*) (worldwide distributed species)

Several species remain to be described in Africa.

##### Gastraspis

Taxon classificationAnimaliaHymenopteraFigitidae

Lin, 1988

###### Remarks.

Rare. Possibly congeneric with *Ganaspis*.

###### Diagnosis.

Small *Ganaspis*-type eucoilines most easily characterised by the elongate female metasoma. These specimens also show the following characteristics: short coxal hair tuft; a rather large, flat scutellar plate and relatively large scutellar foveae; metapleural margin with an elongate excision; wing with a very slightly emarginate apex and an elongate marginal cell. Associated males are very difficult to discern within the range of variation present in *Ganaspis*.

**Figure 33.**
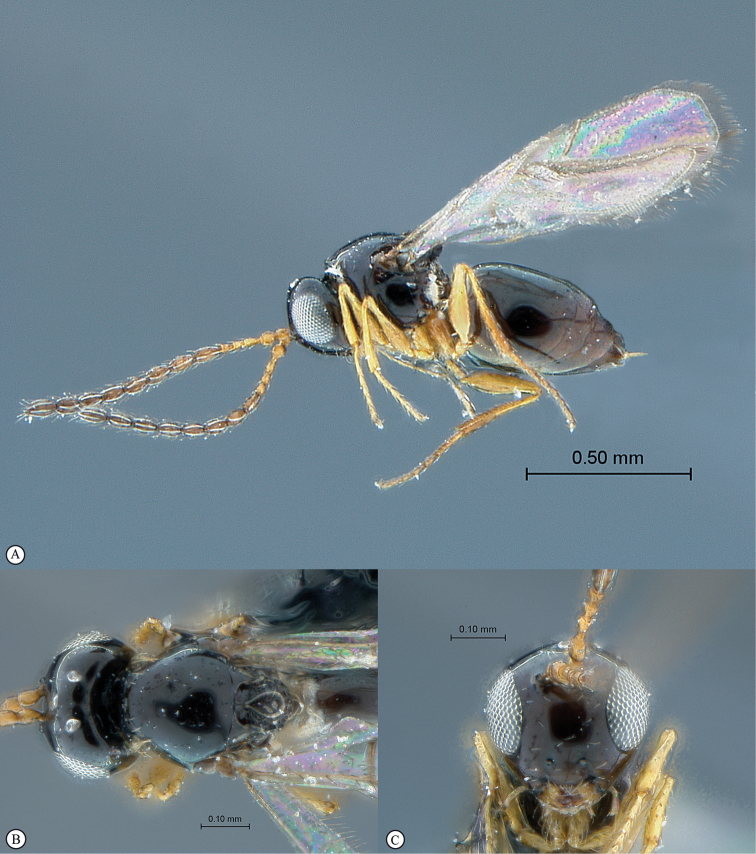
*Gastraspis* species (Central African Republic). **A** habitus lateral view **B** head and mesosoma dorsal view **C** head, anterior view.

###### Distribution.

Described from Taiwan, but here reported also from Africa. Afrotropical records: Central African Republic, Kenya (here).

###### Biology.

Hosts unknown.

###### Species richness.

Undescribed species in the region.

##### Hexacola

Taxon classificationAnimaliaHymenopteraFigitidae

Förster, 1869

Hexacola (synonym *Daruna* Benoit, 1956a, **syn. n.**)

###### Remarks.

Common throughout the Afrotropical region. Not always easily distinguished from *Ganaspis* and *Didyctium*. *Daruna* was erected by Benoit (1956a) for a specimen from Ruanda (the generic name being an anagram thereof), which, according to the original description, had dual glandular release pits of the scutellar plate, both the common posterior pit, and the central pit characteristic of most Diglyphosematini. Inspection of the holotype (in RMCA) by MB and MF revealed only the normal posterior glandular pit though, and nothing separates *Daruna* from a normal *Hexacola*. Linguistic gender of *Hexacola* is, according to etymology in the original description, neuter, but many species names have been given in the feminine previously.

###### Diagnosis.

*Hexacola* are usually very characteristic habitus-wise, being rather elongate eucolines with a characteristically globular head; a characteristic wing which is rather narrow with a narrow, closed, mostly triangular marginal cell; a very characteristic scutellum which is convex in its entirety; a very wide, convex, scutellar plate covering most of the surface; mostly striate sculpture on the narrow surrounding dorsal surfaces of the scutellum; and narrow, oblique scutellar foveae. Certain forms, however appear intermediate between the typical *Hexacola* and *Dicyctium* or *Ganaspis*. Occasionally the general bodyshape as well as scutellar striae of *Hexacola* may cause confusion with *Kleidotoma*, though the wings and metapleura are very different, or with *Rhoptromeris*, which can always be recognised by the pronotal plate with closed lateral bridges (and modified F2 in males).

**Figure 34.**
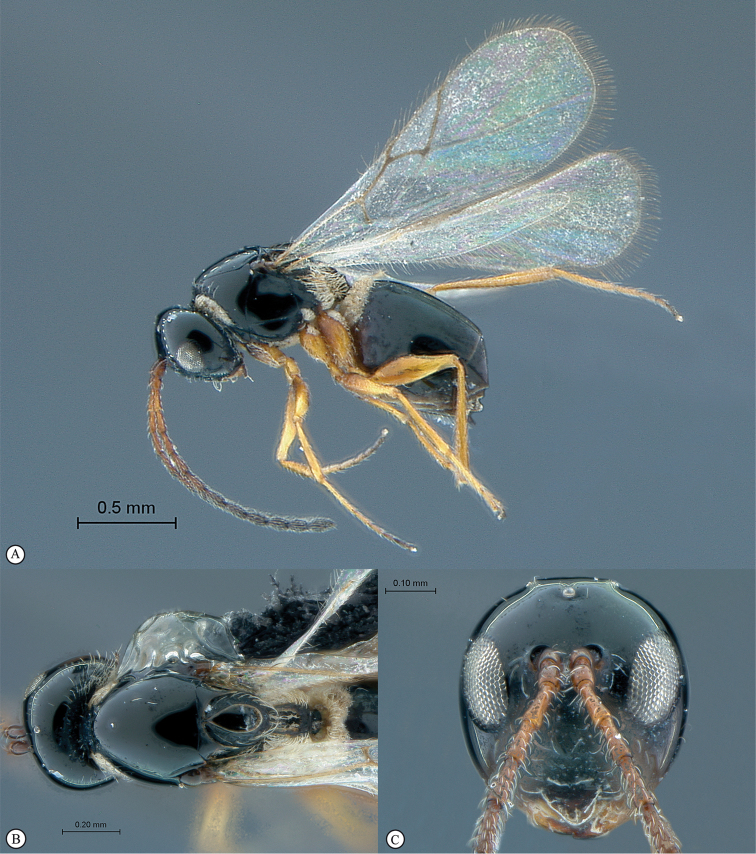
*Hexacola* species (South Africa). **A** habitus lateral view **B** head and mesosoma dorsal view **C** head, anterior view.

###### Distribution.

Worldwide. Afrotropical records: Rwanda (Benoit 1956a), Cameroon, Democratic Republic of Congo, Madagascar, Nigeria, South Africa, Uganda, Zimbabwe (Quinlan 1986), St Helena (Notton 2008), Burkina Faso, Central African Republic, Ethiopia, Gambia, Ivory Coast, Kenya, Malawi, Mauritius, Republic of Congo, Sierra Leone, Tanzania, Yemen (here).

###### Biology.

Reported from many fly hosts and habitats worldwide, commonly Chloropidae and Ephydridae in agricultural or wet habitats (Allen and Pienkowski 1973, Beardsley 1989, Bhattacharyya 1957, Diamond et al. 2001, Díaz and Gallardo 2010, Gaddi et al. 2010, Kerrich and Quinlan 1960, Moore 1983, Nordlander and Grijpma 1991, Quinlan 1978, Simmonds 1952, Streams and Greenberg 1969, and others). African taxa so far only reared from Chloropidae (label data).

###### Species richness.

*Hexacola absensa* Quinlan, 1986 (Democratic Republic of Congo)

*Hexacola amantia* Quinlan, 1986 (Nigeria, Uganda, Democratic Republic of Congo)

*Hexacola atropos* Quinlan, 1986 (Cameroon, Democratic Republic of Congo)

*Hexacola bifarium* Quinlan, 1986 (Nigeria, Democratic Republic of Congo, Zimbabwe)

*Hexacola camerounensis* (Risbec, 1956), **comb. n.** (*Eucoila*) (Cameroon) Type specimen expected to be in the MNHN, but not yet located. Original description does not state any very informative characters, but based on the illustration, *Hexacola* seems like a probable option until type material surfaces.

*Hexacola compactum* Quinlan, 1986 (Democratic Republic of Congo)

*Hexacola fringa* Quinlan, 1986 (Zimbabwe)

*Hexacola hexatoma* (Hartig, 1841) (*Cothonaspis*) (Uganda; widely distributed species)

*Hexacola muhavara* (Benoit, 1956a), **comb. n.** (*Daruna*) (cf comments above) (Rwanda)

*Hexacola octoclavum* Quinlan, 1986 (Democratic Republic of Congo)

*Hexacola pallidum* Quinlan, 1986 (Democratic Republic of Congo)

*Hexacola quinqueclavatum* Quinlan, 1986 (Cameroon, Madagascar, Nigeria, Democratic Republic of Congo, Zimbabwe)

*Hexacola quisnama* Quinlan, 1986 (South Africa, Uganda, Democratic Republic of Congo)

*Hexacola septemium* Quinlan, 1986 (Democratic Republic of Congo)

*Hexacola zama* Quinlan, 1986 (Democratic Republic of Congo)

#### Kleidotomini

This tribe, formerly treated as the ‘Kleidotoma group’, was determined to be monophyletic in Fontal-Cazalla et al. (2002). Forshage and Nordlander (2008) reinstated this tribe following Hellén (1960). Members of this group typically possess a ham-hock shaped forewing (Buffington and Sandler 2012), an abbreviated marginal cell in the forewing, the overall body is slender and elongate, and species for which biology is known, are typically ‘diggers’ who search for hosts in substrates such as old dung, algae, and fungus.

##### Cothonaspis

Taxon classificationAnimaliaHymenopteraFigitidae

Hartig, 1840

###### Remarks.

Rare.

###### Diagnosis.

Small elongate eucoilines without a hairy ring, with narrow wings with narrow triangular marginal cells. May be confused with certain *Leptopilina* with strongly reduced hairy ring, but *Cothonaspis* are far more elongate in shape, have globular heads, and a pointed metapleural corner, whereas *Leptopilina* are stout, have more transverse heads and an oblique metapleural corner. Easily separated from their closest relatives in the region, *Kleidotoma*, by the reduced hairy ring, wing apex truncate (not incised), and male F2 modified (not F1).

**Figure 35.**
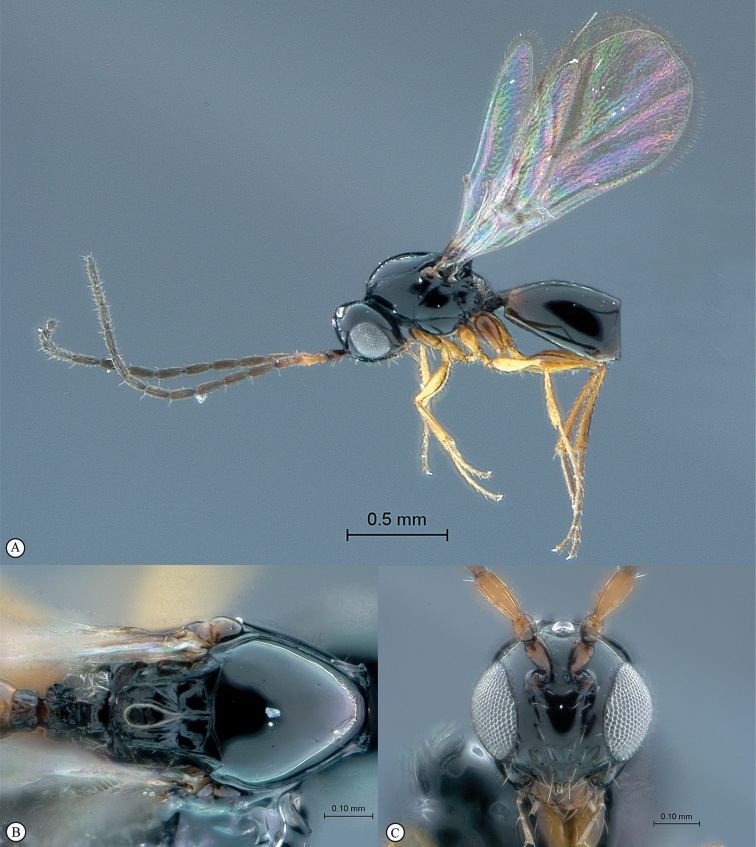
*Cothonaspis* species (Uganda). **A** habitus lateral view **B** head and mesosoma dorsal view **C** head, anterior view.

###### Distribution.

Mainly Palearctic, but also present in Nearctic and Afrotropical regions, and with a widespread species present throughout the Old World tropics. Afrotropical records: South Africa (Quinlan 1986), Central African Republic, Democratic Republic of Congo, Madagascar, Uganda, Yemen (here).

###### Biology.

Parasitoids of Sepsidae in dung (Nordlander 1976, Quinlan 1978, Pont and Meier 2002).

###### Species richness.

*Cothonaspis ealis* Quinlan, 1986 (South Africa)

Additional species, unidentified or undescribed.

##### Kleidotoma

Taxon classificationAnimaliaHymenopteraFigitidae

Westwood, 1833

###### Remarks.

Common throughout the Afrotropical region.

###### Diagnosis.

Usually elongate eucoilines of varying size, in most cases unmistakable through their incised wing apex alone (shared only with *Thoreauella* of Emargininae), but in forms with indistinctly incised wings (or where the wing shape is not readily visible) there are several other diagnostic characters: patchily reduced wing pubescence and the reduced wing venation with a small triangular marginal cell; fore wing veins of uneven width; distinctly pointed metapleural corner; narrow scutellar plate; and longitudinally striate dorsal surface of the scutellum. May be confused with *Hexacola*, who share the striate scutellum, the globular head, the often strongly modified male F1 and the sometimes narrow triangular marginal cell, but *Hexacola* typically have a very large and convex scutellar plate and narrow oblique scutellar foveae – and always a rectangular metapleural corner and a non-incised wing apex.

*Kleidotoma* is a taxon that is difficult to overview, and globally there are very few recognisable species-groups that are not obviously artificial. There is a general spectrum from tiny, often brown, species with little reduction of wing pubescence, and large, often black, species with very strong reduction of wing pubescence. And there are the aberrant brachypterous taxa (often ripicolous species occuring in algae or wrack, sometimes on isolated islands; but at least in the Holarctic also in ground-dwelling species in grasslands). The wingless or brachypterous forms among *Kleidotoma* are the only cynipoids with this state found in the Afrotropical region so far (though in other regions, *Rhoptromeris* and *Alloxysta* occasionally show brachyptery too, and such specimens may be found in the Afrotropical region). However, beyond what is already said here, we are not at the level of knowledge to start discussing species groups in *Kleidotoma* in a meaningful way.

**Figure 36.**
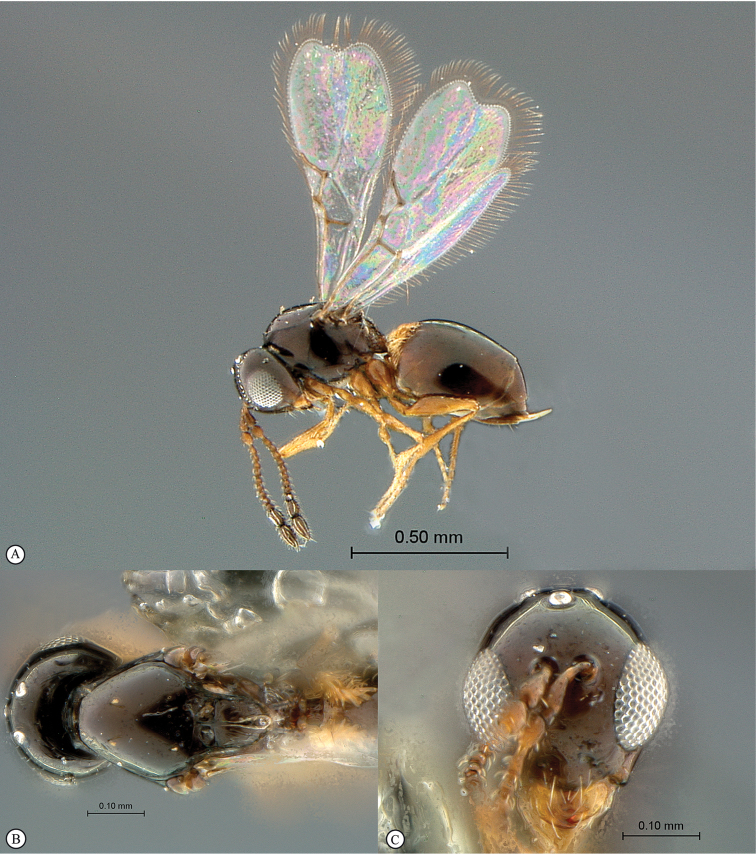
*Kleidotoma* species (Central African Republic). **A** habitus lateral view **B** head and mesosoma dorsal view **C** head, anterior view.

###### Distribution.

Worldwide. Afrotropical records: Cameroon, Democratic Republic of Congo, Ethiopia, Kenya, Nigeria, Rwanda, South Africa, Uganda, Zimbabwe (Quinlan 1986), St Helena (Dessart 1976), Burkina Faso, Burundi, Cape Verde, Central African Republic, Gabon, Gambia, Madagascar, Mozambique, Sierra Leone, Somalia, Yemen (here).

###### Biology.

Parasitoids of various flies in concealed and mostly decomposing habitats; debris, dung, carrion, fruit, fungi, grass, seawrack, aquatic plants etc. Broad host range, common hosts in other parts of the world include Drosophilidae, Sepsidae, Ephydridae and others (Baker 1979, Beardsley 1990, 1993, Belizin 1963, Burghele 1959, Carton et al. 1986, Díaz and Gallardo 1996, Driessen et al. 1990, Jonsell et al. 1999, Pont and Meier 2002, Quinlan 1978, Weld 1952, and others; plus additional label data).

###### Species richness.

*Kleidotoma arbitra* Quinlan, 1986 (Democratic Republic of Congo, South Africa, Uganda, Zimbabwe)

*Kleidotoma bifurcata* Quinlan, 1986 (Democratic Republic of Congo)

*Kleidotoma conica* Quinlan, 1986 (Democratic Republic of Congo)

*Kleidotoma distenda* Quinlan, 1986 (Democratic Republic of Congo, Nigeria, South Africa)

*Kleidotoma eala* Quinlan, 1986 (Democratic Republic of Congo)

*Kleidotoma elongula* Quinlan, 1986 (Democratic Republic of Congo, South Africa, Zimbabwe)

*Kleidotoma erebus* Quinlan, 1986 (Democratic Republic of Congo)

*Kleidotoma favus* Quinlan, 1986 (Cameroon, Democratic Republic of Congo, Kenya, Nigeria, Uganda, Zimbabwe)

*Kleidotoma fimbriata* Quinlan, 1986 (Democratic Republic of Congo)

*Kleidotoma miroscutellaris* (Dessart, 1976) (*Polbourdouxia*) (St Helena)

*Kleidotoma montana* Kieffer, 1910d (Rwanda)

syn *Kleidotoma africana* Benoit, 1956a nec Kieffer, 1910d (lapsus)

*Kleidotoma morsum* Quinlan, 1986 (Democratic Republic of Congo, South Africa)

*Kleidotoma nigrans* Quinlan, 1986 (Democratic Republic of Congo)

*Kleidotoma nitidiuscula* Quinlan, 1986 (Democratic Republic of Congo)

*Kleidotoma norma* Quinlan, 1986 (Zimbabwe)

*Kleidotoma strigosa* Quinlan, 1986 (Cameroon, Democratic Republic of Congo, Kenya, Nigeria, Uganda, Zimbabwe)

*Kleidotoma ventosa* Quinlan, 1986 (Democratic Republic of Congo, Ethiopia, South Africa)

Also undescribed species.

#### Trichoplastini

Trichoplastini is a tribe that is easily recognisable by the striking morphological synapomorphy of having a pronotal plate with lateral bridges closing the lateral cavities. The tribe may ultimately be synonymised with Eucoilini; the two tribes currently constitute a monophyletic clade. Throughout Africa, *Rhoptromeris* is by far the most commonly encountered Trichoplastini, and in fact, may be the most commonly encountered figitid group.

##### Angustacorpa

Taxon classificationAnimaliaHymenopteraFigitidae

Quinlan, 1988

###### Remarks.

Rare. This genus may be an apomorphic ingroup of *Trichoplasta*, but for the time being we recognize this taxon as a valid genus.

###### Diagnosis.

Very characteristic through their spectacular degree of lateral compression. Otherwise in all respects similar to *Trichoplasta* (pronotal plate with lateral bridges, narrow scutellar plate, extended posterior lobe of scutellum), and some *Trichoplasta* do indeed approach the *Angustacorpa* habitus (indicating the possibility of *Angustacorpa* being just an apomorphic ingroup), but the true *Angustacorpa* are always recognisable by their head: compound eyes are placed at mid-height of head, are pubescent, and their outline does not project from the general outline of the head capsule.

**Figure 37.**
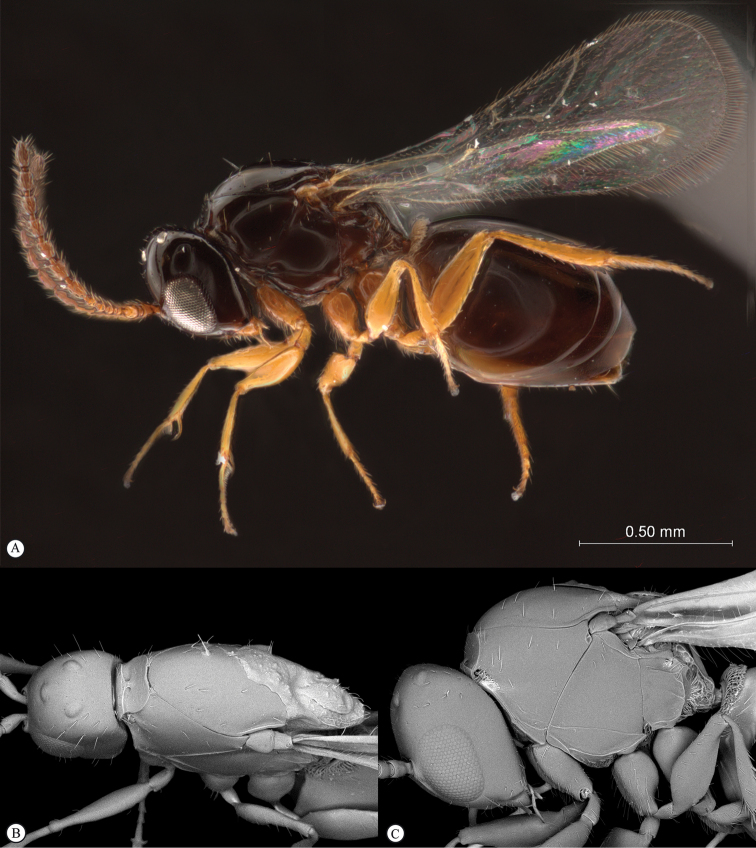
*Angustacorpa* species (Kenya). **A** habitus lateral view **B** head and mesosoma dorsal view **C** head and mesosoma lateral view.

###### Distribution.

Endemic to the Afrotropical region: Democratic republic of Congo, Kenya (Quinlan 1988), Cameroon, Central African Republic, Guinea, Republic of Congo, South Africa, Uganda, Yemen (here).

###### Biology.

Host unknown, but expected to attack a dipterous host in a narrow space such as under tree bark.

###### Species richness.

*Angustacorpa apsus* Quinlan, 1988 (Democratic Republic of Congo, Kenya)

*Angustacorpa persa* Quinlan, 1988 (Democratic Republic of Congo)

*Angustacorpa prodicus* Quinlan, 1988 (Democratic Republic of Congo)

*Angustacorpa triton* Quinlan, 1988 (Democratic Republic of Congo)

##### Nanocthulhu

Taxon classificationAnimaliaHymenopteraFigitidae

Buffington, 2012

###### Remarks.

Rare. Possibly an apomorphic ingroup of *Rhoptromeris*.

###### Diagnosis.

Species of this genus possess a three-pronged protrusion (fuscina) atop a dorsally elongate clypeus. Superficially, this taxon is similar to *Stentorceps*, since both genera have species with extensive projections from the head region. However, *Stentorceps* has a corniculum protruding from the face, and lacks a modified clypeus. The rest of the body is similar to that of the smallest *Rhoptromeris*. Some *Rhoptromeris* and *Hexacola* also have protrusions from the clypeal region, but they are merely points or small conical projections.

**Figure 38.**
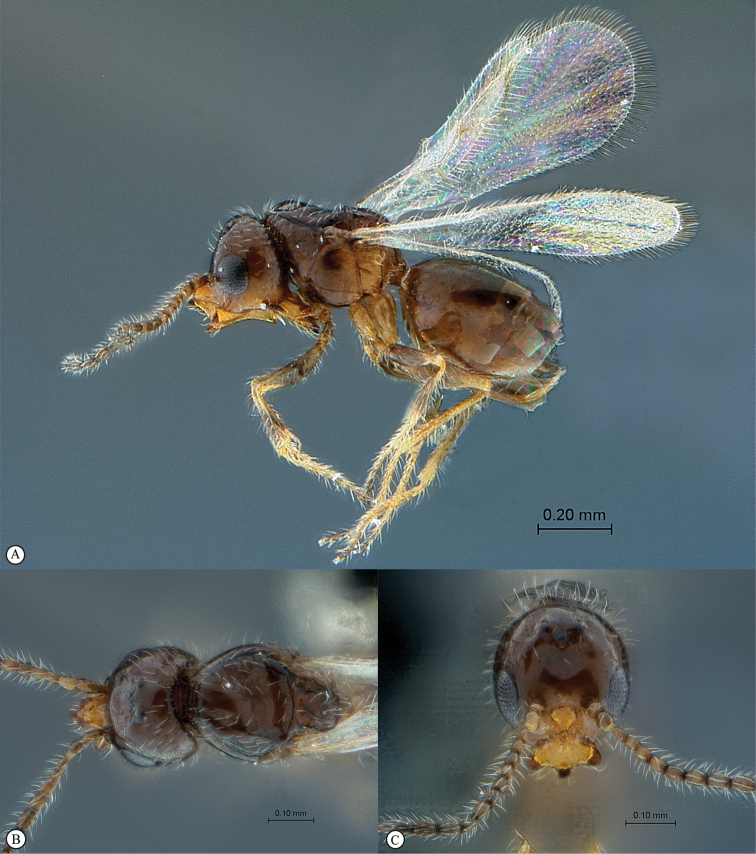
*Nanocthulhu lovecrafti* (Holotype, South Africa). **A** habitus lateral view **B** head and mesosoma dorsal view **C** head, anterior view.

###### Distribution.

Endemic to the Afrotropical region: South Africa (Buffington 2012), Mozambique (L. Masner pers. comm.).

###### Biology.

Host unknown.

###### Species richness.

*Nanocthulhu lovecrafti* Buffington, 2012 (South Africa)

An additional undescribed species is known from Mozambique.

##### Rhoptromeris

Taxon classificationAnimaliaHymenopteraFigitidae

Förster, 1869

###### Remarks.

Ubiquitous; the most common genus of eucoilines in Africa. This taxon keys out three times in the generic key above, which reflects the morphological plasticity and complexity of the genus. Though many Afrotropical species have been described, there are undoubtedly many more undescribed species awaiting description. Further, Quinlan’s (1986) reliance on antennal characters to separate species within *Rhoptromeris* will have to be revisited too, since antennal characters themselves without other characters are frequently unreliable in figitid taxonomy.

###### Diagnosis.

Mostly small and elongate eucoilines, with narrow wings and a narrow triangular marginal cell. Males usually have a strongly modified antennal F2. *Rhoptromeris* are easily recognised by the combination of a pronotal plate with lateral bridges, a scutellum lacking a protruding posterior lobe, and the dorsal surface of the scutellum distinctly striate, especially anteriorly. These characters separate the genus from the morphologically similar *Trichoplasta*. However, intermediate forms between these two genera have been examined, creating difficulties in separating the two genera; in these cases, focusing on the striate dorsal surface of the scutellum will reliably run these species in the key. If the pronotal plate is not obvious, the diversity of morphotypes of *Rhoptromeris* in this region may create confusion with several other genera.

The most common Afrotropical specimens are small, brown and elongate; have narrow elongate wings with a narrow triangular marginal cell; and have a circular incision near metapleural corner. Some, often larger, have a more elongate incised (or depressed) area along metapleural posterior margin. These may possibly be mistaken for several other eucoiline genera of a similar general build; *Hexacola*, *Ganaspis*, *Kleidotoma* or *Cothonaspis*. *Rhoptromeris* is separated from all of these by the pronotal plate, from all but *Cothonaspis* by the male antennae, and from most of these genera by differences in the metapleuron and scutellar plate.

Larger taxa are in general stouter, less elongate, have darker colour, broader wings, a deeper marginal cell and a shorter hair fringe on the wings; and approach *Leptopilina* or some *Trybliographa* or *Trichoplasta* in appearance.

A distinct species group is represented by tiny specimens with an indistinct or absent mesopleural line, a very long hair fringe on the wing, and often a shorter marginal cell; these are superficially very similar to *Micreriodes* and *Endecameris*, but easily recognised by their pronotal plate.

Another distinct species group have a pointed posterior lobe of the scutellum and are very easily confused with *Trichoplasta*; separated only by the general build of the scutellum, which has a convex scutellar plate and at least partly smooth or lineate sculpture of the dorsal surface. Of these we have seen a small, stout black taxon from South Africa, a large dark brown taxon from Uganda, and some less characteristic small brown forms from elsewhere.

One or several species groups have protuberances on the face, small often pointed tubercles on the clypeus and/or malar spaces and sometimes modified mandibles; they are small or tiny, and more stoutly built than typical *Rhoptromeris*; their appearance approaches *Nanocthulhu* or *Stentorceps*, both of which have more spectacular facial protuberances. Most of these specimens are from South Africa, but we have also seen singletons from East African countries.

**Figure 39.**
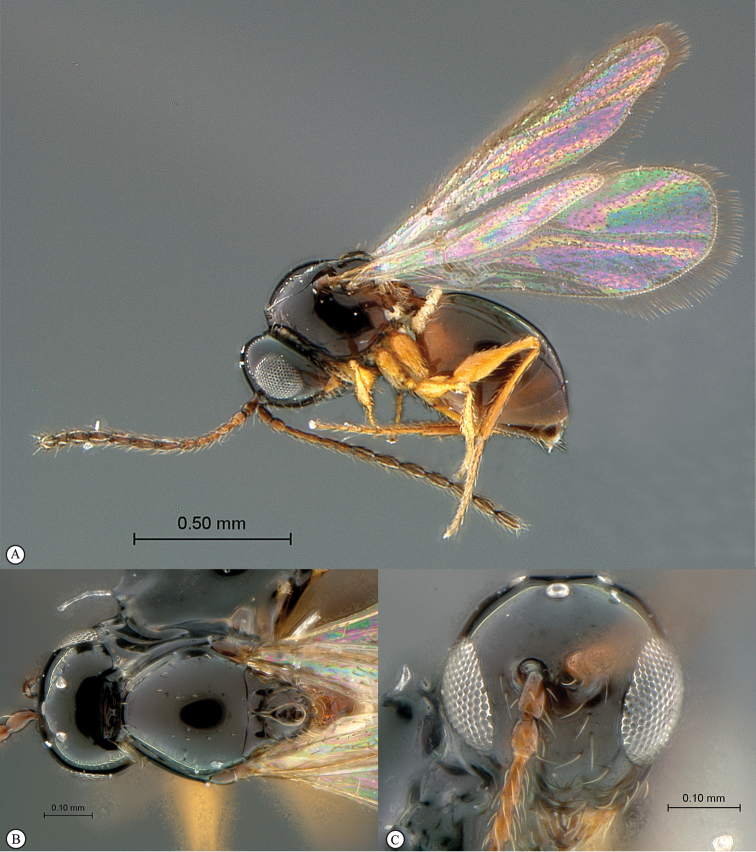
*Rhoptromeris* species (Uganda). **A** habitus lateral view **B** head and mesosoma dorsal view **C** head, anterior view.

###### Distribution.

Worldwide. Afrotropical records: Cameroon, Democratic Republic of Congo, Kenya, Madagascar, Nigeria, Reunion, South Africa, Uganda (Quinlan 1986), Botswana, Burkina Faso, Cape Verde, Central African Republic, Comoros, Ethiopia, Gabon, Ghana, Ivory Coast, Malawi, Republic of Congo, Rwanda, Senegal, Seychelles, Sierra Leone, Tanzania, Zimbabwe, Yemen (here).

###### Biology.

As far as known they attack Chloropidae on grasses and fungi (numerous sources including Bhattacharyya 1957, Quinlan 1978, Nordlander 1978, Nordlander and Grijpma 1991), but no certain records are from within the Afrotropical region.

###### Species richness.

*Rhoptromeris abba* Quinlan, 1986 (Democratic Republic of Congo, Kenya)

*Rhoptromeris afer* Quinlan, 1986 (Democratic Republic of Congo, Uganda)

*Rhoptromeris agis* Quinlan, 1986 (Democratic Republic of Congo)

*Rhoptromeris attis* Quinlan, 1986 (Democratic Republic of Congo)

*Rhoptromeris bicolor* Quinlan, 1986 (Democratic Republic of Congo, Nigeria, Zimbabwe)

*Rhoptromeris bupalus* Quinlan, 1986 (Democratic Republic of Congo, Kenya, Reunion, Uganda, Zimbabwe)

*Rhoptromeris cepheus* Quinlan, 1986 (Kenya)

*Rhoptromeris connata* Quinlan, 1986 (Democratic Republic of Congo)

*Rhoptromeris crito* Quinlan, 1986 (Madagascar)

*Rhoptromeris cubitalis* Quinlan, 1986 (Democratic Republic of Congo)

*Rhoptromeris diversa* Quinlan, 1986 (Democratic Republic of Congo, South Africa, Uganda)

*Rhoptromeris enna* Quinlan, 1986 (Democratic Republic of Congo)

*Rhoptromeris equalis* Quinlan, 1986 (Cameroon, Democratic Republic of Congo)

*Rhoptromeris hebe* Quinlan, 1986 (Democratic Republic of Congo)

*Rhoptromeris heptoma* (Hartig, 1840) (Democratic Republic of Congo, South Africa; Palearctic species)

*Rhoptromeris navius* Quinlan, 1986 (Democratic Republic of Congo, Kenya, Zimbabwe)

*Rhoptromeris naxos* Quinlan, 1986 (Democratic Republic of Congo, Uganda)

*Rhoptromeris oeta* Quinlan, 1986 (Democratic Republic of Congo)

*Rhoptromeris pagasa* Quinlan, 1986 (Cameroon, Democratic Republic of Congo)

*Rhoptromeris pallida* Quinlan, 1986 (Democratic Republic of Congo, Nigeria)

*Rhoptromeris persius* Quinlan, 1986 (Democratic Republic of Congo)

*Rhoptromeris punctata* Quinlan, 1986 (Democratic Republic of Congo)

*Rhoptromeris rufula* Quinlan, 1986 (Democratic Republic of Congo, South Africa)

*Rhoptromeris rutshuris* Quinlan, 1986 (Cameroon, Democratic Republic of Congo)

*Rhoptromeris rwanki* Quinlan, 1986 (Democratic Republic of Congo)

*Rhoptromeris sinis* Quinlan, 1986 (Cameroon, Democratic Republic of Congo)

*Rhoptromeris temesa* Quinlan, 1986 (Democratic Republic of Congo)

*Rhoptromeris thales* Quinlan, 1986 (Democratic Republic of Congo, South Africa)

*Rhoptromeris velia* Quinlan, 1986 (Democratic Republic of Congo, South Africa)

*Rhoptromeris zetes* Quinlan, 1986 (Democratic Republic of Congo)

*Rhoptromeris zeus* Quinlan, 1986 (Democratic Republic of Congo)

##### Stentorceps

Taxon classificationAnimaliaHymenopteraFigitidae

Quinlan, 1984

###### Remarks.

Rare. Possibly an apomorphic group in *Trichoplasta*.

###### Diagnosis.

Unmistakable head morphology, with distinct trumpet-shaped protrution (corniculum) present between toruli. Superficially similar to *Nanocthulhu*, since both have extensive projections from the head region. However, *Stentorceps* has a corniculum protruding from the frons, and *Nanocthulhu* has a three-pronged extension (fuscina) atop a protruding clypeus. The rest of the body is similar to a *Trichoplasta*, thus with a posterior protrusion on the scutellum, which is another character that separates them from *Nanocthulhu*. Some species of *Rhoptromeris* and *Hexacola* have protrusions from the malar space and clypeus, but they also lack the corniculum of *Stentorceps*.

**Figure 40.**
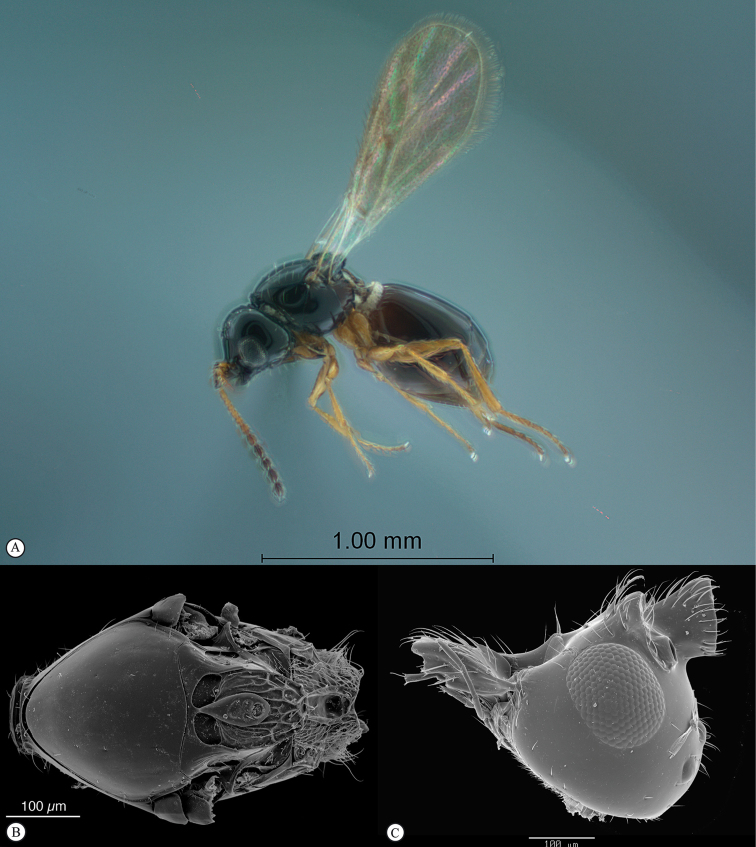
*Stentorceps tubicen* (Kenya). **A** habitus lateral view **B** head and mesosoma dorsal view **C** head, anterior view.

###### Distribution.

Endemic to the Afrotropical region and mainly occurring in East Africa. Afrotropical records: Kenya (Quinlan 1984), Botswana, Madagascar, Nigeria, Rwanda, Somalia, South Africa, Uganda (Nielsen and Buffington 2011), Republic of Congo, Tanzania, Yemen (here).

###### Biology.

Hosts unknown.

###### Species richness.

*Stentorceps abbotti* Nielsen & Buffington, 2011 (Kenya)

*Stentorceps heimdalli* Nielsen & Buffington, 2011 (Kenya, Nigeria, Republic of Congo, Rwanda, Somalia, South Africa, Uganda)

*Stentorceps tubicen* Quinlan, 1984 (Kenya, Zimbabwe)

*Stentorceps vuvuzela* Nielsen & Buffington, 2011 (Kenya)

*Stentorceps weedlei* Nielsen & Buffington, 2011 (Botswana, Madagascar)

*Stentorceps zuparkoi* Nielsen & Buffington, 2011 (Madagascar, South Africa)

##### Trichoplasta

Taxon classificationAnimaliaHymenopteraFigitidae

Benoit, 1956a

###### Remarks.

Common.

###### Diagnosis.

Often easily recognisable by the combination of a pronotal plate with lateral bridges, a posteriorly protruding scutellum, and the dorsal surface of the scutellum distinctly foveate. Species with a moderately protruding scutellum can be difficult to distinguish from *Rhoptromeris*, but focusing on the foveate sculpture of the dorsal surface of the scutellum will run an unknown species through the key effectively.

**Figure 41.**
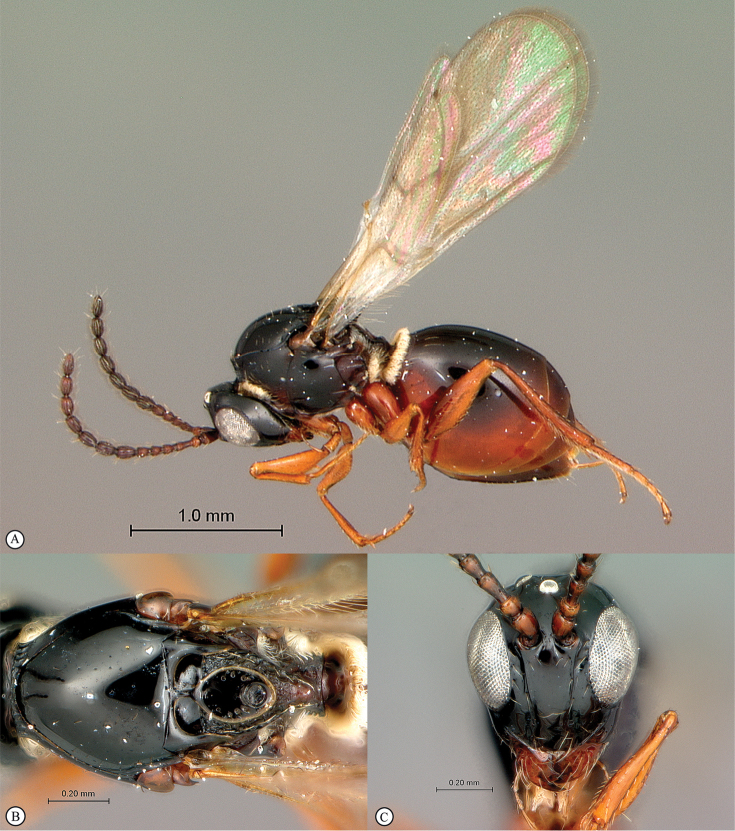
*Trichoplasta tanganyikensis* (Central African Republic). **A** habitus lateral view **B** head and mesosoma dorsal view **C** head, anterior view.

###### Distribution.

Holarctic region and Old World Tropics. Appears to have its largest diversity in the Afrotropical region. Afrotropical records: Cameroon, Democratic Republic of Congo, Kenya, Nigeria, Rwanda, South Africa, Tanzania, Uganda, Zimbabwe (Quinlan 1986), Burkina Faso, Cape Verde, Cameroon, Central African Republic, Comoros, Gabon, Gambia, Ghana, Guinea-Bissau, Ivory Coast, Malawi, Republic of Congo, Sierra Leone, Sudan, Yemen (here).

###### Biology.

Specimen label data suggests tropical species appear to attack fruit-infesting Lonchaeidae and Muscidae (label data), but in the Holarctic they are mostly wood-associated, attacking Drosophilidae and Lonchaeidae under tree bark (label data), and these habits are probably present in some Afrotropical taxa too.

###### Species richness.

*Trichoplasta afrobicolor* **nom. n.** (*Trichoplasta bicolor* Quinlan, 1986 secondary junior homonym of *Trichoplasta bicolor* (Ionescu, 1969) (*Hypolethria*), which was transferred to *Trichoplasta* by Nordlander (1982) so Quinlan’s name was a homonym already at the time of its original description) (South Africa)

*Trichoplasta brevispina* (Masner, 1960) (*Odonteucoila*) (Kenya, Zimbabwe)

*Trichoplasta conica* Quinlan, 1986 (Democratic Republic of Congo, Kenya, Nigeria, Uganda)

*Trichoplasta contrasta* Quinlan, 1986 (Democratic Republic of Congo, Nigeria)

*Trichoplasta equalis* Quinlan, 1986 (Democratic Republic of Congo, Nigeria)

*Trichoplasta extensus* Quinlan, 1986 (Democratic Republic of Congo)

*Trichoplasta filiformis* Quinlan, 1986 (Democratic Republic of Congo, South Africa)

*Trichoplasta gracilicornis* (Kieffer, 1910c) (*Coneucoila*) (Democratic Republic of Congo, South Africa)

*Trichoplasta longispina* (Masner, 1960) (*Odonteucoila*) (Democratic Republic of Congo)

*Trichoplasta medlia* Quinlan, 1986 (Democratic Republic of Congo, Nigeria)

*Trichoplasta narrata* Quinlan, 1986 (Democratic Republic of Congo, Uganda)

*Trichoplasta novema* Quinlan, 1986 (Democratic Republic of Congo, Uganda)

*Trichoplasta octonarius* Quinlan, 1986 (Democratic Republic of Congo)

*Trichoplasta quinclava* Quinlan, 1986 (Democratic Republic of Congo)

*Trichoplasta rufa* Quinlan, 1986 (Democratic Republic of Congo, Madagascar)

*Trichoplasta tanganyikensis* (Weld, 1944) (*Coneucoila*) (Cameroon, Democratic Republic of Congo, Rwanda, Tanzania)

*Trichoplasta testacea* Quinlan, 1986 (Nigeria)

*Trichoplasta unicolora* Quinlan, 1986 (Democratic Republic of Congo)

*Trichoplasta zeus* Quinlan, 1986 (Cameroon)

#### Tribal placement uncertain

##### Garudella

Taxon classificationAnimaliaHymenopteraFigitidae

Buffington & Forshage, 2014

###### Remarks.

Very rare, in Africa only known from a single specimen in the Republic of Congo. The tribal placement of this taxon is uncertain. Key characters make it run to Trichoplastini, but both habitus and a number of other characters are more similar to *Cothonaspis*, and in the original description it is tentatively placed in Kleidotomini. It may, along with *Cothonaspis* and *Triplasta* Kieffer, represent a basal lineage in that tribe, but in the absence of a proper phylogenetic analysis, this remains little more than just a guess.

###### Diagnosis.

The thick basal part of the pronotal plate and the very robust petiole are unique characters in the Eucoilinae. The overall body shape is similar to *Cothonaspis*, and the wings are similar to *Cothonaspis* and a number of other genera (*Rhoptromeris*, *Trichoplasta*, and many *Leptopilina*), but *Garudella* can be separated based on the morphology of the pronotal plate and the uniquely robust propodeal-petiolar complex.

**Figure 42.**
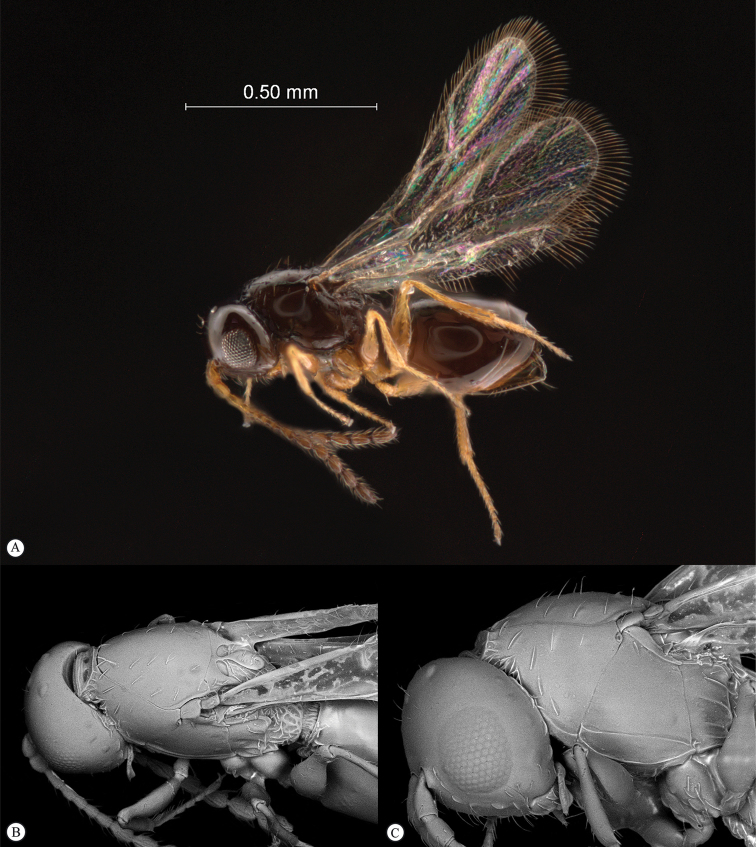
*Garudella afrotropica* (Republic of Congo). **A** habitus lateral view **B** head and mesosoma dorsal view **C** head and mesosoma lateral view.

###### Distribution.

Oriental and Afrotropical regions. Afrotropical records: Republic of Congo.

###### Biology.

Host unknown.

###### Species richness.

*Garudella afrotropica* Buffington & Forshage, 2014 (Republic of Congo)

##### *Leptolamina* group

This is a distinct group of eucoiline genera that is currently not assignable to a tribe. Included genera were previously associated with the informal “*Chrestosema* group” (Nordlander 1982), most representatives of which are now included in the Ganaspini. Buffington et al. (2007) recovered *Leptolamina* and *Sirenes* as a sister-group clade to what is now Eucoilini and Trichoplastini. With the synonymisation of these two genera, this group currently only contains *Leptolamina* and *Micreriodes*; additional sampling in the Old World tropics may yield more genera that would be assignable to this group.

###### Leptolamina

Taxon classificationAnimaliaHymenopteraFigitidae

Yoshimoto, 1962

Leptolamina (synonym *Sirenes* Quinlan, 1988, **syn. n.**)

####### Remarks.

*Leptolamina* was originally described from the Pacific region (Yoshimoto 1962). Quinlan (1988), in his treatment of the Afrotropical fauna, cites Nordlander (1982) recognising *Leptolamina* as a member of the *Chrestosema* group of genera, but does not consider the taxon with regards to the African fauna. The *Glauraspidia* described by Quinlan in the same work (1988) are in fact *Leptolamina* (new combinations below). Also in the same work (1988), he described the genus *Sirenes* to accommodate species of eucoilines that lacked a mesopleural line, but whose surface sculpture was matte, and otherwise conformed to his concept of *Glauraspidia*. After examining many specimens collected throughout the Afrotropical region, we have determined that *Sirenes* is at best one end of a morphological spectrum, which also includes *Leptolamina*. On the *Sirenes* end, forms are typically larger, slightly more matte, heads slightly more elongate, and lateral depressions of the mesoscutum slightly more developed. Altogether, we have found many intermediate forms, without any distinct morphological features to suggest monophyly of each genus; we hypothesize that the features mentioned above are the result of allometry related to overall body size. Hence, we hereby make *Sirenes* a junior synonym of *Leptolamina* (see below). This decision finds further motivation in the phylogenetic analysis of Buffington et al. (2007) which found that *Sirenes* rendered *Leptolamina* paraphyletic.

####### Diagnosis.

Entire wasp lightly to heavily matte over entire body, but particularly on head and mesoscutum. Mesopleural line entirely absent. Pronotal plate distinctly directed anteriorly, anterior half (just behind head) wider than posterior half. Pronotal fovea indistinct in most cases; when visible, closed laterally (lateral bridge present). Hind coxae often entirely without hairpatch. Setae on wings ranging from normal to a particular form with dark, broad sockets; setae along anterior aspect of marginal cell very stout in larger forms. Face elongate to round, mandibles ranging from blocky, subquadrate to smaller and triangular; malar space with very slight striations running from the ventral margin of the compound eye to the mandibular base. Scutellar plate typically narrow, elongate; occasionally wider, tear-drop shaped. Longitudinal lateral depressions of mesoscutum present in larger specimens, absent in smaller ones. Shares several of its characteristics (matte finish, longidutinal lateral depressions of mesoscutum, very elongate coxae etc) with *Chrestosema*, but the latter taxon always has a mesopleural line and an elongate hairline along metacoxae. Very small specimens of *Leptolamina* will approach the appearance of *Micreriodes*, but the latter is far less “foamy”.

**Figure 43.**
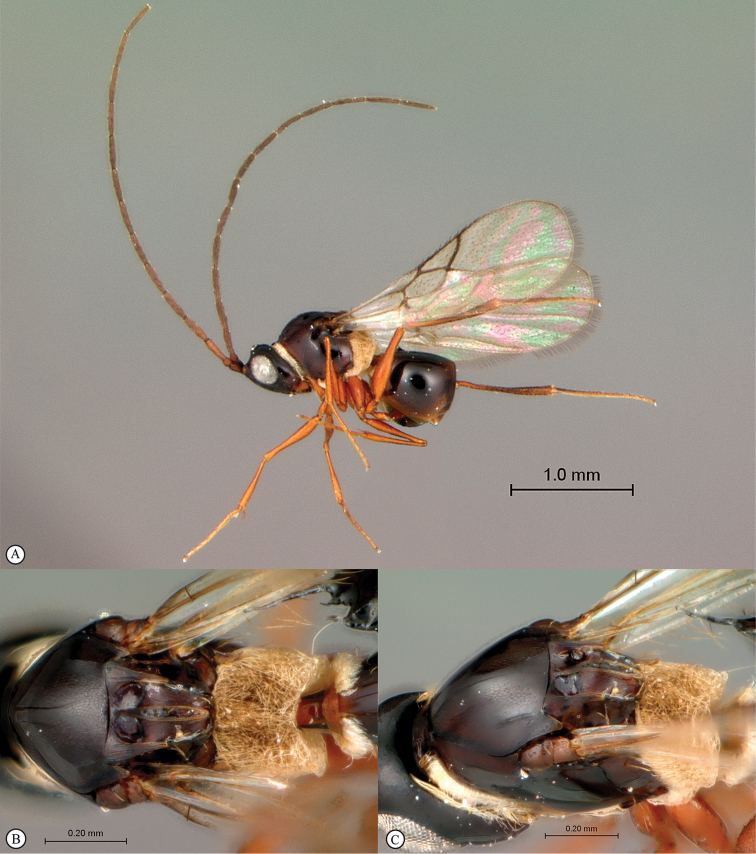
*Leptolamina* species (Central African Republic). **A** habitus lateral view **B** mesosoma dorsal view **C** head and mesosoma dorsolateral view.

####### Distribution.

Mainly Old World tropics, but also in eastern Palaearctic. Afrotropical records: Democratic Republic of Congo, Ivory Coast, Seychelles, Zambia, Zimbabwe (Quinlan 1986), Cameroon, Central African Republic, Comoros, Gabon, Kenya, Madagascar, Mauritius, Republic of Congo, South Africa, Uganda (here).

####### Biology.

Reared from Drosophilidae in Japan (label data).

####### Species richness.

*Leptolamina casca* (Quinlan, 1988), **comb. n.** (*Glauraspidia*) Type in BMNH studied by MF (Seychelles)

*Leptolamina floccus* (Quinlan, 1988), **comb. n.** (*Sirenes*) Type in RMCA studied by MB (Democratic Republic of Congo)

*Leptolamina orbilus* (Quinlan, 1988), **comb. n.** (*Sirenes*) Type in RMCA studied

by MB (Democratic Republic of Congo)

*Leptolamina scyphus* (Quinlan, 1988), **comb. n.** (*Glauraspidia*) Type in BMNH studied by MF (Democratic Republic of Congo, Ivory Coast, Zambia, Zimbabwe)

*Leptolamina seychellensis* (Kieffer, 1911a) (*Eucoila*) (Seychelles)

*Leptolamina silenus* (Quinlan, 1988), **comb. n.** (*Sirenes*) Type in BMNH studied by MF (Democratic Republic of Congo, Kenya)

*Leptolamina sinis* (Quinlan, 1988), **comb. n.** (*Sirenes*) type in BMNH studied by MF (Cameroon, Democratic Republic of Congo)

*Leptolamina spio* (Quinlan, 1988), **comb. n.** (*Sirenes*) Type in RMCA studied by MB (Democratic Republic of Congo)

*Leptolamina steropes* (Quinlan, 1988), **comb. n.** (*Sirenes*) Type in BMNH studied by MF (Democratic Republic of Congo, Kenya, South Africa)

*Leptolamina syrinx* (Quinlan, 1988), **comb. n.** (*Sirenes*) Type in RMCA studied by MB (Democratic Republic of Congo, South Africa)

*Leptolamina syrtes* (Quinlan, 1988), **comb. n.** (*Sirenes*) Type in RMCA studied by MB (Democratic Republic of Congo)

###### Micreriodes

Taxon classificationAnimaliaHymenopteraFigitidae

Yoshimoto, 1962

####### Remarks.

Rare in Afrotropical region. Previously known only from the Pacific, but actually distributed worldwide.

####### Diagnosis.

Tiny wasps with a well-developed set of “dwarfication” characters: a globular head, short antennae, sometimes a reduction in antennomere number, a narrow scutellum, very narrow wings with very long hair fringe, wing venation of uneven width, and fore wing marginal cell short and wide open. Similar to *Endecameris* and to some *Rhoptromeris* in some or all of these characters, but separated from the latter by the combination of having a pronotum with open lateral foveae, and lacking a mesopleural line.

**Figure 44.**
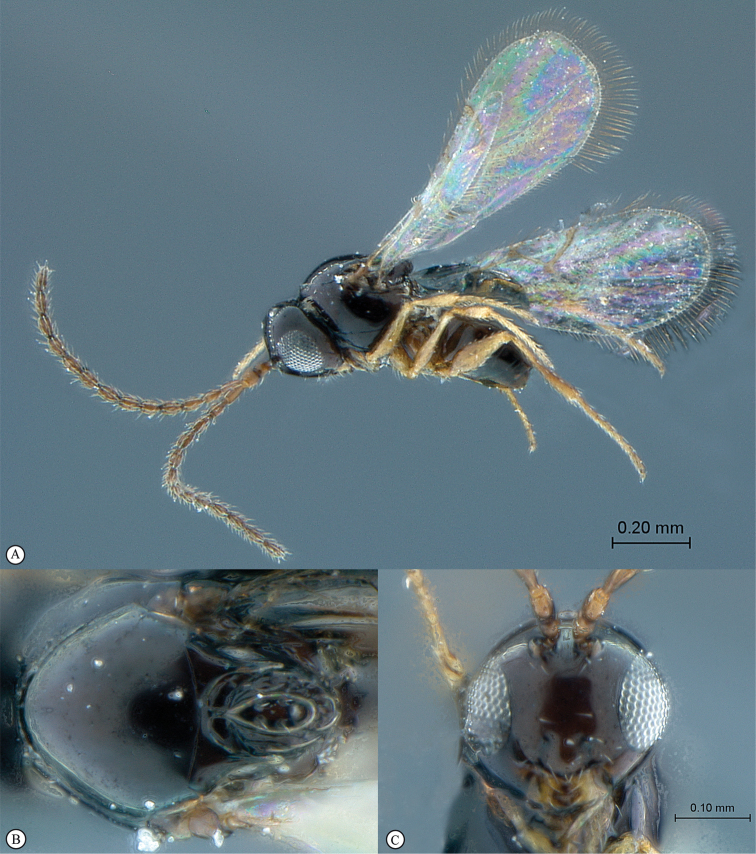
*Micreriodes* species (Central African Republic). **A** habitus lateral view **B** mesosoma dorsal view **C** head, anterior view.

####### Distribution.

Previously only known from the Pacific region, but are in fact widespread in the Old World tropics and recently a specimen was found in North America. Afrotropical records: Cameroon, Central African Republic, Madagascar, Nigeria, Reunion, Sierra Leone, Uganda (here).

####### Biology.

Hosts unknown, assumed to be Drosophilidae.

####### Species richness.

Undescribed species.

### Figitinae

The Figitinae are represented in the Afrotropical region by four genera containing 7 described species. A number of undescribed species are present in world collections. Figitinae contains a heterogenous assemblage of genera and is probably not monophyletic (Ronquist 1999, Buffington et al. 2007). However, the genera native to the Afrotropical region all belong to a very distinct lineage (big black wasps, with strongly reduced wing pubescence, hairy compound eyes, no hair patches at metasomal base, often with lateral striation of the mesosoma, often with scutellar spines, attacking calyptrate Diptera in dung and carrion), which is safely monophyletic, and in fact, present a very interesting morphological and life history convergence with some genera of Eucoilinae (in the Afrotropical region, namely *Bothrochacis*). Though in addition to the native genera, there is the genus *Lonchidia*, of which we have so far encountered only one Afrotropical specimen of a species present in Europe, which may be an accidental introduction or possibly an established population of synanthropic origin. This genus is very different from the major faunal component of Figitinae, and represents a separate lineage that renders the subfamily paraphyletic in phylogenetic analyses. It is easily recognisable by its confluent scutellar foveae.

**Biology.** Host records are lacking for Afrotropical species of Figitinae, however, Buffington et al. (2012) cite all confirmed host records for the group, and these all relate to calyptrate flies in dung and carrion.

**Distribution.** The subfamily is represented in all biogeographical regions (except the Antarctic) with the majority of described species occurring in the Holarctic and Neotropical regions. The paucity of Afrotropical and Oceanic species, as well as the almost total absence of Oriental records, may very well only reflect a poor state of taxonomic knowledge.

#### Key to Afrotropical figitine genera

(after van Noort et al. 2014)

**Table:** 

	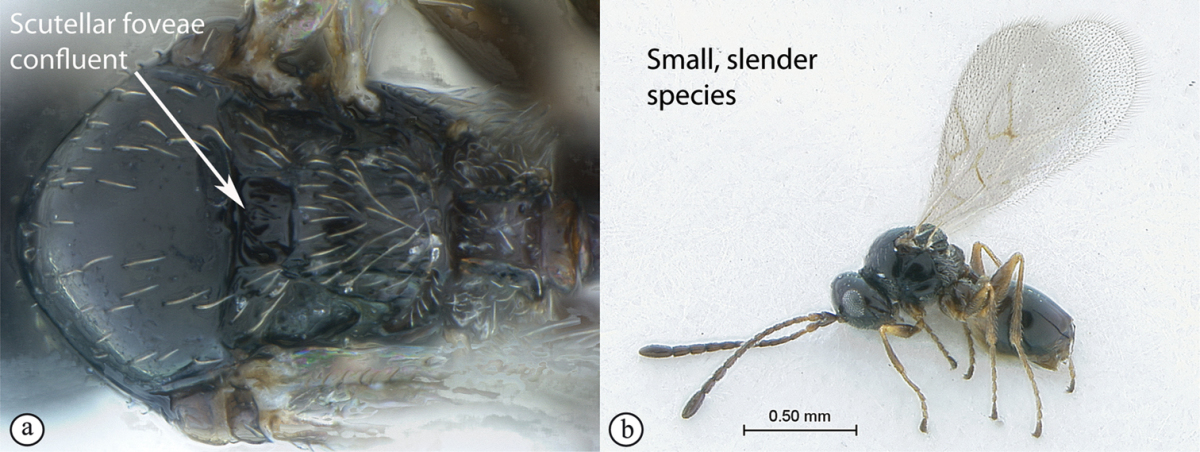	
1	Scutellar foveae confluent (a). Small, rather slender species (b)	***Lonchidia***
	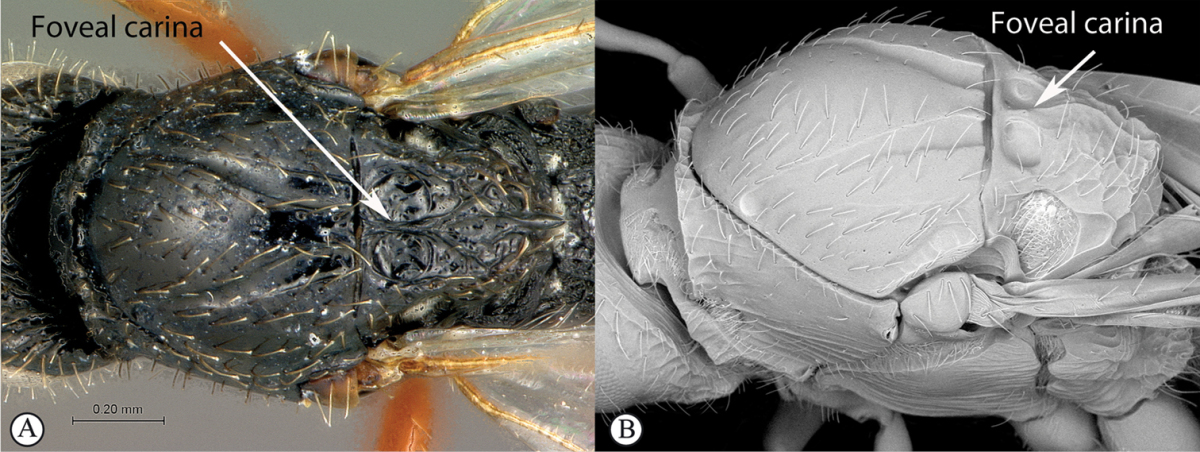	
–	Scutellar foveae distinctly separated by a median carina (A, B). Larger, strongly built species	**2**
	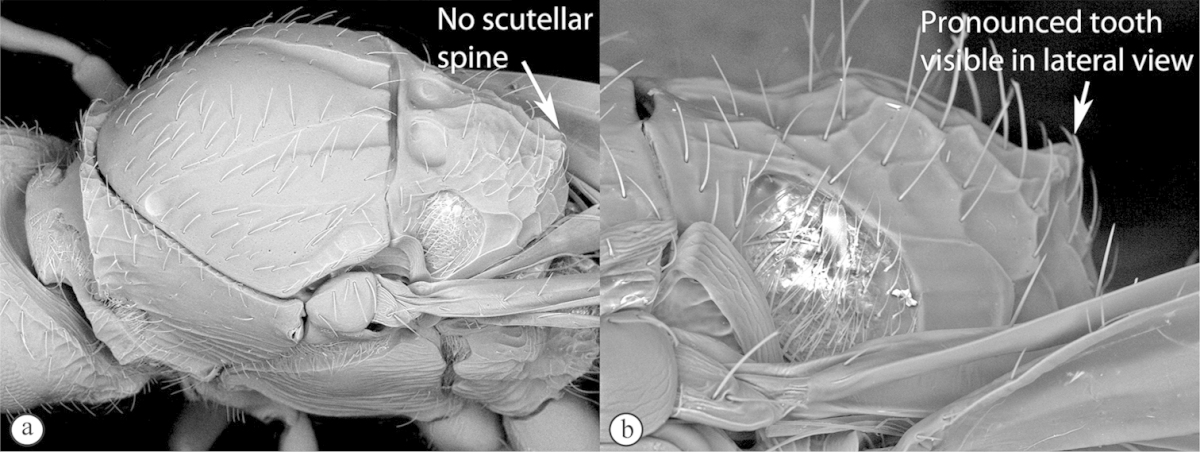	
2	No distinct scutellar spine (a), outline of scutellum in dorsal view rounded (however there is often a more or less pronounced tooth at the posterior most point of the circumscutellar carina, which may look like a small “spine” in lateral view) (b)	***Figites***
	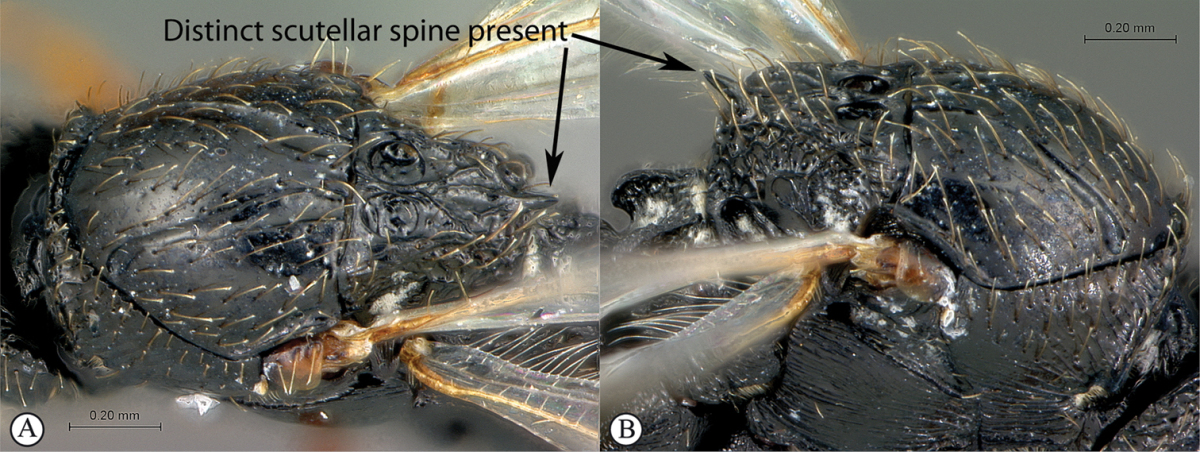	
–	Distinct scutellar spine present, obviously protruding from scutellar outline in dorsal view (A, B)	3
	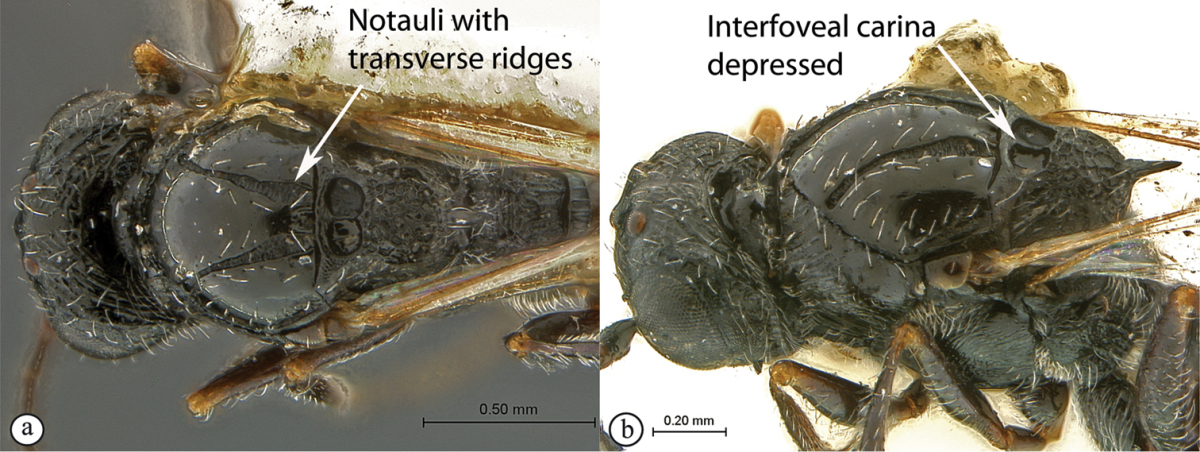	
3	Notauli sculptured with small transverse ridges (a). Interfoveal carina depressed, much lower than the level of the foveal edge (b)	***Xyalophora***
	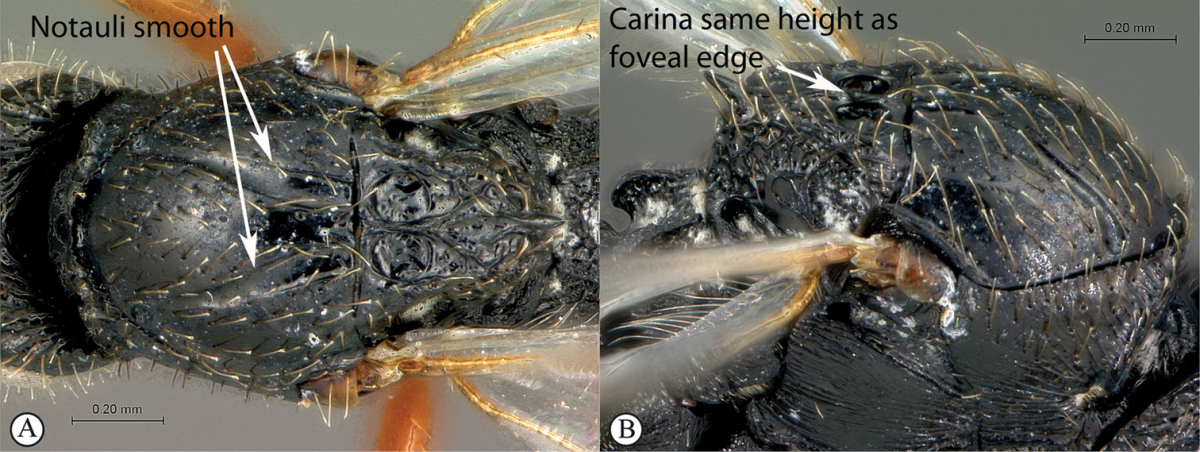	
–	Notauli smooth (A). Interfoveal carina as high as the foveal edge (B)	***Neralsia***

#### Figites

Taxon classificationAnimaliaHymenopteraFigitidae

Latreille, 1802

##### Remarks.

This is a rare genus in the region. The Afrotropical representatives of the genus were recently revised and four of Benoit’s species described in 1956 were sunk into synonymy with *Figites aciculatus* (van Noort et al. 2014). On a global scale, it is a poorly circumscribed genus versus several smaller genera, and many of its nominal species are of doubtful identity.

##### Diagnosis.

Large figitines with reduced pubescence on wings (often completely hairless) and more or less striate mesosomal sides. Easily separated from *Xyalophora* and *Neralsia* by the rounded scutellum (no indication of a spine in outline in dorsal view). Stiff, stout hairs present across most of body, distally bifurcate.

**Figure 45.**
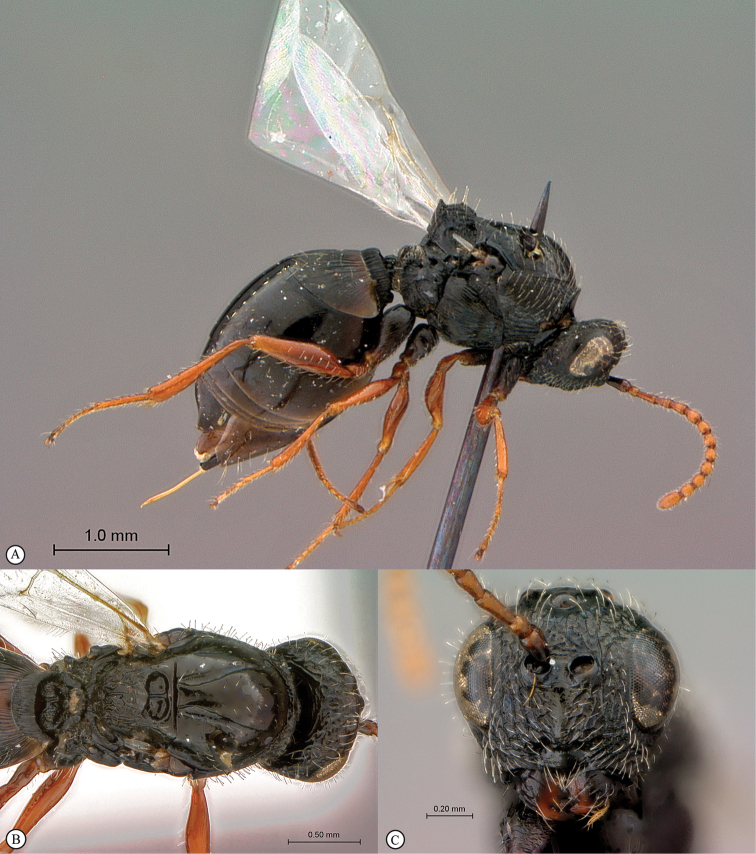
*Figites aciculatus* (Holotype, Democratic Republic of Congo). **A** habitus lateral view **B** head and mesosoma dorsal view **C** head, anterior view.

##### Distribution.

Probably worldwide, but to date no records from the Oriental and Oceanic regions have been published. Afrotropical records: Democratic Republic of Congo (Benoit 1956d), Cameroon, Ethiopia, Kenya, South Africa, Uganda, Yemen (van Noort et al. 2014).

##### Biology.

Parasitoids of calyptrate Brachycera larvae in decomposing substrates (Hennig 1976, James 1928, Thomas and Morgan 1972).

##### Species richness.

*Figites aciculatus* (Benoit, 1956d) (*Xyalophora*) (Cameroon, Democratic Republic of Congo, Ethiopia, Kenya, South Africa, Uganda, Yemen)

syn *Figites effossus* Benoit, 1956d (Democratic Republic of Congo)

syn *Figites favonius* Benoit, 1956d (Democratic Republic of Congo)

syn *Figites furvus* Benoit, 1956d (Democratic Republic of Congo)

syn *Figites fraudator* Benoit, 1956d (Democratic Republic of Congo)

#### Lonchidia

Taxon classificationAnimaliaHymenopteraFigitidae

Thomson, 1862

##### Remarks.

The only Afrotropical specimen seen so far is from South Africa and may be an accidental introduction. It corresponds to a form present in Europe, which is currently considered as belonging to *Lonchidia clavicornis* Thomson, but which differs from the type specimen in some minor respects. Further studies may possibly show that this is a separate, currently unnamed, species.

##### Diagnosis.

Small, rather slender, and more or less strongly pubescent figitines, easily recognised by the confluent scutellar foveae. Pubescence is dense in patches on the sides of the large metasomal tergite, as a collar on the pronotum, on the propodeum, and rather dense also on metapleura and metacoxae. The marginal cell of the forewing is characteristically short, and the antennae in females end with an enlarged apical flagellomere.

**Figure 46.**
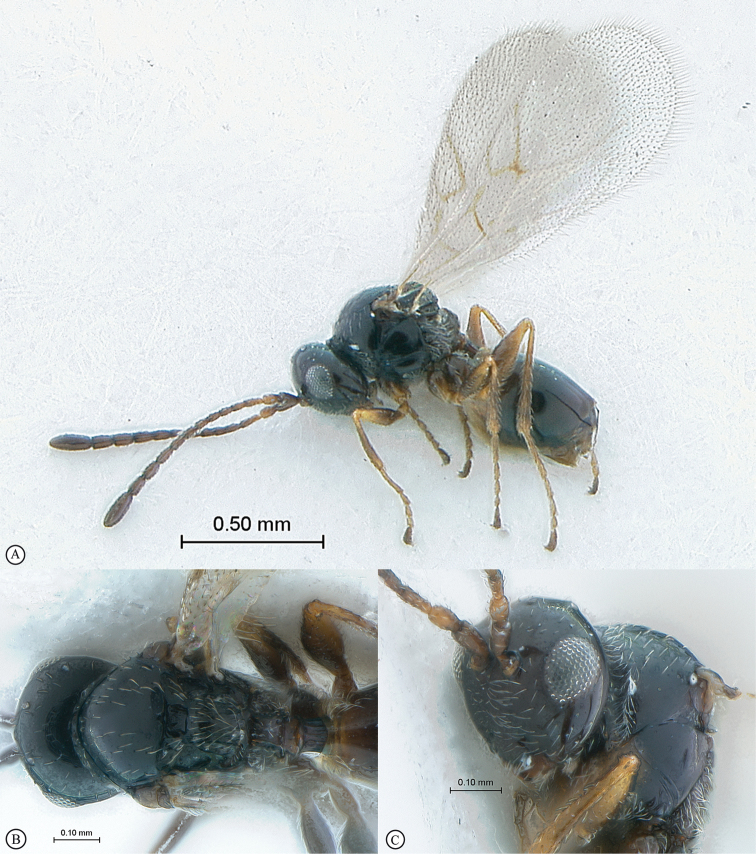
*Lonchidia clavicornis* (South Africa). **A** habitus lateral view **B** head and mesosoma dorsal view **C** head anterior lateral view.

##### Distribution.

Mostly an Holarctic genus, here reported for the first time from the Afrotropical region. Afrotropical records: South Africa (here).

##### Biology.

No host records exist. Hosts are expected to be saprophagous Brachycera larvae.

##### Species richness.

*Lonchidia clavicornis* Thomson, 1862 (South Africa)

#### Neralsia

Taxon classificationAnimaliaHymenopteraFigitidae

Cameron, 1883

##### Remarks.

Rare in the Afrotropical region. The genus is extremely species-rich in the Neotropical region and has recently been revised in a series of papers by Jiménez et al. (2004, 2005a, 2005b, 2005c, 2006, 2007, 2008a, 2008b); Jiménez and Pujade-Villar (2009); Petersen-Silva and Pujade-Villar (2010); Petersen-Silva et al. (2010) and Pujade-Villar et al. (2006). *Neralsia* is also common throughout the Nearctic Region, but species limits have not been thoroughly established (Buffington pers. obs.).

##### Diagnosis.

*Neralsia* and *Xyalophora* are the only known figitines in the Afrotropical region with a scutellar spine. *Neralsia* can be distinguished from *Xyalophora* by whether or not the notauli are horizontally striate: smooth in *Neralsia*, striate in *Xyalophora* (Jimenez et al. 2008). Also, most *Neralsia* have a longer, more robust scutellar spine than *Xyalophora*, but in specimens we have examined, this character varies with overall size of the specimen. This genus also resembles some members of Aspicerinae, most notably *Prosaspicera*, which also possess a distinct scutellar spine, but can be separated from *Prosaspicera* by the lack of a facial impression on the head (present in *Prosaspicera*), and lack of a ligulate metasoma T2.

**Figure 47.**
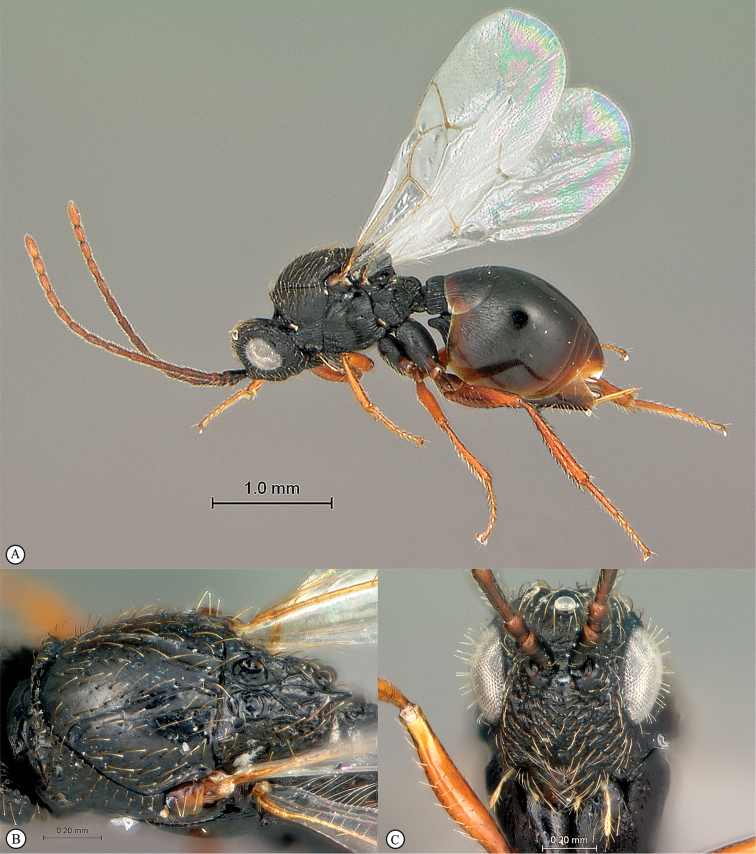
*Neralsia haddocki* (Holotype, Central African Republic). **A** habitus lateral view **B** mesosoma dorsolateral view **C** head anterior view.

##### Distribution.

Mainly Neotropical, but with single species in the Nearctic and the Afrotropical regions. Purported records from the Oriental region and the east Palearctic are unconfirmed. Afrotropical records: Central African Republic, South Africa (van Noort et al. 2014).

##### Biology.

Parasitoids of calyptrate Brachycera larvae in decomposing substrates (Díaz et al. 2000, Thomas and Morgan 1972, Buffington et al. 2012).

##### Species richness.

*Neralsia haddocki* van Noort, Buffington & Forshage, 2014 (Central African Republic, South Africa)

#### Xyalophora

Taxon classificationAnimaliaHymenopteraFigitidae

Kieffer, 1901

##### Remarks.

Rare. Recently revised by Jimenez et al. (2008) and van Noort et al. (2014).

##### Diagnosis.

*Xyalophora* shares the presence of a scutellar spine with *Neralsia*, absent in *Figites* and *Lonchidia*. *Xyalophora* can be separated from *Neralsia* by the presence of transversely striate notauli (smooth in *Neralsia*), and an often slightly smaller scutellar spine; this second character, however, is often linked to adult body size and should be used with caution. As in the case of *Neralsia*, species of *Xyalophora* can be superficially similar to *Prosaspicera* (Aspicerinae), but can be separated from that taxon by the lack of a facial impression on the head, as well as the lack of a ligulate metasomal T2. All three African species have the occipital carinae directed towards the ocellar area and separated in the middle by a smooth surface as well as a smooth interocellar area.

**Figure 48.**
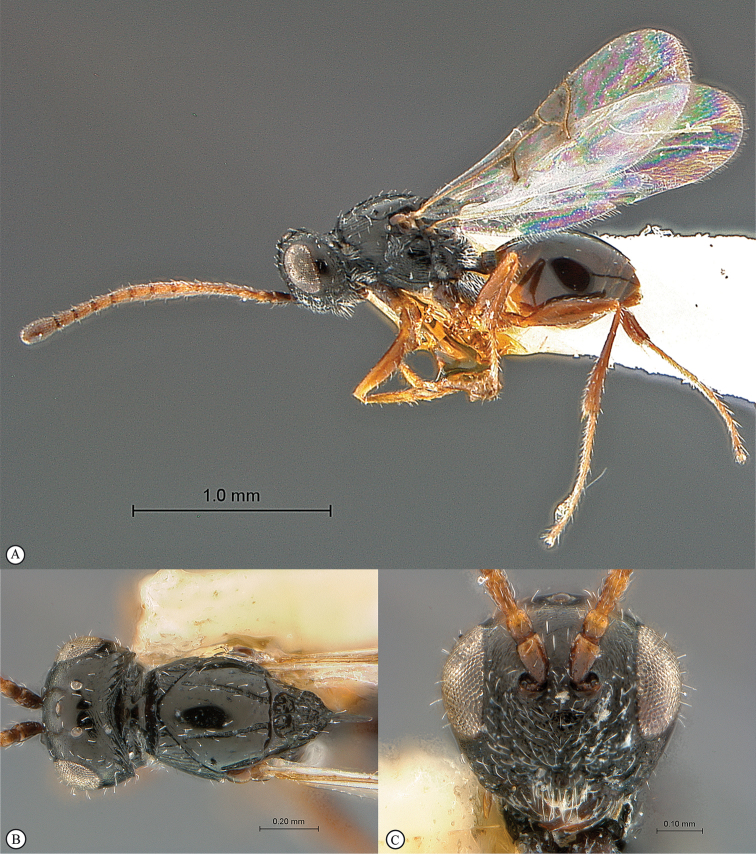
*Xyalophora tedjoansi* (Mali). **A** habitus lateral view **B** head and mesosoma dorsal view **C** head, anterior view.

##### Distribution.

Probably worldwide, but no records from the Oriental region are published. Afrotropical records: Burkina Faso (Jiménez et al. 2008c); Democratic Republic of Congo, Mali, Namibia, South Africa (van Noort et al. 2014).

##### Biology.

Parasitoids of calyptrate Brachycera larvae in decomposing substrates (Ionescu 1969).

##### Species richness.

*Xyalophora tedjoansi* van Noort, Buffington & Forshage, 2014 (Mali)

*Xyalophora provancheri* Jiménez & Pujade-Villar, 2008 (Burkina Faso)

*Xyalophora tintini* van Noort, Buffington & Forshage, 2014 (Democratic Republic of Congo, Namibia, South Africa)

### Pycnostigminae

The Pycnostigminae are represented in the Afrotropical region by two genera containing eight described species. Revised by Buffington and van Noort (2007). A number of undescribed species have since been collected (SAMC).

**Biology.** Unknown. Available phylogenetic evidence suggests that pycnostigmines may be parasitoids of gall-inducing Hymenoptera (Buffington et al. 2012).

**Distribution.** The subfamily is restricted to the Afrotropical and Palearctic regions (Buffington and van Noort 2007).

#### Key to Afrotropical pycnostigmine genera

(after Buffington and van Noort 2007)

**Table:** 

	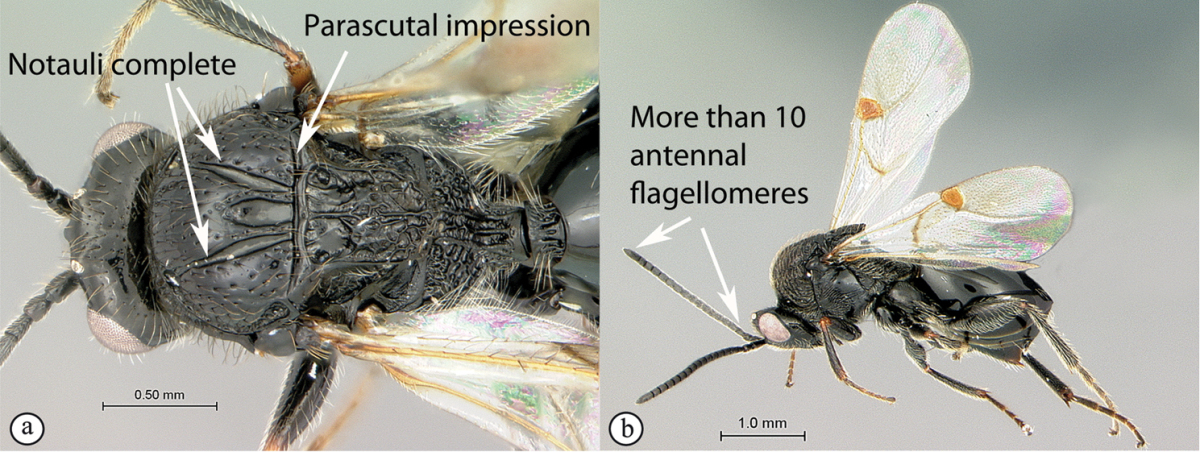	
1	Notauli complete from anterior margin of mesoscutum (at junction with pronotum) to posterior margin (at junction with scutellum) (a); parascutal impression present (a); female with more than 10 flagellomeres (b)	***Pycnostigmus***
	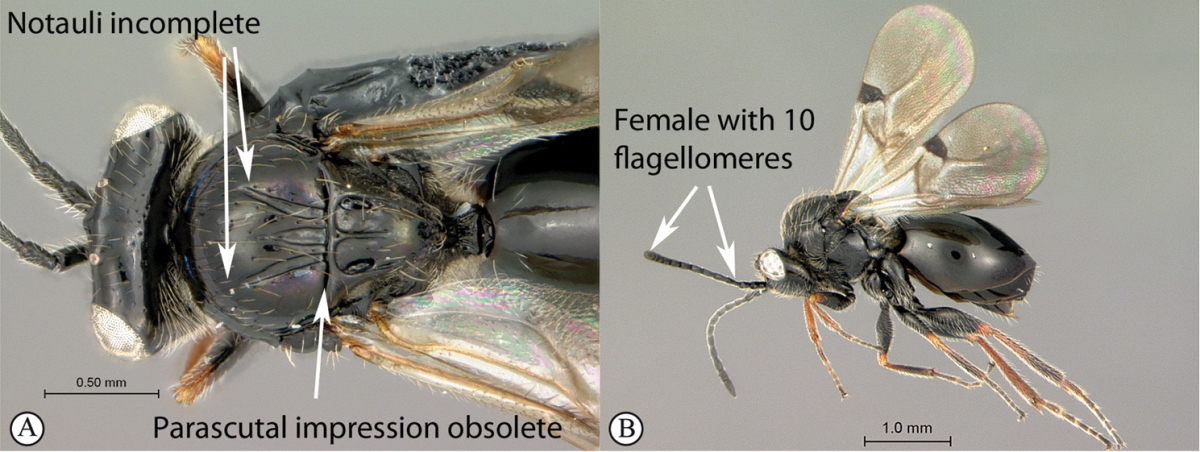	
–	Notauli incomplete, not reaching the pronotum, but present midway across mesoscutum and continuing to junction with scutellum (A); parascutal impression absent (A); female with 10 flagellomeres (B)	***Tylosema***

#### Pycnostigmus

Taxon classificationAnimaliaHymenopteraFigitidae

Cameron, 1905

##### Remarks.

Revised by Buffington and van Noort (2007).

##### Diagnosis.

Within the Afrotropical region, *Pycnostigmus* can be confused with *Tylosema*. However, *Pycnostigmus* species lack notauli on the mesoscutum, an easily observable and reliable character. Outside the Afrotropical region, *Pycnostigmus* is more readily confused with *Trjapitziniola*; presently, this latter taxon is only known from the Palearctic region (Armenia, UAE), but it may be present in the Afrotropical region.

**Figure 49.**
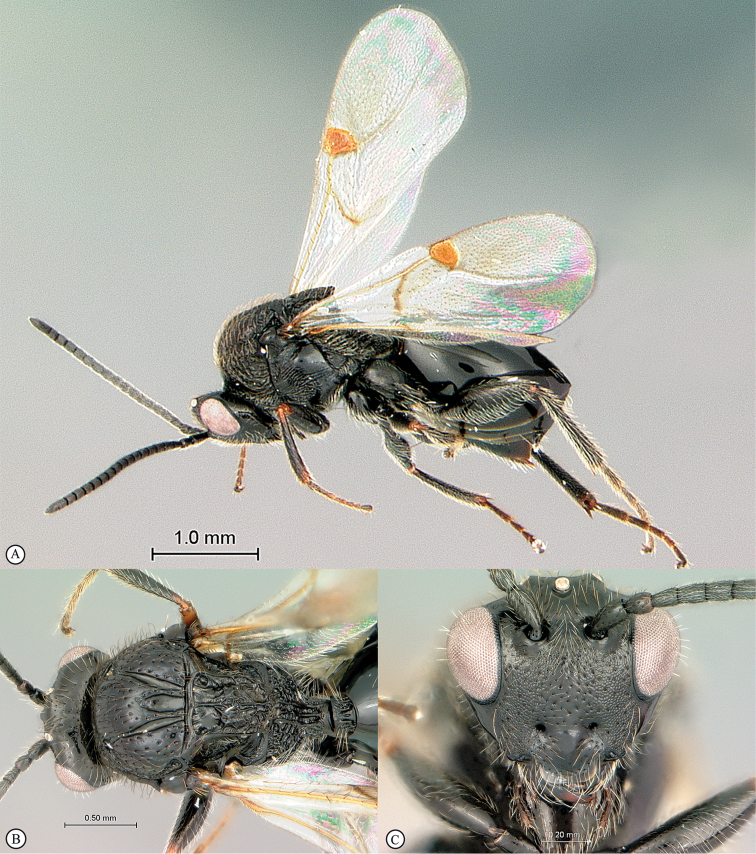
*Pycnostigmus rostratus* (South Africa). **A** habitus lateral view **B** head and mesosoma dorsal view **C** head, anterior view.

##### Distribution.

Endemic to South Africa (Buffington and van Noort 2007).

##### Biology.

Hosts unknown. The phylogeny of Buffington et al. (2012) suggest the host might be a gall-inducing hymenopteran.

##### Species richness.

*Pycnostigmus fossilensis* Buffington & van Noort, 2007 (South Africa)

*Pycnostigmus hoerikwaggoensis* Buffington & van Noort, 2007 (South Africa)

*Pycnostigmus incognito* Buffington & van Noort, 2007 (South Africa)

*Pycnostigmus mastersonae* Buffington & van Noort, 2007 (South Africa)

*Pycnostigmus rostratus* Cameron, 1905 (South Africa)

#### Tylosema

Taxon classificationAnimaliaHymenopteraFigitidae

Kieffer, 1905

##### Remarks.

Revised by Buffington and van Noort (2007).

##### Diagnosis.

Within the Afrotropical region, *Tylosema* can be confused with *Pycnostigmus*. However, *Pycnostigmus* lack notauli on the mesoscutum, and *Tylosema* have complete notauli; this is very easy to observe and the character is reliable. Outside the Afrotropical region, *Tylosema* could be confused with the Palearctic *Trjapitziniola*, but this latter taxon lacks notauli, and has not been recorded from Africa (but see above). *Tylosema nigerrimum* was taken in Algeria, indicating this taxon is present in Mediterranean Africa.

**Figure 50.**
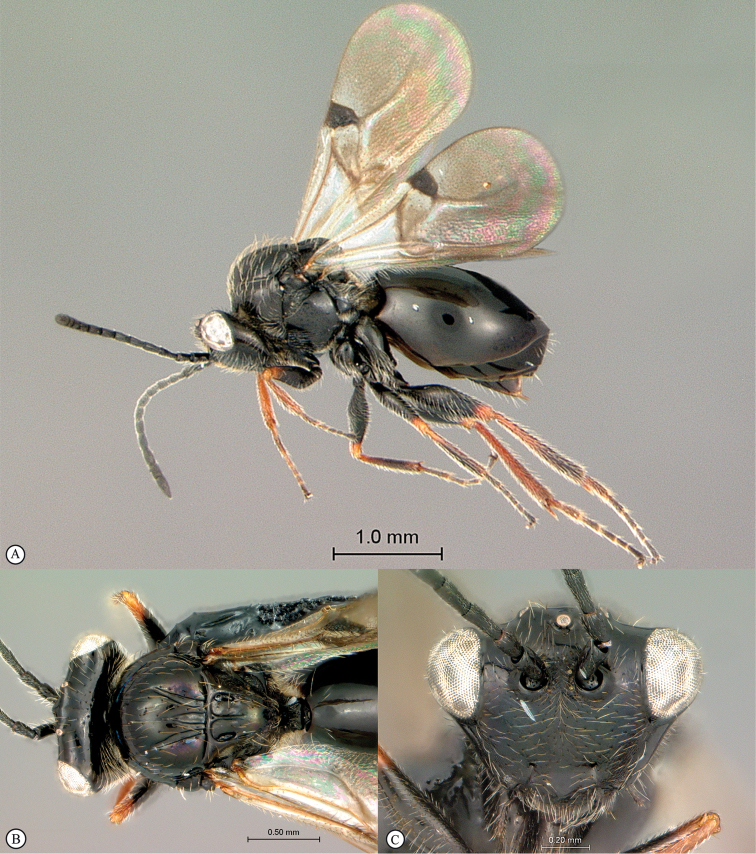
*Tylosema dayae* (South Africa). **A** habitus lateral view **B** head and mesosoma dorsal view **C** head, anterior view.

##### Distribution.

Occurs in South Africa as well as in southwestern Palearctic (north Africa). Afrotropical records: South Africa (Buffington and van Noort 2007).

##### Biology.

Unknown. The phylogeny of Buffington et al. (2012) suggest the host might be a gall-inducing hymenopteran.

##### Species richness.

*Tylosema dayae* Buffington & van Noort, 2007 (South Africa)

*Tylosema ronquisti* Buffington & van Noort, 2007 (South Africa)

## Ibaliidae

The Ibaliidae is represented in the Afrotropical region by a single introduced species.

**Biology.** See below under *Ibalia*.

**Distribution.** The family is represented in nearly all biogeographical regions with the majority of species occurring in the northern hemisphere (Nordlander et al. 1996). Introduced for biological control into Australia and South America (Nordlander et al. 1996).

### Ibalia

Taxon classificationAnimaliaHymenopteraIbaliidae

Latreille, 1802

#### Remarks.

In the Afrotropical region, the Ibaliidae are represented by an introduced Holarctic species. It was introduced to Australia, New Zealand and South Africa (1995–2007) to control *Sirex noctilio* Fabricius, 1793, a pest in pine plantations (*Pinus radiata* D. Don.) (van Noort and Picker 2011).

#### Diagnosis.

Readily distinguished from all other Afrotropical Cynipoidea by the sheer size of this wasp. Adults easily reach 1.5 cm; the closest, by size, to this group of wasps are some species of *Oberthuerella*, especially *Oberthuerella lenticularis* and *Oberthuerella cyclopia*. An additional character that readily seperates *Ibalia* from other cynipoids (and most other Hymenoptera, for that matter), is the extremely laterally flattened mesosoma. In dorsal view, the metasoma of *Ibalia* is blade-like, housing a long, coiled ovipositor. The large liopterids, mentioned above, all have a distinctly ovate metasoma, never laterally flattened. As in the case of the liopterids, ibaliids have a distinctly horizontally strigate mesoscutum, which is hypothesized to be critical in emerging from their wood-boring hosts (Ronquist 1999).

**Figure 51.**
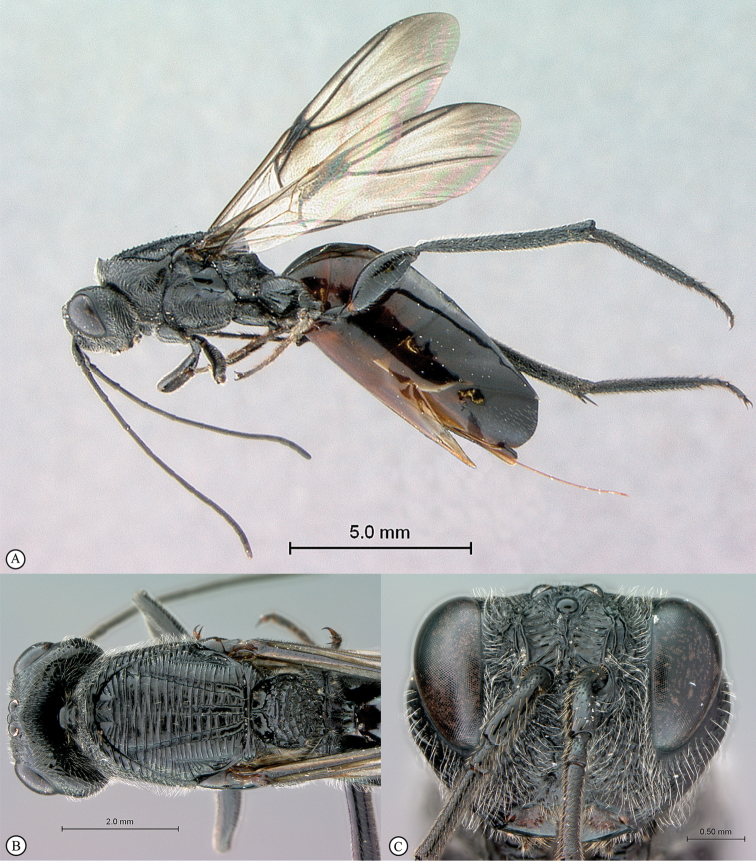
*Ibalia leucospoides* (South Africa). **A** habitus lateral view **B** head and mesosoma dorsal view **C** head, anterior view.

#### Distribution.

Holarctic and transgressing into the northeast Oriental region, introduced elsewhere. Afrotropical records: South Africa (Hurley et al. 2007).

#### Biology.

Parasitoids of woodwasps: *Sirex*, *Urocerus* and *Xeris* (Siricidae) living in conifers. Males mate with females while they are laying eggs. The female inserts her ovipositor down the tunnel bored in pine trees by the host woodwasp larva, to lay an egg either into the egg of the host or into the young host larva. On hatching the ibaliid wasp larva emerges from the body of the host and feeds externally (Hurley et al. 2007).

#### Species richness.

*Ibalia leucospoides* (Hochenwarth, 1785) (*Ichneumon*) ssp. *leucospoides* Hochenwarth, 1785 (South Africa; extralimital distribution throughout the Holarctic region and introduced elsewhere)

## Liopteridae

The Liopteridae are represented in the Afrotropical region by two of the three world subfamilies: Oberthuerellinae and Mayrellinae, with the former having been recently revised by Buffington and van Noort (2012) and the latter by Liu et al. (2007) and van Noort and Buffington (2013). A key to Afrotropical liopterid genera was published in Buffington and van Noort (2012).

**Biology.** The biology of the Liopteridae is unknown, though a few published observations suggest hosts could be Coleoptera in rotting wood: two species of *Kiefferiella* Ashmead emerged from logs infested with buprestids (*Acmaeodera pulchella* (Herbst)) (Weld 1956); a *Kieferiella* species and a *Paramblynotus* Cameron species were reared from trees in the family Fabaceae, *Prosopis glandulosa* Torr. and *Dalberghia fusca* Pierre, respectively (Ronquist 1995a). These associations are all for representatives of Mayrellinae with no records available for Oberthuerellinae. No verified host records exist for Liopteridae (Buffington et al. 2012, Buffington and van Noort 2012).

**Distribution.** The family is represented in all biogeographical regions except for the Western Palaearctic with the majority of species occurring in tropical or subtropical regions (Liu et al. 2007, Ronquist 1995a). The subfamily Liopterinae is restricted to the New World and is centered in the Neotropical region with a few species extending north into the Nearctic region (Ronquist 1995a).

### Key to Afrotropical liopterid subfamilies and genera

(after Buffington and van Noort 2012)

**Table:** 

	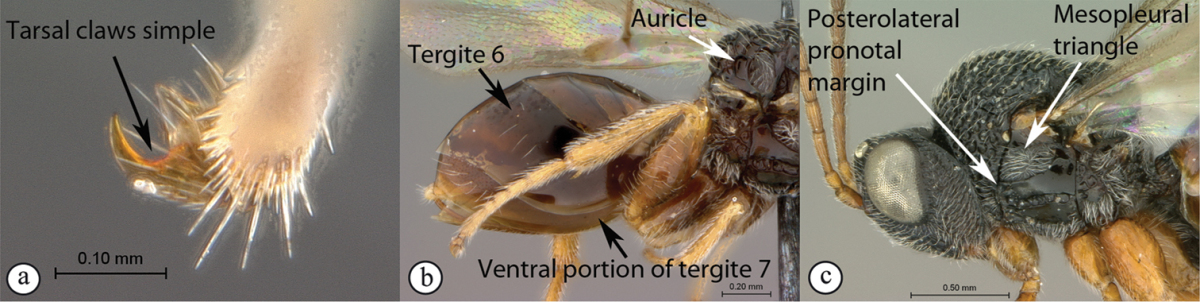	
1	Tarsal claws simple (a). Scutellum with auricula (laterally with semilunar, slightly impressed area set off by distinct carina) (b). Metasomal tergite 6 of females longer dorsally than ventrally in lateral view, posteroventral margin sinuate, strongly curving forward in lateral view, and not covering ventral portion of T7 (b). Posterolateral pronotal margin not incised (c), mesopleural triangle not deeply impressed anteriorly (c) (**Mayrellinae**)	***Paramblynotus***
	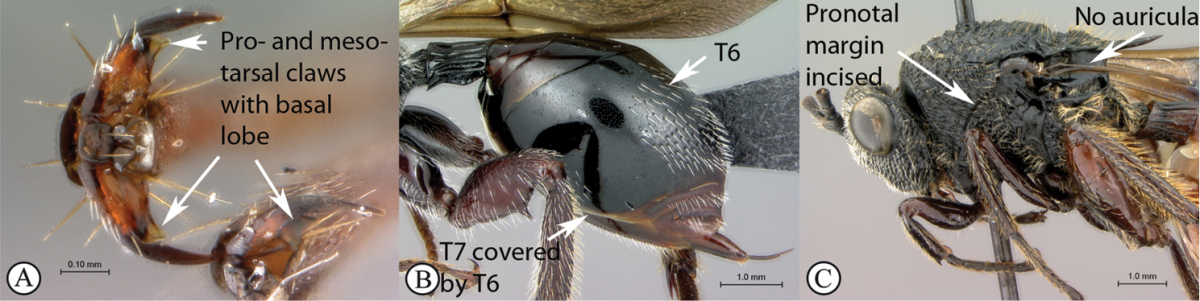	
–	Pro- and mesotarsal claws with basal, lamellate lobe (A). Scutellum laterally without auricula impressed (C). Metasomal tergite 6 of females as long ventrally as dorsally in lateral view (B); posterior margin straight to gently curved in lateral view (B); T6 covering ventral portion of T7 (B). Posterolateral pronotal margin distinctly incised in front of mesopleural triangle, the latter deeply impressed anteriorly (C) (**Oberthuerellinae**)	**2**
	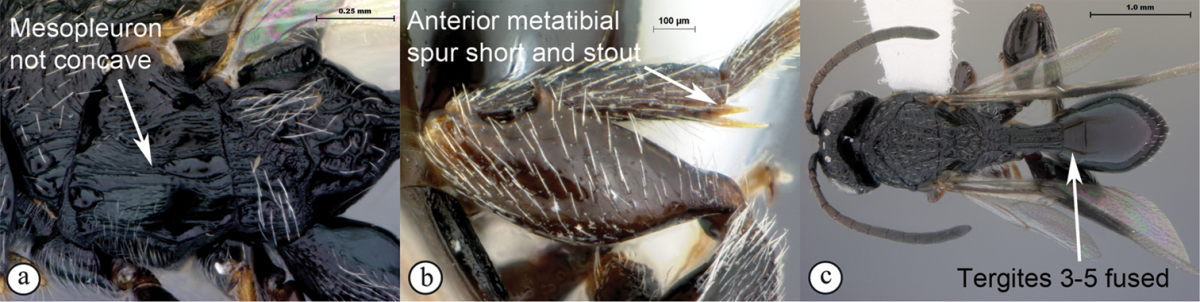	
2	Mesopleural surface not concave (a); mesopleural impression present; lower pleuron at least partly horizontally strigate. Anterior metatibial spur short and stout (b). Metasomal terga 3–5 fused (c), inter-tergal sutures at least partly invisible	***Xenocynips***
	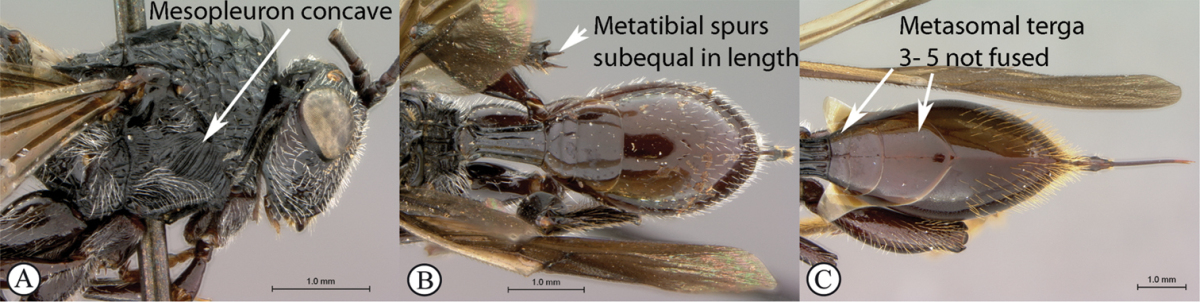	
–	Mesopleural surface distinctly concave, the concavity forming oblique, shallow femoral groove (A); mesopleural impression absent; lower pleuron without horizontal, linear sculpture. Metatibial spurs subequal in length, elongate (B). Metasomal terga 3–5 not fused, inter-tergal sutures distinct (C)	**3**
	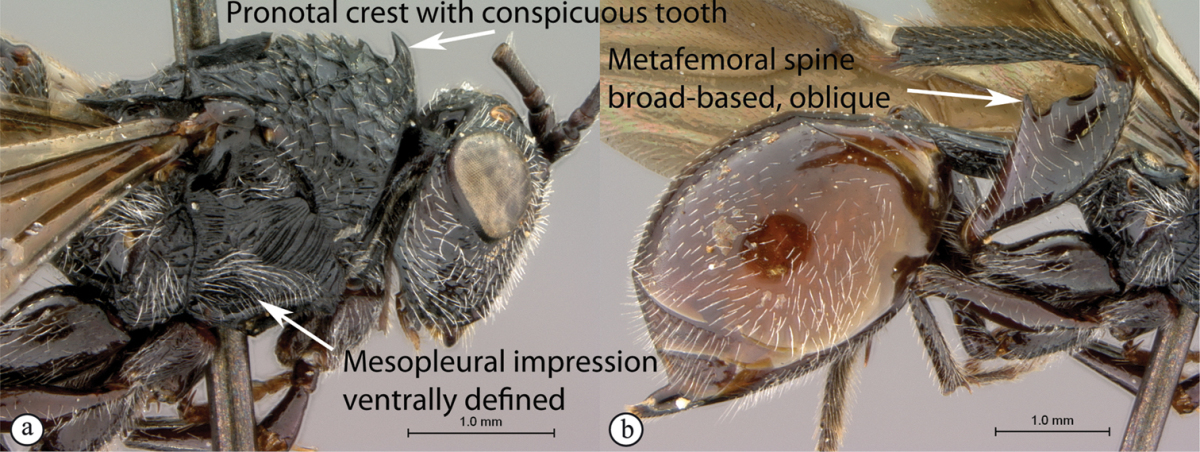	
3	Pronotal crest produced into conspicuous toothlike process (a). Ventral margin of mesopleural impression visible as well-defined ventral margin of obliquely costate area of mesopleuron (a). Metanotal trough absent. Metafemoral spine triangular, broad-based, oblique (b)	***Tessmannella***
	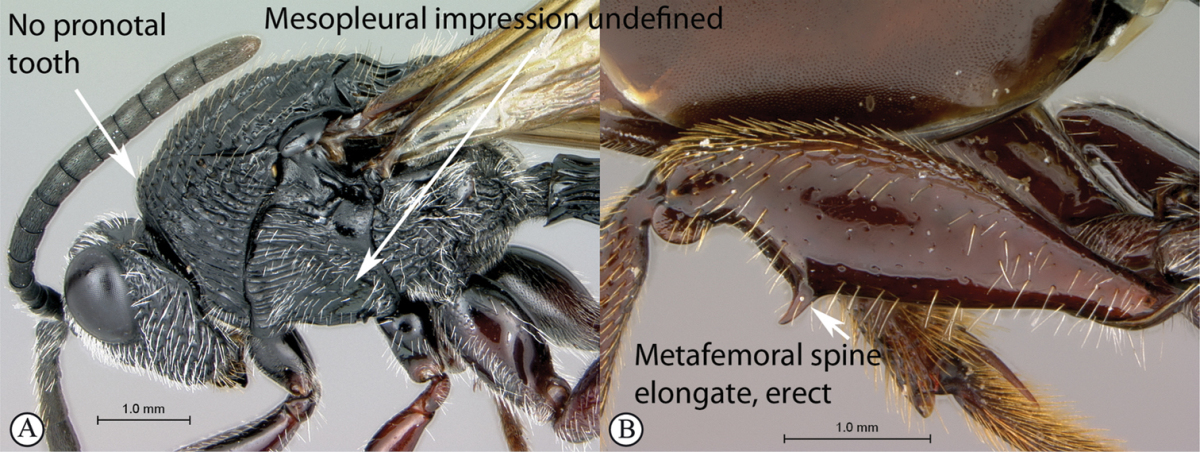	
–	Pronotal crest not produced into conspicuous toothlike process (A), but occasionally produced into small, triangular process. Ventral margin of mesopleural impression not marked (A). Metanotal trough clearly indicated. Metafemoral spine elongate, narrow-based, erect (B)	***Oberthuerella***

### Mayrellinae

Globally this subfamily is represented by two genera, *Kiefferiella* and *Paramblynotus*, with the latter genus occurring in the Afrotropical region (Ronquist 1995a, Liu et al. 2007, van Noort and Buffington 2012).

#### Paramblynotus

Taxon classificationAnimaliaHymenopteraLiopteridae

Cameron, 1908

Paramblynotus (synonyms: *Paraegilips* Kieffer, 1910a, *Allocynips* Kieffer, 1914, *Holocynips* Kieffer, 1916, *Diholocynips* Rohwer & Fagan, 1917, *Mayrella* Hedicke, 1922, *Paribalia* Weld, 1922, *Stylobrachys* Belizin, 1951, *Baviana* Barbotin, 1954, *Decellea* Benoit, 1956b)

##### Remarks.

The genus was recently revised by Liu et al. (2007) and van Noort and Buffington (2013). The latter paper described a further 9 species, including a new species group from Madagascar. *Paramblynotus* species are rare in collections.

##### Diagnosis.

Medium sized to very small cynipoids. Very small species look superfically like cynipids, but careful attention to the relative size of the metasomal terga will help seperate *Paramblynotus* from cynipids. Some superficially resemble figitids, especially Thrasorinae (not found in Africa), but can be separated from the latter by having a deeply foveate pronotum and mesoscutum, as well as diagnostically liopterid metasomal terga. Within Afrotropical Liopteridae, *Paramblynotus* can be distinguished by lacking any scutellar armament, by the lack of any sort of lobe at the base of the tarsal claws, and the presence of an auricula on the side of the scutellum.

**Figure 52.**
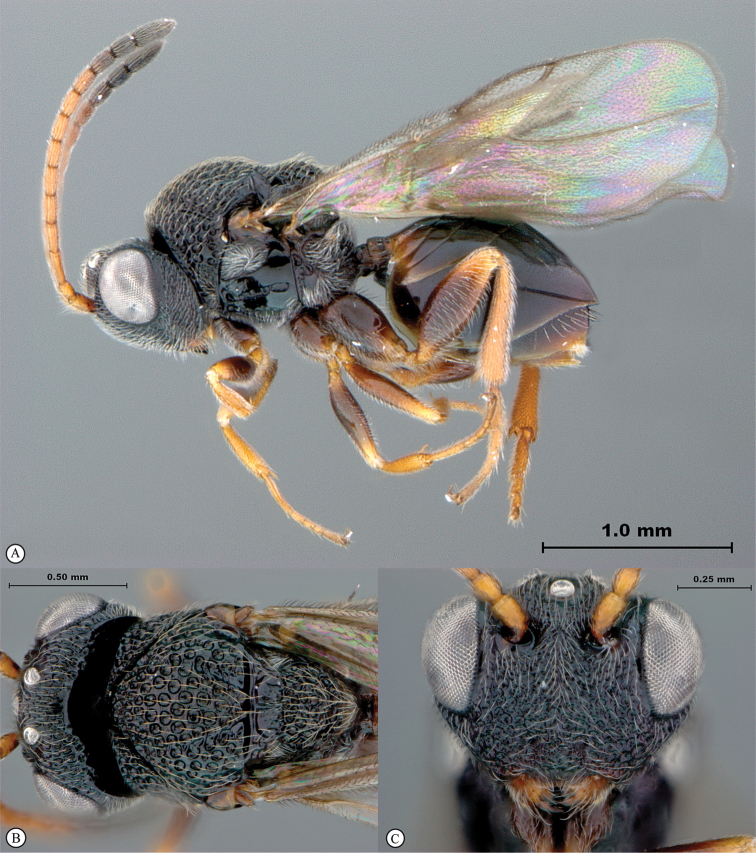
*Paramblynotus parinari* (Central African Republic). **A** habitus lateral view **B** head and mesosoma dorsal view **C** head, anterior view.

##### Distribution.

The genus is represented in all biogeographical regions except for the Western Palaearctic and Australia (Liu et al. 2007, Ronquist 1995a). Three species groups are present in, and endemic to the Afrotropical region: the *Paramblynotus trisetosus* and *Paramblynotus yangambicolus* species groups (two of the seven species groups recognized by Liu et al. (2007)) and the *Paramblynotus seyrigi* species group erected by van Noort and Buffington (2013).

##### Biology.

The type female of *Paramblynotus yangambicola* was captured on a *Drypetes gossweileri* S. Moore (Euphorbiaceae) log in Democratic Republic of Congo (Zaire) (Benoit 1956b). Two females of *Paramblynotus yangambicola* from Uganda are labelled “ex Coleoptera”; two other females from Uganda are labeled “ex Lepidoptera” (Ronquist 1995). Inferred association with Lepidoptera and Coleoptera and rearing of *Paramblynotus yangambicolous* from a rotten log, suggest that *Paramblynotus* species are parasitoids of beetle larva (Liu et al. 2007). The extensive backward pointing ridges on the pronotum and mesoscutum in a number of species suggest an adaption for exiting from (or burrowing in to find) concealed hosts in a confined substrate such as dense leaf litter or rotten logs (van Noort and Buffington 2013). Ronquist (1995) proposed that these structures help with host tunnel negotiation. These effective backward pointing teeth would facilitate the negotiation of such substrates, preventing slippage and promoting forward movement down the tunnels or through the substrate.

#### *Paramblynotus seyrigi* species group

Erected by van Noort and Buffington (2013) to accommodate a single species that is likely to be a Madagascan endemic.

**Diagnosis.** *Paramblynotus seyrigi* has closest affinities with the two Oriental species groups *Paramblynotus ruficollis* and *Paramblynotus punctulatus* of Liu et al. (2007). The *Paramblynotus seyrigi* species group shares the sculptural arrangement of the vertex (large ocelli with three distinct carina extending to or between the toruli) with the two aforementioned Oriental species groups, but the lack of an occipital carina in combination with an absence of a pronotal crest or tooth (uniquely the posterior pronotal margin is represented by a swollen rim), reduced sculpture on the mesoscutum and a unique scutellar foveal character state separate it from these two groups. It is distinct from the two African species groups *Paramblynotus yangambicolus* and *Paramblynotus trisetosus* in a number of characters including a glabrous mesopleuron without sculpture, F1 equal in length to F2, and the presence of an angled latero-ventral pronotal margin.

**Species richness.**

*Paramblynotus seyrigi* van Noort & Buffington, 2013 (Madagascar)

#### *Paramblynotus trisetosus* species group

This is the most species rich group within the Afrotropical region with 28 described species (Liu et al. 2007, van Noort and Buffington 2013). The species group is only known from the African mainland.

**Diagnosis.** Species in this group are typically smaller than those in other species groups, and are the easiest to confuse with Figitidae. They are characterized by having a flat pronotal crest (or, pronotal crest absent); the mesoscutum is foveate-reticulate or with continuous transverse carina with fovea set in rows looking like saw teeth in lateral view; in most species, the speculum is perfectly smooth (gently striate in *Paramblynotus vannoorti*); and the median propodeal area is distinctly delimited by lateral propodeal carinae, and posteriorly is not foveoate-reticulate. Careful attention to the metasomal sclerites will prevent confusing *trisetosus*-group *Paramblynotus* with Figitidae.

**Species richness.**

*Paramblynotus alexandriensis* Buffington & van Noort, 2013 (South Africa)

*Paramblynotus angolensis* Liu, Ronquist & Nordlander, 2007 (Angola)

*Paramblynotus antistatus* Liu, Ronquist & Nordlander, 2007 (Democratic Republic of Congo)

*Paramblynotus bayangensis* van Noort & Buffington, 2013 (Central African Republic)

*Paramblynotus cameroonensis* Liu, Ronquist & Nordlander, 2007 (Cameroon)

*Paramblynotus carinatus* Liu, Ronquist & Nordlander, 2007 (Democratic Republic of Congo)

*Paramblynotus claripennis* Liu, Ronquist & Nordlander, 2007 (Uganda)

*Paramblynotus coxatus* Liu, Ronquist & Nordlander, 2007 (South Africa)

*Paramblynotus diminutus* Liu, Ronquist & Nordlander, 2007 (Zimbabwe)

*Paramblynotus dzangasangha* van Noort & Buffington, 2013 (Central African Republic)

*Paramblynotus femoratus* Liu, Ronquist & Nordlander, 2007 (South Africa)

*Paramblynotus fuscicapiculus* Liu, Ronquist & Nordlander, 2007 (South Africa, Zimbabwe)

*Paramblynotus immaculatus* Liu, Ronquist & Nordlander, 2007 (Namibia)

*Paramblynotus jacksoni* Liu, Ronquist & Nordlander, 2007 (Cameroon)

*Paramblynotus kekenboschi* Liu, Ronquist & Nordlander, 2007 (Democratic Republic of Congo)

*Paramblynotus maculipennis* Liu, Ronquist & Nordlander, 2007 (Democratic Republic of Congo)

*Paramblynotus matele* van Noort & Buffington, 2013 (Central African Republic, Democratic Republic of Congo)

*Paramblynotus minutus* Liu, Ronquist & Nordlander, 2007 (South Africa)

*Paramblynotus nigricornis* Benoit, 1956b (Democratic Republic of Congo)

*Paramblynotus parinari* Buffington & van Noort, 2013 (Kenya, Uganda)

*Paramblynotus prinslooi* Liu, Ronquist & Nordlander, 2007 (South Africa)

*Paramblynotus ruvubuensis* van Noort & Buffington, 2013 (Burundi)

*Paramblynotus rwandensis* Liu, Ronquist & Nordlander, 2007 (Rwanda)

*Paramblynotus samiatus* Liu, Ronquist & Nordlander, 2007 (South Africa)

*Paramblynotus scalptus* Liu, Ronquist & Nordlander, 2007 (South Africa)

*Paramblynotus townesorum* Liu, Ronquist & Nordlander, 2007 (South Africa)

*Paramblynotus trisetosus* Benoit, 1956b (Democratic Republic of Congo)

*Paramblynotus vannoorti* Liu, Ronquist & Nordlander, 2007 (Kenya, South Africa, Tanzania)

*Paramblynotus zairensis* Liu, Ronquist & Nordlander, 2007 (Democratic Republic of Congo)

#### Paramblynotus yangambicolus species group

Previously only known from the African mainland with three described species (Liu et al. 2007); van Noort and Buffington (2013) described two species from Madagascar.

**Diagnosis.** This species group is characterized in females by excavations (spiracular peritremata) on the terminal portion of T8 associated with the spiracle. A distinct pronotal crest is present, medially forming a conspicuous, slightly backward pointing, ridge-like tooth. The mesoscutum has rough discontinuous transverse costae produced into irregularly raised and slightly backward pointing teeth. The speculum is longitudinally costate, and the median propodeal area is not delimited by lateral propodeal carinae.

**Species richness.**

*Paramblynotus alveolatus* Liu, Ronquist & Nordlander, 2007 (Cameroon)

*Paramblynotus behara* van Noort & Buffington, 2013 (Madagascar)

*Paramblynotus mixtus* Liu, Ronquist & Nordlander, 2007 (Kenya)

*Paramblynotus yangambicolus* (Benoit, 1956b) (*Decellea*) (Democratic Republic of Congo)

*Paramblynotus zohy* van Noort & Buffington, 2013 (Madagascar)

### Oberthuerellinae

This subfamily is represented by three genera, *Oberthuerella*, *Tessmannella*, and *Xenocynips* all of which are endemic to the Afrotropical region (Ronquist 1995a, van Noort and Buffington 2012).

#### Oberthuerella

Taxon classificationAnimaliaHymenopteraLiopteridae

Saussure, 1890

Oberthuerella Saussure, 1890: plate 20, fig. 20. Type species: *Oberthuerella lenticularis* Saussure, by monotypy.

##### Diagnosis.

*Oberthuerella* can be readily distinguished from *Xenocynips* by having distinct metasomal terga (tergites 3–5) with the inter-tergal sutures not fused. The mesopleuron is also distinctly concave, the concavity forming an oblique, shallow femoral groove; the mesopleural impression is absent and the ventral part of the mesopleuron is without horizontal, linear sculpture; the metatibial spurs are subequal in length, elongate. The lack of a pronotal crest produced into a conspicuous toothlike process easily distinguishes *Oberthuerella* from *Tessmannella*.

**Figure 53.**
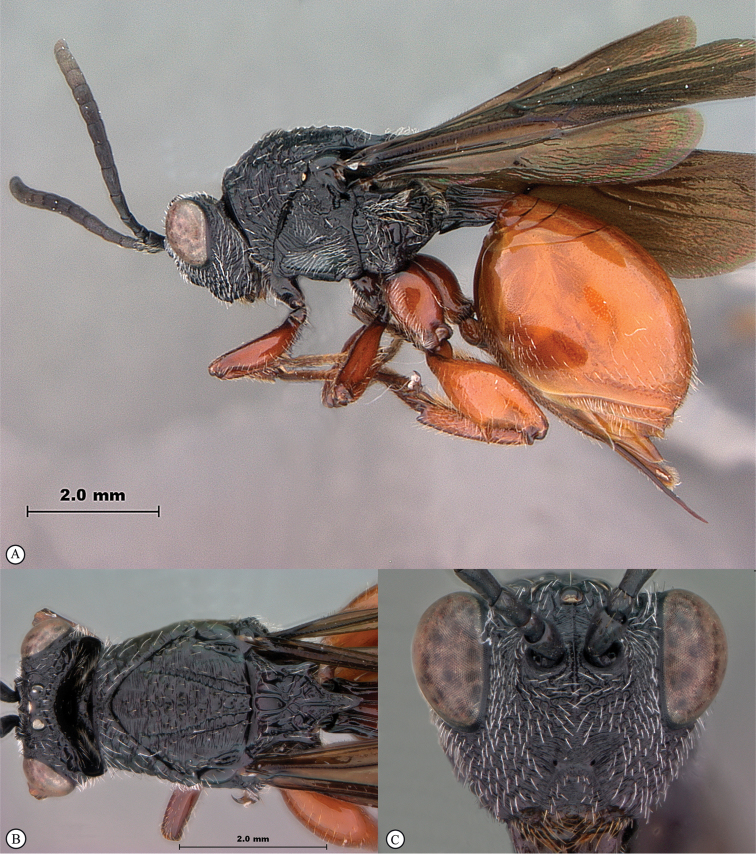
*Oberthuerella sharkeyi* (Republic of Congo). **A** habitus lateral view **B** head and mesosoma dorsal view **C** head, anterior view.

##### Identification.

Dichotomous and online interactive keys to species are available in Buffington and van Noort (2012) and van Noort (2004–2015).

##### Distribution.

Cameroon, Democratic Republic of Congo, Equatorial Guinea, Gabon, Ivory Coast, Kenya, Liberia, Madagascar, Malawi, Republic of Congo, South Africa, Tanzania, Uganda, Zambia, Zimbabwe (Buffington and van Noort 2012).

##### Biology.

Unknown.

##### Species richness.

*Oberthuerella abscinda* Quinlan, 1979 (Democratic Republic of the Congo, Zambia).

*Oberthuerella aureopilosa* Benoit, 1955 (Democratic Republic of the Congo)

*Oberthuerella breviscutellaris* Benoit, 1955 (Democratic Republic of Congo, Kenya, Zimbabwe)

*Oberthuerella crassicornis* Benoit, 1955 (Democratic Republic of Congo, Malawi).

syn *Oberthuerella compressa* Benoit, 1955

*Oberthuerella cyclopia* Buffington and van Noort, 2012 (Democratic Republic of Congo)

*Oberthuerella eschara* Buffington and van Noort, 2012 (Liberia)

*Oberthuerella kibalensis* van Noort and Buffington, 2012 (Uganda)

*Oberthuerella lenticularis* Saussure, 1890 (Ivory Coast, Madagascar, Malawi, South Africa)

*Oberthuerella longicaudata* Benoit, 1955 (Democratic Republic of the Congo)

*Oberthuerella longispinosa* Benoit, 1955 (Democratic Republic of Congo; Gabon; Ivory Coast; Malawi)

*Oberthuerella nigra* Kieffer, 1910b (Equatorial Guinea)

*Oberthuerella nigrescens* Benoit, 1955 (Democratic Republic of the Congo)

*Oberthuerella pardolatus* Buffington and van Noort, 2012 (Democratic Republic of the Congo)

*Oberthuerella sharkeyi* Buffington and van Noort, 2012 (Republic of the Congo)

*Oberthuerella simba* Buffington and van Noort, 2012 (Democratic Republic of the Congo)

*Oberthuerella tibialis* Kieffer, 1904 (Cameroon, South Africa; Zimbabwe)

*Oberthuerella transiens* (Benoit, 1955) (*Tessmanella*) (Democratic Republic of the Congo)

*Oberthuerella triformis* Quinlan, 1979 (Tanzania)

#### Tessmannella

Taxon classificationAnimaliaHymenopteraLiopteridae

Hedicke, 1912

##### Diagnosis.

Female antenna 13–segmented, subclavate; male 14–segmented. Face with reticulate to rugose sculpture and scattered pubescence. Pronotum coarsely rugose with median tooth or spine viewed laterally. Mesonotum with coarse variable sculpture, propodeum without pronounced side margins. Segment 1 of metasoma (petiole) three times as long as broad, segments 2–4 short viewed laterally and dorsally, segment 5 the largest. Metafemora with a rounded lobe between medial area and apex, tooth on metafemur angled, hind tibia with a distinct lobe apically, opposite the tibial spines. Scutellum with three foveae.

**Identification.** Dichotomous and online interactive keys to species are available in Buffington and van Noort (2012) and van Noort (2004–2015).

**Figure 54.**
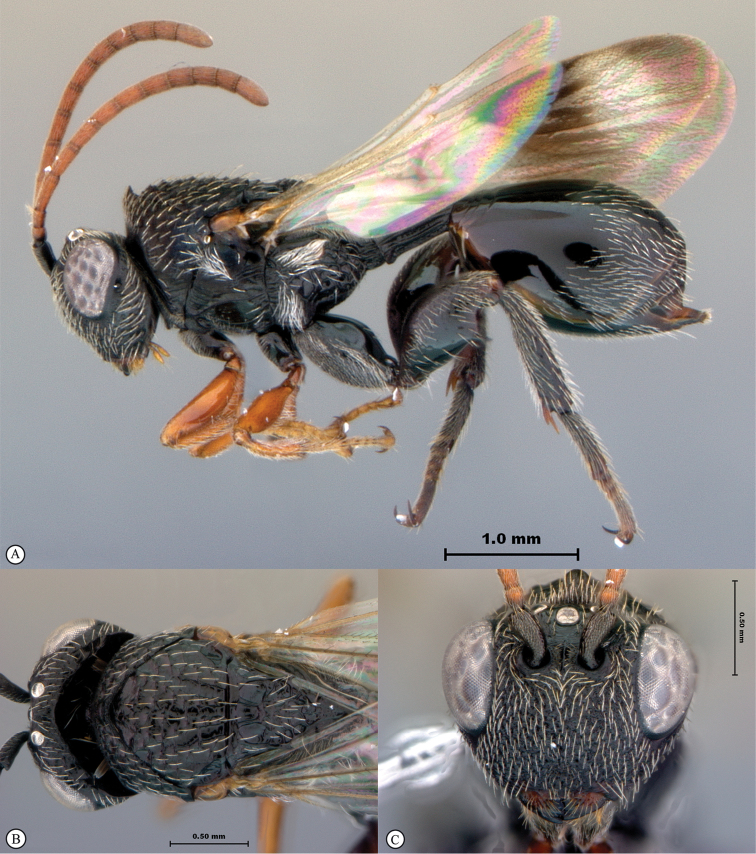
*Tessmannella kiplingi* (Republic of Congo). **A** habitus lateral view **B** head and mesosoma dorsal view **C** head, anterior view.

##### Distribution.

Central African Republic, Democratic Republic of Congo, Equatorial Guinea, Gabon, Kenya, Republic of Congo (Buffington and van Noort 2012).

##### Biology.

Unknown.

##### Species richness.

*Tessmannella copelandi* Buffington & van Noort, 2012 (Kenya)

*Tessmannella expansa* Quinlan, 1979 (Gabon)

*Tessmannella kiplingi* Buffington & van Noort, 2012 (Republic of Congo)

*Tessmannella nigra* Hedicke, 1912 (Democratic Republic of Congo, Equatorial Guinea)

*Tessmannella roberti* Buffington & van Noort, 2012 (Central African Rebublic)

*Tessmannella spinosa* Hedicke, 1912 (Equatorial Guinea)

#### Xenocynips

Taxon classificationAnimaliaHymenopteraLiopteridae

Kieffer, 1910a

##### Diagnosis.

Metasomal terga 3–5 fused, with intertergal sutures partially visible; lower mesopleuron horizontally striate. *Tessmannella* is most easily confused with *Xenocynips*; the fusion of terga in *Xenocynips* is a very reliable and clearly visible character. Additionally, most species of *Xenocynips* possess a dorsoventrally striate lateral aspect of the scutellum, posterior to the auricula; this is useful for specimens in which the metasoma is missing.

**Figure 55.**
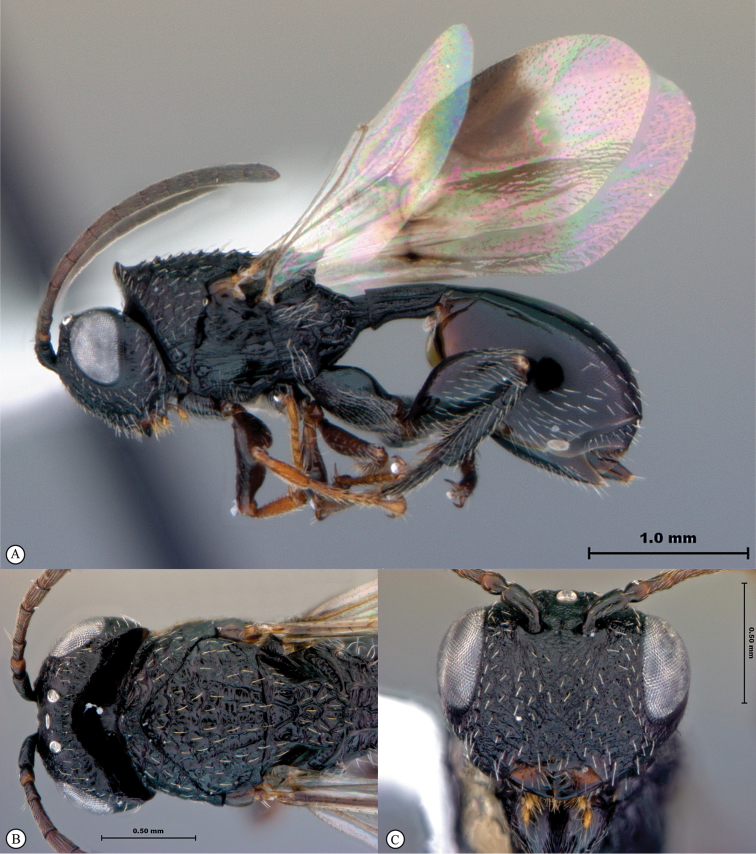
*Xenocynips rhothion* (Central African Republic). **A** habitus lateral view **B** head and mesosoma dorsal view **C** head, anterior view.

##### Identification.

Dichotomous and online interactive keys to species are available in Buffington and van Noort (2012) and van Noort (2004–2015).

##### Distribution.

Cameroon, Central African Republic, Democratic Republic of Congo, Republic of Congo (Buffington and van Noort 2012).

##### Biology.

Unknown.

##### Species richness.

*Xenocynips rhothion* Buffington & van Noort, 2012 (Central African Republic; Republic of Congo).

*Xenocynips ronquisti* Buffington & van Noort, 2012 (Democratic Republic of Congo).

*Xenocynips subsquamata* Kieffer, 1910a (Cameroon)

## Discussion

Hyper-diverse insect groups provide a challenge for identification, particularly from regions where they are poorly studied and where knowledge of generic and species diversity is wanting. Years of frustrated scrabbling through scattered historical literature to find poorly illustrated and out of date keys to identify Afrotropical Hymenoptera led to the formulation of the Afrotropical Hymenoptera Initiative (AHI) to address this hiatus (van Noort et al. 2010). This project will provide a sorely needed synthesised resource to enable the identification of Afrotropical Hymenoptera to generic level, with a summary of described species and biology and introduction to the relevant literature. Richly illustrated user-friendly web-available keys will provide a tool for coping with the phenomenal biological diversity of the region, the production of which will require major international collaboration between specialists across the included taxa. The Cynipoidea were taken on as the first phase of the project since the superfamily is reasonably diverse at generic level and currently actively worked on for the region. Hence ouput was attainable within a reasonable time period to set the approach for the remaining superfamilies. Groups such as the Chalcidoidea and Ichneumonoidea will provide a far greater challenge.

With the completion of this phase of the Afrotropical Hymenoptera Initiative project, the taxonomic knowledge base of cynipoids in the Afrotropical region joins a similar knowledge base for the Western Palearctic and Australian regions, with modern keys to genera available for most cynipoid groups (Fergusson 1986, Forshage and Nordlander 2008, Melika 2006, Nieves-Aldrey 2001, Paretas-Martinez et al. 2011, 2013).

It may be premature to promise the treatment of another major region sometime soon. Since we have observed, and indeed are concluding here, that for Figitidae there is a substantial overlap, with mostly shared genera, between the Afrotropical and Oriental regions, the Oriental region might be the logical next step to address. Nevertheless, major collecting efforts still reveal new genera in Eucoilinae, and for the Cynipidae, the rich fauna of the region is only now being discovered (e.g. Tang et al. 2009, 2011a, 2011b, Melika et al. 2011a, 2011b). When the Oriental fauna is better understood, combining this knowledge with that of the Western Palearctic might possibly make the treatment of the Eastern Palearctic a relatively easy task, but collection efforts from this region are still very scattered and often difficult to access. Additionally, the Oceanic fauna outside of Australia (the Pacific islands) presents a special problem as there are endemic radiations of Eucoilinae that are poorly understood from taxonomic and phylogenetic perspectives. This fauna has only fairly recently been subjected to preliminary assessment (Beardsley 1988, 1989, 1990, 1992a, 1992b) and contrary to early assumptions has been shown, at least partly, to be a part of the shared Paleotropical fauna (Forshage and Buffington pers. obs.) Additionally, circumscription of the Oceanic fauna is further confounded by what appears to be repeated introductions of eucoilines from the west coast of North America (Buffington, pers. obsv.).

The cynipid and figitid diversity are both relatively high in North America, and while Figitidae systematics of the region has become more stable in recent years, including an updated catalogue of Eucoilinae (Forshage et al. 2013), many taxonomic changes are still underway within Cynipidae, making the generation of an updated key to North American genera still some time off (Melika pers. comm.). However, once North America has been treated, the last major region to require extensive assessment is that of the Neotropics. Efforts in the Neotropical region, however, are hampered by a general lack of comprehensive collections, as well as a truly infantile knowledge of diversity with respect to other regions. In a recent trip to Brazil, for instance, MB sorted undetermined Figitidae from a major biodiversity survery in Espirito Santo, recovering an estimated 400 morphospecies and, at the very least, 4 undescribed genera. However, we must note the recent important advances which are being published on cynipoid faunas of Chile (Nieves-Aldrey et al. 2009, Buffington and Nieves-Aldrey 2011); Costa Rica (Pujade et al. 2012); and Panama (Nieves-Aldrey and Medianero 2011, 2013, Medianero and Nieves-Aldrey 2011a, 2011b). While Buffington et al. (2006) provided a key to genera of the Neotropical region, this key itself is out of date, and likely covers only a portion of the actual generic-level diversity (and no species level diversity). Lastly, a major challenge to understanding the Neotropical cynipoid fauna, as in other tropical regions, rests squarely upon habitat destruction and disturbance, inhibiting our gaining knowledge of many species before they become locally rare and/or extinct.

For the Afrotropical region, patterns of cynipoid diversity have become clearer through this project. The most notable is an over-arching distribution pattern of high taxon affinity between the Afrotropical and Oriental regions. This parallels, and is probably influenced by, host specificity across the various trophic levels, driven by the underlying high degree of floral similarity between the two regions. Both regions have their share of endemic genera and species, but they also possess a significant overlap. Two groups stand out immediately: *Paramblynotus* (Liopteridae) and *Afrostilba* (Figitidae). *Paramblynotus* is particularly diverse in both the Afrotropical (van Noort and Buffington 2013) and Oriental regions (Liu et al. 2007), with what appears to be very little species overlap (this study); elsewhere in the world, the genus is either rare (Buffington and Gates 2013) or not nearly as speciose (Liu et al. 2007). The eucoiline *Afrostilba* can often be a dominant taxon in bulk sampling efforts, with many stable morphospecies shared between the Afrotropical (many countries sampled; this study) and the Oriental regions (Thailand, Indonesia, Bangladesh, India; Buffington pers. obsv.). However, until a revision of *Afrostilba* has been completed, the scope and accuracy of taxonomic comparisons across these regions is limited. Several other genera of Eucoilinae have been shown to have a distribution extending across the Afrotropical and Oriental regions, recently or in this paper. Originally described from Taiwan, *Paradiglyphosema*, *Linoeucoila* and *Gastraspis*, occur both in the Afrotropical and the Oriental regions. *Bothrochacis*, *Afrostilba*, *Ealata* and *Nordlanderia* were described from the Afrotropical region, but are now known to occur in both. Even genera such as *Endecameris*, *Micreriodes* and *Leptolamina*, originally described from the Pacific, have been shown to occur both in Asia and in Africa, suggesting that a substantial portion of the fauna is generally Paleotropical. For several of the mentioned genera (*Linoeucoila*, *Gastraspis*, *Bothrochacis*, *Endecameris* and *Micreriodes*), the geographical records allowing these observations are first published here, for others (*Afrostilba*, *Leptolamina*) they are dependent on nomenclatural acts made here.

A few figitid lineages show unique diversification within the Afrotropical region. The Pycnostigminae are one of the most enigmatic cynipoid lineages sub-endemic to the region with 80% of the species restricted to sub-Saharan Africa (two genera *Tylosema* and *Trjapitziniola* are represented by single species in the Mediterranean region). The only known metallic-colored cynipoid, *Pycnostigmus mastersonae*, falls within this unusual group. Worldwide, Emargininae tend to be a rather rarely encountered and collected taxon (Buffington and Forshage pers. obsv.); however, within the Afrotropical region, and particularly Madagascar, the group can often dominate a bulk sample of cynipoids. The species-level diversity within Madagascar is spectacular (as noted above), and this group will make for an exciting species-level revision in the future. The aspicerine *Anacharoides*, and the anacharitine *Acanthaegilopsis*, are both genera unique to the Afrotropical region. Within Eucoilinae, a number of notable endemic or particularly species-rich groups have been recorded over the past three decades and are treated in this paper. Specialised morphological adaptations are uniquely exhibited by a number of Afrotropical taxa. *Angustacorpa* is bizarrely flattened, and *Stentorceps* and *Nanocthulhu* are both characterized by arguably grotesque, unique protrusions from their frons and clypeal regions; the function of which is unknown. Hyperdiverse groups include *Rhoptromeris*, *Hexacola* and *Didyctium*, all of which are common outside the Afrotropical region, but were previously not known to include the species-level diversity observed here. Finally, *Leptopilina* and *Trichoplasta* appear to have a larger diversity in the region than elsewhere.

Since assessment of generic diversity, species richness and distribution of Afrotropical cynipoids is in its infancy, with major gaps in sampling effort and habitat coverage, we can only forward hypothetical conjecture regarding biogeographical patterns based on limited data. Nevertheless, these hypotheses are a starting point and will be tested and revised as we proceed with the documentation of the region’s hymenopteran fauna. Van Noort and Buffington (2013) hypothesized, based on data in Liu et al. (2007) and Buffington et al. (2012), that African *Paramblynotus* diverged from the remaining Palaearctic members of the genus between the late Oligocene to early Miocene periods (26–23 mya). Expanding this schema to Figitidae is certainly within reason, since Buffington et al. (2012) recovered the majority of tribe- to genus-level diversification events within most subfamilies to have occurred at roughly the same time, especially within Eucoilinae. This would result in a few recently diverged, uniquely African groups that potentially evolved and diversified as a result of the aridification of the continent and formation of the savanna biome during the Oligocene-early Miocene c. 33–20 mya (Couvreur et al. 2008, Sepulchre et al. 2006) (e.g. the Oberthuerellinae, Pycnostigminae, the aspicerine *Anacharoides*, and the eucoiline genera, *Stentorceps* and *Nanocthulhu*), as well as a few lineages present in other regions, but with unique species present only in Africa (e.g. *Rhoptromeris* and *Afrostilba*). Buffington et al. (2012), however, did provide weak evidence of a Gondwanian element to the Afrotropical fauna. In that analysis, the thrasorines (southern South America), the mikeiines (Australian) and pycnostigmines (southern Africa) clustered in a clade. However, the posterior probability was very low (69; figure 1), and the stem-group divergence estimates for the group were centered around 75 mya, somewhat young for a Gondwanian explanation of distribution. Another question worth expanding is that of the origin of cynipoids, other than *Paramblynotus*, present on Madagascar. Since there is evidence of Madagascar separating from mainland Africa 160–120 mya (Ali et al. 2008), for groups with large numbers of endemic species, such as the Malagasy Emargininae, some form of dispersal event would be expected to have taken place from the great continent. This observation is reinforced by the fact that Buffington et al. (2012) recovered the emarginines to have a crown group age of 40 mya, making a hypothesis of vicariance from the African mainland unlikely for this group of cynipoids. However, Madagascar’s cynipoid species richness and endemism is likely a combination of numerous historical evolutionary processes, including persistence of paleoendemic lineages and more recent rapid speciation of younger lineages, as holds for many other groups of animals and plants on this island (Vences et al. 2009, Burkei et al. 2013).

Studies on endemic Afrotropical cynipoids (liopterids: Buffington and van Noort 2012, van Noort and Buffington 2013; aspicerines: Buffington and van Noort 2008; eucoilines: Nielsen and Buffington 2011, Buffington 2012; pycnostigmines: Buffington and van Noort 2007) recover a similar set of patterns with respect to distribution: A) a general ‘hydrophilic belt’ of diversity along equatorial Africa, with a ‘southern swath’ southward along the central eastern seaboard, ultimately ending in the more Mediterranean-esque portion of South Africa (aspicerines, some liopterids, many eucoilines and specifically *Stentorceps*); B) a ‘hydro-phobic’ patchy distribution within South Africa, often focused on the Western Cape (but theoretically this should extend into Namibia and Botswana) with linkages to the arid north-eastern areas of Africa and the middle east (pycnostigmines, some liopterids, some eucoilines). However, it should be pointed out that few new samples are being generated from, or are presently in world collections for the majority of the African countries, and hence any assessment of finer scale biogeographical patterns based on current data is premature.

In terms of species richness hot spots the arid Sahel belt paralleling the southern edge of the Sahara desert and extending south down the eastern side of Africa through Ethiopia, Somalia and Kenya to Tanzania and the south-western aridness of Africa may potentially contain a reasonably diverse assemblage of cynipoids (as does the arid southwest of the United States and north Mexico, and arid Central Asia). With continued collecting in South Africa, this region may perhaps prove to be a hot spot of species richness in Africa. The uniqueness of the Cape Floral Kingdom, containing a diverse range of vegetation types (Mucina and Rutherford 2006), coupled with geographic characteristics typified by rugged, highly stratified mountain ridges (Cape Fold Mountain belt) likely played a role in promoting speciation within the group. Likewise the East African Rift valley extending from Ethiopia in the north to Zimbabwe in the south, encompassing the rugged topography of eastern DRC, Rwanda and Burundi, which started formation with upliftment in the Eocene-Oligocene period (Sepulchre et al. 2006), together with the belt of eastern arc mountains in Tanzania, each with isolated Afromontane forest refugia (Burgess et al. 2007) will likely prove to be another rich area of species diversity and endemism, as holds for plants and vertebrates (Myers et al. 2000, Lovett 2005). These eastern arc forests have strong connections with the Guineo-Congolian lowland rainforest and may have been isolated for 30 Myr from the start of the breakup of the pan African forest swath as a result of aridification in east Africa during the Oligocene-early Miocene (c. 33-20 Myr) (Couvreur et al. 2008). The vast expanse of the relatively homogenous Congo basin lowland rainforest [Congolian Region of Linder et al. (2012) equating to the Guineo-Congolian biome of White (1983)] may prove to harbour fairly widespread species, and hence exhibit low degrees of endemism. The forest has, however, undergone numerous contractions, fragmentations and re-expansion from the mid-Tertiary onwards (c. 33–2 Myr) (Couvreur et al. 2008, Marks 2010) promoting speciation and endemism, and coupled with this high energy tropical ecosystem, cynipoid species richness could arguably be expected to be elevated in the Congo basin.

The majority of cynipoid species, world-wide, have unknown biological roles; the exception being Cynipidae where much of the taxonomy of the group is based on rearing records. Afrotropical cynipids are highly depauperate compared to the diverse northern hemisphere richness for the family, but their biology is relatively better known than their parasitoid counterparts in Africa. South Africa harbours a couple of enigmatic cynipids including an endemic, specialist lethal inquiline (van Noort et al. 2007) and gall formers of both herbs and trees (Liljeblad et al. 2011, Melika and Prinsloo 2007). *Phanacis* is poorly studied and indications, based on undescribed species at hand, are that the genus may be richer in the Cape Floral Kingdom than currently perceived. As mentioned previously, the hosts of Liopteridae remain uncertain, though some association with wood-boring beetles has been discussed extensively in the literature (Buffington et al. 2012). Figitids fall into three major categories of host preference: those associated with gall forming Hymenoptera (Euceroptrinae, Thrasorinae, Plectocynipinae, and Mikeiinae), those associated with aphid predators and parasitoids (Charipinae as hyperparasitoids, usually with braconid parasitic wasps as immediate hosts; Anacharitinae on Neuroptera; Aspicerinae on Diptera), and those associated with cyclorrhaphous Diptera (Figitinae and Eucoilinae, which compose the majority of Afrotropical cynipoid diversity). Hosts are completely unknown for Emargininae and Pycnostigminae; the former has been associated with driver ant refuse piles (Buffington et al. 2012), while the latter, based on phylogeny, has been speculated to attacking gall-forming Hymenoptera (Buffington and van Noort 2012).

Eucoilines can be further divided into two major divisions of host use: those which utilize leaf-miners (Agromyzidae) hosts up in the canopy; and those which attack Diptera in habitats like decomposing organic matter (debris, dung, carrion, wood etc), or algae and mushrooms (e.g. Ephydridae, Drosophilidae, Muscidae, Calliphoridae) on the ground. *Afrostilba* and *Nordlanderia* are the dominant leaf-miner parasitoids, with *Afrostilba* common along equatorial Africa as well as down the ‘southern swath’; *Nordlanderia* dominates in much more Mediterranean habitats, and these species are common in the Western Cape and along the southern coast of South Africa. Throughout sub-Saharan Africa, the drosophilid-parasitic genera *Leptopilina* and *Ganaspis* are extremely common, some of which may be tramp species that are cosmopolitan (Buffington and Forshage pers. obsv.). However, throughout sub-Saharan Africa, it is the chloropid, drosophilid, and ephydrid parasitic *Rhoptromeris* and *Hexacola* respectively that are frequently the most abundant in bulk samples. Furthermore, there are members of both of these genera, as well as the unusual *Nanocthulhu* and *Stentorceps*, that all possess some form of protrusions of the frons and/or clypeal region. The frequency of this condition in the Afrotropical region is unparalleled in other biogeographic regions (Buffington and Forshage pers. obs.). While some of these genera are related (*Nanocthulhu*, *Stentorceps*, and *Rhoptromeris*, all belonging to Trichoplastini), *Hexacola* belongs to Ganaspini, and the facial protrusions are therefore an example of convergence with these other genera. While we do not yet know the hosts for these species, we do know the facial traits are not sexually dimorphic; ergo, we speculate these structures are used in escaping from the host puparium, the surroundings of the puparium (e.g. soil), or both.

The authors hope this initial chapter on the Hymenoptera of the Afrotropical region marks a turning point in the larger understanding and appreciation of this incredibly diverse and important order of insects. The final AHI production will provide an essential resource for identification of Afrotropical Hymenoptera by a diverse array of end-users, from specialists, ecologists, and conservationists, to the applied forestry and agricultural sectors, enabling effective long-term conservation of an economically important and ecologically significant component of African and Madagascan ecosystems. Elucidating wasp systematics is a fundamental requirement for the future preservation of ecosystems that play an essential life support function for continued human survival.

## Supplementary Material

XML Treatment for Phanacis

XML Treatment for Rhoophilus

XML Treatment for Qwaqwaia

XML Treatment for Acanthaegilopsis

XML Treatment for Aegilips

XML Treatment for Anacharis

XML Treatment for Xyalaspis

XML Treatment for Anacharoides

XML Treatment for Aspicera

XML Treatment for Melanips

XML Treatment for Prosaspicera

XML Treatment for Alloxysta

XML Treatment for Apocharips

XML Treatment for Dilyta

XML Treatment for Phaenoglyphis

XML Treatment for Thoreauella

XML Treatment for Afrostilba

XML Treatment for Ealata

XML Treatment for Ganaspidium

XML Treatment for Gronotoma

XML Treatment for Nordlanderia

XML Treatment for Paradiglyphosema

XML Treatment for Afrodontaspis

XML Treatment for Bothrochacis

XML Treatment for Leptopilina

XML Treatment for Linoeucoila

XML Treatment for Trybliographa

XML Treatment for Aganaspis

XML Treatment for Didyctium

XML Treatment for Endecameris

XML Treatment for Ganaspis

XML Treatment for Gastraspis

XML Treatment for Hexacola

XML Treatment for Cothonaspis

XML Treatment for Kleidotoma

XML Treatment for Angustacorpa

XML Treatment for Nanocthulhu

XML Treatment for Rhoptromeris

XML Treatment for Stentorceps

XML Treatment for Trichoplasta

XML Treatment for Garudella

XML Treatment for Leptolamina

XML Treatment for Micreriodes

XML Treatment for Figites

XML Treatment for Lonchidia

XML Treatment for Neralsia

XML Treatment for Xyalophora

XML Treatment for Pycnostigmus

XML Treatment for Tylosema

XML Treatment for Ibalia

XML Treatment for Paramblynotus

XML Treatment for Oberthuerella

XML Treatment for Tessmannella

XML Treatment for Xenocynips

## Supplementary materials

Supplementary material 1 Supplementary material 1: Multi-entry, matrix-based identification key to the Afrotropical families of Cynipoidea (Hymenoptera) as exported from Lucid Builder 3.5 in XML SDD format Data type: multi-entry, matrix-based key in SDD format Explanation note: Multi-entry, matrix-based identification key to the Afrotropical families of Cynipoidea (Hymenoptera) as exported from Lucid Builder 3.5 in XML SDD format. This dataset is made available under the Open Database License (http://opendatacommons.org/licenses/odbl/1.0/). The Open Database License (ODbL) is a license agreement intended to allow users to freely share, modify, and use this Dataset while maintaining this same freedom for others, provided that the original source and author(s) are credited.Simon van Noort, Matthew L. Buffington, Mattias Forshage

Supplementary material 2 Multi-entry, matrix-based identification key to the Afrotropical genera of Cynipidae (Cynipoidea, Hymenoptera) as exported from Lucid Builder 3.5 in XML SDD format Data type: multi-entry, matrix-based key in SDD format Explanation note: Multi-entry, matrix-based identification key to the Afrotropical genera of Cynipidae (Cynipoidea, Hymenoptera) as exported from Lucid Builder 3.5 in XML SDD format. This dataset is made available under the Open Database License (http://opendatacommons.org/licenses/odbl/1.0/). The Open Database License (ODbL) is a license agreement intended to allow users to freely share, modify, and use this Dataset while maintaining this same freedom for others, provided that the original source and author(s) are credited.Simon van Noort, Matthew L. Buffington, Mattias Forshage

Supplementary material 3 Multi-entry, matrix-based identification key to the Afrotropical subfamilies of Figitidae (Cynipoidea, Hymenoptera) as exported from Lucid Builder 3.5 in XML SDD format Data type: multi-entry, matrix-based key in SDD format Explanation note: Multi-entry, matrix-based identification key to the Afrotropical genera of Figitidae (Cynipoidea, Hymenoptera) as exported from Lucid Builder 3.5 in XML SDD format This dataset is made available under the Open Database License (http://opendatacommons.org/licenses/odbl/1.0/). The Open Database License (ODbL) is a license agreement intended to allow users to freely share, modify, and use this Dataset while maintaining this same freedom for others, provided that the original source and author(s) are credited.Simon van Noort, Matthew L. Buffington, Mattias Forshage

Supplementary material 4 Multi-entry, matrix-based identification key to the Afrotropical genera of Anacharitinae (Figitidae, Cynipoidea, Hymenoptera) as exported from Lucid Builder 3.5 in XML SDD format Data type: multi-entry, matrix-based key in SDD format Explanation note: Multi-entry, matrix-based identification key to the Afrotropical genera of Anacharitinae (Figitidae, Cynipoidea, Hymenoptera) as exported from Lucid Builder 3.5 in XML SDD format. This dataset is made available under the Open Database License (http://opendatacommons.org/licenses/odbl/1.0/). The Open Database License (ODbL) is a license agreement intended to allow users to freely share, modify, and use this Dataset while maintaining this same freedom for others, provided that the original source and author(s) are credited.Simon van Noort, Matthew L. Buffington, Mattias Forshage

Supplementary material 5 Multi-entry, matrix-based identification key to the Afrotropical genera of Aspicerinae (Figitidae, Cynipoidea, Hymenoptera) as exported from Lucid Builder 3.5 in XML SDD format Data type: multi-entry, matrix-based key in SDD format Explanation note: Multi-entry, matrix-based identification key to the Afrotropical genera of Aspicerinae (Figitidae, Cynipoidea, Hymenoptera) as exported from Lucid Builder 3.5 in XML SDD format. This dataset is made available under the Open Database License (http://opendatacommons.org/licenses/odbl/1.0/). The Open Database License (ODbL) is a license agreement intended to allow users to freely share, modify, and use this Dataset while maintaining this same freedom for others, provided that the original source and author(s) are credited.Simon van Noort, Matthew L. Buffington, Mattias Forshage

Supplementary material 6 Multi-entry, matrix-based identification key to the Afrotropical genera of Charipinae (Figitidae, Cynipoidea, Hymenoptera) as exported from Lucid Builder 3.5 in XML SDD format Data type: multi-entry, matrix-based key in SDD format Explanation note: Multi-entry, matrix-based identification key to the Afrotropical genera of Charipinae (Figitidae, Cynipoidea, Hymenoptera) as exported from Lucid Builder 3.5 in XML SDD format. This dataset is made available under the Open Database License (http://opendatacommons.org/licenses/odbl/1.0/). The Open Database License (ODbL) is a license agreement intended to allow users to freely share, modify, and use this Dataset while maintaining this same freedom for others, provided that the original source and author(s) are credited.Simon van Noort, Matthew L. Buffington, Mattias Forshage

Supplementary material 7 Multi-entry, matrix-based identification key to the Afrotropical genera of Eucoilinae (Figitidae, Cynipoidea, Hymenoptera) as exported from Lucid Builder 3.5 in XML SDD format Data type: multi-entry, matrix-based key in SDD format Explanation note: Multi-entry, matrix-based identification key to the Afrotropical genera of Eucoilinae (Figitidae, Cynipoidea, Hymenoptera) as exported from Lucid Builder 3.5 in XML SDD format This dataset is made available under the Open Database License (http://opendatacommons.org/licenses/odbl/1.0/). The Open Database License (ODbL) is a license agreement intended to allow users to freely share, modify, and use this Dataset while maintaining this same freedom for others, provided that the original source and author(s) are credited.Simon van Noort, Matthew L. Buffington, Mattias Forshage

Supplementary material 8 Multi-entry, matrix-based identification key to the Afrotropical genera of Figitinae (Figitidae, Cynipoidea, Hymenoptera) as exported from Lucid Builder 3.5 in XML SDD format Data type: multi-entry, matrix-based key in SDD format Explanation note: Multi-entry, matrix-based identification key to the Afrotropical genera of Figitinae (Figitidae, Cynipoidea, Hymenoptera) as exported from Lucid Builder 3.5 in XML SDD format This dataset is made available under the Open Database License (http://opendatacommons.org/licenses/odbl/1.0/). The Open Database License (ODbL) is a license agreement intended to allow users to freely share, modify, and use this Dataset while maintaining this same freedom for others, provided that the original source and author(s) are credited.Simon van Noort, Matthew L. Buffington, Mattias Forshage

Supplementary material 9 Multi-entry, matrix-based identification key to the Afrotropical genera of Pycnostigminae (Figitidae, Cynipoidea, Hymenoptera) as exported from Lucid Builder 3.5 in XML SDD format Data type: multi-entry, matrix-based key in SDD format Explanation note: Multi-entry, matrix-based identification key to the Afrotropical genera of Pycnostigminae (Figitidae, Cynipoidea, Hymenoptera) as exported from Lucid Builder 3.5 in XML SDD format This dataset is made available under the Open Database License (http://opendatacommons.org/licenses/odbl/1.0/). The Open Database License (ODbL) is a license agreement intended to allow users to freely share, modify, and use this Dataset while maintaining this same freedom for others, provided that the original source and author(s) are credited.Simon van Noort, Matthew L. Buffington, Mattias Forshage

Supplementary material 10 Multi-entry, matrix-based identification key to the Afrotropical genera of Liopteridae (Cynipoidea, Hymenoptera) as exported from Lucid Builder 3.5 in XML SDD format Data type: multi-entry, matrix-based key in SDD format Explanation note: Multi-entry, matrix-based identification key to the Afrotropical genera of Liopteridae (Cynipoidea, Hymenoptera) as exported from Lucid Builder 3.5 in XML SDD format This dataset is made available under the Open Database License (http://opendatacommons.org/licenses/odbl/1.0/). The Open Database License (ODbL) is a license agreement intended to allow users to freely share, modify, and use this Dataset while maintaining this same freedom for others, provided that the original source and author(s) are credited.Simon van Noort, Matthew L. Buffington, Mattias Forshage
